# Synthesis and enzyme-based evaluation of analogues *L*-tyrosine thiol carboxylic acid inhibitor of metallo-β-lactamase IMP-1

**DOI:** 10.1080/14756366.2019.1651314

**Published:** 2019-08-11

**Authors:** Omid Khalili Arjomandi, Mahboubeh Kavoosi, Hadi Adibi

**Affiliations:** a Pharmaceutical Sciences Research Center, Health Institute, Kermanshah University of Medical Sciences, Kermanshah, Iran;; b The University of Queensland, School of Chemistry and Molecular Biosciences, Brisbane, Australia;; c Department of Biochemistry, Pasteur Institute of Iran, Tehran, Iran

**Keywords:** β-lactam, β-antibiotic resistance, metallo-β-lactamase, inhibitor, computational modelling

## Abstract

The emergence of drug-resistant pathogenic bacteria is occurring due to the global overuse and misuse of β-lactam antibiotics. Infections caused by some bacteria which secrete metallo-β-lactamases (enzymes that inactivate β-lactam antibiotics) are increasingly prevalent and have become a major worldwide threat to human health. These bacteria are resistant to β-lactam antibiotics and MBL-inhibitor/β-lactam antibiotic combination therapy can be a strategy to overcome this problem. So far, no clinically available inhibitors of metallo-β-lactamases (MBLs) have been reported. In this study, *L*-benzyl tyrosine thiol carboxylic acid analogues (**2a–2k**) were synthesized after the study of computational simulation by adding of methyl, chloro, bromo and nitro groups to the benzyl ring for investigation of SAR analysis. Although the synthesized molecules **2a–k** shows the potent inhibitory effects against metallo-β-lactamase (IMP-1) with the range of *K_ic_* values of 1.04**–**4.77 µM, they are not as potent as the candidate inhibitor.

## GRAPHICAL ABSTRACT

**Figure:**
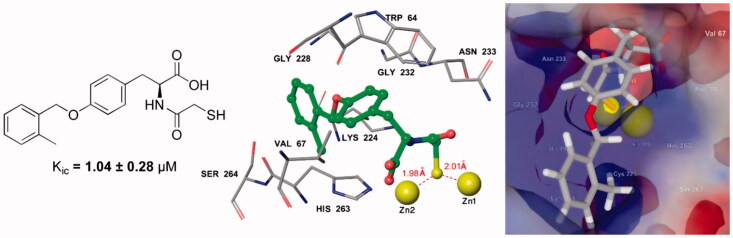

## Introduction

The β-lactam antibiotics are the most frequently prescribed drugs used in the treatment of bacterial infections since the 1940s. The β-lactam antibiotic resistance crisis has been attributed to the global overuse and misuse of these medications. The study of classifications β-lactam antibiotics and mechanisms of their inactivation by resistant bacteria can assist scientists to design a strategy to overcome this problem. So far, the β-lactamases have been classified to serine-β-lactamases (SBLs, classes A, C and D) and metallo-β-lactamases (MBLs, class B) based on their mechanisms of action. Moreover, MBLs are further divided into three subclasses, B1, B2 and B3, which differ in their amino acid sequences and metal occupancies.^1–7^ A fourth MBL subclass B4 has been tentatively identified as well.^8–9^ What MBLs have in common is having an active site that is mostly occupied by two zinc ions and Table-1 summarise amino acid types chelated into Zinc ions.^10–18^

**Figure 1.**
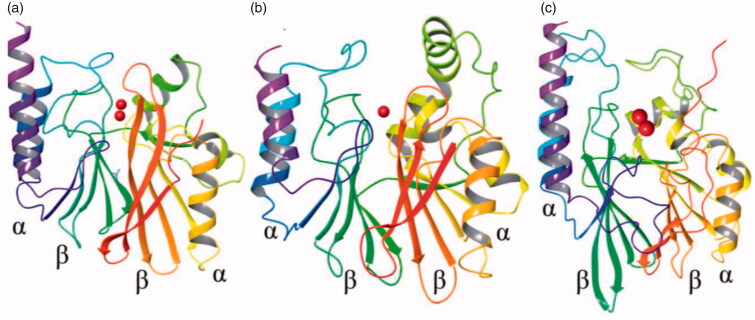
3D structures representing MBLs. The α-helices are shown in purple and yellow, β-sheets in green and red and Zn^2+^ ions as red spheres. (a) Subclass B1, IMP-1 (PDB code: 1JJT). (b) Subclass B2, CphA (PDB code: 1X8G). (c) Subclass B3, L1 (PDB code: 1SML).

In total, all MBLs are characterised by αββα quaternary structural fold and zinc ions of the active sites are located at the edge of the ββ sheets at a distance measurement range nearly 3.4–4.4 Å^19^ (Figure 1).

**Figure 2.**
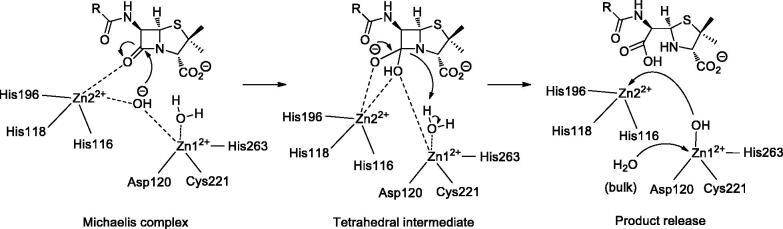
The proposed mechanism of hydrolysis of β-lactam antibiotics by IMP-1.

In this research, only IMP-1, a subclass B1 enzyme, was only investigated. IMP-1 is secreted by bacteria such as *Pseudomonas aeruginosa* and *Serratia marcescens*. In the active site of this enzyme, Zinc 1 is coordinated by a water molecule and three identical amino acids (His116, His118 and His196) with tetrahedral arrangement while Zinc 2 is coordinated by two water molecules and three different amino acids (Asp120, Cys221 and His263) in distorted trigonal bipyramidal geometry. The proposed mechanism in Figure 2 shows that how the activated water molecule starts the process of hydrolysis of β-lactam antibiotics and convert them to useless drugs.^20–22^

One strategy to overcome this problem is to find a potent inhibitor that can attach to the active site of IMP-1 and prevent the hydrolysis of the β-lactam rings of antibiotics. Although numerous MBLs inhibitors have been reported in the literature, there is no clinically available inhibitor. Moreover, none of the clinically useful serine-β-lactamases inhibitors, such as clavulanic acid, which is co-administered with penicillin is not effective against MBLs.^23^ Therefore, research and discovery of a clinically MBLs inhibitor is still in progress. So far, various inhibitors of MBLs have been reported in different categories such as thiols,^24–30^ sulphates,^31^ dicarboxylic acids,^32–37^ trifluoromethylketones and alcohols,^38^ hydroxamates,^39^ tetrazoles,^40^ sulphonamides and sulphonyl hydrazones.^41^ Compounds containing a zinc-binding sulphur atom comprise the first category of MBLs inhibitors.^7^

**Figure 3.**
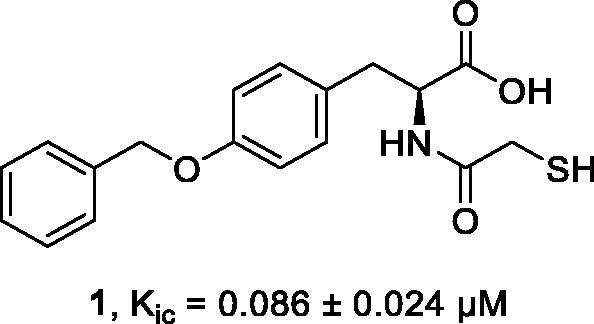
The chemical structure and *K_ic_* value of inhibitor **1** against IMP-1.

Thiol carboxylic acid compounds have been reported as the most common inhibitors of IMP-1.^7^ For instance, thiomandelic acid and 2-mercapto-5-phenylpentanoic acid were discovered as a broad-spectrum inhibitor for MBLs where Cd-NMR studies demonstrated that the sulphur atom of the compound intercalates the metal ions within the active site and displaces the bridging water molecule that is essential for the MBL hydrolase activity. Further potent thiol containing compound reported with good IC50 by Büttner et al.^42^ The other potent thiol carboxylic acid compounds, bisthiazolidines, reported by González et al.^43^ with *Ki* values in a range of 7**–**19 µM against NDM-1. The co-crystallised enzyme/inhibitors of these compounds demonstrated that the sulphur atom of the mercaptomethyl group is positioned nearly equidistant between the two zinc ions, displacing the bridging water/hydroxide molecule that is proposed to be the attacking nucleophile, but with little impact on the Zn1 − Zn2 distance.^43^ Further bisthiazolidines derivatives reported by Hinchliffe et al.^44^ with a broader range of assay on MBLs. The result of inhibition study is fascinating as these molecules capable of inhibition of all three subclasses with a highest *Ki* value of 41 µM against GOB-18.^44^ These compounds were very potent against IMP-14 with *Ki* values in a range of 6**–**14 µM. Arjomandi et al.^1^ also demonstrated L-benzyl tyrosine thiol carboxylic acid (Figure 3) as a potent MBL inhibitor with the *Kic* value of 86 nM against IMP-1. In this study, this inhibitor was used as a candidate molecule for further modification and development with the aim of improving inhibition activity.

**Figure 4.**
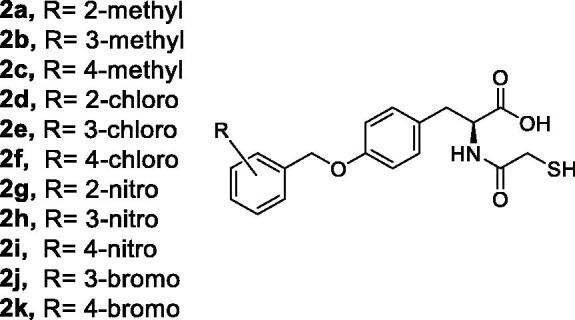
The chemical structures of analogues *L*-thiol carboxylic acid.

Various modifications on the metal chelating inhibitor **1** can be exerted and investigated for the aim the study of improving inhibition activity. The study of computational modelling predicts the thiol group of the candidate inhibitor is coordinated to both Zinc ions of the active site of the enzyme which is probably one of the key interactions for strong chelating. Therefore, the substitution of the carboxylic acid group with a thiol moiety may improve the potency of the inhibitory activity as there will be two thiol groups for metal chelating. On the other hand, computational simulation is only a predicted model and does not reveal the real configuration of the ligand and its interactions with the amino acids of the active site, thus, the replacement of the thiol group with the carboxylic acid can also be investigated when the modified molecule would have two carboxylic acid groups. The alternative modification is the substitution of hydrogen(s) of the methylene groups of the skeleton with the electron-donating and electron-withdrawing groups or hydrogen acceptor and donor moieties and examination of their interactions with the amino acids of the active site for the study of a better fixing in the active site. The subsequent modification is the substituting of the central phenyl ring with heteroaromatic rings which may lead to a better fixing of the molecule into the active site due to the creation of further interactions like hydrogen bond(s). Moreover, removing either one or two methylene groups of the skeleton reduce the size of the molecule that may create a better conformation and configuration with the creation of stronger interactions with Zinc ions or amino acids of the active site. The other modification is the study of addition of hydrogen acceptor and donor groups or electron withdrawing or electron donating moieties on either central phenyl ring or the tale benzyl ring. In this study, the latest modification was investigated by the addition of methyl, chloro, bromo and nitro groups to the benzyl ring of the candidate molecule (Figure 4) and in silico modelling was used for the prediction of ligand binding into the active site of IMP-1.

### Computational modelling

**Figure 5.**
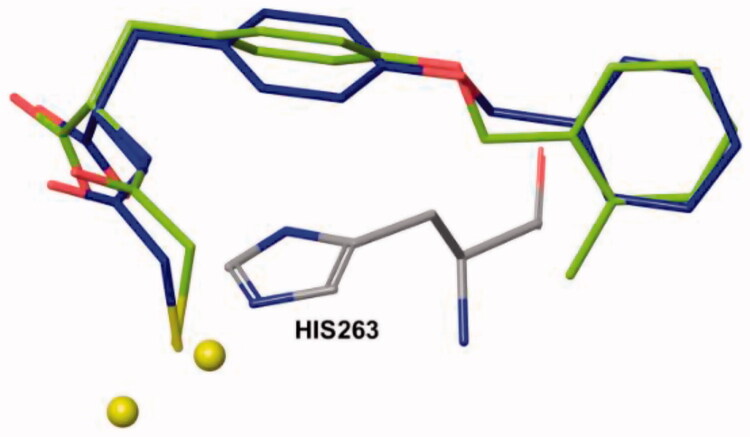
The typical superimposed of docked inhibitor **2a** (green) and the reference inhibitor **1** (blue) into the active site of IMP-1.

**Figure 6.**
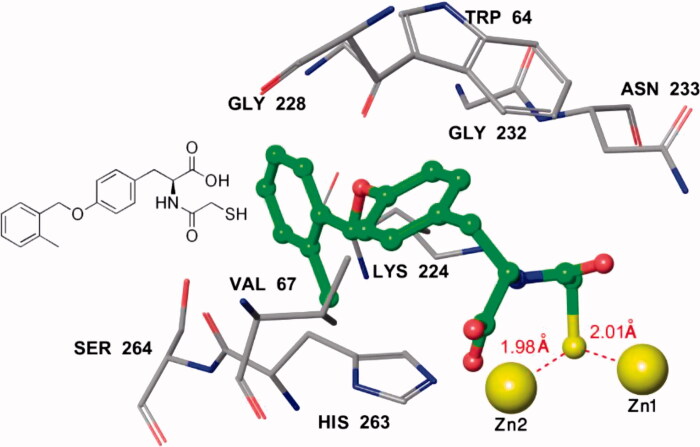
Interaction of pose **2a** at the active site of IMP-1 (PDB: 1JJT). Atom colours are as follows: blue – nitrogen, red – oxygen, grey – carbon and yellow − zinc atom (of IMP-1), and red – oxygen, blue – nitrogen, green – carbon, and yellow – sulphur (on inhibitor).

For the study of computational modelling, the crystal structure of IMP-1 enzyme (PDB code: 1JJT) was downloaded from Protein Data Bank (PDB) and Chain B was deleted as the topologies of the active sites of both chains A and B are identical. Further elimination for chain A was carried out by removing water molecules (solvents), single Zinc ion and the coordinated ligand of the active site. The cavity of the active site with two zinc ions was set by MolDock for the investigation of docking study. Compounds **2a–2k** and inhibitor **1** as the reference molecule were docked into the active site of IMP-1 and the study of their coordination indicates that they almost have similar configurations and conformation in the active site with slightly rotation of the central phenyl ring but are still flanked between Trp64 and His263 which may create π**–**π stacking interactions (Figure 5). The calculated docking energies (*E_score_*) of all analogues were higher and better than the lead compound. On the other hand, the predicted distance of the coordination of the thiol group of most analogues to Zinc 2 is shorter than Inhibitor **1** which can be considered as one of the key factors for stronger chelation. The predicted distance between Zinc 1 and the thiol group of target molecules and the reference ligand is nearly unaffected (Table 1). The docking also anticipates the creation of hydrogen bond between carboxylic acid group and HIS263 for all molecules including the reference compound (Figure 6). Altogether and based on the energy of the computational modelling of analogues and prediction of stronger binding to one of Zinc ions, the molecules were synthesized and their *in vitro* inhibitions were measured against IMP-1.

**Table 1.** Subclasses of MBLs and their active site amino acids that chelated Zinc ions.

Subclass	Examples	Zinc–amino acid ligands		Involvement of Zn2+ ions for catalytic activity
		Zn1 ligands	Zn2 ligands	
B1	IMP-1, CcrA, VIM, NDM-1, BcII	His 116	Asp 120	Mainly di-zinc but BcII is active with either one or two Zn^2+^ ions
		His 118	Cys 221	
		His 196	His 263	
B2	CphA, Imis	Asn 116	Asp 120	Mono-zinc (Zn2 site) but binding of Zn1 inhibits the MBL
		His 118	Cys 221	
		His 196	His 263	
B3	L1 (tetramer), FEZ-1, GOB-18	His/Gln 116	Asp 120	Di-zinc
		His 118	His 121	
		His 196	His 263	

### Enzyme kinetics studies

**Figure 7.**
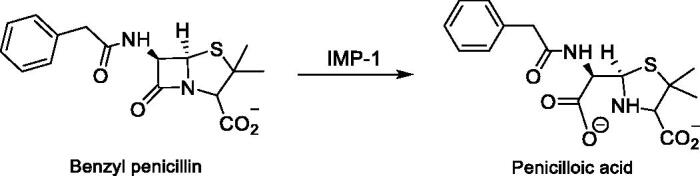
Inactivation of benzyl penicillin by MBLs.

The IMP-1 enzyme, lacking the first 21 signal peptide amino acid residues, was expressed and purified using the protocol of Vella et al.^45–46^ The reported methods for monitoring of MBLs activities are based on the consequences of the hydrolysis of β-lactam ring of substrates. The MBL-catalysed reaction is usually monitored by following the changing absorbance at the wavelength where the difference in the molar absorptivity, ΔɛM, is maximal or near the maximum value. There are a few β-lactam substrates which can be used for *in vitro* assays of MBLs inhibitors. CENTA,^47^ a type of cephalosporin, and Penicillin G (benzyl penicillin)^48^ are well-known substrates due to commercial availability, inexpensive prices and comfort of their syntheses. For this study, Penicillin G was used as CENTA was not compatible with the inhibitor type as reported by Arjomandi et al.^1^ The monitoring of hydrolysis of benzyl penicillin by the synthesized compounds was carried out in UV plate at wavelength 235 nm as no chromosphere is released (Figure 7). The concentration of benzyl penicillin is reduced upon hydrolysis by IMP-1 causing a decrease in the absorption at 235 nm over the time, thereby allowing a way to monitor the reaction. Inhibitors of metallo-β-lactamase decrease the rate of hydrolysis of benzyl penicillin, thus, providing a way of measuring the activities of inhibitors against MBLs.

Benzyl penicillin solutions were prepared fresh at the time of assay as their activities will change and stop after 24 h even with keeping in the fridge.^1^ In the first step, the inhibitory activities (IC_50_ values) of the inhibitors **2a–k** were measured by a screening assay which evaluated the residual activity of the enzyme. The assay results showed that all compounds **2a–k** exhibited approximately 50% inhibition at the range of concentration of 1**–**5 µM. Further kinetic analyses then allowed the determination of *K_ic_* values of these compounds based on the procedure reported by Arjomandi et al.^1^ Inhibition assays for IMP-1 were performed in 96 well 400 µL UV multi-titre plates using a UV multi-plate spectrophotometer. The hydrolysis rate of benzyl penicillin (penicillin G sodium salt) was measured at *λ* = 235 nm.^49^ The initial screening assay (IC_50_ values) was performed in duplicate, with benzyl penicillin as the substrate and HEPESX (50 mM HEPES, 0.1 M NaCl, 100 mM ZnCl_2_, pH 7.0) as the buffer at 25 °C. The final concentrations of enzyme and benzyl penicillin were 10 nM and 500 µM, respectively. Bovine serum albumin (a final concentration of 20 µg/mL) was added to the enzyme for stability. For each well, 196 µL substrate and 4 µL inhibitor were added followed by the addition of 200 µL of enzyme. The rate of the hydrolysis of substrate was recorded for 5 min. To measure *K_ic_* value, four solutions with different concentrations for each molecule were prepared and then were assayed based on the aforementioned procedure while the concentrations of substrate in wells were 200, 400, 600, 800 and 1000 µM. The raw kinetic data were analysed by non-linear regression using the Win Curve Fit program (Kevin Raner Software) and the *K_ic_* value was calculated for each molecule.^1^ The *K_ic_* values of molecules **2a–k** are in the range of 1.04**–**4.77 µM which was unexpected based on the computational modelling results. Although the introduced compounds are still potent enzyme-based inhibitors, they are nearly at least ten times less as potent as the lead inhibitor is. Due to the less inhibition activities of the synthesized molecules in compare to the reference compound, their cell-based assays have not carried out.

Among the reported molecules, compounds with methyl groups **2a–c** are better enzyme-based inhibitors when compared compounds **2d–k**. In particular *ortho* and *para* substituted compounds **2a** and **2c** are similar and better than *meta*-substituted compound **2b** which indicates the influence of electron donating group. Although the docking poses failed to show any interaction of the benzyl group in the receptor site, apparently a monosubstituted electron donating group on benzyl ring can lead to a better and stronger affinity of the inhibitor in the active site while electron withdrawing one decrease that affinity.

The achieved experimental results indicate that all compounds **2a–k** are potent inhibitors against IMP-1 and the *ortho*-substituted compounds on benzyl ring show the highest inhibitory activities than *meta*- and *para*-substituted ones (Table 2).

**Table 2.** The computational modelling results and *K_ic_* values of inhibitor 1 and compounds 2a–k.

Compound	MolDock score (kcal/mol)	Distance (Å) between Zn1 ion and SH	Distance (Å) between Zn2 ion and SH	*K_ic_* (µM)
1	−190.9	1.93	1.94	0.086 ± 0.024
2a	−201.1	2.04	1.85	1.04 ± 0.28
2b	−202.9	1.93	1.96	1.15 ± 0.49
2c	−194.1	1.94	1.90	1.07 ± 0.21
2d	−197.3	2.04	1.85	1.81 ± 0.90
2e	−194.2	1.94	1.90	4.77 ± 2.81
2f	−195.5	2.03	1.88	3.20 ± 0.59
2g	−201.9	1.94	1.91	1.65 ± 0.34
2h	−199.8	1.94	1.90	2.86 ± 1.23
2i	−207.0	1.94	1.90	3.20 ± 0.62
2j	−199.5	1.93	1.95	3.14 ± 0.71
2k	−194.1	1.93	1.94	3.30 ± 0.90

To compare the information of the computational modelling and the achieved experimental data, the *K_ic_* values were compared with the predicted calculated energy and predicted distances of Zinc ions (Table 2). Although all compounds **2a–k** were potent inhibitors against IMP-1, the trend of comparison of theoretical energy did not match with the activities of inhibitors. Therefore, adding substituents to any position of the benzyl ring of the reference compound **1** apparently leads to a decrease in their inhibitory activities which can described based on the restricted rotation of the benzyl group of the inhibitor(s). Consequently, the addition of either electron donor or electron withdrawing group to the benzyl ring can decrease the potency of inhibitors against IMP-1.

### Chemistry

**Scheme 1:**
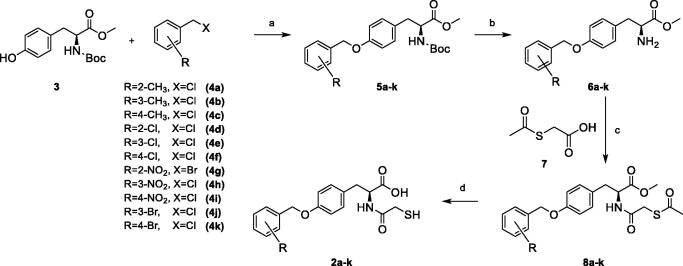
Reagents and conditions: (a) Acetone, K_2_CO_3_, *Δ*, 24 h; (b) TFA, CHCl_3_, r.t, overnight, 98%; (c) Compound **7**, HBTU, DIPEA, THF, r.t, 24 h; (d) NaOH, H_2_O/MeOH, r.t, 1 h, under nitrogen.

The synthesis pathway of compounds **2a–2k** was similar to what had been reported by Arjomandi et al.^1^ However, the purchased Boc-protected *L*-tyrosine methyl ester **3** was used as the precursor to reduce the number of steps in the synthesis (Scheme 1). Compounds **5a–k** were synthesized by Williamson ether synthesis procedure with refluxing a mixture of Boc *L*-tyrosine methyl ester **3**, compounds **4a–k** and potassium carbonate in acetone with the isolated yields over 80%.^50^ The BOC group then was removed by adding compounds **5a–k** into the mixture of TFA in chloroform to produce compounds **6a–k** with the isolated yields of 98%.^51^ Compound **7** (acetylthio acetic acid) was synthesized by the procedure that was reported by Arjomandi et al.^1^ The amidation of *L*-amino acid methyl esters **6a–k** and acetylthio acetic acid **7** was carried out by HBTU as a coupling reagent and DIPEA as a base to produce compounds **8a–k** which then purified by flash column chromatography.^52^ One of the by-products of HBTU is tetramethyl urea which is formed in the reaction and its ^1^H NMR signal appears at 2.7 ppm. To remove this impurity, running a mixture of 30–70% petroleum ether**–**ethyl acetate solvent in flash column chromatography can take out tetra methyl urea as well as other impurities. The monitoring of the fraction samples by TLC indicated that no product(s) is available. The final products then were collected with increase the percentage of the polar component of the solvent. Intermediates **8a–k** are new compounds, and their ^1^H NMR and ^13 ^C-NMR spectra (as observed by the appearance of the characteristic thioacetyl methyl group) as well as LRMS and HRMS (positive mode) were recorded and confirmed the constitutions of molecules. The final hydrolysed compounds **2a–k** were prepared by the treatment of intermediates **8a–k** with sodium hydroxide solution under nitrogen to give the final thiol carboxylic acid products (Scheme 1 This is extremely important for the final hydrolysis reaction to be carried out under nitrogen as thiol carboxylic acids are sensitive to aerial oxidation. In addition, the isolated compounds **2a–k** need to be kept in the sealed container as well. Compounds **2a–k** are new and ^1^H NMR and ^13^C-NMR spectra (by evaluating of the disappearance of acetyl and methyl groups and the appearance of SH and COOH groups), as well as LRMS and HRMS (negative mode), were recorded and confirmed the constitutions of molecules.

## Conclusions

We described a straightforward and efficient synthesis of analogues of *L*-benzyl tyrosine thiol carboxylic acid **1**. These compounds demonstrated potent enzyme-based *in vitro* inhibitory activities against IMP-1 with *K_ic_* values of ranging 1.04 ± 0.28**–**4.77 ± 2.81 µM but they were less potent than the lead compound. The compounds with electron donating group to *ortho*, *meta* and *para* of the benzyl ring of candidate molecule show better inhibition than electron withdrawing one. The addition of substitution to the benzyl ring of the potent cell-based inhibitor **1** decreases its activity against IMP-1.

## Experimental

### Compound characterisation. General information

All chemicals were purchased from Sigma-Aldrich (Chicago, IL), Merck (Kenilworth, NJ) and Fluka Chemical Companies (Milwaukee, WI). All NMR experiments were recorded on Bruker AVANCE 500, 400 or 300 MHz spectrometers (Billerica, MA). Chemical shifts are reported in parts per million (ppm) on a *δ* scale and referenced to the residual solvent peak (^1^H, 7.24 ppm, ^13 ^C, 77.0 ppm for CDCl_3_; ^1^H, 2.49 ppm, ^13 ^C, 39.5 ppm for DMSO-d_6_; ^1^H, 3.30 ppm, ^13 ^C, 49.5 ppm for CD_3_OD). Coupling constants (*J*) are reported in Hertz. Multiplicities of the peaks are abbreviated as follows: s for singlet, bs for broad singlet, d for doublet, t for triplet and q for quartet. Low- and high-resolution EI-MS were measured on a Finnigan MAT 900 XL-Trap mass spectrometer in the positive and the negative ionisation mode. LR-ESI were recorded on a Bruker HCT 3D Ion Trap and HR-ESI were performed on a BrukerMicrOTof-Q with the DIONEX Ultimate 3000 LC in the positive and the negative electrospray ionisation mode, with CH_3_OH as a solvent. For IR analyses, samples were measured neat on a PerkinElmer Frontier FT-IR spectrometer (PerkinElmer, Waltham, MA).

### Synthesis

#### General procedure 1: Williamson ether synthesis

The general procedure reported by Wolf et al.^50^ was used for the synthesis of compounds **5a–k**. A mixture of *N*-Boc-*L*-tyrosine methyl ester (2.0 g, 6.8 mmol), benzyl derivative **4a–k** (13.5 mmol), potassium carbonate (1.87 g, 13.5 mmol) and sodium iodide (100 mg as the catalytic amount) in acetone (50 mL) was heated under reflux, under nitrogen, for 24 h. The resulting mixture was then filtrated and evaporated under the vacuum to give the crude oil which was purified by flash chromatography (30–100% EtOAc in petroleum ether).

#### General procedure 2: Boc deprotection

The general procedure reported by Benthem et al.^51^ was used for the synthesis of compounds **6a–k**. A mixture of *L*-Boc protected compounds **5a–k** (5 mmol), chloroform (30 mL) and TFA (1.14 g, 0.76 mL, 10 mmol) was stirred at room temperature, and the progress of the reaction was monitored by TLC. Then the organic solution was washed with saturated sodium bicarbonate (30 mL), dried over sodium sulphate and evaporated under the vacuum to give the products **6a–k** which was used for the next step without further purification.

#### General procedure 2: Amide coupling reactions

The general method reported by Knorr et al.^52^ was used for the synthesis of compounds **8a–k**. A mixture of deprotected Boc compounds **6a–k** (4.0 mmol), 2-(acetylthio)acetic acid **7** (0.59 g, 4.4 mmol), HBTU (1.67 g, 4.4 mmol) and DIPEA (1.56 g, 2.10 mL, 12.00 mmol) in THF (30 mL) was stirred at room temperature for 24 h. Then the organic solvent was evaporated under the vacuum, and the residue was dissolved in DCM (50 mL). The organic phase was then washed with saturated sodium bicarbonate (2 × 50 mL) and evaporated under the vacuum to give the crude oil which was purified by flash chromatography (30–100% EtOAc in petroleum ether) to give the pure products **8a–k**.

#### General procedure 3: Saponification

Compounds **2a–k** were synthesized using the following general procedure: compounds **8a–k** (3.0 mmol) were dissolved in methanol (10 mL), then sodium hydroxide solution (1 M, 10 mL) was added and the solution was stirred under nitrogen for an hour. The organic solvent (methanol) was evaporated under the vacuum, then water (50 mL) was added and the combined water phase was washed with ethyl acetate (50 mL) and DCM (50 mL) under nitrogen. Then pH was adjusted to 1 using concentrated HCl. The product in the water phase was extracted with ethyl acetate (50 mL) and washed with water (5 × 50 mL)^1^ to remove acetic acid. The organic phase was dried over sodium sulphate, filtered and evaporated under the vacuum to give the pure product **2a–k**. All ^1^H NMR, ^13 ^C NMR, LRMS and HRMS (negative mode) spectra were recorded.

#### Analytical information of synthesized molecules

##### (*S*)-Methyl 2-((tert-butoxycarbonyl)amino)-3–(4-((2-methylbenzyl)oxy)phenyl)propanoate (**5a**)

Pale yellow viscous oil, 2.17 g (80%). ^1^H NMR (500 MHz, CDCl_3_): *δ* 7.34 (1H, d, *J =* 7.1 Hz, Ar-H), 7.26–7.16 (3H, m, Ar-H), 7.03 (2H, d, *J =* 8.5 Hz, Ar-H), 6.89 (2H, d, *J =* 8.5 Hz, Ar-H), 4.98 (2H, s, CH_2_O), 4.96 (1H, d-broad, *J =* 7.5 Hz, NH), 4.54 (1H, q, *J =* 6.9 Hz, CH_2_CHNH), 3.70 (3H, s, CH_3_O), 3.04 (1H, dd, *J* = 13.8, 5.8 Hz, CH_A_H_B_CHNH), 2.99 (1H, dd, *J* = 13.8, 5.8 Hz, CH_A_H_B_CHNH), 2.35 (3H, s, CH_3_Ph), 1.40 (9H, s, C(CH_3_)_3_). ^13 ^C NMR (125 MHz, CDCl_3_): *δ* 172.4 (CO_2_CH_3_), 158.0 (CO_2_Boc), 155.1 (Ar-C), 136.7 (Ar-C), 134.7 (Ar-C), 130.4 (Ar-C), 130.2 (Ar-C), 128.6 (Ar-C), 128.2 (Ar-C), 128.2 (Ar-C), 126.0 (Ar-C), 114.8 (Ar-C), 79.9 (C(CH_3_)_3_**)**, 68.6 (CH_2_O), 54.5 (CH_2_CHNH), 52.1 (CH_3_OCO), 37.5 (CH_2_CHNH), 28.3 (C(CH_3_)_3_), 18.8 (CH_3_Ph). HRMS calculated for C_23_H_29_NNaO_5_ [M + Na]^+^ 422.1938, found 422.1939.

##### (*S*)-Methyl 2-amino-3–(4-((2-methylbenzyl)oxy)phenyl)propanoate (**6a**)

Orange solid, 1.47 g (98%). ^1^H NMR (500 MHz, CDCl_3_): *δ* 7.39 (1H, d, *J =* 7.1 Hz, Ar-H), 7.27–7.17 (3H, m, Ar-H), 7.11 (2H, d, *J =* 8.5 Hz, Ar-H), 6.93 (2H, d, *J =* 8.5 Hz, Ar-H), 5.00 (2H, s, CH_2_O), 3.71 (3H, s, CH_3_O), 3.70 (1H, t, *J =* 6.6 Hz, CH_2_CHNH), 3.04 (1H, dd, *J* = 13.8, 5.2 Hz, CH_A_H_B_CHNH), 2.84 (1H, dd, *J* = 13.8, 7.7 Hz, CH_A_H_B_CHNH), 2.36 (3H, s, CH_3_Ph), 1.97 (2H, bs, NH_2_). ^13 ^C NMR (125 MHz, CDCl_3_): *δ* 175.3 (CO), 157.9 (Ar-C), 136.6 (Ar-C), 134.7 (Ar-C), 130.3 (Ar-C), 130.3 (Ar-C), 129.2 (Ar-C), 128.6 (Ar-C), 128.2 (Ar-C), 126.0 (Ar-C), 114.8 (Ar-C), 68.6 (CH_2_O), 55.8 (CH_2_CHNH), 52.0 (CH_3_O), 39.9 (CH_2_CHNH), 18.8 (CH_3_Ph). HRMS calculated for C_18_H_22_NO_3_ [M + H]^+^ 300.1594, found 300.1595.

##### (*S*)-Methyl 2-(2-(acetylthio)acetamido)-3-(4-((2-methylbenzyl)oxy)phenyl)propanoate (8a)

Pale yellow viscous oil, 1.33 g (80%). ^1^H NMR (400 MHz, CDCl_3_): *δ* 7.37 (1H, d, *J =* 7.1 Hz, Ar-H), 7.27–7.17 (3H, m, Ar-H), 6.99 (2H, d, *J =* 8.5 Hz, Ar-H), 6.88 (2H, d, *J =* 8.5 Hz, Ar-H), 6.60 (1H, d, *J =* 7.7 Hz, NH), 4.98 (2H, s, CH_2_O), 4.77 (1H, dt, *J* = 7.7, 5.8 Hz, CH_2_CHNH), 3.71 (3H, s, CH_3_O), 3.52 (2H, dd, *J =* 25.0, 15.0 Hz, CH_2_S), 3.08 (1H, dd, *J* = 13.8, 5.5 Hz, CH_A_H_B_CHNH), 3.00 (1H, dd, *J* = 13.8, 6.1 Hz, CH_A_H_B_CHNH), 2.35 (3H, s, CH_3_CO), 2.32 (3H, s, CH_3_Ph). ^13 ^C NMR (100 MHz, CDCl_3_): *δ* 195.2 (COS), 171.6 (CO_2_), 167.6 (CONH), 158.0 (Ar-C), 136.6 (Ar-C), 134.7 (Ar-C), 130.4 (Ar-C), 130.3 (Ar-C), 128.6 (Ar-C), 128.3 (Ar-C), 127.8 (Ar-C), 126.0 (Ar-C), 114.8 (Ar-C), 68.5 (CH_2_O), 53.5 (CH_2_CHNH), 52.3 (CH_3_O), 36.7 (CH_2_CHNH), 32.7 (CH_2_S), 30.1 (CH_3_CO), 18.8 (CH_3_Ph). HRMS calculated for C_22_H_25_NNaO_5_S [M + Na]^+^ 438.1346, found 438.1349.

##### (*S*)-2-(2-Mercaptoacetamido)-3-(4-((2-methylbenzyl)oxy)phenyl)propanoic acid (**2a**)

Off white solid, 0.66 g (61%). ^1^H NMR (400 MHz, CD_3_OD): *δ* 7.34 (1H, d, *J =* 7.3 Hz, Ar-H), 7.24–7.10 (5H, m, Ar-H), 6.99 (2H, d, *J =* 8.5 Hz, Ar-H), 5.00 (2H, s, ArCH_2_O), 4.62 (1H, dd, *J =* 8.3, 5.3 Hz, CH_2_CHNH), 3.14 (1H, dd, *J* = 14.0, 6.0), 313 (2H, s, CH_2_SH), 2.95 (1H, dd, *J* = 14.0, 8.2 Hz, CH_A_H_B_CHNH), 2.33 (3H, s, CH_3_). ^13 ^C NMR (100 MHz, CD_3_OD): *δ* 174.4 (CO_2_H), 172.9 (CONH), 159.3 (Ar-C), 138.0 (Ar-C), 136.4 (Ar-C), 131.5 (Ar-C), 131.3 (Ar-C), 130.3 (Ar-C), 129.7 (Ar-C), 129.2 (Ar-C), 126.9 (Ar-C), 115.8 (Ar-C), 69.7 (PhCH_2_O), 55.3 (CH_2_CHNH), 37.5 (CH_2_CHNH), 28.0 (CH_2_SH), 18.9 (CH_3_). HRMS calculated for C_19_H_20_NO_4_S [M-H]^−^ 358.1119, found 358.1125.

##### (*S*)-Methyl 2-((tert-butoxycarbonyl)amino)-3-(4-((3-methylbenzyl)oxy)phenyl)propanoate (**5b**)

Pale yellow viscous oil, 2.17 g (80%). ^1^H NMR (500 MHz, CDCl_3_): *δ* 7.28–7.17 (3H, m, Ar-H), 7.12 (1H, d, *J =* 7.4 Hz, Ar-H), 7.02 (2H, d, *J =* 8.5 Hz, Ar-H), 6.88 (2H, d, *J =* 8.5 Hz, Ar-H), 4.98 (2H, s, CH_2_O), 4.94 (1H, d, *J =* 7.5 Hz, NH), 4.33 (1H, q, *J =* 6.9 Hz, CH_2_CHNH), 3.69 (3H, s, CH_3_O), 3.07–2.93 (2H, m, CH_2_CHNH), 2.35 (3H, s, CH_3_Ph), 1.40 (9H, s, C(CH_3_)_3_). ^13 ^C NMR (125 MHz, CDCl_3_): *δ* 172.4 (CO_2_CH_3_), 158.0 (CO_2_Boc), 155.1 (Ar-C), 138.3 (Ar-C), 136.9 (Ar-C), 130.3 (Ar-C), 128.7 (Ar-C), 128.5 (Ar-C), 128.2 (Ar-C), 128.2 (Ar-C), 124.6 (Ar-C), 114.9 (Ar-C), 79.9 (C(CH_3_)_3_**)**, 70.1 (CH_2_O), 54.5 (CH_2_CHNH), 52.2 (CH_3_OCO), 37.5 (CH_2_CHNH), 28.3 (C(CH_3_)_3_), 21.4 (CH_3_Ph). HRMS calculated for C_23_H_29_NNaO_5_ [M + Na]^+^ 422.1938, found 422.1935.

##### (*S*)-Methyl 2-amino-3-(4-((3-methylbenzyl)oxy)phenyl)propanoate (**6b**)

Olive green solid, 1.47 g (98%). ^1^H NMR (500 MHz, CDCl_3_): *δ* 7.91 (2H, bs, NH_2_), 7.24 (1H, t, *J =* 7.6 Hz, Ar-H), 7.20 (1H, s, Ar-H), 7.17 (1H, d, *J =* 7.6 Hz, Ar-H), 7.11 (1H, d, *J =* 7.4 Hz, Ar-H), 7.07 (2H, d, *J =* 8.5 Hz, Ar-H), 6.91 (2H, d, *J =* 8.5 Hz, Ar-H), 4.94 (2H, s, CH_2_O), 4.23 (1H, dd, *J =* 7.7, 5.2 Hz, CH_2_CHNH), 3.73 (3H, s, CH_3_O), 3.23 (1H, dd, *J* = 13.8, 5.2 Hz, CH_A_H_B_CHNH), 3.13 (1H, dd, *J* = 13.8, 7.7 Hz, CH_A_H_B_CHNH), 2.34 (3H, s, CH_3_Ph). ^13 ^C NMR (125 MHz, CDCl_3_): *δ* 169.2 (CO), 158.8 (Ar-C), 138.3 (Ar-C), 136.5 (Ar-C), 130.3 (Ar-C), 128.9 (Ar-C), 128.5 (Ar-C), 128.3 (Ar-C), 124.6 (Ar-C), 124.6 (Ar-C), 115.6 (Ar-C), 70.1 (CH_2_O), 54.5 (CH_2_CHNH), 53.4 (CH_3_O), 35.2 (CH_2_CHNH), 21.4 (CH_3_Ph). HRMS calculated for C_18_H_22_NO_3_ [M + H]^+^ 300.1594, found 300.1592.

##### (*S*)-Methyl 2-(2-(acetylthio)acetamido)-3-(4-((3-methylbenzyl)oxy)phenyl)propanoate (**8b**)

Viscous yellow oil, 1.33 g (80%). ^1^H NMR (500 MHz, CDCl_3_): *δ* 7.29–7.16 (3H, m, Ar-H), 7.12 (1H, d, *J =* 7.4 Hz, Ar-H), 6.98 (2H, d, *J =* 8.5 Hz, Ar-H), 6.86 (2H, d, *J =* 8.5 Hz, Ar-H), 6.62 (1H, bs, NH), 4.97 (2H, s, CH_2_O), 4.75 (1H, q, *J =* 6.5 Hz, CH_2_CHNH), 3.71 (3H, s, CH_3_O), 3.52 (2H, dd, *J* = 32.0, 15.1 Hz, CH_2_S), 3.07 (1H, dd, *J* = 13.8, 5.5 Hz, CH_A_H_B_CHNH), 2.99 (1H, dd, *J* = 13.8, 6.1 Hz, CH_A_H_B_CHNH), 2.35 (3H, s, CH_3_CO), 2.31 (3H, s, CH_3_Ph). ^13 ^C NMR (125 MHz, CDCl_3_): *δ* 195.2 (COS), 171.6 (CO_2_), 167.7 (CONH), 158.0 (Ar-C), 138.3 (Ar-C), 136.8 (Ar-C), 130.3 (Ar-C), 128.7 (Ar-C), 128.5 (Ar-C), 128.2 (Ar-C), 127.7 (Ar-C), 124.6 (Ar-C), 114.9 (Ar-C), 70.0 (CH_2_O), 53.5 (CH_2_CHNH), 52.4 (CH_3_O), 36.7 (CH_2_CHNH), 32.7 (CH_2_S), 30.1 (CH_3_CO), 21.4 (CH_3_Ph). HRMS calculated for C_22_H_25_NNaO_5_S [M + Na]^+^ 438.1346, found 438.1343.

##### (*S*)-2-(2-Mercaptoacetamido)-3-(4-((3-methylbenzyl)oxy)phenyl)propanoic acid (2b)

Off white solid, 0.70 g (65%). ^1^H NMR (400 MHz, CD_3_OD): *δ* 7.25–7.16 (3H, m, Ar-H), 7.15–7.08 (3H, m, Ar-H), 6.89 (2H, d, *J =* 8.5 Hz, Ar-H), 4.99 (2H, s, ArCH_2_O), 4.61 (1H, dd, *J =* 8.3, 5.3 Hz, CH_2_CHNH), 3.14 (1H, dd, *J* = 14.0, 6.0, CH_A_H_B_CHNH), 3.12 (2H, s, CH_2_SH), 2.93 (1H, dd, *J* = 14.0, 8.2 Hz, CH_A_H_B_CHNH), 2.33 (3H, s, CH_3_). ^13 ^C NMR (100 MHz, CD_3_OD): *δ* 174.4 (CO_2_H), 172.9 (CONH), 159.3 (Ar-C), 139.3 (Ar-C), 138.7 (Ar-C), 131.4 (Ar-C), 130.3 (Ar-C), 129.5 (Ar-C), 129.4 (Ar-C), 129.1 (Ar-C), 125.6 (Ar-C), 115.9 (Ar-C), 71.0 (PhCH_2_O), 55.3 (CH_2_CHNH), 37.5 (CH_2_CHNH), 28.0 (CH_2_SH), 21.4 (CH_3_). HRMS calculated for C_19_H_20_NO_4_S [M-H]^−^ 358.1119, found 358.1129.

##### (*S*)-Methyl 2-((tert-butoxycarbonyl)amino)-3-(4-((4-methylbenzyl)oxy)phenyl)propanoate (**5c**)

Off white solid, 2.44 g (90%). ^1^H NMR (400 MHz, CDCl_3_): *δ* 7.29 (2H, d, *J =* 7.9 Hz, Ar-H), 7.17 (2H, d, *J =* 7.9 Hz, Ar-H), 7.01 (2H, d, *J =* 8.5 Hz, Ar-H), 6.87 (2H, d, *J =* 8.5 Hz, Ar-H), 4.97 (2H, s, CH_2_O), 4.94 (1H, bs, NH), 4.61–4.44 (1H, m, CH_2_CHNH), 3.68 (3H, s, CH_3_O), 3.02–2.92 (2H, m, CH_2_CHNH), 2.34 (3H, s, CH_3_Ph), 1.40 (9H, s, C(CH_3_)_3_). ^13 ^C NMR (100 MHz, CDCl_3_): *δ* 172.4 (CO_2_CH_3_), 158.0 (CO_2_Boc), 155.1 (Ar-C), 137.7 (Ar-C), 133.9 (Ar-C), 130.3 (Ar-C), 129.3 (Ar-C), 128.1 (Ar-C), 127.6 (Ar-C), 114.9 (Ar-C), 79.9 (C(CH_3_)_3_), 69.9 (CH_2_O), 54.5 (CH_2_CHNH), 52.2 (CH_3_OCO), 37.5 (CH_2_CHNH), 28.3 (C(CH_3_)_3_), 21.2 (CH_3_Ph). HRMS calculated for C_23_H_29_NNaO_5_ [M + Na]^+^ 422.1938, found 422.1936.

##### (*S*)-Methyl 2-amino-3-(4-((4-methylbenzyl)oxy)phenyl)propanoate (6c)

Pale yellow viscous oil, 1.47 g (98%). ^1^H NMR (400 MHz, CDCl_3_): *δ* 7.29 (2H, d, *J =* 7.9 Hz, Ar-H), 7.17 (2H, d, *J =* 7.9 Hz, Ar-H), 7.07 (2H, d, *J =* 8.5 Hz, Ar-H), 6.89 (2H, d, *J =* 8.5 Hz, Ar-H), 4.97 (2H, s, CH_2_O), 3.74 (1H, dd, *J* = 7.3, 5.3 Hz, CH_2_CHNH), 3.70 (3H, s, CH_3_O), 3.03 (1H, dd, *J* = 13.8, 5.2 Hz, CH_A_H_B_CHNH), 2.84 (1H, dd, *J* = 13.8, 7.7 Hz, CH_A_H_B_CHNH), 2.68 (2H, bs, NH_2_), 2.34 (3H, s, CH_3_Ph). ^13 ^C NMR (100 MHz, CDCl_3_): *δ* 174.5 (CO), 158.0 (Ar-C), 137.7 (Ar-C), 133.9 (Ar-C), 130.3 (Ar-C), 129.3 (Ar-C), 128.6 (Ar-C), 127.6 (Ar-C), 115.0 (Ar-C), 69.9 (CH_2_O), 55.6 (CH_2_CHNH), 52.2 (CH_3_O), 39.4 (CH_2_CHNH), 21.2 (CH_3_Ph). HRMS calculated for C_18_H_22_NO_3_ [M + H]^+^ 300.1594, found 300.1591.

##### (*S*)-Methyl 2-(2-(acetylthio)acetamido)-3-(4-((4-methylbenzyl)oxy)phenyl)propanoate (**8c**)

Off white solid, 1.36 g (82%). ^1^H NMR (500 MHz, CDCl_3_): *δ* 7.28 (2H, d, *J =* 7.9 Hz, Ar-H), 7.17 (2H, d, *J =* 7.9 Hz, Ar-H), 6.97 (2H, d, *J =* 8.6 Hz, Ar-H), 6.86 (2H, d, *J =* 8.6 Hz, Ar-H), 6.57 (1H, d, *J =* 7.7 Hz, NH), 4.97 (2H, s, CH_2_O), 4.75 (1H, dt, *J* = 7.8, 5.5 Hz, CH_2_CHNH), 3.70 (3H, s, CH_3_O), 3.51 (2H, dd, *J =* 31.0, 14.8 Hz, CH_2_S), 3.06 (1H, dd, *J* = 13.8, 5.5 Hz, CH_A_H_B_CHNH), 2.99 (1H, dd, *J* = 13.8, 6.1 Hz, CH_A_H_B_CHNH), 2.34 (3H, s, CH_3_CO), 2.31 (3H, s, CH_3_Ph). ^13 ^C NMR (125 MHz, CDCl_3_): *δ* 195.2 (COS), 171.6 (CO_2_), 167.6 (CONH), 158.0 (Ar-C), 137.8 (Ar-C), 133.9 (Ar-C), 130.3 (Ar-C), 129.3 (Ar-C), 127.7 (Ar-C), 127.6 (Ar-C), 114.9 (Ar-C), 69.9 (CH_2_O), 53.5 (CH_2_CHNH), 52.3 (CH_3_O), 36.8 (CH_2_CHNH), 32.8 (CH_2_S), 30.1 (CH_3_CO), 21.2 (CH_3_Ph). HRMS calculated for C_22_H_25_NNaO_5_S [M + Na]^+^ 438.1346, found 438.1342.

##### (*S*)-2-(2-Mercaptoacetamido)-3-(4-((4-methylbenzyl)oxy)phenyl)propanoic acid (2c)

Off white solid, 0.73 g (68%). ^1^H NMR (500 MHz, CD_3_OD): *δ* 7.26 (2H, d, *J =* 9.0 Hz, Ar-H), 7.19–7.06 (4H, m, Ar-H), 6.88 (2H, d, *J =* 8.5 Hz, Ar-H), 4.96 (2H, s, ArCH_2_O), 4.61 (1H, dd, *J* = 8.3, 5.3 Hz, CH_2_CHNH), 3.13 (1H, dd, *J* = 14.0, 6.0), 3.11 (2H, s, CH_2_SH), 2.93 (1H, dd, *J* = 14.0, 8.2 Hz, CH_A_H_B_CHNH), 2.31 (3H, s, CH_3_). ^13 ^C NMR (125 MHz, CD_3_OD): *δ* 174.4 (CO_2_H), 172.9 (CONH), 159.2 (Ar-C), 138.7 (Ar-C), 135.7 (Ar-C), 131.4 (Ar-C), 130.2 (Ar-C), 130.1 (Ar-C), 128.7 (Ar-C), 115.9 (Ar-C), 70.9 (PhCH_2_O), 55.3 (CH_2_CHNH), 37.5 (CH_2_CHNH), 28.0 (CH_2_SH), 21.2 (CH3), 1.90 (1H, s, SH). HRMS calculated for C_19_H_20_NO_4_S [M-H]^-^ 358.1191, found 358.1192.

##### (*S*)-Methyl 2-((tert-butoxycarbonyl)amino)-3-(4-((2-chlorobenzyl)oxy)phenyl)propanoate (**5d**)

Off white solid, 2.34 g (82%). ^1^H NMR (500 MHz, CDCl_3_): *δ* 7.53 (1H, dd, *J* = 7.1, 1.4 Hz, Ar-H), 7.38 (1H, dd, *J* = 7.1, 1.4 Hz, Ar-H), 7.30–7.20 (2H, m, Ar-H), 7.03 (2H, d, *J =* 8.5 Hz, Ar-H), 6.88 (2H, d, *J =* 8.5 Hz, Ar-H), 5.12 (2H, s, CH_2_O), 4.96 (1H, d, *J =* 6.7 Hz, NH), 4.53 (1H, q, *J =* 7.0 Hz, CH_2_CHNH), 3.69 (3H, s, CH_3_O), 3.04 (1H, dd, *J* = 13.8, 5.8 Hz, CH_A_H_B_CHNH), 2.97 (1H, dd, *J* = 13.8, 6.0 Hz, CH_A_H_B_CHNH), 1.40 (9H, s, C(CH_3_)_3_). ^13 ^C NMR (125 MHz, CDCl_3_): *δ* 172.4 (CO_2_CH_3_), 157.6 (NHCO_2_), 155.1 (Ar-C), 134.7 (Ar-C), 132.5 (Ar-C), 130.4 (Ar-C), 129.3 (Ar-C), 128.9 (Ar-C), 128.7 (Ar-C), 128.5 (Ar-C), 126.9 (Ar-C), 114.9 (Ar-C), 79.9 (C(CH_3_)_3_), 67.1 (CH_2_O), 54.5 (CH_2_CHNH), 52.2 (CH_3_O), 37.5 (CH_2_CHNH), 28.3 (C(CH_3_)_3_). HRMS calculated for C_22_H_26_ClNNaO_5_ [M + Na]^+^ 442.1392, found 442.1397.

##### (*S*)-Methyl 2-amino-3-(4-((2-chlorobenzyl)oxy)phenyl)propanoate (**6d**)

Green viscous oil, 1.57 g (98%). ^1^H NMR (500 MHz, CDCl_3_): *δ* 7.53 (1H, dd, *J* = 7.1, 1.4 Hz, Ar-H), 7.37 (1H, dd, *J* = 7.1, 1.4 Hz, Ar-H), 7.28–7.20 (2H, m, Ar-H), 7.10 (2H, d, *J =* 8.5 Hz, Ar-H), 6.90 (2H, d, *J =* 8.5 Hz, Ar-H), 5.12 (2H, s, CH_2_O), 3.69 (4H, s: 1H for CH_2_CHNH and 3H for CH_3_O), 3.01 (1H, dd, *J* = 13.6, 5.1 Hz, CH_A_H_B_CHNH), 2.81 (1H, dd, *J* = 13.7, 7.7 Hz, CH_A_H_B_CHNH), 1.67 (2H, bs, NH_2_). ^13 ^C NMR (125 MHz, CDCl_3_): *δ* 175.3 (CO), 157.4 (Ar-C), 134.7 (Ar-C), 132.5 (Ar-C), 130.3 (Ar-C), 129.6 (Ar-C), 129.3 (Ar-C), 128.9 (Ar-C), 128.7 (Ar-C), 126.9 (Ar-C), 114.9 (Ar-C), 67.1 (CH_2_O), 55.8 (CH_2_CHNH), 51.9 (CH_3_O), 40.0 (CH_2_CHNH). HRMS calculated for C_17_H_19_ClNO_3_ [M + H]^+^ 320.1048, found 320.1049.

##### (*S*)-Methyl 2-(2-(acetylthio)acetamido)-3-(4-((2-chlorobenzyl)oxy)phenyl)propanoate (**8d**)

Yellow viscous oil, 1.43 g (82%). ^1^H NMR (500 MHz, CDCl_3_): *δ* 7.53 (1H, dd, *J* = 7.1, 1.4 Hz, Ar-H), 7.37 (1H, dd, *J* = 7.1, 1.4 Hz, Ar-H), 7.28–7.20 (2H, m, Ar-H), 7.00 (2H, d, *J =* 8.5 Hz, Ar-H), 6.88 (2H, d, *J =* 8.5 Hz, Ar-H), 6.60 (1H, d, *J =* 7.7 Hz, NH), 5.12 (2H, s, CH_2_O), 4.75 (1H, q, *J =* 6.4 Hz, CH_2_CHNH), 3.70 (3H, s, CH_3_O), 3.51 (2H, dd, *J =* 32.0, 15.0 Hz, CH_2_S), 3.07 (1H, dd, *J* = 13.8, 5.5 Hz, CH_A_H_B_CHNH), 2.99 (1H, dd, *J* = 13.8, 6.1 Hz, CH_A_H_B_CHNH), 2.32 (3H, s, CH_3_CO). ^13 ^C NMR (125 MHz, CDCl_3_): *δ* 195.1 (COS), 171.6 (CO_2_), 167.6 (CONH), 157.6 (Ar-C), 134.6 (Ar-C), 132.5 (Ar-C), 130.3 (Ar-C), 129.3 (Ar-C), 129.0 (Ar-C), 128.7 (Ar-C), 128.1 (Ar-C), 126.9 (Ar-C), 114.8 (Ar-C), 67.1 (CH_2_O), 53.5 (CH_2_CHNH), 52.3 (CH_3_O), 36.7 (CH_2_CHNH), 32.7 (CH_2_S), 30.1 (CH_3_CO). HRMS calculated for C_21_H_22_ClNNaO_5_S [M + Na]^+^ 458.0799, found 458.0803.

##### (*S*)-3-(4-((2-Chlorobenzyl)oxy)phenyl)-2-(2-mercaptoacetamido)propanoic acid (2d)

Tan solid, 0.79 g (69%). ^1^H NMR (500 MHz, CD_3_OD): *δ* 7.52 (1H, m, Ar-H), 7.40 (1H, m, Ar-H), 7.31–7.25 (2H, m, Ar-H), 7.14 (2H, d, *J =* 8.5 Hz, Ar-H), 6.90 (2H, d, *J =* 8.5 Hz, Ar-H), 5.10 (2H, s, CH_2_O), 4.62 (1H, dd, *J* = 8.0, 5.4 Hz, CH_2_CHNH), 3.14 (1H, dd, *J* = 13.8, 5.5 Hz, CH_A_H_B_CHNH), 3.12 (2H, s, CH_2_SH), 2.95 (1H, dd, *J* = 13.8, 6.1 Hz, CH_A_H_B_CHNH). ^13 ^C NMR (125 MHz, CD_3_OD): *δ* 174.3 (CO_2_H), 172.9 (CONH), 159.0 (Ar-C), 136.1 (Ar-C), 134.0 (Ar-C), 131.5 (Ar-C), 130.6 (Ar-C), 130.4 (Ar-C), 130.4 (Ar-C), 130.3 (Ar-C), 128.1 (Ar-C), 115.9 (Ar-C), 68.3 (PhCH_2_O), 55.3 (CH_2_CHNH), 37.5 (CH_2_CHNH), 28.0 (CH_2_SH). HRMS calculated for C_18_H_17_ClNO_4_S [M-H]^−^ 378.0572, found 378.0566.

##### (*S*)-Methyl 2-((tert-butoxycarbonyl)amino)-3-(4-((3-chlorobenzyl)oxy)phenyl)propanoate (**5e**)

Pale yellow oil, 2.28 g (80%). ^1^H NMR (500 MHz, CDCl_3_): *δ* 7.41 (1H, s, Ar-H), 7.28 (3H, s, Ar-H), 7.02 (2H, d, *J =* 8.5 Hz, Ar-H), 6.86 (2H, d, *J =* 8.5 Hz, Ar-H), 4.99 (2H, s, CH_2_O), 4.96 (1H, d, *J =* 7.5 Hz, NH), 4.53 (1H, q, *J =* 7.2 Hz, CH_2_CHNH), 3.69 (3H, s, CH_3_O), 3.03 (1H, dd, *J* = 13.8, 5.8 Hz, CH_A_H_B_CHNH), 2.98 (1H, dd, *J* = 13.8, 6.0 Hz, CH_A_H_B_CHNH), 1.40 (9H, s, C(CH_3_)_3_). ^13 ^C NMR (125 MHz, CDCl_3_): *δ* 172.4 (CO_2_CH_3_), 157.5 (NHCO_2_), 155.1 (Ar-C), 139.1 (Ar-C), 134.5 (Ar-C), 130.3 (Ar-C), 129.8 (Ar-C), 128.6 (Ar-C), 128.0 (Ar-C), 127.4 (Ar-C), 125.3 (Ar-C), 114.9 (Ar-C), 79.9 (C(CH_3_)_3_), 69.1 (CH_2_O), 54.5 (CH_2_CHNH), 52.2 (CH_3_O), 37.5 (CH_2_CHNH), 28.3 (C(CH_3_)_3_). HRMS calculated for C_22_H_26_ClNNaO_5_ [M + Na]^+^ 442.1392, found 442.1394.

##### (*S*)-Methyl 2-amino-3-(4-((3-chlorobenzyl)oxy)phenyl)propanoate (**6e**)

Yellow solid, 1.57 g (98%). ^1^H NMR (500 MHz, CDCl_3_): *δ* 7.40 (1H, s, Ar-H), 7.26 (3H, s, Ar-H), 7.08 (2H, d, *J =* 8.6 Hz, Ar-H), 6.87 (2H, d, *J =* 8.6 Hz, Ar-H), 4.97 (2H, s, CH_2_O), 3.68 (3H, s, CH_3_O), 3.66 (1H, m, CH_2_CHNH), 3.00 (1H, dd, *J* = 13.6, 5.1 Hz, CH_A_H_B_CHNH), 2.80 (1H, dd, *J* = 13.6, 7.7 Hz, CH_A_H_B_CHNH), 1.65 (2H, bs, NH_2_). ^13 ^C NMR (125 MHz, CDCl_3_): *δ* 175.3 (CO), 157.3 (Ar-C), 139.0 (Ar-C), 134.4 (Ar-C), 130.2 (Ar-C), 129.7 (Ar-C), 129.6 (Ar-C), 127.9 (Ar-C), 127.3 (Ar-C), 125.2 (Ar-C), 114.8 (Ar-C), 69.0 (CH_2_O), 55.7 (CH_2_CHNH), 51.8 (CH_3_O), 40.0 (CH_2_CHNH). HRMS calculated for C_17_H_19_ClNO_3_ [M + H]^+^ 320.1048, found 320.1047.

##### (*S*)-Methyl 2-(2-(acetylthio)acetamido)-3-(4-((3-chlorobenzyl)oxy)phenyl)propanoate (**8e**)

Brown viscous oil, 1.45 g (83%). ^1^H NMR (500 MHz, CDCl_3_): *δ* 7.40 (1H, s, Ar-H), 7.29 (3H, s, Ar-H), 6.99 (2H, d, *J =* 8.6 Hz, Ar-H), 6.85 (2H, d, *J =* 8.6 Hz, Ar-H), 6.59 (1H, d, *J =* 7.7 Hz, NH), 4.99 (2H, s, CH_2_O), 4.76 (1H, dt, *J =* 7.7, 5.9 Hz, CH_2_CHNH), 3.70 (3H, s, CH_3_O), 3.51 (2H, dd, *J =* 27.5, 14.8 Hz, CH_2_S), 3.07 (1H, dd, *J* = 13.8, 5.5 Hz, CH_A_H_B_CHNH), 2.99 (1H, dd, *J* = 13.8, 6.1 Hz, CH_A_H_B_CHNH), 2.32 (3H, s, CH_3_CO). ^13 ^C NMR (125 MHz, CDCl_3_): *δ* 195.2 (COS), 171.5 (CO_2_), 167.6 (CONH), 157.6 (Ar-C), 139.0 (Ar-C), 134.5 (Ar-C), 130.4 (Ar-C), 129.9 (Ar-C), 128.2 (Ar-C), 128.1 (Ar-C), 127.4 (Ar-C), 125.3 (Ar-C), 114.8 (Ar-C), 69.1 (CH_2_O), 53.5 (CH_2_CHNH), 52.4 (CH_3_O), 36.8 (CH_2_CHNH), 32.8 (CH_2_S), 30.1 (CH_3_CO). HRMS calculated for C_21_H_22_ClNNaO_5_S [M + Na]^+^ 458.0799, found 458.0800.

##### (*S*)-3-(4-((3-chlorobenzyl)oxy)phenyl)-2–(2-mercaptoacetamido)propanoic acid (**2e**)

Tan solid, 0.77 g (68%). ^1^H NMR (500 MHz, CD_3_OD): *δ* 7.42 (1H, s, Ar-H), 7.33–7.26 (3H, m, Ar-H), 7.13 (2H, d, *J =* 8.6 Hz, Ar-H), 6.89 (2H, d, *J =* 8.6 Hz, Ar-H), 5.02 (2H, s, ArCH_2_O), 4.62 (1H, dd, *J* = 8.2, 5.2 Hz, CH_2_CHNH), 3.14 (1H, dd, *J* = 18.0, 6.0 Hz, CH_A_H_B_CHNH), 3.12 (2H, s, CH_2_SH), 2.94 (1H, dd, *J* = 18.0, 8.0 Hz, CH_A_H_B_CHNH). ^13 ^C NMR (125 MHz, CD_3_OD): *δ* 174.3 (CO_2_H), 172.9 (CONH), 158.9 (Ar-C), 141.2 (Ar-C), 135.4 (Ar-C), 131.5 (Ar-C), 131.0 (Ar-C), 130.6 (Ar-C), 128.8 (Ar-C), 128.3 (Ar-C), 126.6 (Ar-C), 115.9 (Ar-C), 70.0 (PhCH_2_O), 55.3 (CH_2_CHNH), 37.5 (CH_2_CHNH), 28.0 (CH_2_SH). HRMS calculated for C_18_H_17_ClNO_4_S [M-H]^−^ 378.0572, found 378.0569.

##### (*S*)-Methyl 2-((tert-butoxycarbonyl)amino)-3-(4-((4-chlorobenzyl)oxy)phenyl)propanoate (**5f**)

Yellow solid, 2.28 g (82%). ^1^H NMR (400 MHz, CDCl_3_): *δ* 7.33 (4H, s, Ar-H), 7.02 (2H, d, *J =* 8.5 Hz, Ar-H), 6.85 (2H, d, *J =* 8.5 Hz, Ar-H), 4.98 (2H, s, CH_2_O), 4.94 (1H, d, *J =* 7.5 Hz, NH), 4.59–4.46 (1H, m, CH_2_CHNH), 3.69 (3H, s, CH_3_O), 3.05 (1H, dd, *J* = 13.8, 5.8 Hz, CH_A_H_B_CHNH), 2.95 (1H, dd, *J* = 13.8, 6.0 Hz, CH_A_H_B_CHNH), 1.40 (9H, s, C(CH_3_)_3_). ^13 ^C NMR (100 MHz, CDCl_3_): *δ* 172.4 (CO_2_CH_3_), 157.6 (NHCO_2_), 155.1 (Ar-C), 135.5 (Ar-C), 133.7 (Ar-C), 130.3 (Ar-C), 128.7 (Ar-C), 128.5 (Ar-C), 128.2 (Ar-C), 114.9 (Ar-C), 79.9 (C(CH_3_)_3_), 69.2 (CH_2_O), 54.5 (CH_2_CHNH), 52.2 (CH_3_O), 37.5 (CH_2_CHNH), 28.3 (C(CH_3_)_3_). HRMS calculated for C_22_H_26_ClNNaO_5_ [M + Na]^+^ 442.1392, found 442.1395.

##### (*S*)-Methyl 2-amino-3-(4-((4-chlorobenzyl)oxy)phenyl)propanoate (**6f**)

Brown solid, 1.57 g (98%). ^1^H NMR (500 MHz, CDCl_3_): *δ* 7.33 (4H, s, Ar-H), 7.08 (2H, d, *J =* 8.6 Hz, Ar-H), 6.87 (2H, d, *J =* 8.6 Hz, Ar-H), 4.98 (2H, s, CH_2_O), 3.69 (4H, s, (1H for CH_2_CHNH and 3H for CH_3_O)), 3.01 (1H, dd, *J* = 13.8, 5.8 Hz, CH_A_H_B_CHNH), 2.80 (1H, dd, *J* = 13.8, 6.0 Hz, CH_A_H_B_CHNH), 1.71 (2H, s, NH_2_). ^13 ^C NMR (125 MHz, CDCl_3_): *δ* 175.3 (CO), 157.5 (Ar-C), 135.5 (Ar-C), 133.7 (Ar-C), 130.3 (Ar-C), 129.6 (Ar-C), 128.9 (Ar-C), 128.7 (Ar-C), 114.9 (Ar-C), 69.2 (CH_2_O), 55.8 (CH_2_CHNH), 52.0 (CH_3_O), 40.0 (CH_2_CHNH). HRMS calculated for C_17_H_19_ClNO_3_ [M + H]^+^ 320.1048, found 320.1046.

##### (*S*)-Methyl 2-(2-(acetylthio)acetamido)-3-(4-((4-chlorobenzyl)oxy)phenyl)propanoate (**8f**)

Brown viscous oil, 1.39 g (80%). ^1^H NMR (500 MHz, CDCl_3_): *δ* 7.33 (4H, s, Ar-H), 6.98 (2H, d, *J =* 8.7 Hz, Ar-H), 6.84 (2H, d, *J =* 8.7 Hz, Ar-H), 6.59 (1H, d, *J =* 7.7 Hz, NH), 4.98 (2H, s, CH_2_O), 4.75 (1H, dt, *J* = 7.8, 5.5 Hz, CH_2_CHNH), 3.70 (3H, s, CH_3_O), 3.51 (2H, dd, *J =* 27.5, 14.2 Hz, CH_2_S), 3.07 (1H, dd, *J* = 13.8, 5.5 Hz, CH_A_H_B_CHNH), 2.98 (1H, dd, *J* = 13.8, 6.1 Hz, CH_A_H_B_CHNH), 2.32 (3H, s, CH_3_CO). ^13 ^C NMR (125 MHz, CDCl_3_): *δ* 195.2 (COS), 171.6 (CO_2_), 167.7 (CONH), 157.7 (Ar-C), 135.4 (Ar-C), 133.8 (Ar-C), 130.4 (Ar-C), 130.3 (Ar-C), 128.7 (Ar-C), 128.1 (Ar-C), 114.9 (Ar-C), 69.2 (CH_2_O), 53.5 (CH_2_CHNH), 52.4 (CH_3_O), 36.8 (CH_2_CHNH), 32.7 (CH_2_S), 30.1 (CH_3_CO). HRMS calculated for C_21_H_22_ClNNaO_5_S [M + Na]^+^ 458.0799, found 458.0806.

##### (*S*)-3-(4-((4-Chlorobenzyl)oxy)phenyl)-2-(2-mercaptoacetamido)propanoic acid (**2f**)

Off white soild. 0.80 g (70%). ^1^H NMR (500 MHz, CD_3_OD): *δ* 7.39 (2H, d, *J =* 8.5 Hz, Ar-H), 7.34 (2H, d, *J =* 8.5 Hz, Ar-H), 7.13 (2H, d, *J =* 8.5 Hz, Ar-H), 6.89 (2H, d, *J =* 8.5 Hz, Ar-H), 5.02 (2H, s, ArCH_2_O), 4.61 (1H, dd, *J* = 8.3, 5.3 Hz, CH_2_CHNH), 3.14 (1H, *J* = 14.0, 5.5 Hz, CH_A_H_B_CHNH), 3.13 (2H, s, CH_2_SH), 2.94 (1H, dd, *J* = 14.0, 8.2 Hz, CH_A_H_B_CHNH). ^13 ^C NMR (125 MHz, CD_3_OD): *δ* 174.3 (CO_2_H), 172.9 (CONH), 159.0 (Ar-C), 137.6 (Ar-C), 134.6 (Ar-C), 131.5 (Ar-C), 130.5 (Ar-C), 130.1 (Ar-C), 129.6 (Ar-C), 115.9 (Ar-C), 70.1 (PhCH_2_O), 55.3 (CH_2_CHNH), 37.5 (CH_2_CHNH), 28.00 (CH_2_SH). HRMS calculated for C_18_H_17_ClNO_4_S [M-H]^−^ 378.0572, found 378.0557.

##### (*S*)-Methyl 2-((tert-butoxycarbonyl)amino)-3-(4-((2-nitrobenzyl)oxy)phenyl)propanoate (**5g**)

Pale yellow viscous oil, 2.78 g (95%). ^1^H NMR (500 MHz, CDCl_3_): *δ* 8.13 (1H, d, *J =* 8.0 Hz, Ar-H), 7.85 (1H, d, *J =* 7.8 Hz, Ar-H), 7.65 (1H, t, *J =* 7.6 Hz, Ar-H), 7.44 (1H, t, *J =* 8.0 Hz, Ar-H), 7.03 (2H, d, *J =* 8.5 Hz, Ar-H), 6.88 (2H, d, *J =* 8.5 Hz, Ar-H), 5.44 (2H, s, CH_2_O), 4.97 (1H, d, *J =* 6.7 Hz, NH), 4.52 (1H, q, *J =* 7.0 Hz, CH_2_CHNH), 3.69 (3H, s, CH_3_O), 3.04 (1H, dd, *J* = 13.8, 5.8 Hz, CH_A_H_B_CHNH), 2.97 (1H, dd, *J* = 13.8, 6.0 Hz, CH_A_H_B_CHNH), 1.39 (9H, s, C(CH_3_)_3_). ^13 ^C NMR (125 MHz, CDCl_3_): *δ* 172.3 (CO_2_CH_3_), 157.2 (NHCO_2_), 155.0 (Ar-C), 146.8 (Ar-C), 134.0 (Ar-C), 133.9 (Ar-C), 130.4 (Ar-C), 128.9 (Ar-C), 128.5 (Ar-C), 128.3 (Ar-C), 124.9 (Ar-C), 114.9 (Ar-C), 79.9 (C(CH_3_)_3_), 66.8 (CH_2_O), 54.5 (CH_2_CHNH), 52.2 (CH_3_O), 37.4 (CH_2_CHNH), 28.3 (C(CH_3_)_3_). HRMS calculated for C_22_H_26_N_2_NaO_7_ [M + Na]^+^ 453.1632, found 453.1635.

##### (*S*)-Methyl 2-amino-3-(4-((2-nitrobenzyl)oxy)phenyl)propanoate (**6g**)

Tan yellow viscous oil, 1.62 g (98%). ^1^H NMR (500 MHz, CDCl_3_): *δ* 8.13 (1H, d, *J =* 8.0 Hz, Ar-H), 7.86 (1H, d, *J =* 7.8 Hz, Ar-H), 7.65 (1H, t, *J =* 7.6 Hz, Ar-H), 7.46 (1H, t, *J =* 8.0 Hz, Ar-H), 7.10 (2H, d, *J =* 8.5 Hz, Ar-H), 6.90 (2H, d, *J =* 8.5 Hz, Ar-H), 5.44 (2H, s, CH_2_O), 3.69 (4H, s: 1H for CH_2_CHNH and 3H for CH_3_O), 3.01 (1H, dd, *J* = 13.6, 5.1 Hz, CH_A_H_B_CHNH), 2.82 (1H, dd, *J* = 13.7, 7.7 Hz, CH_A_H_B_CHNH), 1.95 (2H, bs, NH_2_). ^13 ^C NMR (125 MHz, CDCl_3_): *δ* 175.2 (CO), 157.1 (Ar-C), 146.9 (Ar-C), 134.0 (Ar-C), 133.9 (Ar-C), 130.4 (Ar-C), 129.9 (Ar-C), 128.5 (Ar-C), 128.3 (Ar-C), 124.9 (Ar-C), 115.0 (Ar-C), 66.8 (CH_2_O), 55.7 (CH_2_CHNH), 52.0 (CH_3_O), 39.9 (CH_2_CHNH). HRMS calculated for C_17_H_19_N_2_O_5_ [M + H]^+^ 331.1288, found 331.1290.

##### (*S*)-Methyl 2-(2-(acetylthio)acetamido)-3-(4-((2-nitrobenzyl)oxy)phenyl)propanoate (**8g**)

Pale yellow solid, 1.48 g (83%). ^1^H NMR (500 MHz, CDCl_3_): *δ* 8.13 (1H, d, *J =* 8.0 Hz, Ar-H), 7.86 (1H, d, *J =* 7.8 Hz, Ar-H), 7.66 (1H, t, *J =* 7.6 Hz, Ar-H), 7.47 (1H, t, *J =* 8.0 Hz, Ar-H), 7.00 (2H, d, *J =* 8.5 Hz, Ar-H), 6.88 (2H, d, *J =* 8.5 Hz, Ar-H), 6.59 (1H, d, *J =* 7.6 Hz, NH), 5.43 (2H, s, CH_2_O), 4.76 (1H, dt, *J =* 7.8, 5.8 Hz, CH_2_CHNH), 3.70 (3H, s, CH_3_O), 3.51 (2H, dd, *J =* 28.2, 15.0 Hz, CH_2_S), 3.08 (1H, dd, *J* = 13.8, 5.5 Hz, CH_A_H_B_CHNH), 2.99 (1H, dd, *J* = 13.8, 6.1 Hz, CH_A_H_B_CHNH), 2.33 (3H, s, CH_3_CO). ^13 ^C NMR (125 MHz, CDCl_3_): *δ* 195.2 (COS), 171.5 (CO_2_), 167.6 (CONH), 157.3 (Ar-C), 146.9 (Ar-C), 134.0 (Ar-C), 133.8 (Ar-C), 130.5 (Ar-C), 128.5 (Ar-C), 128.5 (Ar-C), 128.3 (Ar-C), 125.0 (Ar-C), 114.9 (Ar-C), 66.8 (CH_2_O), 53.5 (CH_2_CHNH), 52.4 (CH_3_O), 36.8 (CH_2_CHNH), 32.8 (CH_2_S), 30.2 (CH_3_CO). HRMS calculated for C_21_H_22_N_2_NaO_7_S [M + Na]^+^ 469.1040, found 469.1045.

##### (*S*)-2-(2-Mercaptoacetamido)-3-(4-((2-nitrobenzyl)oxy)phenyl)propanoic acid (**2g**)

Pale yellow solid, 0.79 g (67%). ^1^H NMR (500 MHz, CDCl_3_): *δ* 8.22 (1H, bs, OH), 8.12 (1H, d, *J =* 8.0 Hz, Ar-H), 7.84 (1H, d, *J =* 7.8 Hz, Ar-H), 7.64 (1H, t, *J =* 7.6 Hz, Ar-H), 7.45 (1H, t, *J =* 8.0 Hz, Ar-H), 7.13 (1H, d, *J =* 7.6 Hz, NH), 7.00 (2H, d, *J =* 8.5 Hz, Ar-H), 6.89 (2H, d, *J =* 8.5 Hz, Ar-H), 5.41 (2H, s, CH_2_O), 4.80 (1H, q, *J =* 6.4 Hz, CH_2_CHNH), 3.20 (2H, d, *J =* 9.1 Hz, CH_2_SH), 3.16 (1H, dd, *J* = 13.8, 5.5 Hz, CH_A_H_B_CHNH), 3.07 (1H, dd, *J* = 13.8, 6.1 Hz, CH_A_H_B_CHNH), 1.83 (3H, t, *J =* 9.1 Hz, SH). ^13 ^C NMR (125 MHz, CDCl_3_): *δ* 177.0 (CO_2_H), 170.0 (CONH), 157.4 (Ar-C), 146.9 (Ar-C), 134.0 (Ar-C), 133.7 (Ar-C), 130.6 (Ar-C), 128.5 (Ar-C), 128.3 (Ar-C), 128.3 (Ar-C), 124.9 (Ar-C), 115.1 (Ar-C), 66.8 (PhCH_2_O), 53.5 (CH_2_CHNH), 36.4 (CH_2_CHNH), 28.1 (CH_2_SH). HRMS calculated for C_18_H_17_N_2_O_6_S [M-H]^−^ 389.0813, found 389.0809.

##### (*S*)-Methyl 2-((tert-butoxycarbonyl)amino)-3-(4-((3-nitrobenzyl)oxy)phenyl)propanoate (**5h**)

Yellow viscous oil, 2.63 g (90%). ^1^H NMR (500 MHz, CDCl_3_): *δ* 8.27 (1H, s, Ar-H), 8.15 (1H, d, *J =* 8.2 Hz, Ar-H), 7.72 (1H, d, *J =* 7.6 Hz, Ar-H), 7.52 (1H, t, *J =* 7.9 Hz, Ar-H), 7.03 (2H, d, *J =* 8.5 Hz, Ar-H), 6.86 (2H, d, *J =* 8.5 Hz, Ar-H), 5.09 (2H, s, CH_2_O), 4.98 (1H, d, *J =* 7.9 Hz, NH), 4.51 (1H, q, *J =* 7.0 Hz, CH_2_CHNH), 3.67 (3H, s, CH_3_O), 3.03 (1H, dd, *J* = 13.8, 5.8 Hz, CH_A_H_B_CHNH), 2.96 (1H, dd, *J* = 13.8, 6.0 Hz, CH_A_H_B_CHNH), 1.38 (9H, s, C(CH_3_)_3_). ^13 ^C NMR (125 MHz, CDCl_3_): *δ* 172.3 (CO_2_CH_3_), 157.2 (NHCO_2_), 155.0 (Ar-C), 148.3 (Ar-C), 139.2 (Ar-C), 133.0 (Ar-C), 130.4 (Ar-C), 129.5 (Ar-C), 128.9 (Ar-C), 122.8 (Ar-C), 122.0 (Ar-C), 114.8 (Ar-C), 79.8 (C(CH_3_)_3_), 68.5 (CH_2_O), 54.4 (CH_2_CHNH), 52.1 (CH_3_O), 37.4 (CH_2_CHNH), 28.2 (C(CH_3_)_3_). HRMS calculated for C_22_H_26_N_2_NaO_7_ [M + Na]^+^ 453.1632, found 453.1639.

##### (*S*)-Methyl 2-amino-3-(4-((3-nitrobenzyl)oxy)phenyl)propanoate (**6h**)

Brown solid, 1.62 g (98%). ^1^H NMR (400 MHz, CDCl_3_): *δ* 8.26 (1H, s, Ar-H), 8.12 (1H, d, *J =* 8.2 Hz, Ar-H), 7.72 (1H, d, *J =* 7.6 Hz, Ar-H), 7.52 (1H, t, *J =* 7.9 Hz, Ar-H), 7.09 (2H, d, *J =* 8.5 Hz, Ar-H), 6.87 (2H, d, *J =* 8.5 Hz, Ar-H), 5.08 (2H, s, CH_2_O), 3.72 (1H, dd, *J* = 7.4, 5.1 Hz, CH_2_CHNH), 3.67 (3H, s, CH_3_O), 3.02 (1H, dd, *J* = 13.6, 5.1 Hz, CH_A_H_B_CHNH), 2.84 (1H, dd, *J* = 13.7, 7.7 Hz, CH_A_H_B_CHNH), 2.75 (2H, bs, NH_2_). ^13 ^C NMR (100 MHz, CDCl_3_): *δ* 174.5 (CO), 157.1 (Ar-C), 148.3 (Ar-C), 139.1 (Ar-C), 133.0 (Ar-C), 130.4 (Ar-C), 129.5 (Ar-C), 122.8 (Ar-C), 122.0 (Ar-C), 121.2 (Ar-C), 114.8 (Ar-C), 68.5 (CH_2_O), 55.4 (CH_2_CHNH), 52.0 (CH_3_O), 39.3 (CH_2_CHNH). HRMS calculated for C_17_H_19_N_2_O_5_ [M + H]^+^ 331.1288, found 331.1286.

##### (*S*)-Methyl 2-(2-(acetylthio)acetamido)-3-(4-((3-nitrobenzyl)oxy)phenyl)propanoate (**8h**)

Yellow solid, 1.45 g (81%). ^1^H NMR (400 MHz, CDCl_3_): *δ* 8.28 (1H, s, Ar-H), 8.15 (1H, d, *J =* 8.2 Hz, Ar-H), 7.73 (1H, d, *J =* 7.6 Hz, Ar-H), 7.54 (1H, t, *J =* 7.9 Hz, Ar-H), 7.00 (2H, d, *J =* 8.5 Hz, Ar-H), 6.88 (2H, d, *J =* 8.5 Hz, Ar-H), 6.62 (1H, d, *J =* 7.6 Hz, NH), 5.10 (2H, s, CH_2_O), 4.74 (1H, dt, *J =* 7.8, 5.8 Hz, CH_2_CHNH), 3.69 (3H, s, CH_3_O), 3.51 (2H, dd, *J =* 22.8, 14.9 Hz, CH_2_S), 3.07 (1H, dd, *J* = 13.8, 5.5 Hz, CH_A_H_B_CHNH), 2.98 (1H, dd, *J* = 13.8, 6.1 Hz, CH_A_H_B_CHNH), 2.33 (3H, s, CH_3_CO). ^13 ^C NMR (100 MHz, CDCl_3_): *δ* 195.2 (COS), 171.5 (CO_2_), 167.6 (CONH), 157.3 (Ar-C), 148.4 (Ar-C), 139.1 (Ar-C), 133.1 (Ar-C), 130.5 (Ar-C), 129.5 (Ar-C), 128.6 (Ar-C), 122.9 (Ar-C), 122.1 (Ar-C), 114.8 (Ar-C), 68.6 (CH_2_O), 53.5 (CH_2_CHNH), 52.4 (CH_3_O), 36.8 (CH_2_CHNH), 32.7 (CH_2_S), 30.1 (CH_3_CO). HRMS calculated for C_21_H_22_N_2_NaO_7_S [M + Na]^+^ 469.1040, found 469.1045.

##### (*S*)-2-(2-Mercaptoacetamido)-3-(4-((3-nitrobenzyl)oxy)phenyl)propanoic acid (**2h**)

Pale yellow solid, 0.82 g (70%). ^1^H NMR (400 MHz, CDCl_3_): *δ* 10.56 (1H, bs, OH), 8.25 (1H, s, Ar-H), 8.13 (1H, d, *J =* 8.2 Hz, Ar-H), 7.71 (1H, d, *J =* 7.6 Hz, Ar-H), 7.52 (1H, t, *J =* 7.9 Hz, Ar-H), 7.20 (1H, d, *J =* 7.8 Hz, NH), 7.09 (2H, d, *J =* 8.5 Hz, Ar-H), 6.88 (2H, d, *J =* 8.5 Hz, Ar-H), 5.07 (2H, s, CH_2_O), 4.80 (1H, dt, *J =* 7.8, 5.2 Hz, CH_2_CHNH), 3.20 (2H, d, *J =* 9.1 Hz, CH_2_SH), 3.16 (1H, dd, *J* = 13.8, 5.5 Hz, CH_A_H_B_CHNH), 3.07 (1H, dd, *J* = 13.8, 6.1 Hz, CH_A_H_B_CHNH), 1.84 (3H, t, *J =* 9.1 Hz, SH). ^13 ^C NMR (100 MHz, CDCl_3_): *δ* 174.8 (CO_2_H), 170.3 (CONH), 157.4 (Ar-C), 148.3 (Ar-C), 139.0 (Ar-C), 133.1 (Ar-C), 130.5 (Ar-C), 129.5 (Ar-C), 128.2 (Ar-C), 122.9 (Ar-C), 122.1 (Ar-C), 114.9 (Ar-C), 68.5 (PhCH_2_O), 53.5 (CH_2_CHNH), 36.3 (CH_2_CHNH), 28.0 (CH_2_SH). HRMS calculated for C_18_H_17_N_2_O_6_S [M-H]^−^ 389.0813, found 389.0805.

##### (*S*)-Methyl 2-((tert-butoxycarbonyl)amino)-3-(4-((4-nitrobenzyl)oxy)phenyl)propanoate (**5i**)

Brown viscous oil, 2.69 g (92%). ^1^H NMR (500 MHz, CDCl_3_): *δ* 8.21 (2H, d, *J =* 8.7 Hz, Ar-H), 7.56 (2H, d, *J =* 8.7 Hz, Ar-H), 7.03 (2H, d, *J =* 8.5 Hz, Ar-H), 6.86 (2H, d, *J =* 8.5 Hz, Ar-H), 5.12 (2H, s, CH_2_O), 4.96 (1H, d, *J =* 7.3 Hz, NH), 4.50 (1H, q, *J =* 7.6 Hz, CH_2_CHNH), 3.68 (3H, s, CH_3_O), 3.03 (1H, dd, *J* = 13.8, 5.8 Hz, CH_A_H_B_CHNH), 2.97 (1H, dd, *J* = 13.8, 6.0 Hz, CH_A_H_B_CHNH), 1.38 (9H, s, C(CH_3_)_3_). ^13 ^C NMR (125 MHz, CDCl_3_): *δ* 172.3 (CO_2_CH_3_), 157.2 (NHCO_2_), 155.0 (Ar-C), 147.5 (Ar-C), 144.5 (Ar-C), 130.4 (Ar-C), 129.0 (Ar-C), 127.5 (Ar-C), 123.8 (Ar-C), 114.8 (Ar-C), 79.9 (C(CH_3_)_3_), 68.6 (CH_2_O), 54.5 (CH_2_CHNH), 52.2 (CH_3_O), 37.4 (CH_2_CHNH), 28.2 (C(CH_3_)_3_). HRMS calculated for C_22_H_26_N_2_NaO_7_ [M + Na]^+^ 453.1632, found 453.1636.

##### (*S*)-Methyl 2-amino-3-(4-((4-nitrobenzyl)oxy)phenyl)propanoate (6i)

Dark brown solid, 1.62 g (98%). ^1^H NMR (400 MHz, CDCl_3_): *δ* 8.16 (2H, d, *J =* 8.7 Hz, Ar-H), 7.80 (2H, bs, NH_2_), 7.53 (2H, d, *J =* 8.7 Hz, Ar-H), 7.08 (2H, d, *J =* 8.5 Hz, Ar-H), 6.87 (2H, d, *J =* 8.5 Hz, Ar-H), 5.05 (2H, s, CH_2_O), 4.28 (1H, t, *J =* 6.4 Hz, CH_2_CHNH), 3.77 (3H, s, CH_3_O), 3.26 (1H, dd, *J* = 13.6, 5.1 Hz, CH_A_H_B_CHNH), 3.12 (1H, dd, *J* = 13.7, 7.7 Hz, CH_A_H_B_CHNH). ^13 ^C NMR (100 MHz, CDCl_3_): *δ* 169.5 (CO), 158.1 (Ar-C), 147.4 (Ar-C), 144.3 (Ar-C), 130.4 (Ar-C), 127.5 (Ar-C), 125.1 (Ar-C), 123.8 (Ar-C), 115.5 (Ar-C), 68.5 (CH_2_O), 54.7 (CH_2_CHNH), 53.6 (CH_3_O), 35.2 (CH_2_CHNH). HRMS calculated for C_17_H_19_N_2_O_5_ [M + H]^+^ 331.1288, found 331.1287.

##### (*S*)-Methyl 2-(2-(acetylthio)acetamido)-3-(4-((4-nitrobenzyl)oxy)phenyl)propanoate (**8i**)

Brown viscous oil, 1.48 g (83%). ^1^H NMR (400 MHz, CDCl_3_): *δ* 8.21 (2H, d, *J =* 8.7 Hz, Ar-H), 7.57 (2H, d, *J =* 8.7 Hz, Ar-H), 7.00 (2H, d, *J =* 8.5 Hz, Ar-H), 6.85 (2H, d, *J =* 8.5 Hz, Ar-H), 6.60 (1H, d, *J =* 7.7 Hz, NH), 5.11 (2H, s, CH_2_O), 4.75 (1H, dt, *J =* 7.8, 5.2 Hz, CH_2_CHNH), 3.69 (3H, s, CH_3_O), 3.51 (2H, dd, *J =* 19.9, 14.8 Hz, CH_2_S), 3.07 (1H, dd, *J* = 13.8, 5.5 Hz, CH_A_H_B_CHNH), 2.97 (1H, dd, *J* = 13.8, 6.1 Hz, CH_A_H_B_CHNH), 2.33 (3H, s, CH_3_CO). ^13 ^C NMR (100 MHz, CDCl_3_): *δ* 195.1 (COS), 171.5 (CO_2_), 167.5 (CONH), 157.2 (Ar-C), 147.5 (Ar-C), 144.4 (Ar-C), 130.4 (Ar-C), 128.6 (Ar-C), 127.5 (Ar-C), 123.8 (Ar-C), 114.8 (Ar-C), 68.6 (CH_2_O), 53.5 (CH_2_CHNH), 52.3 (CH_3_O), 36.8 (CH_2_CHNH), 32.7 (CH_2_S), 30.1 (CH_3_CO). HRMS calculated for C_21_H_22_N_2_NaO_7_S [M + Na]^+^ 469.1040, found 469.1042.

##### (*S*)-2-(2-Mercaptoacetamido)-3-(4-((4-nitrobenzyl)oxy)phenyl)propanoic acid (**2i**)

Brown solid, 0.84 g (72%). ^1^H NMR (400 MHz, CDCl_3_): *δ* 9.43 (1H, bs, OH), 8.21 (2H, d, *J =* 8.7 Hz, Ar-H), 7.57 (2H, d, *J =* 8.7 Hz, Ar-H), 7.09 (2H, d, *J =* 8.5 Hz, Ar-H), 6.88 (2H, d, *J =* 8.5 Hz, Ar-H), 5.12 (2H, s, ArCH_2_O), 4.81 (1H, dd, *J* = 7.6, 6.0 Hz, CH_2_CHNH), 3.21 (2H, d, *J =* 9.1 Hz, CH_2_SH), 3.17 (1H, dd, *J* = 14.0, 5.7 Hz, CH_A_H_B_CHNH), 3.08 (1H, dd, *J* = 14.0, 8.0 Hz, CH_A_H_B_CHNH), 1.81 (1H, t, *J =* 9.1 Hz, SH). ^13 ^C NMR (100 MHz, CDCl_3_): *δ* 175.4 (CO_2_H), 169.9 (CONH), 157.4 (Ar-C), 147.6 (Ar-C), 144.3 (Ar-C), 130.6 (Ar-C), 128.3 (Ar-C), 127.6 (Ar-C), 123.8 (Ar-C), 115.0 (Ar-C), 68.6 (PhCH_2_O), 53.5 (CH_2_CHNH), 36.4 (CH_2_CHNH), 28.1 (CH_2_SH). HRMS calculated for C_18_H_17_N_2_O_6_S [M-H]^−^ 389.0813, found 389.0809.

##### (*S*)-Methyl 3-(4-((3-bromobenzyl)oxy)phenyl)-2-((tert-butoxycarbonyl)amino)propanoate (**5j**)

Tan viscous oil, 2.84 g (90%). ^1^H NMR (500 MHz, CDCl_3_): *δ* 7.56 (1H, s, Ar-H), 7.43 (1H, d, *J =* 8.0 Hz, Ar-H), 7.32 (1H, d, *J =* 7.7 Hz, Ar-H), 7.22 (1H, t, *J =* 8.0 Hz, Ar-H), 7.02 (2H, d, *J =* 8.5 Hz, Ar-H), 6.86 (2H, d, *J =* 8.5 Hz, Ar-H), 4.98 (2H, s, CH_2_O), 4.94 (1H, d, *J =* 7.5 Hz, NH), 4.53 (1H, q, *J =* 7.2 Hz, CH_2_CHNH), 3.68 (3H, s, CH_3_O), 3.03 (1H, dd, *J* = 13.8, 5.8 Hz, CH_A_H_B_CHNH), 2.97 (1H, dd, *J* = 13.8, 6.0 Hz, CH_A_H_B_CHNH), 1.40 (9H, s, C(CH_3_)_3_). ^13 ^C NMR (125 MHz, CDCl_3_): *δ* 172.4 (CO_2_CH_3_), 157.5 (NHCO_2_), 155.1 (Ar-C), 139.3 (Ar-C), 131.0 (Ar-C), 130.4 (Ar-C), 130.3 (Ar-C), 130.1 (Ar-C), 128.6 (Ar-C), 125.8 (Ar-C), 122.6 (Ar-C), 114.9 (Ar-C), 79.9 (C(CH_3_)_3_), 69.1 (CH_2_O), 54.5 (CH_2_CHNH), 52.2 (CH_3_O), 37.5 (CH_2_CHNH), 28.3 (C(CH_3_)_3_). HRMS calculated for C_22_H_26_BrNNaO_5_ [M + Na]^+^ 486.0887, found 486.0893.

##### (*S*)-Methyl 2-amino-3-(4-((3-bromobenzyl)oxy)phenyl)propanoate (**6j**)

Brown solid, 1.78 g (98%). ^1^H NMR (500 MHz, CDCl_3_): *δ* 7.56 (1H, s, Ar-H), 7.42 (1H, d, *J =* 8.0 Hz, Ar-H), 7.32 (1H, d, *J =* 7.7 Hz, Ar-H), 7.22 (1H, t, *J =* 7.8 Hz, Ar-H),7.09 (2H, d, *J =* 8.6 Hz, Ar-H), 6.87 (2H, d, *J =* 8.6 Hz, Ar-H), 4.98 (2H, s, CH_2_O), 3.69 (4H: 3H-singlet for CH_3_O and 1H-multiplet for CH_2_CHNH), 3.01 (1H, dd, *J* = 13.6, 5.1 Hz, CH_A_H_B_CHNH), 2.82 (1H, dd, *J* = 13.7, 7.7 Hz, CH_A_H_B_CHNH), 1.90 (2H, bs, NH_2_). ^13 ^C NMR (125 MHz, CDCl_3_): *δ* 175.2 (CO), 157.4 (Ar-C), 139.3 (Ar-C), 130.9 (Ar-C), 130.3 (Ar-C), 130.3 (Ar-C), 130.1 (Ar-C), 129.6 (Ar-C), 125.8 (Ar-C), 122.6 (Ar-C), 114.9 (Ar-C), 69.0 (CH_2_O), 55.7 (CH_2_CHNH), 52.0 (CH_3_O), 39.9 (CH_2_CHNH). HRMS calculated for C_17_H_19_BrNO_3_ [M + H]^+^ 364.0543, found 364.0547.

##### (*S*)-Methyl 2-(2-(acetylthio)acetamido)-3-(4-((3-bromobenzyl)oxy)phenyl)propanoate (**8j**)

Tan viscous oil, 1.63 g (85%). ^1^H NMR (400 MHz, CDCl_3_): *δ* 7.56 (1H, s, Ar-H), 7.42 (1H, d, *J =* 8.0 Hz, Ar-H), 7.31 (1H, d, *J =* 7.7 Hz, Ar-H), 7.22 (1H, t, *J =* 7.8 Hz, Ar-H), 6.98 (2H, d, *J =* 8.6 Hz, Ar-H), 6.84 (2H, d, *J =* 8.6 Hz, Ar-H), 6.61 (1H, d, *J =* 7.7 Hz, NH), 4.97 (2H, s, CH_2_O), 4.75 (1H, dt, *J =* 7.8, 5.9 Hz, CH_2_CHNH), 3.69 (3H, s, CH_3_O), 3.51 (2H, dd, *J =* 24.1, 14.8 Hz, CH_2_S), 3.07 (1H, dd, *J* = 13.8, 5.5 Hz, CH_A_H_B_CHNH), 2.98 (1H, dd, *J* = 13.8, 6.1 Hz, CH_A_H_B_CHNH), 2.32 (3H, s, CH_3_CO). ^13 ^C NMR (100 MHz, CDCl_3_): *δ* 195.2 (COS), 171.5 (CO_2_), 167.6 (CONH), 157.5 (Ar-C), 139.3 (Ar-C), 131.0 (Ar-C), 130.3 (Ar-C), 130.3 (Ar-C), 130.1 (Ar-C), 128.2 (Ar-C), 125.8 (Ar-C), 122.6 (Ar-C), 114.8 (Ar-C), 69.0 (CH_2_O), 53.5 (CH_2_CHNH), 52.3 (CH_3_O), 36.7 (CH_2_CHNH), 32.7 (CH_2_S), 30.1 (CH_3_CO). HRMS calculated for C_21_H_22_BrNNaO_5_S [M + Na]^+^ 502.0294, found 502.0291.

##### (*S*)-3-(4-((3-Bromobenzyl)oxy)phenyl)-2-(2-mercaptoacetamido)propanoic acid (**2j**)

Off white solid, 0.89 g (70%). ^1^H NMR (400 MHz, CD_3_OD): *δ* 7.57 (1H, s, Ar-H), 7.43 (1H, d, *J =* 8.0 Hz, Ar-H), 7.37 (1H, d, *J =* 7.7 Hz, Ar-H), 7.25 (1H, t, *J =* 7.8 Hz, Ar-H),7.13 (2H, d, *J =* 8.6 Hz, Ar-H), 6.89 (2H, d, *J =* 8.6 Hz, Ar-H), 5.01 (2H, s, ArCH_2_O), 4.61 (1H, dd, *J* = 8.2, 5.2 Hz, CH_2_CHNH), 3.14 (1H, dd, *J =* 13.8, 5.5 Hz, CH_A_H_B_CHNH), 3.12 (2H, s, CH_2_SH), 2.94 (1H, dd, *J* = 14.0, 8.0 Hz, CH_A_H_B_CHNH). ^13 ^C NMR (100 MHz, CD_3_OD): *δ* 174.3 (CO_2_H), 172.9 (CONH), 158.9 (Ar-C), 141.5 (Ar-C), 131.8 (Ar-C), 131.5 (Ar-C), 131.3 (Ar-C), 131.3 (Ar-C), 130.6 (Ar-C), 127.1 (Ar-C), 123.4 (Ar-C), 115.9 (Ar-C), 69.9 (PhCH_2_O), 55.3 (CH_2_CHNH), 37.5 (CH_2_CHNH), 28.0 (CH_2_SH). HRMS calculated for C_18_H_17_BrNO_4_S [M-H]^−^ 422.0067, found 422.0071.

##### (*S*)-Methyl 3-(4-((4-bromobenzyl)oxy)phenyl)-2-((tert-butoxycarbonyl)amino)propanoate (**5k**)

Off white solid, 2.90 g (92%). ^1^H NMR (500 MHz, CDCl_3_): *δ* 7.48 (2H, d, *J =* 8.3 Hz, Ar-H), 7.27 (2H, d, *J =* 8.3 Hz, Ar-H), 7.01 (2H, d, *J =* 8.5 Hz, Ar-H), 6.85 (2H, d, *J =* 8.5 Hz, Ar-H), 4.96 (2H, s, CH_2_O), 4.94 (1H, d, *J =* 7.5 Hz, NH), 4.52 (1H, q, *J* = 8.0 Hz, CH_2_CHNH), 3.68 (3H, s, CH_3_O), 3.03 (1H, dd, *J =* 13.8, 5.8 Hz, CH_A_H_B_CHNH), 2.97 (1H, dd, *J =* 13.8, 6.0 Hz, CH_A_H_B_CHNH), 1.39 (9H, s, C(CH_3_)_3_). ^13 ^C NMR (125 MHz, CDCl_3_): *δ* 172.4 (CO_2_CH_3_), 157.6 (NHCO_2_), 155.1 (Ar-C), 136.0 (Ar-C), 131.7 (Ar-C), 130.3 (Ar-C), 129.0 (Ar-C), 128.5 (Ar-C), 121.8 (Ar-C), 114.9 (Ar-C), 79.9 (C(CH_3_)_3_), 69.2 (CH_2_O), 54.5 (CH_2_CHNH), 52.2 (CH_3_O), 37.5 (CH_2_CHNH), 28.3 (C(CH_3_)_3_). HRMS calculated for C_22_H_26_BrNNaO_5_ [M + Na]^+^ 486.0887, found 486.0896.

##### (*S*)-Methyl 2-amino-3-(4-((4-bromobenzyl)oxy)phenyl)propanoate (**6k**)

Brown solid, 1.78 g (98%). ^1^H NMR (500 MHz, CDCl_3_): *δ* 7.50 (2H, d, *J =* 8.3 Hz, Ar-H), 7.30 (2H, d, *J =* 8.4 Hz, Ar-H), 7.10 (2H, d, *J =* 8.5 Hz, Ar-H), 6.88 (2H, d, *J =* 8.5 Hz, Ar-H), 4.96 (2H, s, CH_2_O), 3.69 (3H, s, CH_3_O), 3.67 (1H, CH_2_CHNH_2_), 3.01 (1H, dd, *J =* 13.6, 5.1 Hz, CH_A_H_B_CHNH), 2.81 (1H, dd, *J =* 13.7, 7.7 Hz, CH_A_H_B_CHNH). ^13 ^C NMR (125 MHz, CDCl_3_): *δ* 175.6 (CO), 157.6 (Ar-C), 136.2 (Ar-C), 131.8 (Ar-C), 130.5 (Ar-C), 129.8 (Ar-C), 129.2 (Ar-C), 122.0 (Ar-C), 115.1 (Ar-C), 69.4 (CH_2_O), 56.0 (CH_2_CHNH), 52.1 (CH_3_O), 40.2 (CH_2_CHNH). HRMS calculated for C_17_H_19_BrNO_3_ [M + H]^+^ 364.0543, found 364.0546.

##### (*S*)-Methyl 2-(2-(acetylthio)acetamido)-3-(4-((4-bromobenzyl)oxy)phenyl)propanoate (**8k**)

Tan viscous oil, 1.59 g (83%). ^1^H NMR (400 MHz, CDCl_3_): *δ* 7.48 (2H, d, *J =* 8.3 Hz, Ar-H), 7.27 (2H, d, *J =* 8.3 Hz, Ar-H), 6.97 (2H, d, *J =* 8.5 Hz, Ar-H), 6.83 (2H, d, *J =* 8.5 Hz, Ar-H), 6.60 (1H, d, *J =* 7.5 Hz, NH), 4.96 (2H, s, CH_2_O), 4.75 (1H, dt, *J =* 7.6, 5.9 Hz, CH_2_CHNH), 3.70 (3H, s, CH_3_O), 3.51 (2H, dd, *J =* 23.1, 14.8 Hz, CH_2_S), 3.06 (1H, dd, *J =* 13.8, 5.5 Hz, CH_A_H_B_CHNH), 2.97 (1H, dd, *J =* 13.8, 6.1 Hz, CH_A_H_B_CHNH), 2.31 (3H, s, CH_3_CO). ^13 ^C NMR (100 MHz, CDCl_3_): *δ* 195.2 (COS), 171.5 (CO_2_), 167.6 (CONH), 157.6 (Ar-C), 135.9 (Ar-C), 131.7 (Ar-C), 130.3 (Ar-C), 129.0 (Ar-C), 128.1 (Ar-C), 121.8 (Ar-C), 114.8 (Ar-C), 69.2 (CH_2_O), 53.5 (CH_2_CHNH), 52.4 (CH_3_O), 36.7 (CH_2_CHNH), 32.7 (CH_2_S), 30.1 (CH_3_CO). HRMS calculated for C_21_H_22_BrNNaO_5_S [M + Na]^+^ 502.0294, found 502.0289.

##### (*S*)-3-(4-((4-Bromobenzyl)oxy)phenyl)-2-(2-mercaptoacetamido)propanoic acid (**2k**)

Off white solid, 0.87 g (68%). ^1^H NMR (500 MHz, CD_3_OD): *δ* 7.50 (2H, d, *J =* 8.3 Hz, Ar-H), 7.33 (2H, d, *J =* 8.3 Hz, Ar-H), 7.13 (2H, d, *J =* 8.5 Hz, Ar-H), 6.89 (2H, d, *J =* 8.5 Hz, Ar-H), 5.01 (2H, s, ArCH_2_O), 4.61 (1H, dd, *J =* 8.3, 5.3 Hz, CH_2_CHNH), 3.14 (1H, dd, *J =* 13.8, 5.5 Hz, CH_A_H_B_CHNH), 3.12 (2H, s, CH_2_SH), 2.94 (1H, dd, *J =* 14.0, 8.0 Hz, CH_A_H_B_CHNH). ^13 ^C NMR (125 MHz, CD_3_OD): *δ* 174.4 (CO_2_H), 172.9 (CONH), 159.0 (Ar-C), 138.2 (Ar-C), 132.6 (Ar-C), 131.5 (Ar-C), 130.6 (Ar-C), 130.4 (Ar-C), 122.5 (Ar-C), 116.0 (Ar-C), 70.2 (PhCH_2_O), 55.3 (CH_2_CHNH), 37.5 (CH_2_CHNH), 28.0 (CH_2_SH). HRMS calculated for C_18_H_17_BrNO_4_S [M-H]^−^ 422.0067, found 422.0073.

## Supplementary Material

Supplemental Material

Supplemental Material

## Note

1 The washing step with water can be reduced to once if freeze-dry equipment is used to evaporate acetic acid but this process can take longer time as the sample should be kept overnight.
