# Supplementary material for: Synthesis and enzyme-based evaluation of analogues L-tyrosine thiol carboxylic acid inhibitor of metallo-β-lactamase IMP-1
Source: J Enzyme Inhib Med Chem. 2019 Aug 11;34(1):1414–25. doi: 10.1080/14756366.2019.1651314 (PMC8853707; doi:10.1080/14756366.2019.1651314)
Supplement: Supplemental Material [file IENZ_A_1651314_SM7078.pdf]

Protein Expression

Purification of IMP-1 was carried out by the procedure below:

Materials:

1. Luria bertani (LB) Broth (Schott Bottles Flask)
2. LB Agar
3. Petri dish
4. Kanamycin Stock (50 mg/ml)
5. IPTG Stock (100 mM)
6. Autoclave flask
7. Shaker
8. Water Bath (42 ^{o}C)
9. BL21(DE3) competent cells

Recipes:

Day 0

LB Agar (1L)

10 grams tryptone
5 grams of yeast extract
10 grams of NaCl
Adjust pH to 7.0 with NaOH (Adjust to pH 7.0 is not necessary according to some people)
Add 15 g agar
Melt in microwave. Top up to 1L
Autoclave

LB Broth (2L)

20 grams tryptone
10 grams of yeast extract
20 grams of NaCl
Adjust to pH 7.0 (Adjust to pH 7.0 is not necessary). Then, autoclave.
Tryptone (20 g), yeast extract (10 g) and NaCl (20 g) dissolved in milliQ water (2 L) then the soulution transferred to four big flask (2L). each one 500 ml.

Kanamycin (50 mg/ml) Stock Solution

0.5 g Kanamycin (10 ml MilliQ water)
Filter sterilized with 0.22 micron Aliquot
Store at -20 - -70 °C

Addition (50 µg/ml, dilution factor 1000)

Total volume of LB Broth/flask is 550 ml

550/1000 = 0.55 ml
So 0.55 ml of 50 mg/ml Kanamycin need to be added to 550 ml LB Broth big flask.

IPTG (Isoproyl-β-D-thiogalacto-pyranoside) (100 mM, 25 ml) Stock Solution

Mw: 238.3 g/mole the mass = C x V x Mw = 0.1 M x 0.025L x 238.3 = 0.595 g
So 0.595 g in 25 ml MilliQ water.
Filter sterilized with 0.22 micron
Short term: store at 4°C or -20°C for long term.

Addition (1 mM, dilution factor 100)

Total volume of LB Broth/flask is 550 ml
550/100 = 5.5 ml
So 5.5 ml of 100 mM IPTG need to be added to 550 ml LB Broth big flask.

ZnCl2 (500 mM, 10 ml) Stock Solution

Mw: 136.3 g/mole the mass = C x V x Mw = 0.5 M x 0.01L x 136.3 = 0.6815 g
So 0.6815 g in 10 ml MilliQ water.
Filter sterilized with 0.22 micron
Store at 4 °C.

Addition (25µ M, dilution factor 20000)

Total volume of LB Broth/flask is 550 ml
550/20000 = 0.0275 ml = 28 µL.
So 28 µL of 500 mM ZnCl2 need to be added to 550 ml LB Broth big flask.

Autoclave

1 ml tip box
200 µL tip box
flask
Caps for flask

Day 1

Transformation

Make sure that water bath is set to **42°C** prior to transformation

1. In the biological hood, 2 µL Plasmid + 50-100 µL BL21 (DE3) comptent cell
2. Leave on ice for 25-30 mins
3. Heat shock at 42 ^{o}C 40-45sec
4. Immediately, place on ice for 2 mins
5. Add 750 µL fresh LB broth (no Kanamycin to transformed cells)

6. Incubate for 1-1.5 hour at 37 ^{o}C shaker with agitation (leave everything clean and turn off the biological hood)
7. Slow spin (remove some supernatant). This step carried out through centrifuge 60 sec and another water eppendorf tube the same weight needs to be used for balancing, then suck about 500 µL from the supernatant to concentrate the cells
8. Resuspend cells by mixing (this concentrates cells)
9. Spread 50-100 µL on LB/Kan plate and spread by using the blue spreader
10. Incubate at 37 ^{o}C overnight (16-18 hours), the plate needs to be turned upside down before incubation to keep the agar on the top, then wrap the plate with parafilm.
11. Turn off the biological hood

Day 2

Inoculation

1. Pick single colonies using tips and inoculate in 4 (50 ml volume) falcon tubes each of them filled with 20 ml of fresh LB broth + 20 µL Kanamycin (50 mg/ml) by throwing the tip inside the falcon tube, each colony can be used for two falcon tubes by picking it with two tips.
   NB. The final concentration of Kanamycin is 50 mg/ml / dilution factor (1000) = **50** µg/ml.

2. Grow at 30 ^{o}C and 200 RPM shaker for 12-16 hours (overgrow will create mutations)
   NB. The 4 falcon tubes should be fixed very well in the shaker using elastic rubbers.

Day 3

Enzyme Expression

1. 500 µL of Kanamycin (50 mg/ml) stock needs to be added to 0.5L LB broth (sterilized in the autoclave).

NB. We need 4 big flasks to be used for the 4 falcon tubes.

2. Then add the 20 ml falcon tube contents including the tip (starter culture) to the big flask containing 0.5L LB broth and 500 µL of Kanamycin.

3. Shake the 4 big flasks in the incubator at 37°C and 200 RPM for 1 hour. The 4 flasks need to be fixed very well in the incubator using elastic rubbers and 4 elastic chains.

4. Then add 28 µL of 500 mM $ZnCl_2$ stock to each 550 ml big flask.

NB. Before adding the 5.5 ml IPTG, we need to measure the optical density (OD) for the 4 big flasks and should be in the range 0.5 – 0.8.

To measure the OD, we need to use the spectrophotometer

Steps:

a. Switch on the instrument from the left button
   a. Click the icon UV. Probe 2.31
   b. Choose connect then OK
   c. Put the blank (only LB broth) then hit atuozero
   d. Put the 4 samples (about 1 ml using a certain sterilized pipette into plastic cuvette) from the 4 big flasks (only one for each measurement).
   e. The absorbance should be **0.5 – 0.8** to indicate optimum growth before adding the **IPTG** to induce the bacteria to produce the IMP-1 enzyme.

5. Add 5.5 ml of 100 mM IPTG stock to each 550 ml big flask to induce the enzyme.

6. Collect **1.5 ml** from only one big flask every one hour (1, 2, 3, 4, 5, 6, 7, 8 and 24).
   NB. The temperature needs to be 30 ^{o}C if we need to incubate the culture overnight in LB broth.

Steps of collecting the samples and harvesting the cells

   a. Take a sample every one hour up to 9 samples in eppendorf tubes
   b. To harvest the sample (cells), centrifuge the sample at a maximum speed for 2 min, discard the supernatant. Centrifuge again for 30 sec to discard the remaining supernatant.
   c. Stored cells at -20°C.

7. Transfer the 4 flask contents to 6 centrifuge bottles (bleached and cleaned). The weight of the 6 flasks including their contents and their leads should be the same.

   NB. DO not forget to turn off the incubator.

8. Centrifuge the 6 bottles in the big centrifuge (the purple one) at 5000 RPM for 20 min.
   NB. Do not forget to put the lead, close the big coarse first then the smaller one.

9. Transfer the cells using a certain spatula (like a ruler) from the 4 centrifuge bottles into one new 50 ml falcon tube.

10. Freeze the cells at -20 ^{o}C.

11. In the second day, in the biological hood, to the frozen bacterial cells add little amount of buffer (about 3 plastic pipettes) to be 18 ml total volume approximately.

12. Wait till be defrosted, we can use a normal water bath.

13. Add solid lysozyme 0.2 mg/ml (18 ml x 0.2 mg = 3.6 mg need to be added) to break the cell wall and leave for **30 min** with bacteria.

14. Add a tablet of protease inhibitor (without EDTA) to the falcon tube to stop the lyses effect of the protease enzyme on the proteins including IMP-1 enzyme.

15. Shake very well and leave for 30 min.
    NB. After 30 min it looks like gum.

16. Put the sonicator inside the biological hood, clean the tip with ethanol.

17. Immerse the sonicator tip inside the falcon tube and sonicate for 4 times each time about 2-3 sec till the contents looks like a liquid. Use the ice to cool the cells after each sonication.

    NB. Do not forget to use an ear block and ear lead.

18. Add 1 µg DNAse/ml x 25 ml (the new total volume) = 25 µg to lyses the DNA.

19. Add 0.25 ml $MgCl_2$ (1 M) to the 25 ml falcon tube contents to be 10 mM concentrations.

    NB. $MgCl_2$ helps the effect of DNAse.

20. Leave 20 min in ice, the contents become thinner.

21. Divide the contents into 2 centrifuge tubes (the same mass including the leads)

22. Centrifuge using the black one 12500 RPM for 32 min.

23. Collect the supernatant (the most important part including the IMP-1 enzyme) in a labeled falcon tube (IMP-1 culture).

24. Filter the supernatant through 0.22 micron.
    NB. If the filtration is too slow we can add another amounts of both DNAse and $MgCl_2$ and leave overnight then centrifuge again.

25. Concentrate the sample using Millipore (Amicon ultra) centrifugal filter devices, for 25 min at 5000 speed (balancing tubes i.e. the same mass including the leads of the tubes) because we need less than 5 ml total volume of the sample to be injected to FPLC to get good resolution.

    NB. The Millipore tubes need to be washed firstly with water to get red off polyethylene glycol and azides by centrifuging for 5 min using water. Also, after we finish using the

tubes, we need to rinse the tubes with water, centrifuge, add ethanol and keep in the fridge for the next use.

26. Transfer the sample using a plastic pipette to another 15 ml flacon tube to be ready to be injected into the FPLC.

27. Keep a few drops in an eppendorf tube.

Buffers for the purification of IMP-1

Purification Table

**HEPES A**	**HEPES B**	**HEPES X (ASSAY Buffer)**
50 mM HEPES<br>NO SALT<br>500 µM $ZnCl_2$<br>pH 7, Degas	50 mM HEPES<br>1.0 M NaCl<br>500 µM $ZnCl_2$<br>pH 7, Degas	50 mM HEPES<br>0.1 M NaCl<br>100 µM $ZnCl_2$<br>pH 7, Degas
HEPES 11.91 g<br>NO SALT<br>In one liter of milliQ water	HEPES 11.91 g<br>58.44 g NaCl<br>In one liter of milliQ water	HEPES 11.91 g<br>5.844 g NaCl<br>In one liter of milliQ water
Used in SP Sepharose 16/10 (Amersham Biosciences) along with HEPES B to create an eluting gradient.<br>Used to dialyse out DNAse etc.<br>Used in Gel filtration (superpose 12)	Used in SP Sepharose 16/10 (Amersham Biosciences) along with HEPES A to create an eluting gradient.	Used for assay purposes

Zinc Chloride for Buffers:

ZnCl2 (500 mM, 10 ml) Stock Solution

Mw: 136.3 g/mole the mass = C x V x Mw = 0.5 M x 0.01 L x 136.3 = 0.6815 g
So 0.6815 g in 10 ml MilliQ water.
Filter sterilized with 0.22 micron
Store at 4 ^{o}C.

Addition to HEPES A buffer (500 µM, dilution factor 1000)

Total volume of HEPES A buffer is 1000 ml
1000 ml/1000 (DF) = 1.0 ml.
So 1.0 ml of 500 mM ZnCl2 needs to be added to 1.0L HEPES A buffer.

Addition to HEPES X (Assay Buffer) (100 µM, dilution factor 5000)

Total volume of HEPES X (Assay buffer) is 1000 ml

1000 ml/5000 (DF) = 0.2 ml.

So 0.2 ml of 500 mM ZnCl2 needs to be added to 1.0L HEPES X (Assay buffer).

Mw Table

Substance	Mwt	
HEPES	238.31 g/mol	In a big container
$ZnCl_2$	136.30 g/mol	
NaCl	58.44 g/mol	

SDS Page Gel

SDS page gel is usd to find out the enzyme is pure.

Running Buffer

Methods (5x)

1. About 15 g Tris Base weighed, followed by 72 g Glycine
2. Add 5 g SDS
3. Place above chemicals in a beaker.
4. Add milliQ water to 1L, (pH will be 8.6)
5. Adjusted to pH **8.3** using 32% HCl

Dilution to 1X: Take 200 ml of running buffer and add 800 ml MilliQ water.

Amount of protein to use: 1250 µg/ml (i.e. 1.2 mg/ml)

Stacking Gel (Top)

Water (MilliQ):	5.6 ml
0.5 M TRIS-HCl, pH 6.8	2.5 ml
Bis-acrylamide	0.75 ml
10% SDS	100 µL
10% APS	100 µL
TEMED	10 µL

Separating Gel (bottom)

Water (MilliQ):	4.79 ml
1.5 M TRIS-HCl, pH 8.8	2.5 ml
Bis-acrylamide	2.5 ml
10% SDS	100 µL
10% APS	100 µL
TEMED	10 µL

20 µL of sample + 5 µL of denaturing sample buffer. Aim for 10 µg of protein per lane.

Example

A protein at a concentration of 10 mg/ml = 1 µg/µL
So 10 µL contain 10 µg protein.
Proteins that more concentrated than this (e.g. 4.39 mg/ml) need to be diluted.
Here's how:
4.39 mg/ml protein divided by 1 mg/ml is 4.39 (dilution factor).
So 100 µL (total volume) divided by 4.39df is 22 µL of the concentrated sample needs to be diluted to 100 µL as a total volume.
i.e. so we need to add 22 µL of the concentrated sample, and then add enough water/buffer to make it up to 100 µL.

APS

10% SDS is 0.5 g in 5 ml MilliQ water.
10% APS is 0.1 g in 1 ml or 0.05 g (i.e. 50 mg) in 500 µL
Run at 200 Volts. Running at slower speed takes longer time but gives better resolution.
Coomassie Blue Staining Solution

Destaining Solution

Materials (scale down for 1L solution)
7.5% acetic acid
10% ethanol

Add 300 ml acetic acid and 420 ml 95% ethanol to 3280 ml water.
Transfer to 4L solvent storage bottle.

Fast Performance Liquid Chromatography (FPLC)

Important notices:

1. Keep your eye most of the time on the pressure to avoid to be too high.
2. Every solution must be filtered through 0.22 µM to avoid any particles can be stuck onto the column which can increase the pressure.
3. All used buffers must be degassed to avoid any air bubbles which can increase the pressure.

General Instructions for FPLC

1. Before starting anything, we need to disconnect the super loop (the yellow one) and rinse it very well with water, detergent and water. Try to avoid the air bubbles while reassembling the super loop again. We need to rinse it again also, after using it. Be careful it is very expensive.

NB. The importance of the super loop, it is the area where the sample be loaded in the bottom between the two black lines after the sample injection.

2. All of the column should be stored in ethanol after using them.
3. To use **any new solution**, we need to do pump wash at a pressure 5.000 MPa to get red off any air bubbles and fill the pump with the new solution.
4. After mounting a new column we need to flush it with water firstly to get red off the ethanol in a rate (according to the column instructions).

Procedure

1. Click on **Unicorn**, **System control**, **Manual**, **Pump**, **Pump wash** (it takes about 15 min), On, On then hit **insert**
2. Choose **Alarm&Monitoring**, **Alarm Pressure**, **5 MPa** then hit **insert** and **execute** but after checking the **two tubes immersed** correctly in the **correct solution vessel**.
3. **Dismounting and mounting the column procedures**

a. Pump, flow, 0.2 ml/min then insert (according to the column instructions)
b. Alarm&monitoring, Alarm pressure, 0.3 MPa then insert and hit execute
c. Disconnect the lower connection of the column
d. Put a water syringe to the lower tube (you can leave the syringe for few seconds to fill it with water if required).
e. Put the spring and the lead of the syringe, you need to be very careful in this step.

f. Hit pause till you be ready to mount the new column
g. Disconnect the upper connection of the column
h. Take the column away and clamp it safely.
i. Connect the new column
j. Hit continue
k. Connect the upper connection of the new column drop to drop
l. Disconnect the syringe from the lower part of the column and keep it in a plastic beaker
m. Connect the lower connection of the column (**usually you need a certain connector**).
n. Flush with water to remove all the ethanol (according to the column instructions).

4. Pump wash with buffer then flush the column (according to the column instructions).
5. Inject the 4 ml sample with a 5 ml syringe covered with a small piece of parafilm then get red off any air bubbles.
6. **Flow rate** (according to the column instructions)
7. Gradient **zero flow_path** must be **inject**
8. **Alarm&mon** (according to the column instructions)
9. Volume, other, **acc volume** 2.5 ml to be less than the total volume of the sample to avoid any air bubbles that can be kept in the super loop.

NB. a. We need to wait all of the samples moved to the top of the column (high load) then inject 1 ml water with the same flow rate but volume 0.9 ml.

b. if we need to use another buffer (HEPES B), we need to transfer the line B to the HEPES B vessel after rinsing it with dist water, then as usual flush the pump B with HEBES B.

10. Then now after loading the sample on the top of the column, we need to run the FPLC automatically:

a. Click on **RUN**

b. Choose the correct column method

c. Tick everything in the bottom √ √

d. Change the pressure (according to the column instructions)

e. Sample injection **EMPTY THE LOOP** must be zero

d. hit next

f. wavelength **280** (very important)

g. e.g. Crude IMP-1, Hiload 16/10 sp sepharose, HEPES A, HEPES B, Remarks: type the date then hit next

h. Integrate full report must be ticked, then next, next then type the name as a date then hit **Start**

NB. We need to put the test tubes before starting and after the run, collect the whole test tubes and fridge them.

IMP-1 requires a simple two-step FPLC method<br>Step 1: SP-Sepharose 16/10 (Amersham Biosciences);<br>Step 2: gel filtration, Hiprep 16/60 Sephacryl (superpose 12).

After every purification step, we need to perform two types of assay on the fractions containing the crude protein or enzyme.

Assay A. Protein assay: to quantify how much protein at A280nm absorbance e.g. 400 units activity
B. Kinetic: A235nm absorbance, specific for substrate i.e. penicillin G
Measure the activity of enzyme (rate of change).

A. Protein assay:

1. Turn on the spectrophotometer and the water pump
2. Choose simple reads; wait till the noises stop and the temperature constant
3. Choose **set up** then adjust it to be 280nm
4. Use a clean and air dried quartz cuvette (the plastic one can not be used because the plastic material absorbs light at 280nm i.e. interfere with our samples)
5. Add **990** µL HEPES X (Assay Buffer) to the cuvette then hit autozero (Blanck)
6. Then add **10** µL of the samples, put the lead, mix 4 times and hit read then record the reading of absorbance in nm (measure the odd or even number of fractions only to save time then double the amount in the final calculations).
7. Collect the contents of the cell in a labeled eppendorf tube after each reading.
8. Clean the cell with water and then ethanol and dry with air after every reading
9. Save report as e.g. type the date of assay

B. Kinetic

1. Choose kinetics, then set up 235nm

2. Analyze from the top toolbar, tick both Autocalculate and Display fit

3. Autostore from the top toolbar, choose **the storage on** (**prompt at end**) then hit OK

4. 980 µL HEPES X + 10 µL substrate (filtered penicillin G 50 mM) then add 10 µL sample mix 4 times quickly and read by hitting start then record the slope.
