# Supplementary material for: Synthesis and enzyme-based evaluation of analogues L-tyrosine thiol carboxylic acid inhibitor of metallo-β-lactamase IMP-1
Source: J Enzyme Inhib Med Chem. 2019 Aug 11;34(1):1414–25. doi: 10.1080/14756366.2019.1651314 (PMC8853707; doi:10.1080/14756366.2019.1651314)

Solvent: $CDCl_3$ & NMR: 500 MHz

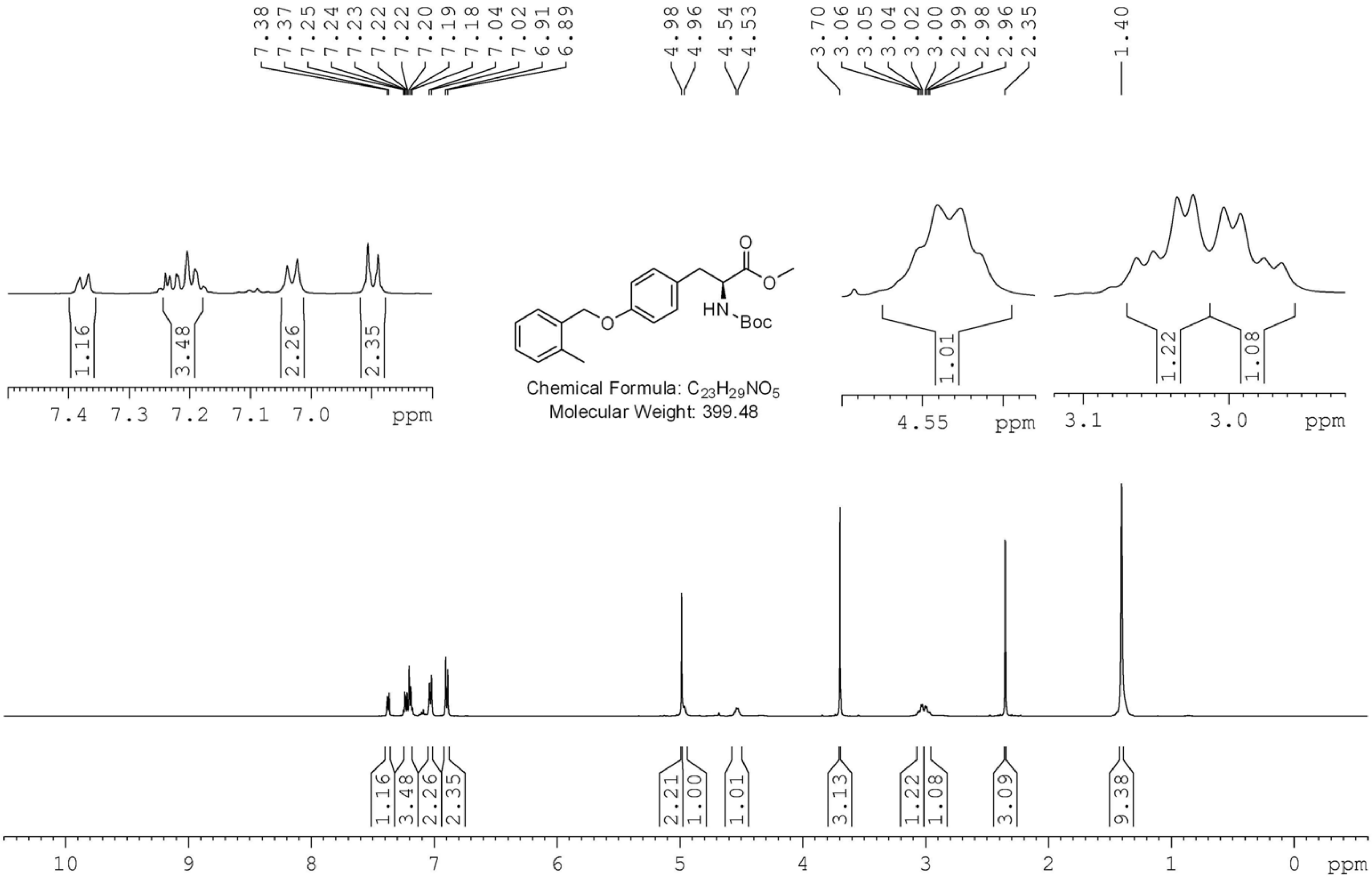

Solvent: $CDCl_3$ & NMR: 125 MHz

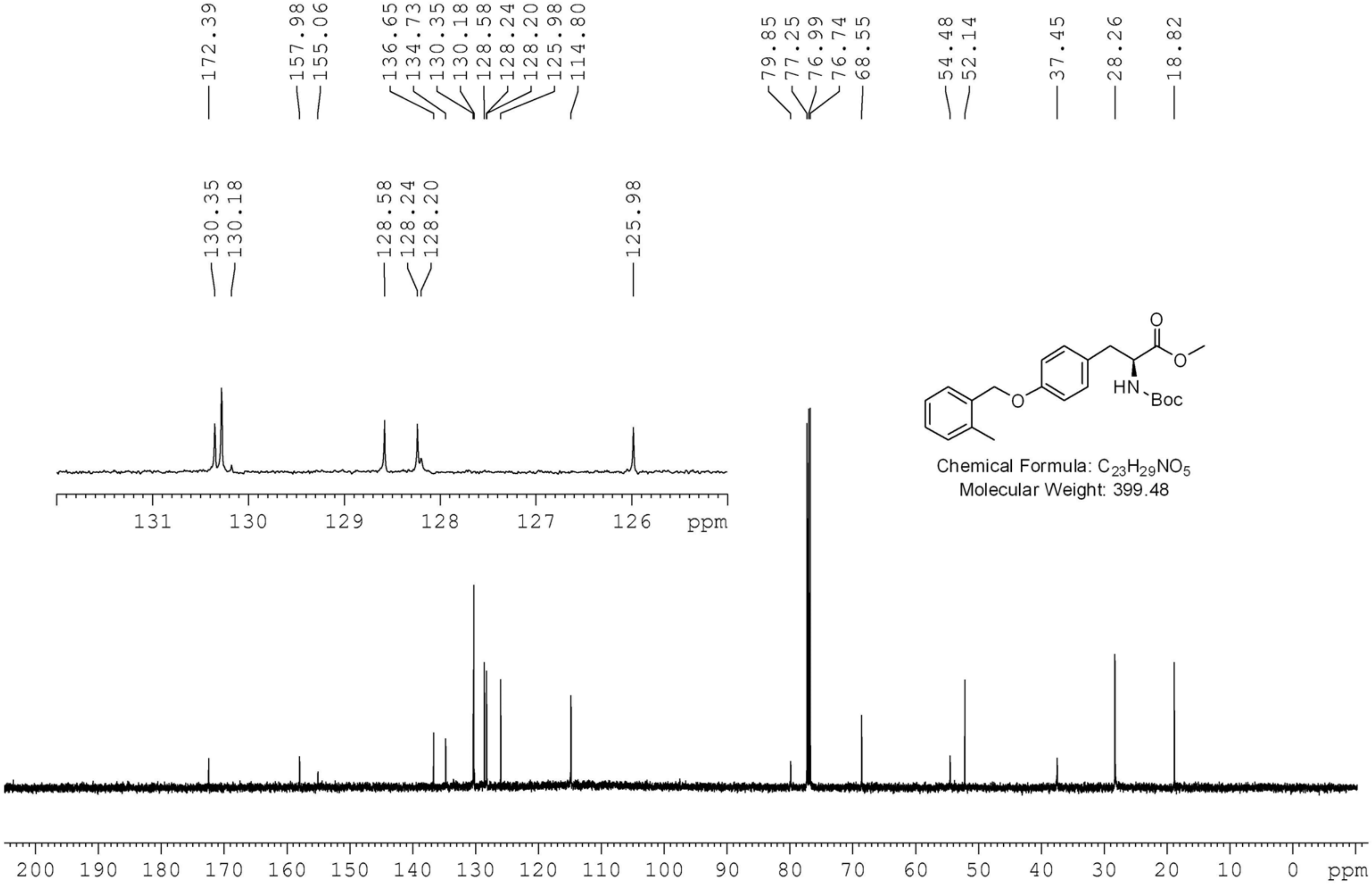

Chemical Formula: $C_{23}H_{29}NO_5$

Molecular Weight: 399.48

Solvent: $CDCl_3$ & NMR: 500 MHz

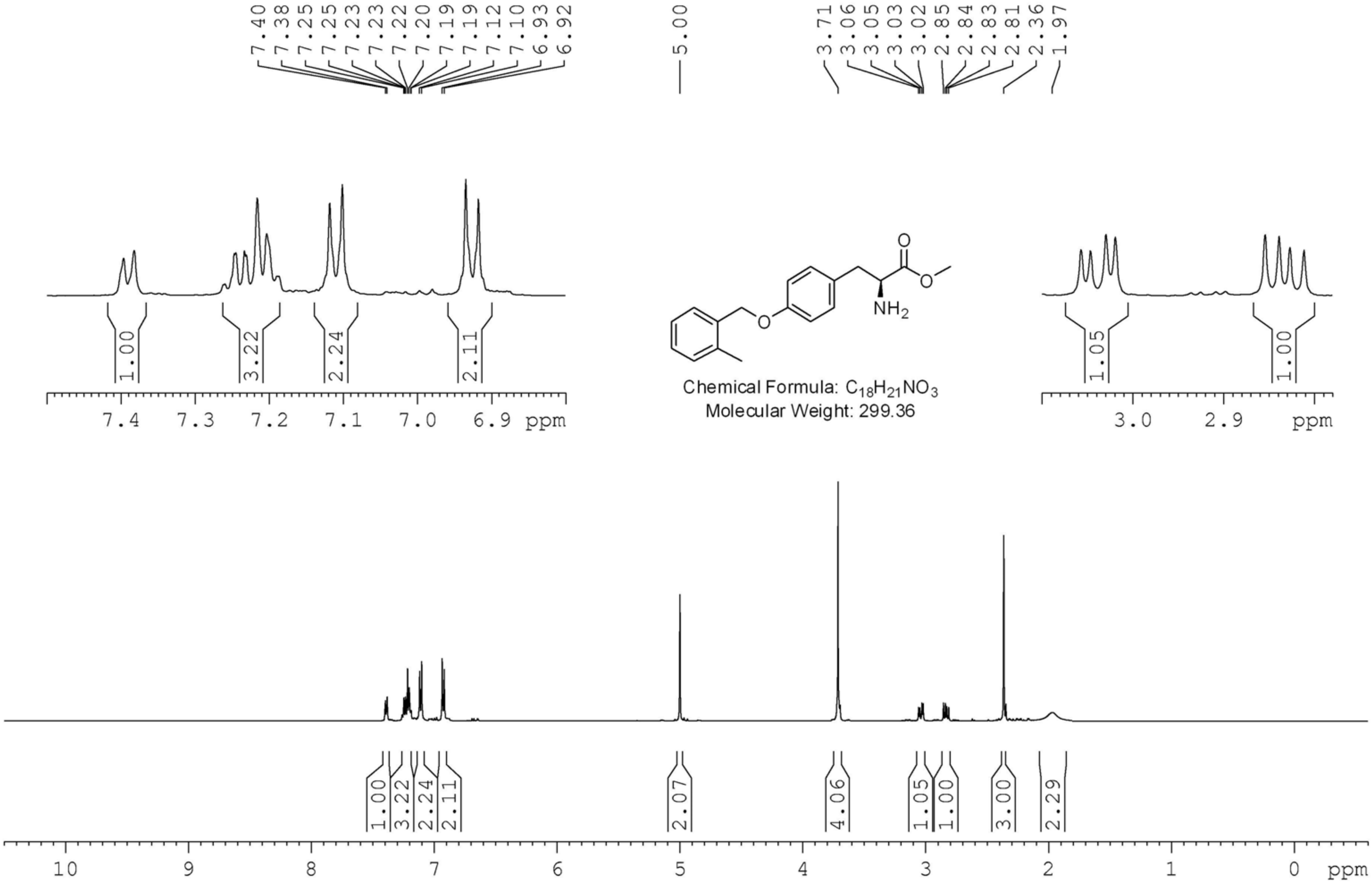

Chemical Formula: $C_{18}H_{21}NO_3$
Molecular Weight: 299.36

Solvent: $CDCl_3$ & NMR: 125 MHz

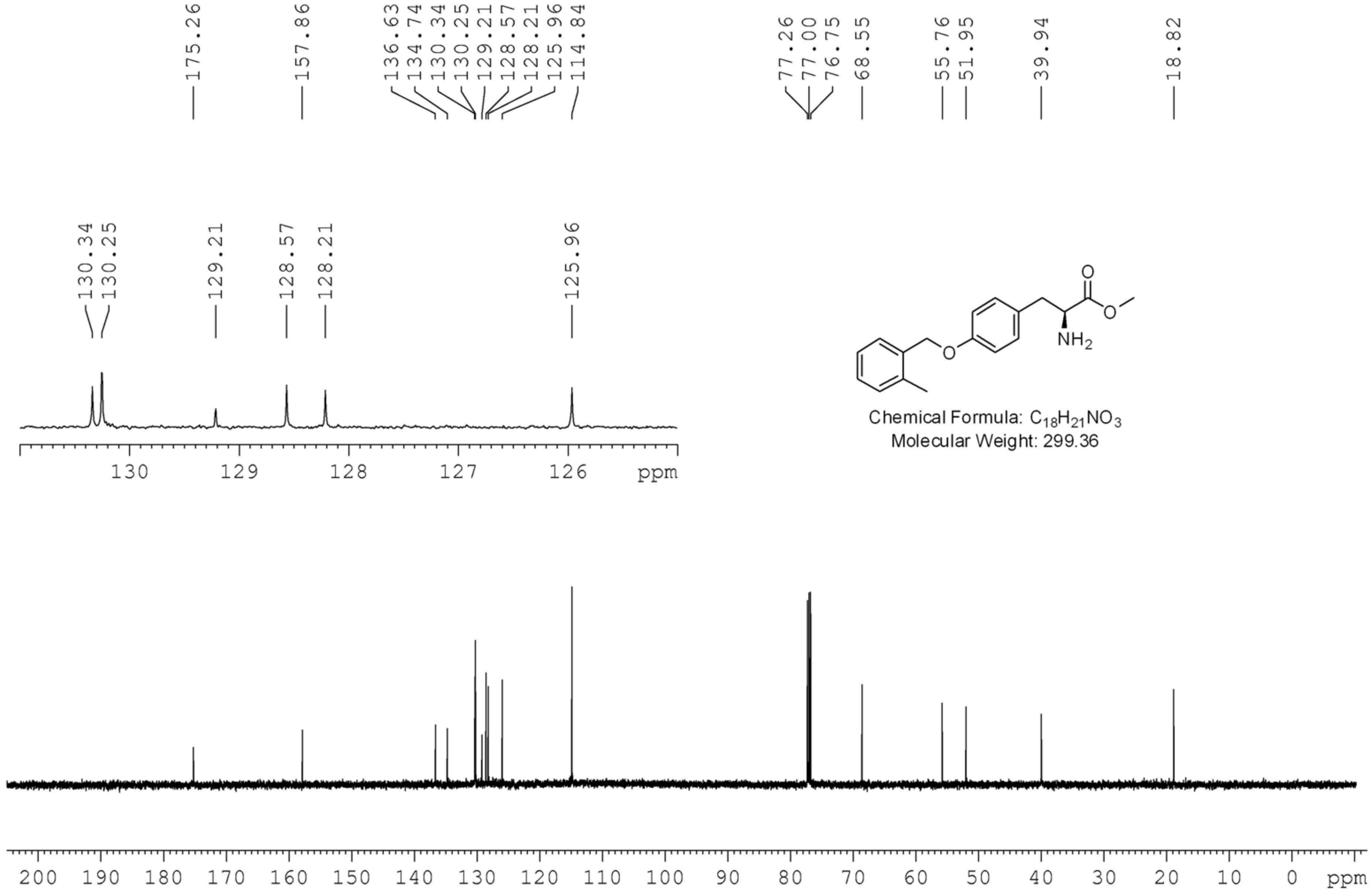

Solvent: $CDCl_3$ & NMR: 400 MHz

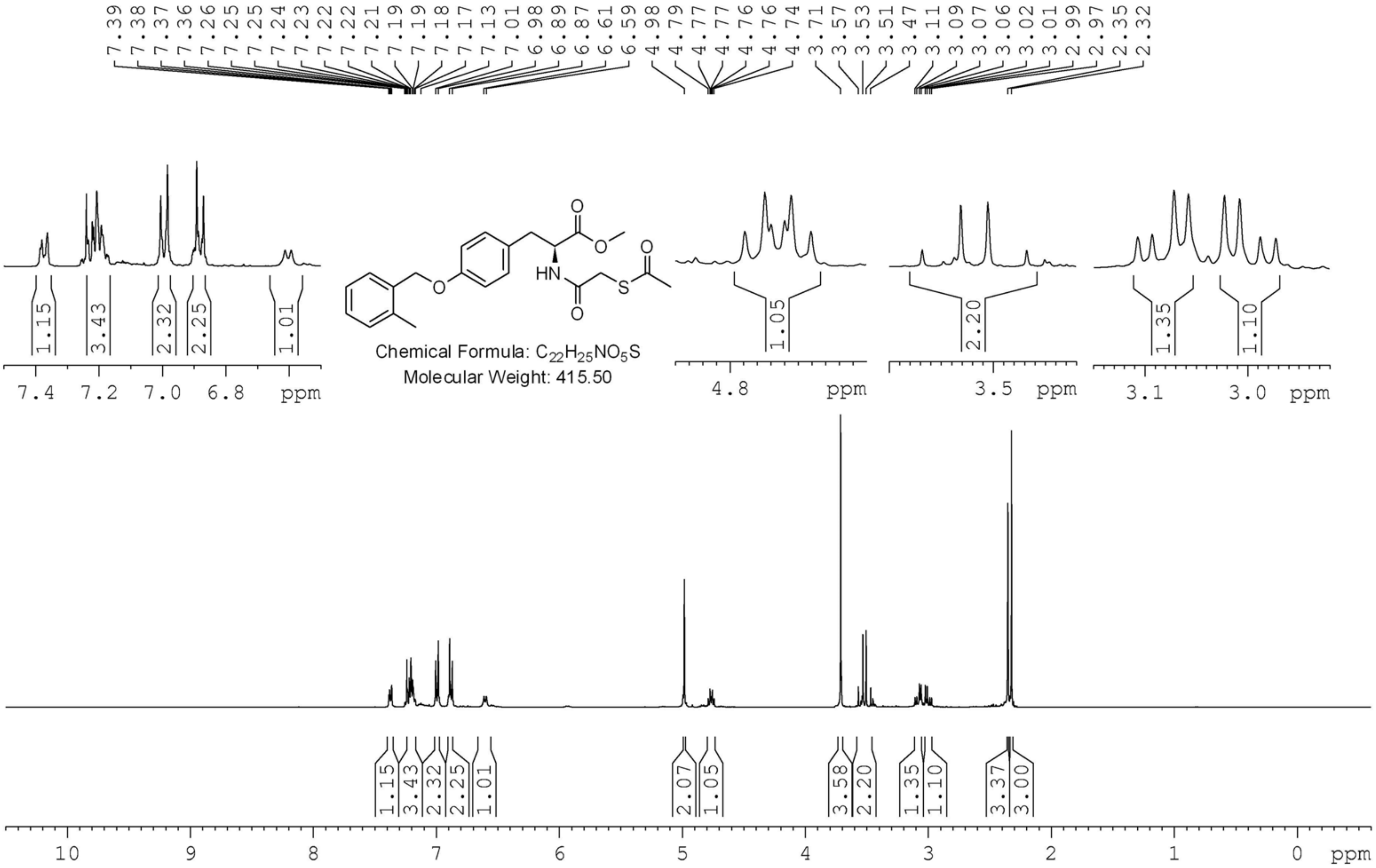

Solvent: $CDCl_3$ & NMR: 100 MHz

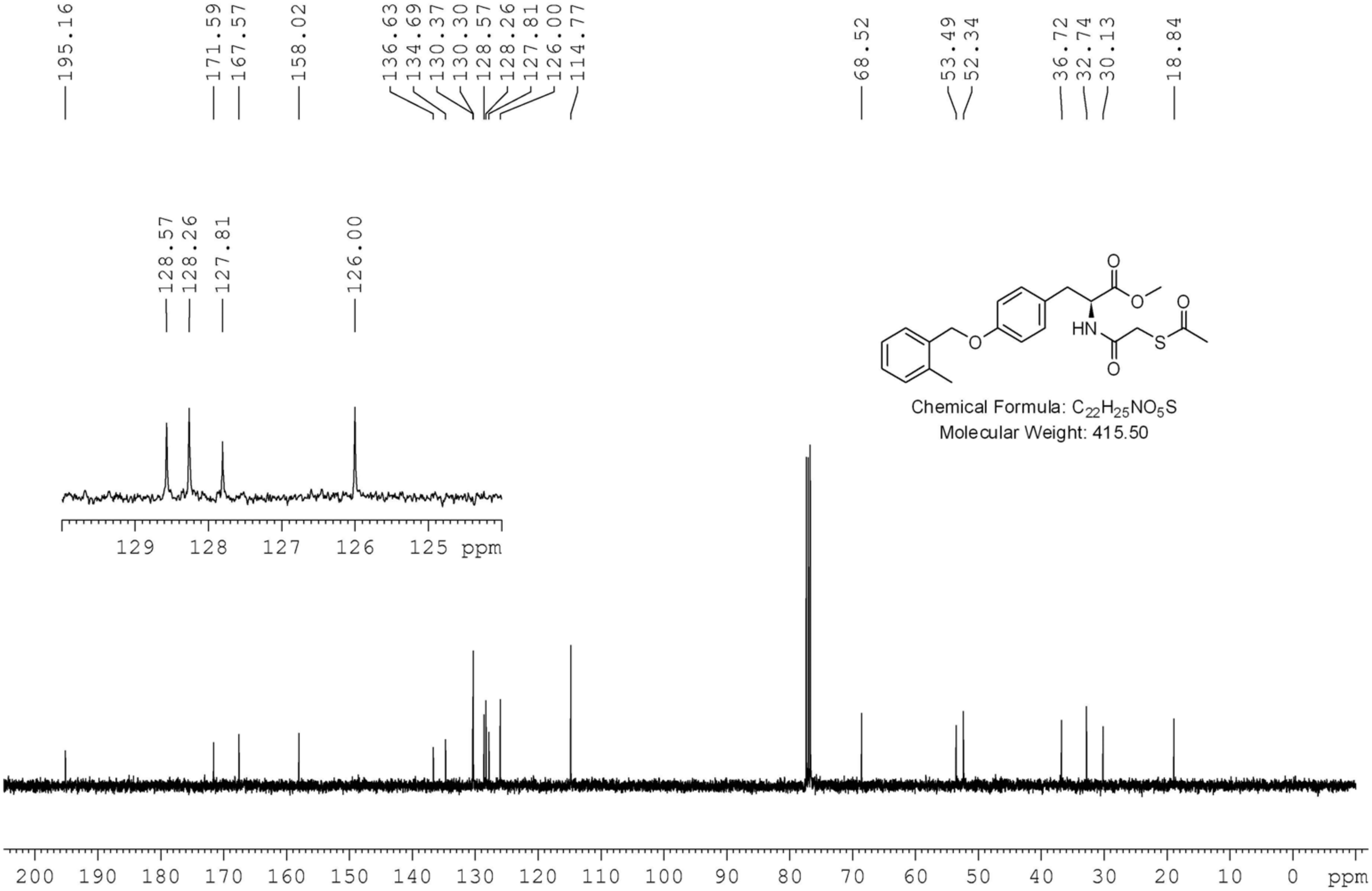

Solvent: CD_3OD & NMR: 400 MHz

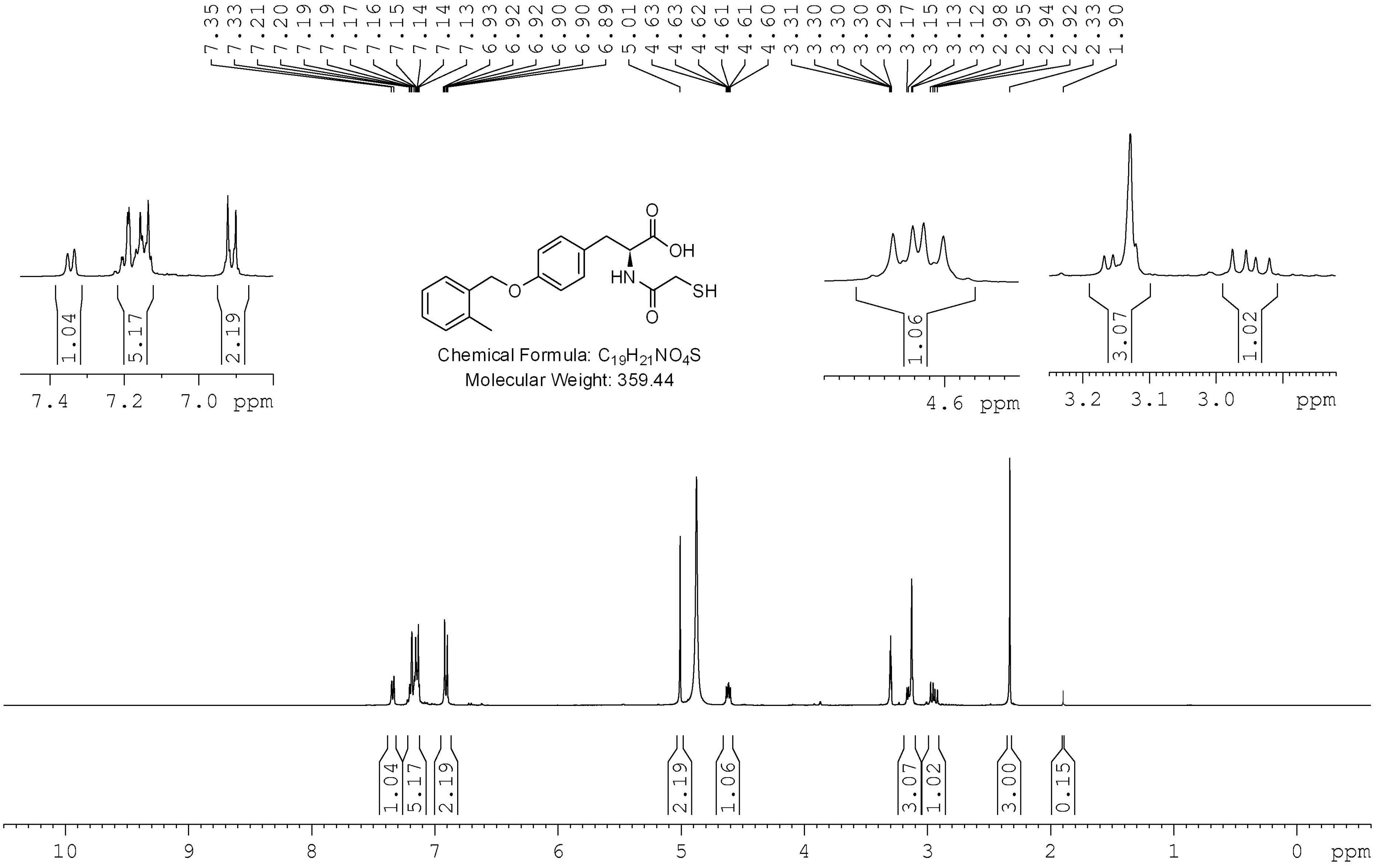

Solvent: CD_3OD & NMR: 100 MHz

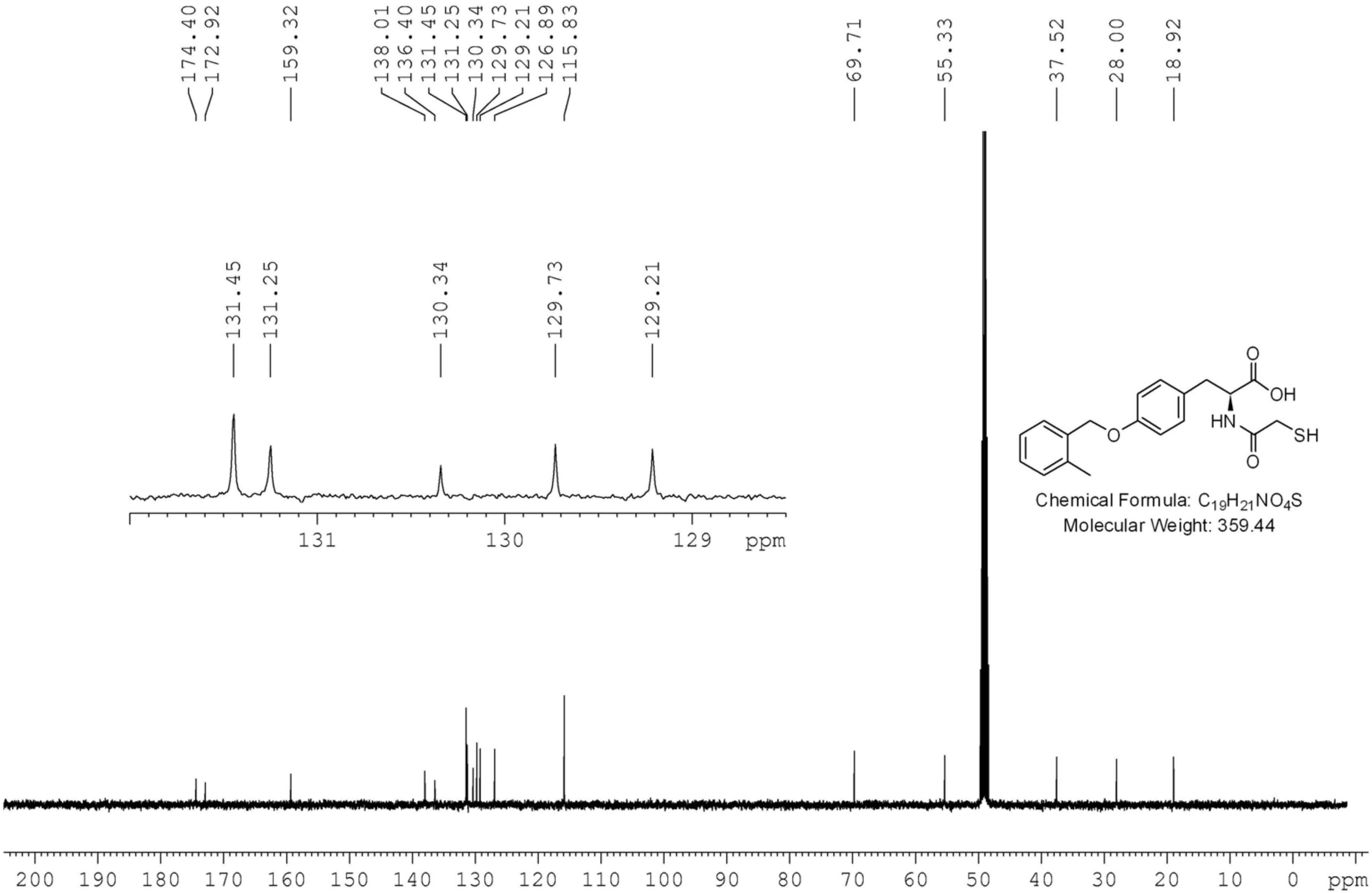

Solvent: $CDCl_3$ & NMR: 500 MHz

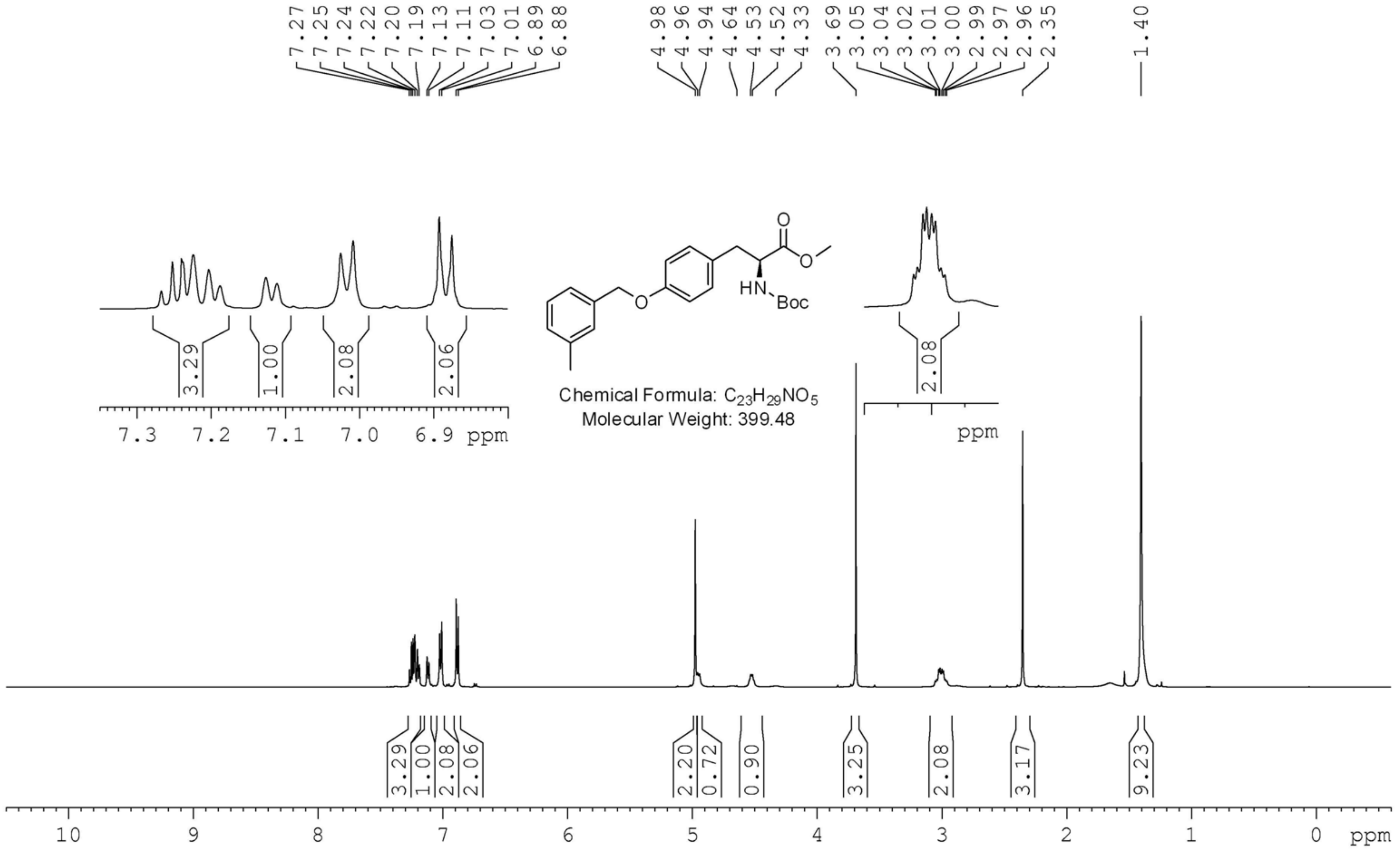

Solvent: $CDCl_3$ & NMR: 125 MHz

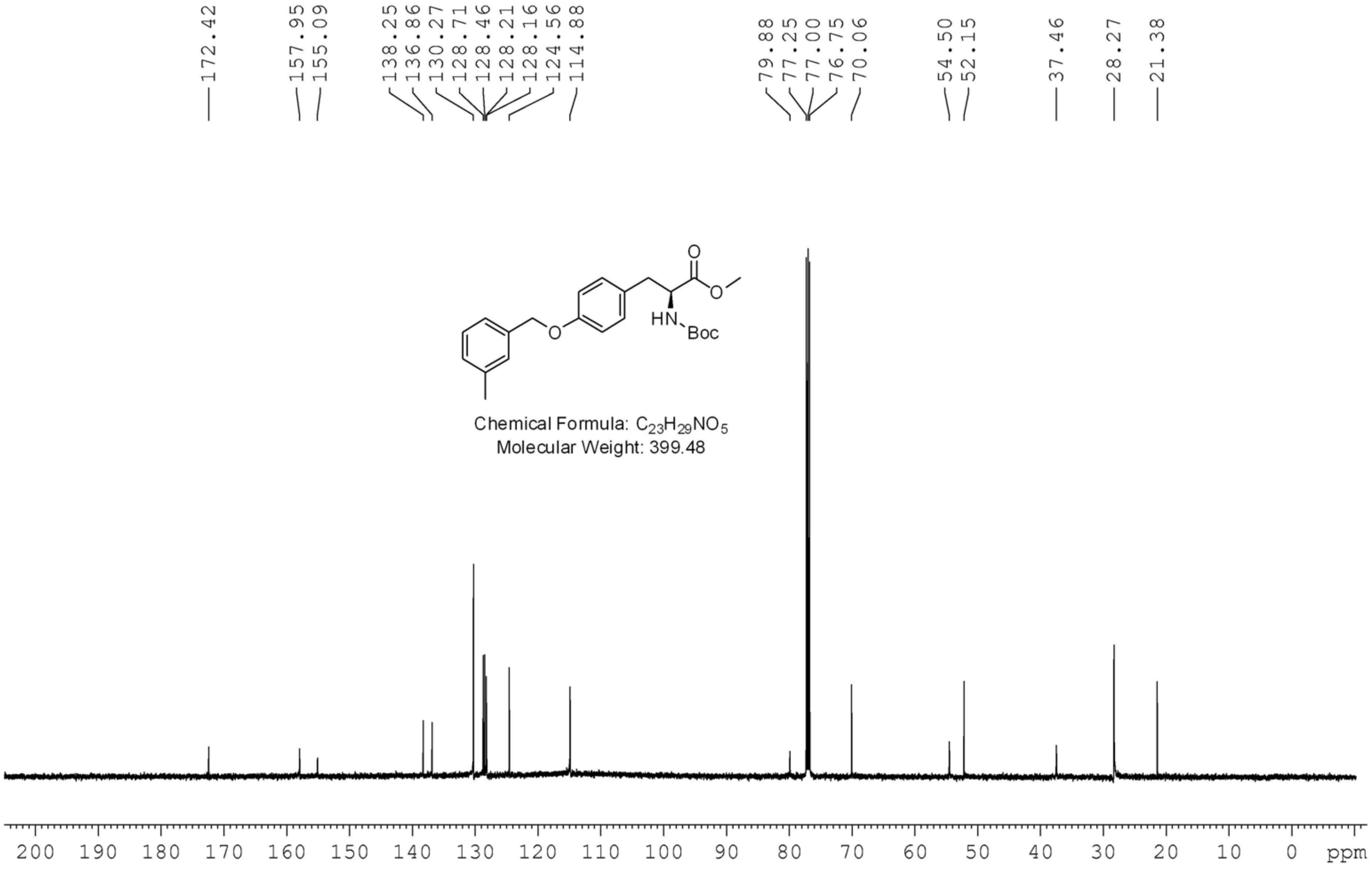

Solvent: $CDCl_3$ & NMR: 500 MHz

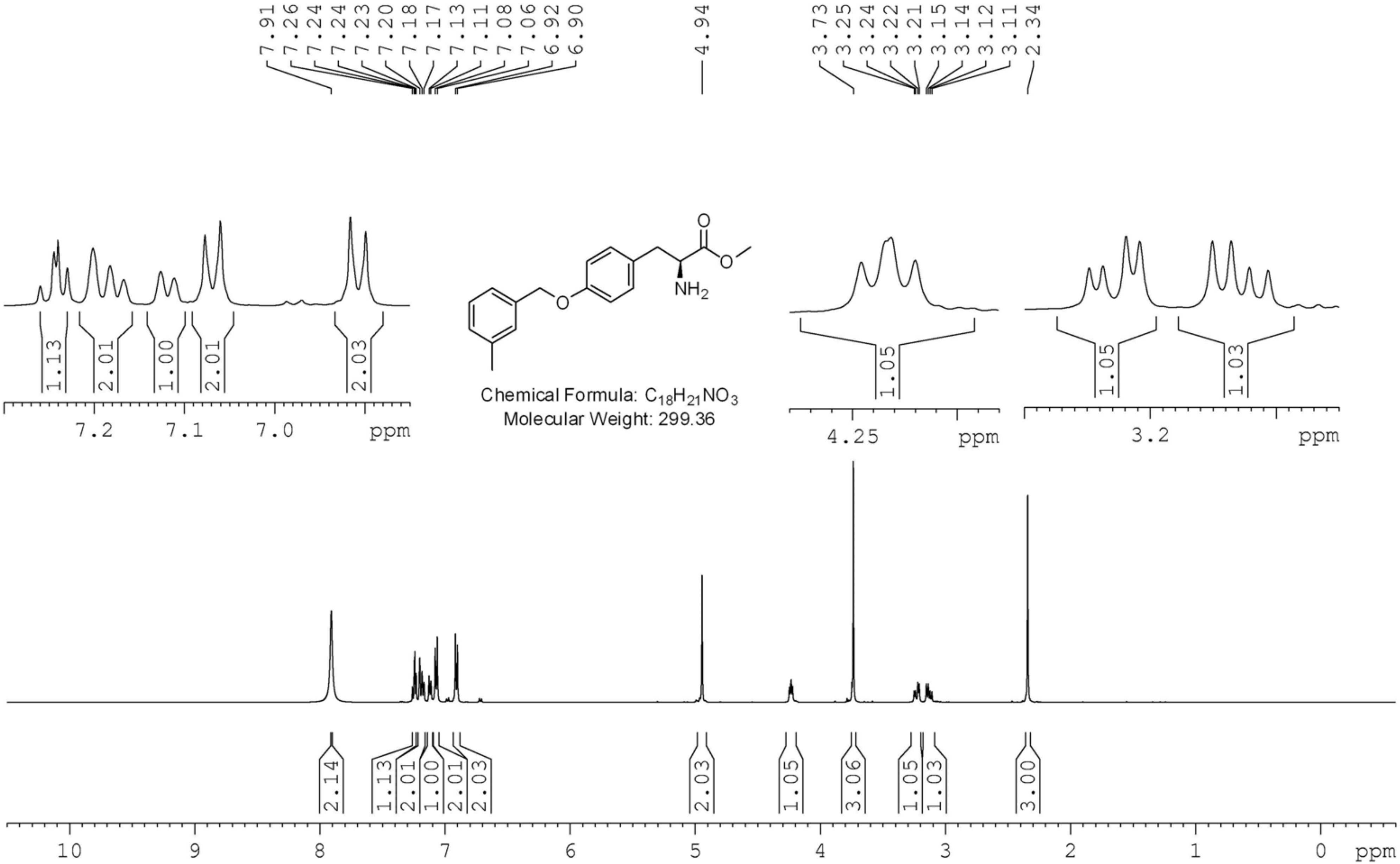

Solvent: $CDCl_3$ & NMR: 125 MHz

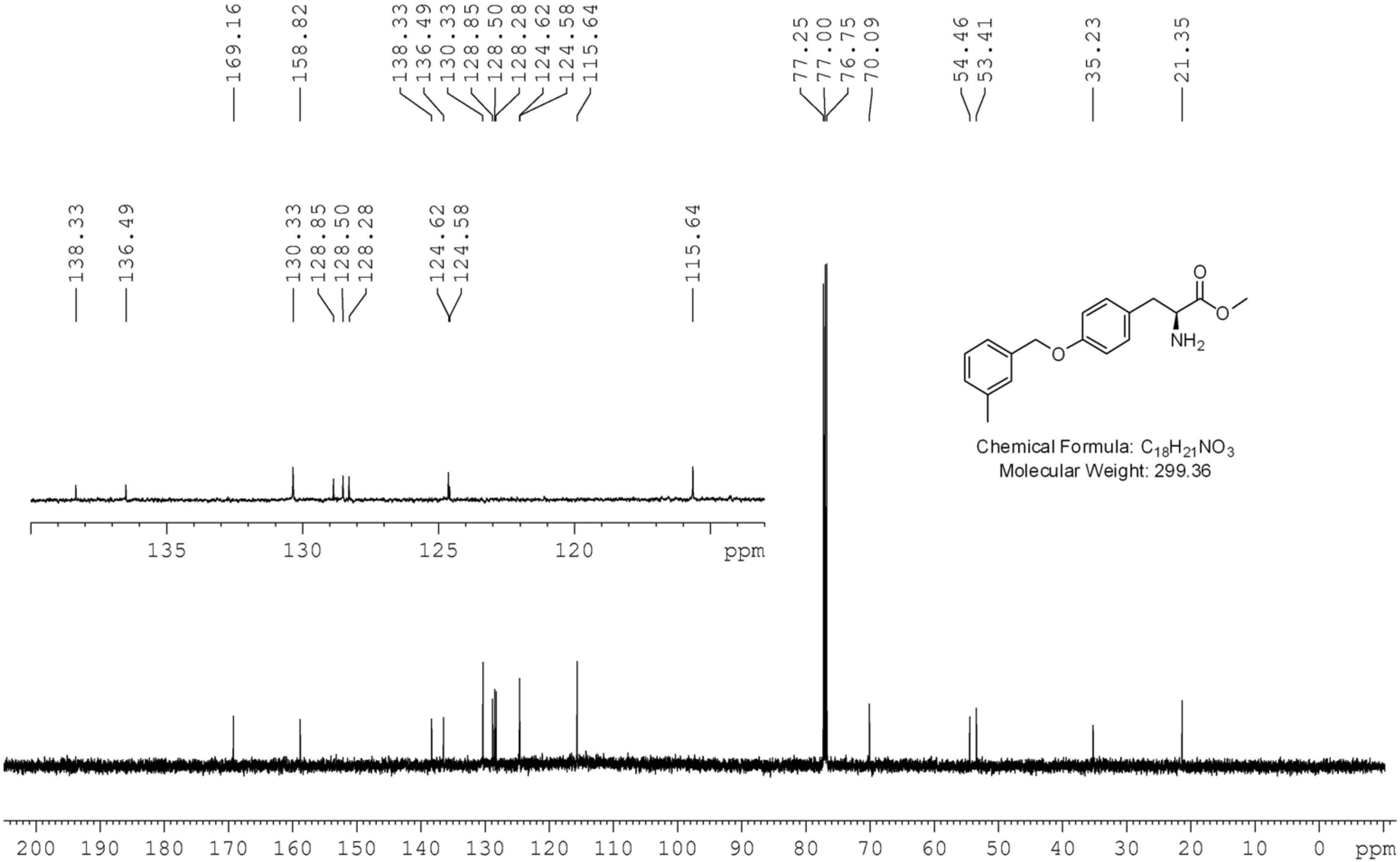

Solvent: $CDCl_3$ & NMR: 500 MHz

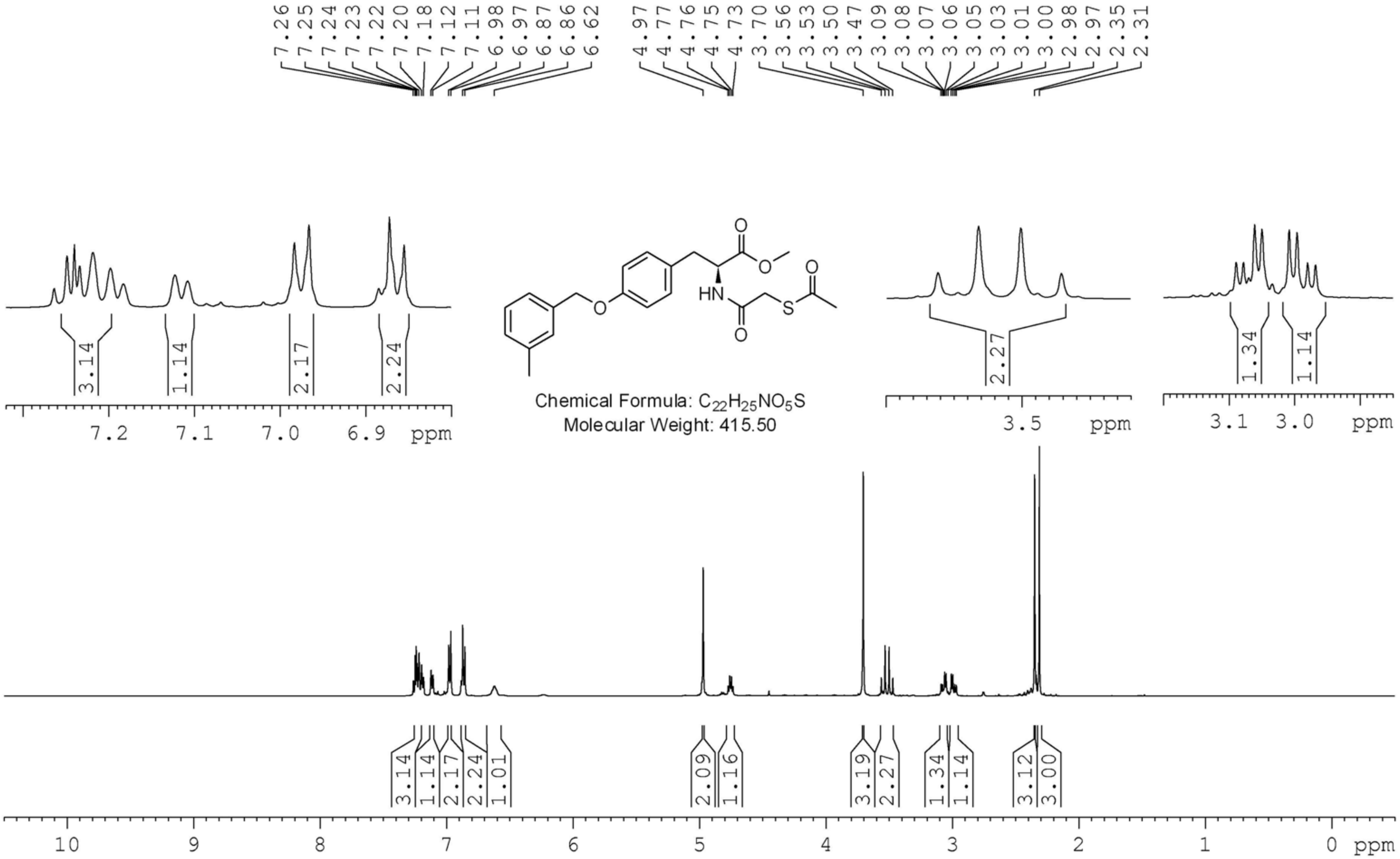

Chemical Formula: $C_{22}H_{25}NO_5S$
Molecular Weight: 415.50

Solvent: $CDCl_3$ & NMR: 125 MHz

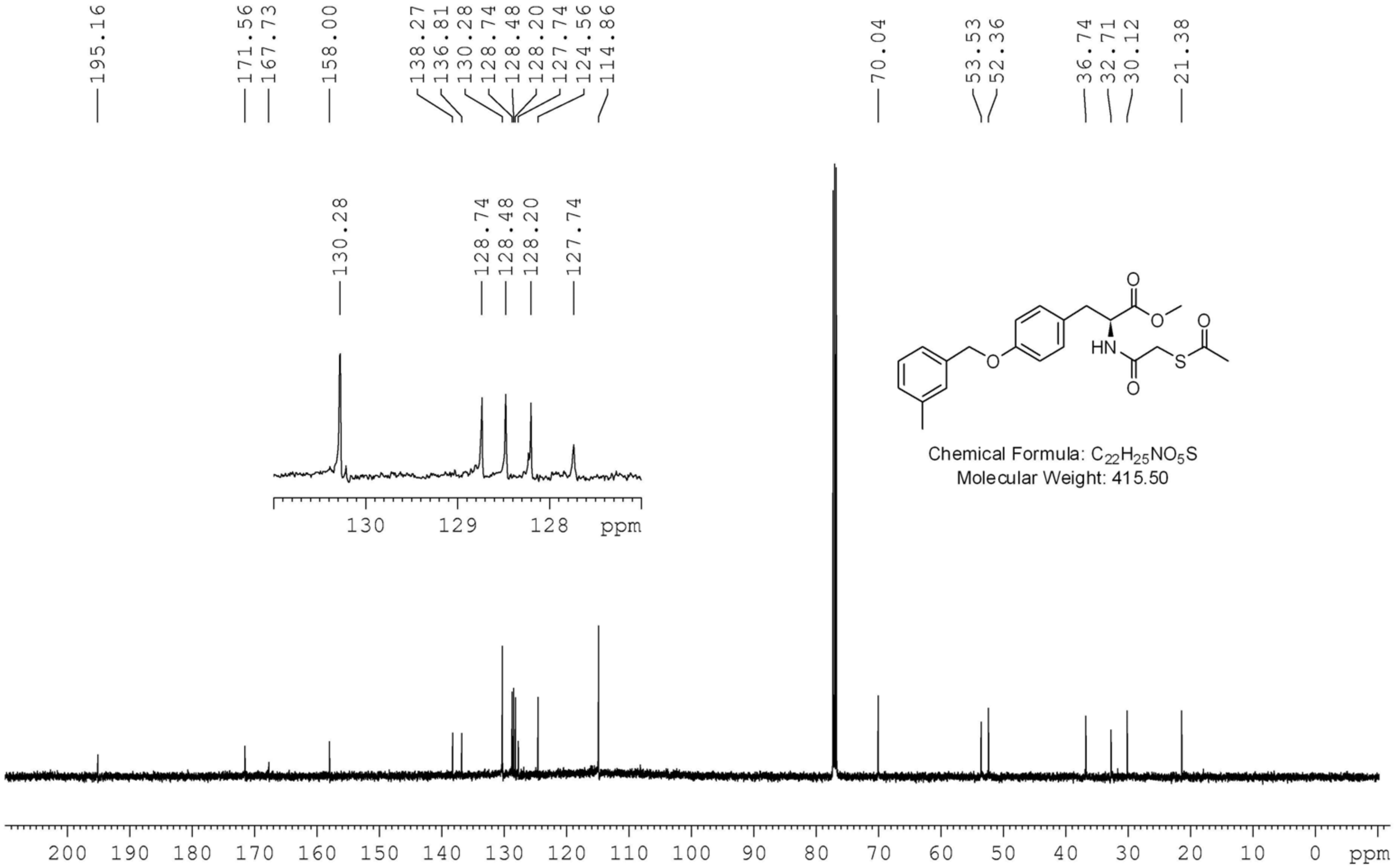

Chemical Formula: $C_{22}H_{25}NO_5S$
Molecular Weight: 415.50

Solvent: CD_3OD & NMR: 400 MHz

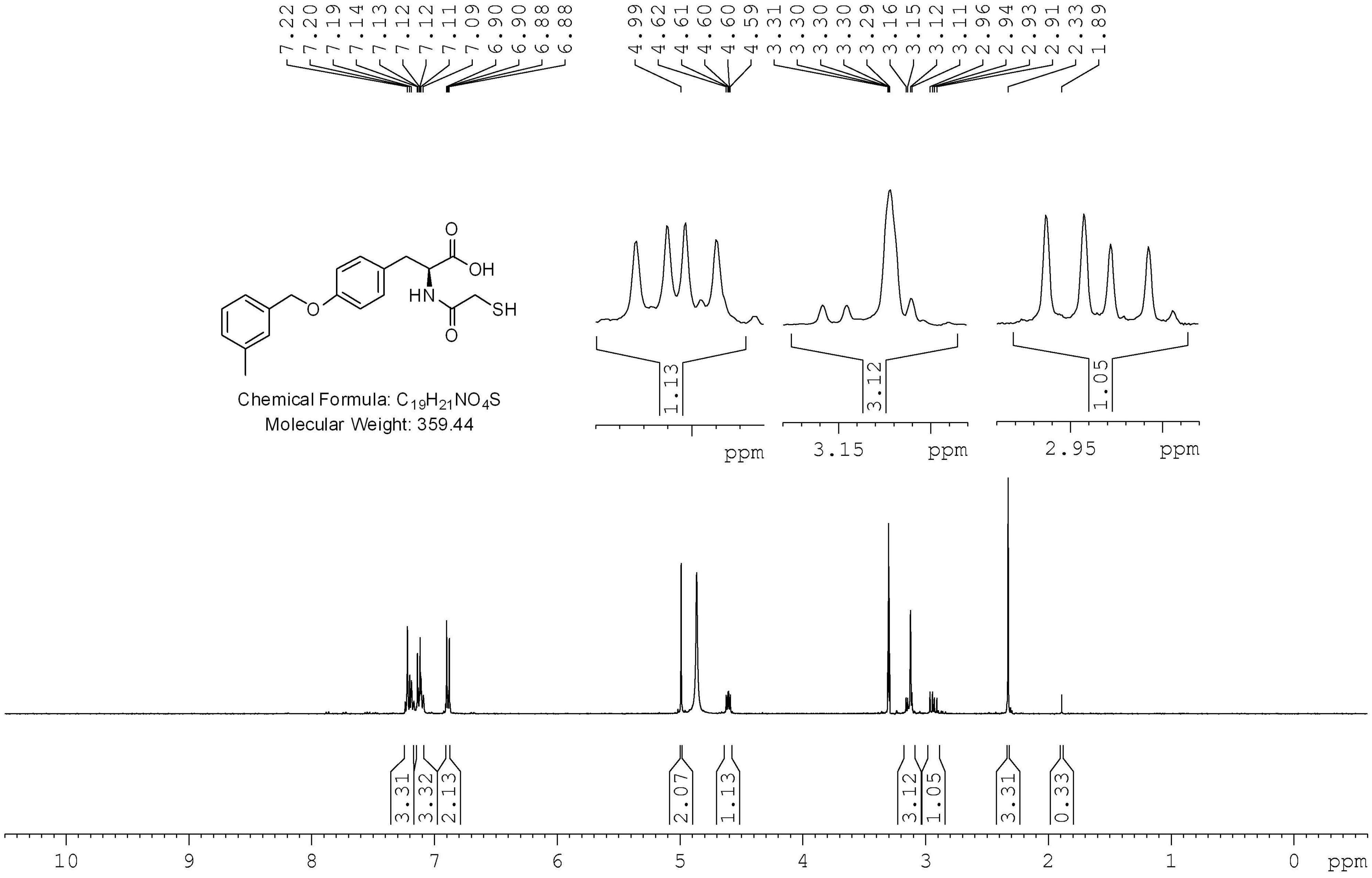

Solvent: CD_3OD & NMR: 100 MHz

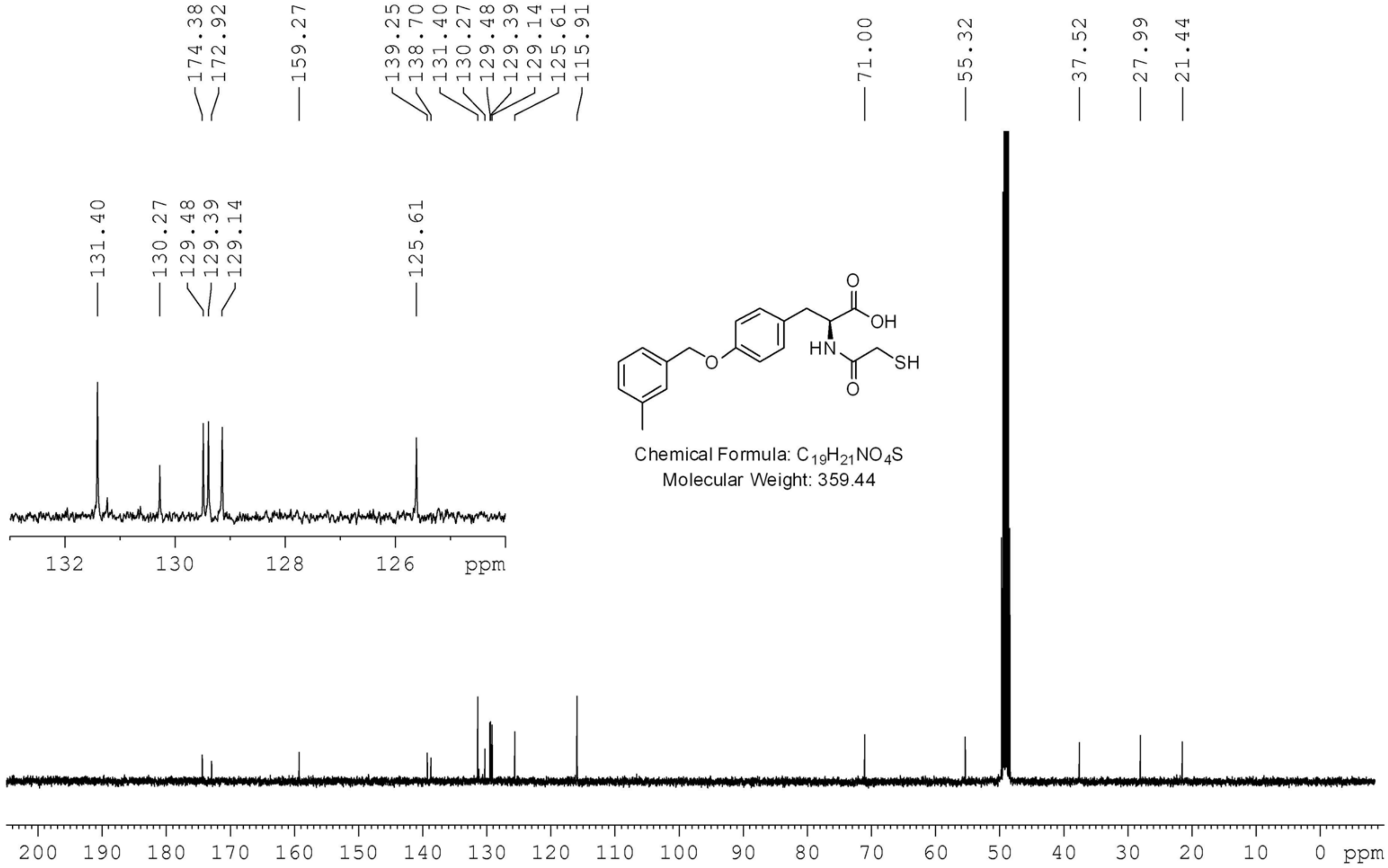

Solvent: $CDCl_3$ & NMR: 400 MHz

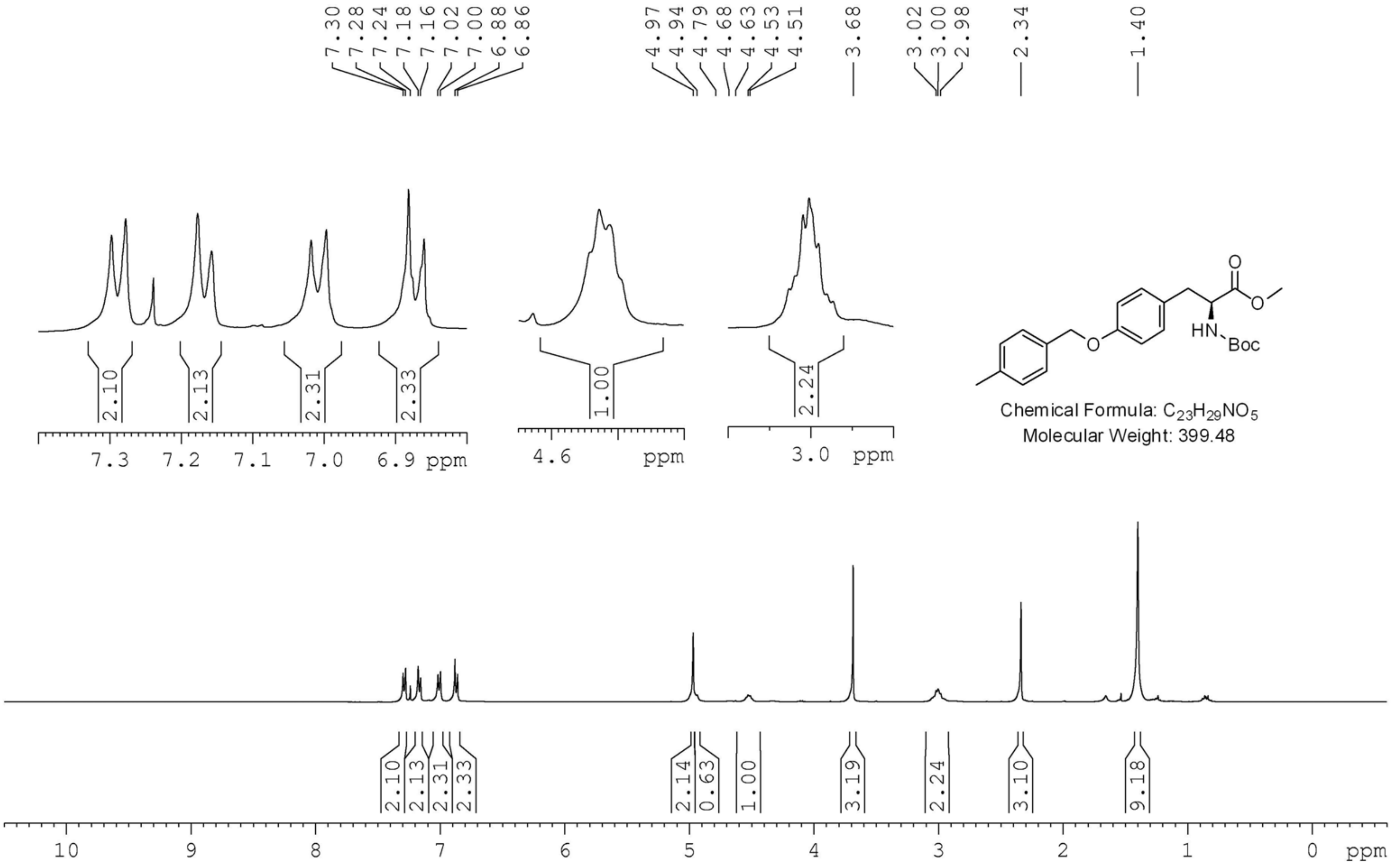

Chemical Formula: $C_{23}H_{29}NO_5$

Molecular Weight: 399.48

Solvent: $CDCl_3$ & NMR: 100 MHz

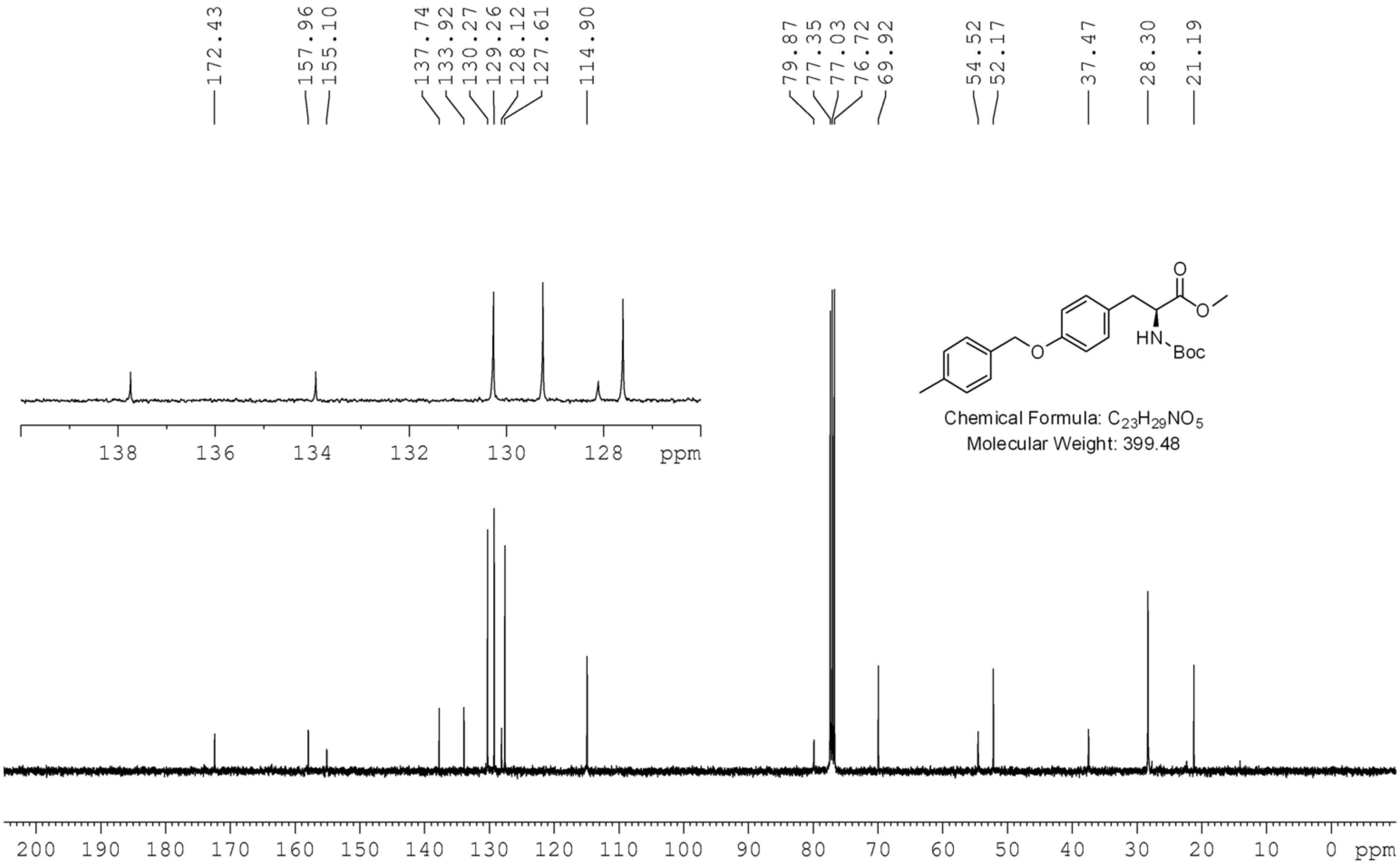

Chemical Formula: $C_{23}H_{29}NO_5$

Molecular Weight: 399.48

Solvent: $CDCl_3$ & NMR: 500 MHz

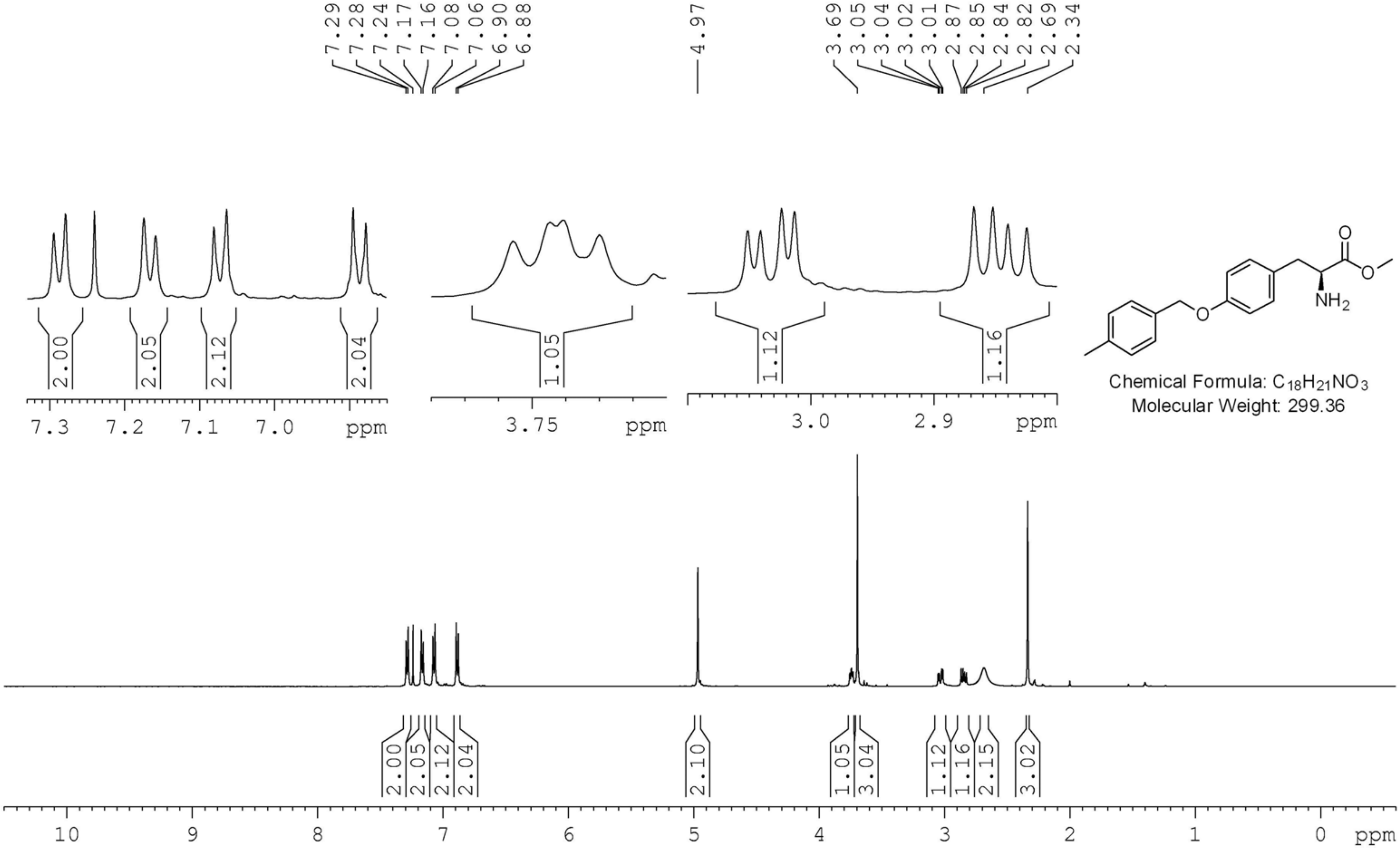

Chemical Formula: $C_{18}H_{21}NO_3$

Molecular Weight: 299.36

Solvent: $CDCl_3$ & NMR: 125 MHz

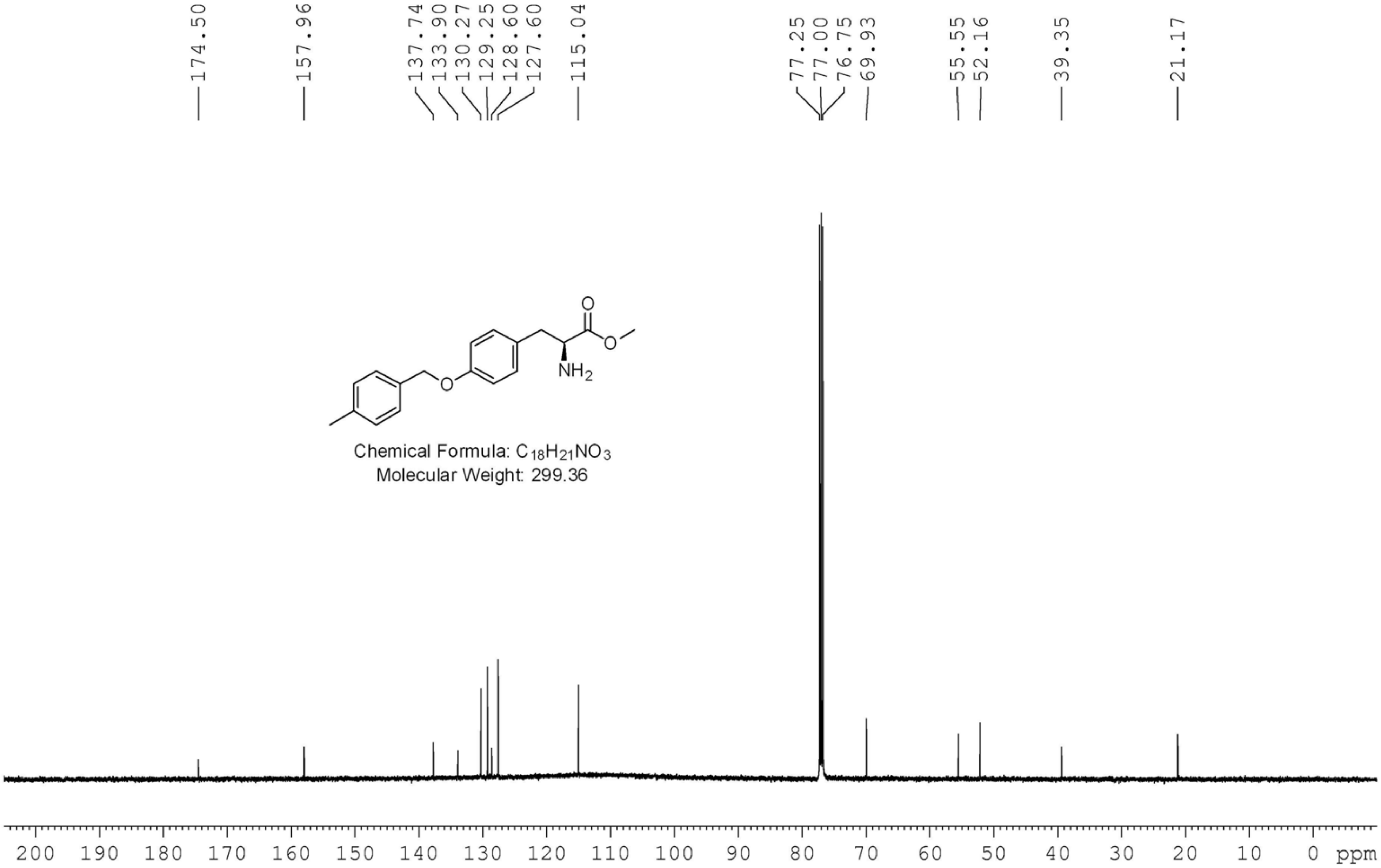

Solvent: $CDCl_3$ & NMR: 500 MHz

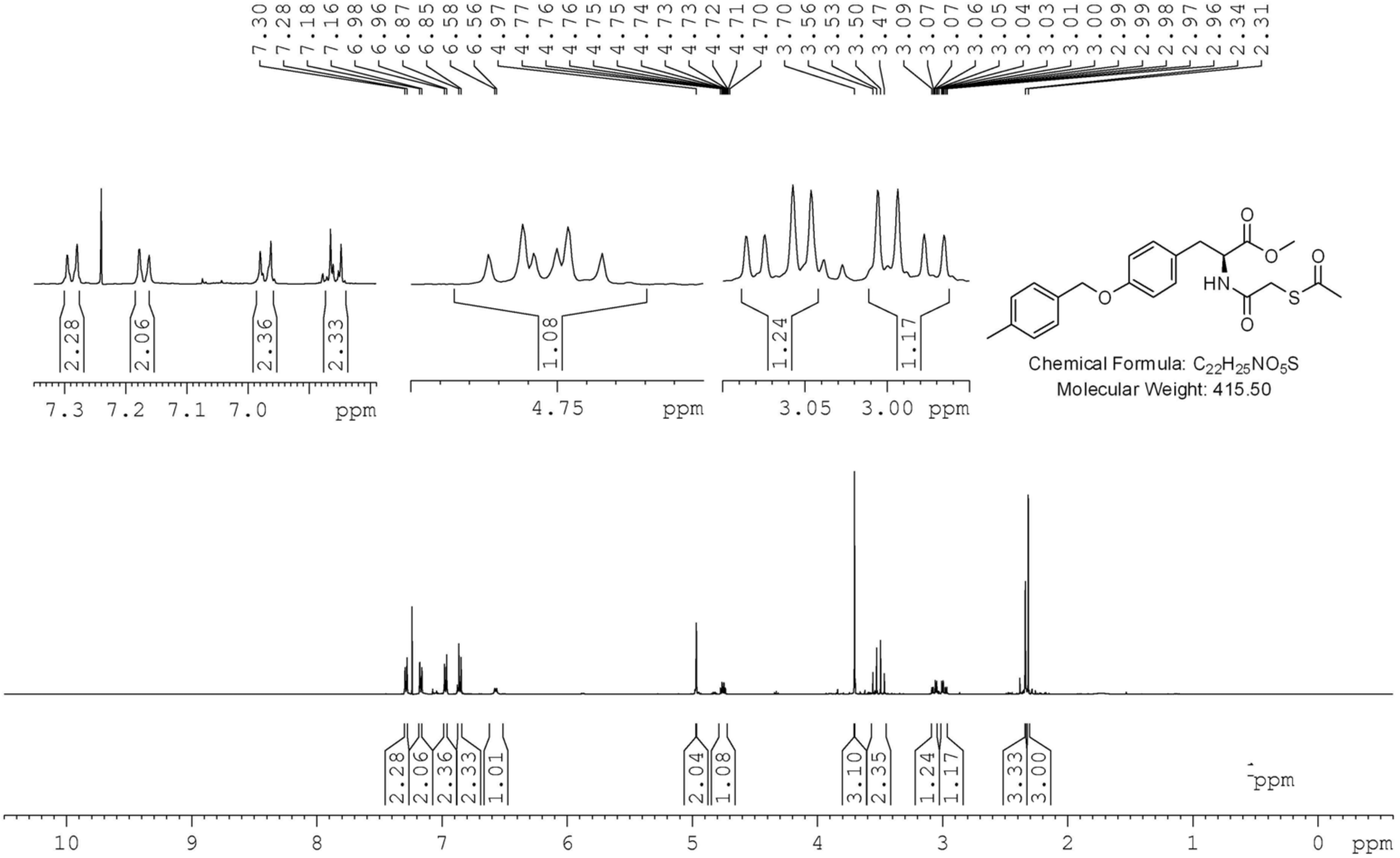

Solvent: $CDCl_3$ & NMR: 125 MHz

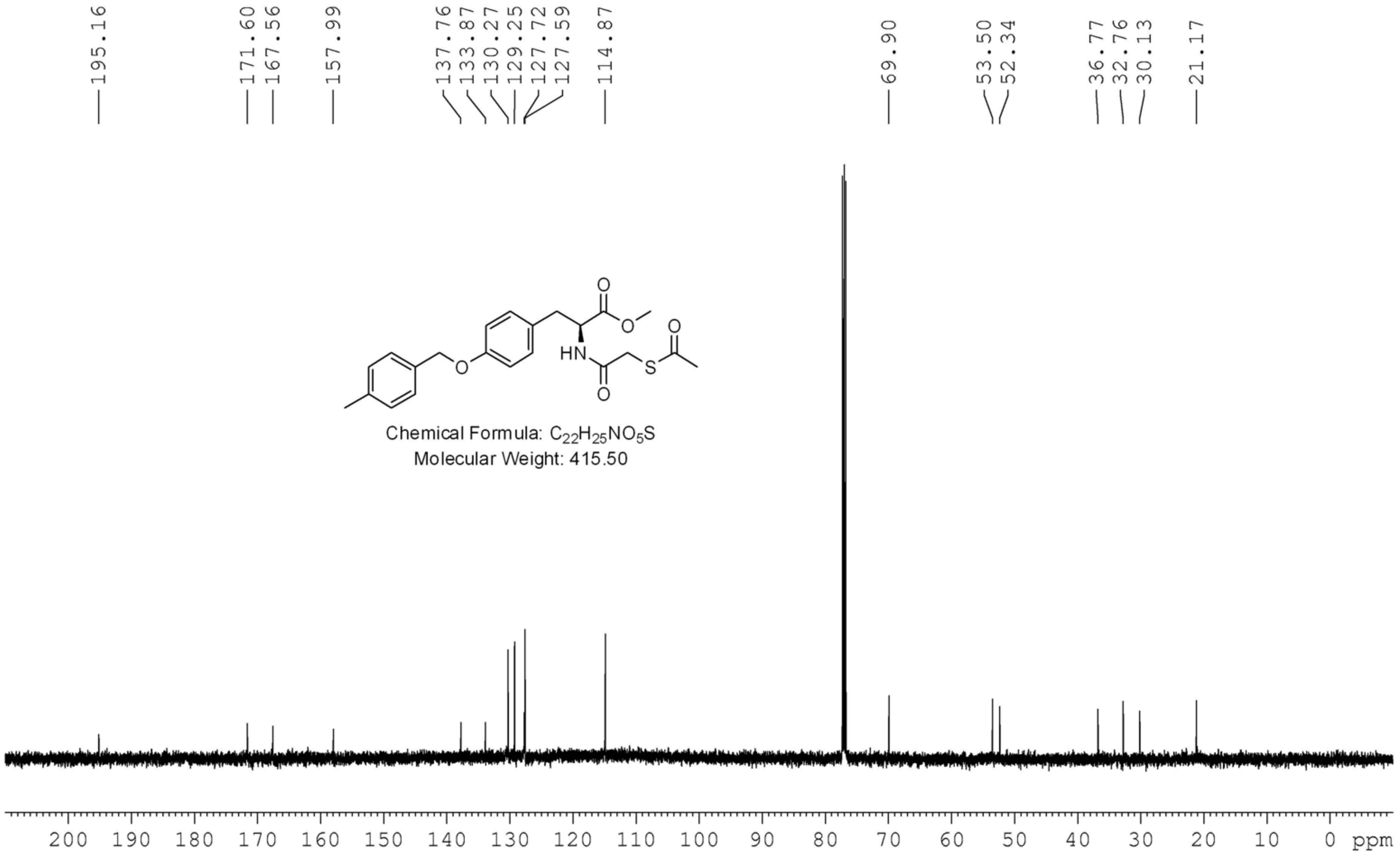

Solvent: CD_3OD & NMR: 500 MHz

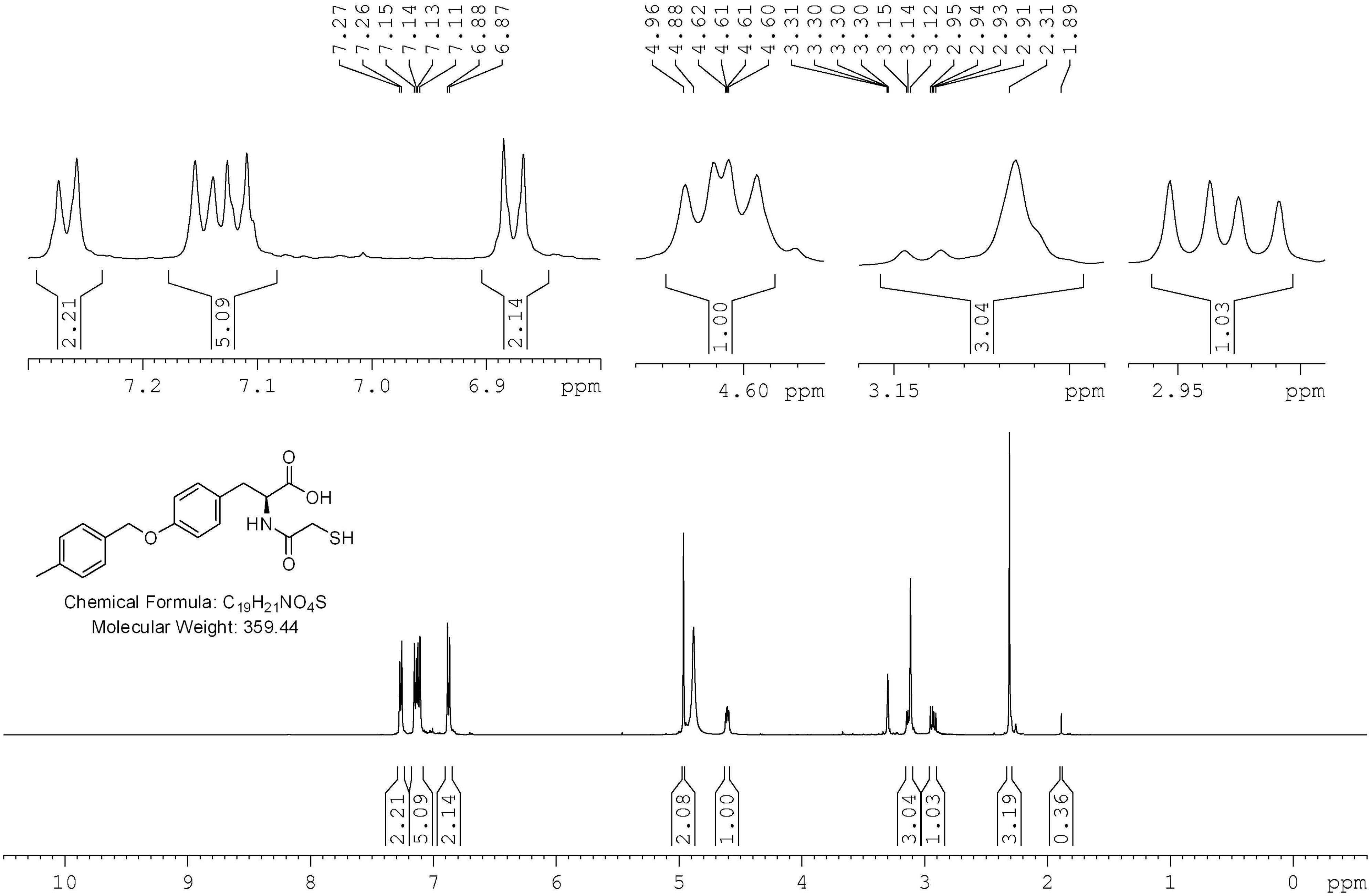

Chemical Formula: $C_{19}H_{21}NO_4S$

Molecular Weight: 359.44

Solvent: CD_3OD & NMR: 125 MHz

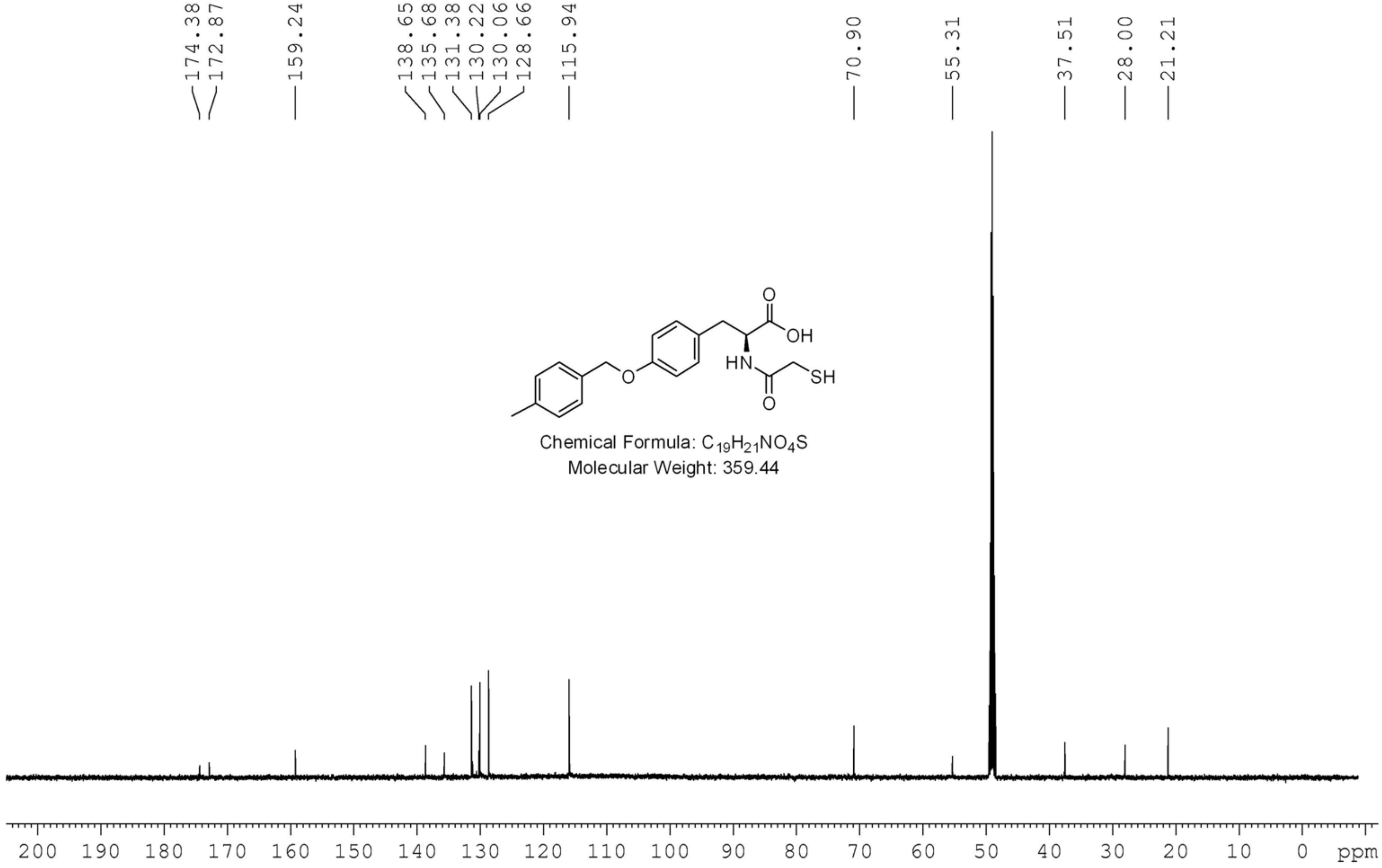

Solvent: $CDCl_3$ & NMR: 500 MHz

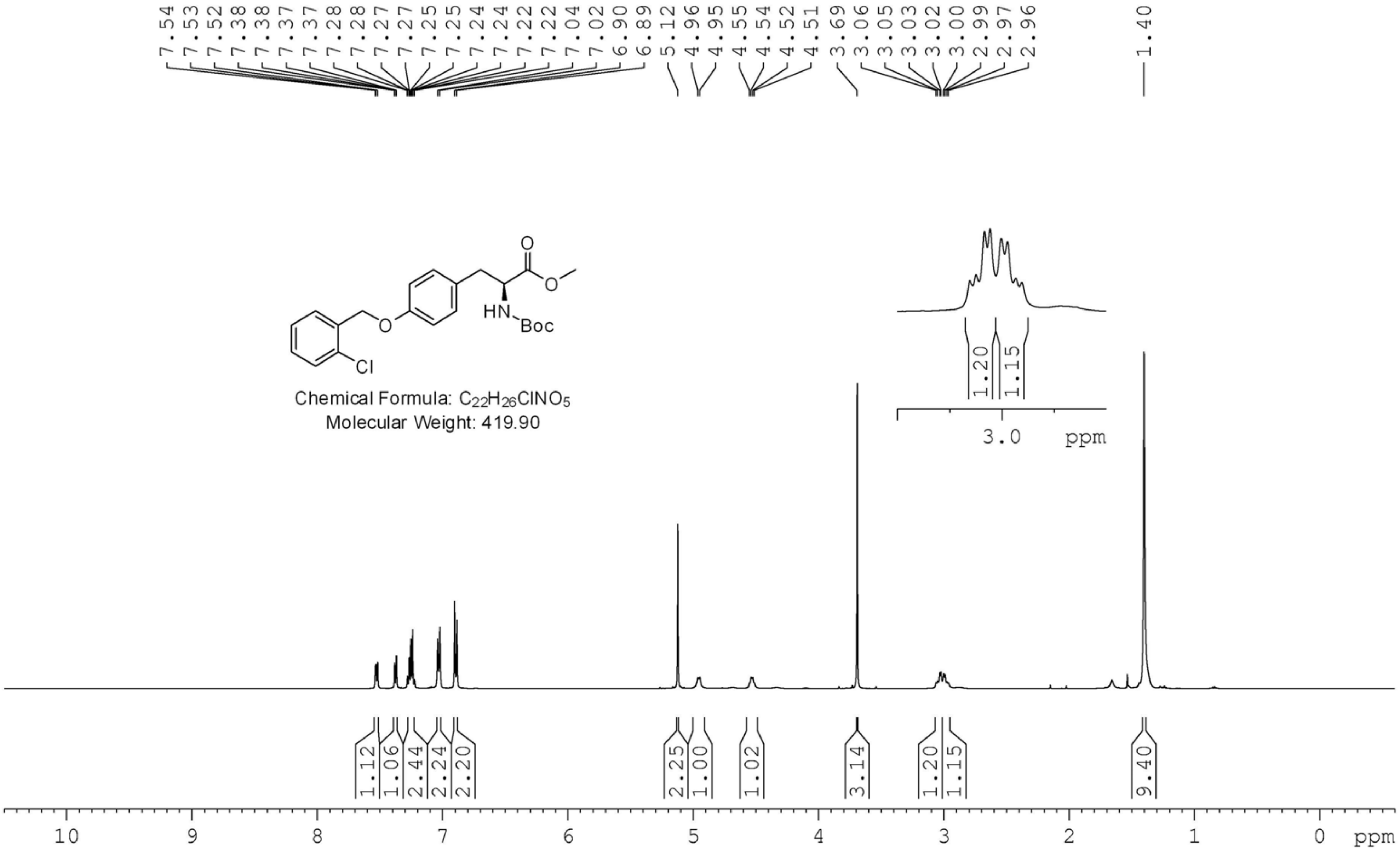

Chemical Formula: $C_{22}H_{26}ClNO_5$
Molecular Weight: 419.90

Solvent: $CDCl_3$ & NMR: 125 MHz

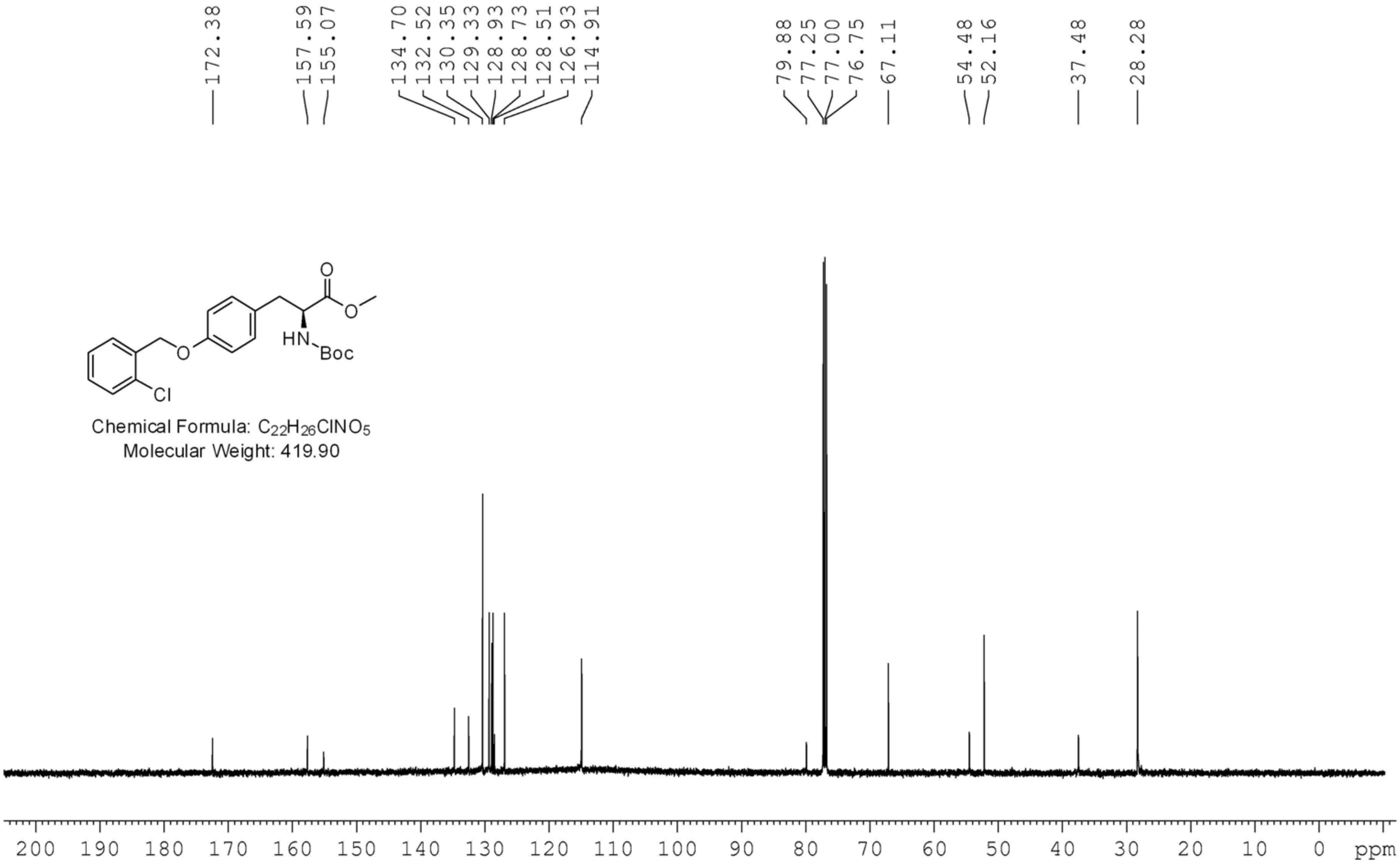

Solvent: $CDCl_3$ & NMR: 500 MHz

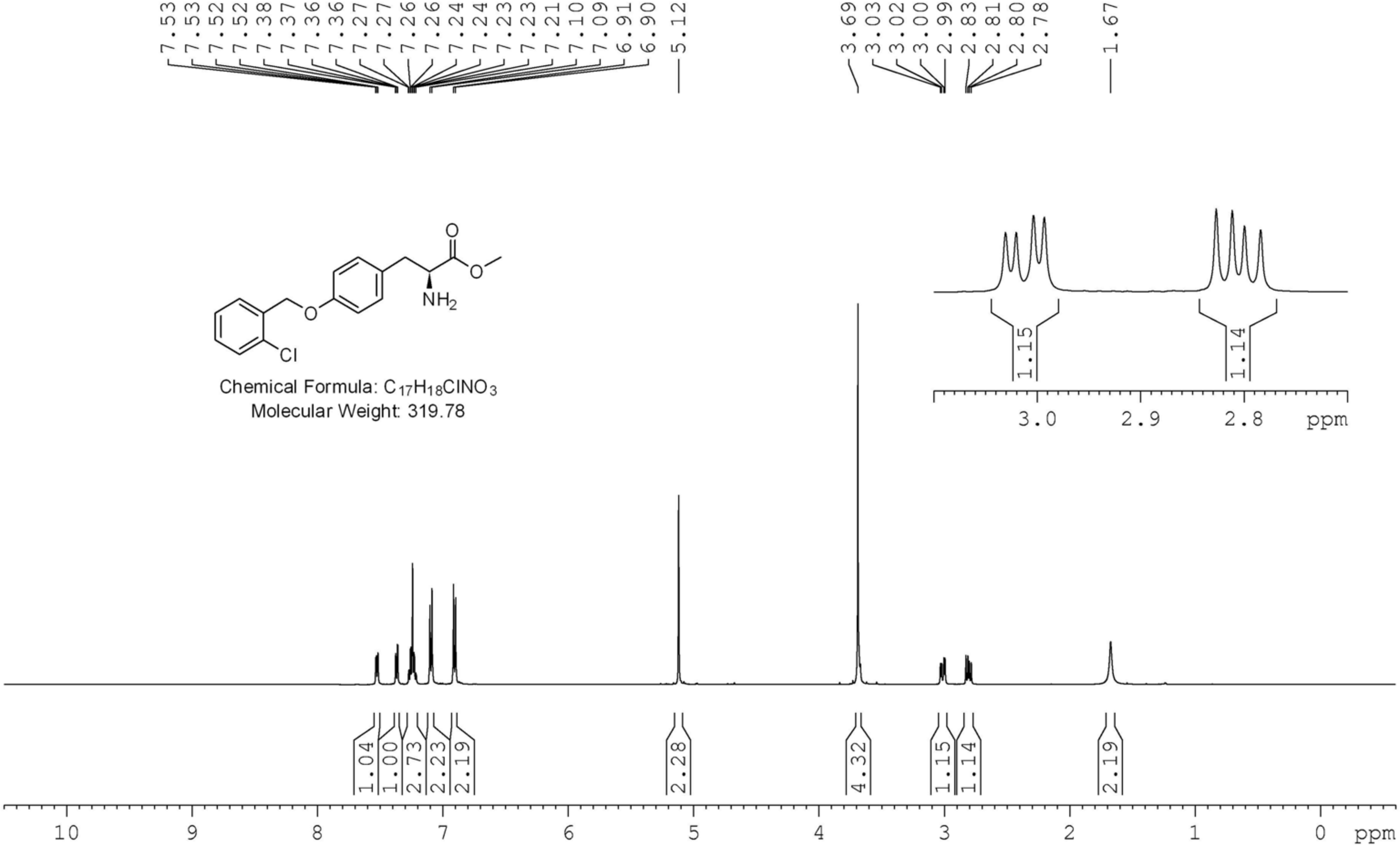

Solvent: $CDCl_3$ & NMR: 125 MHz

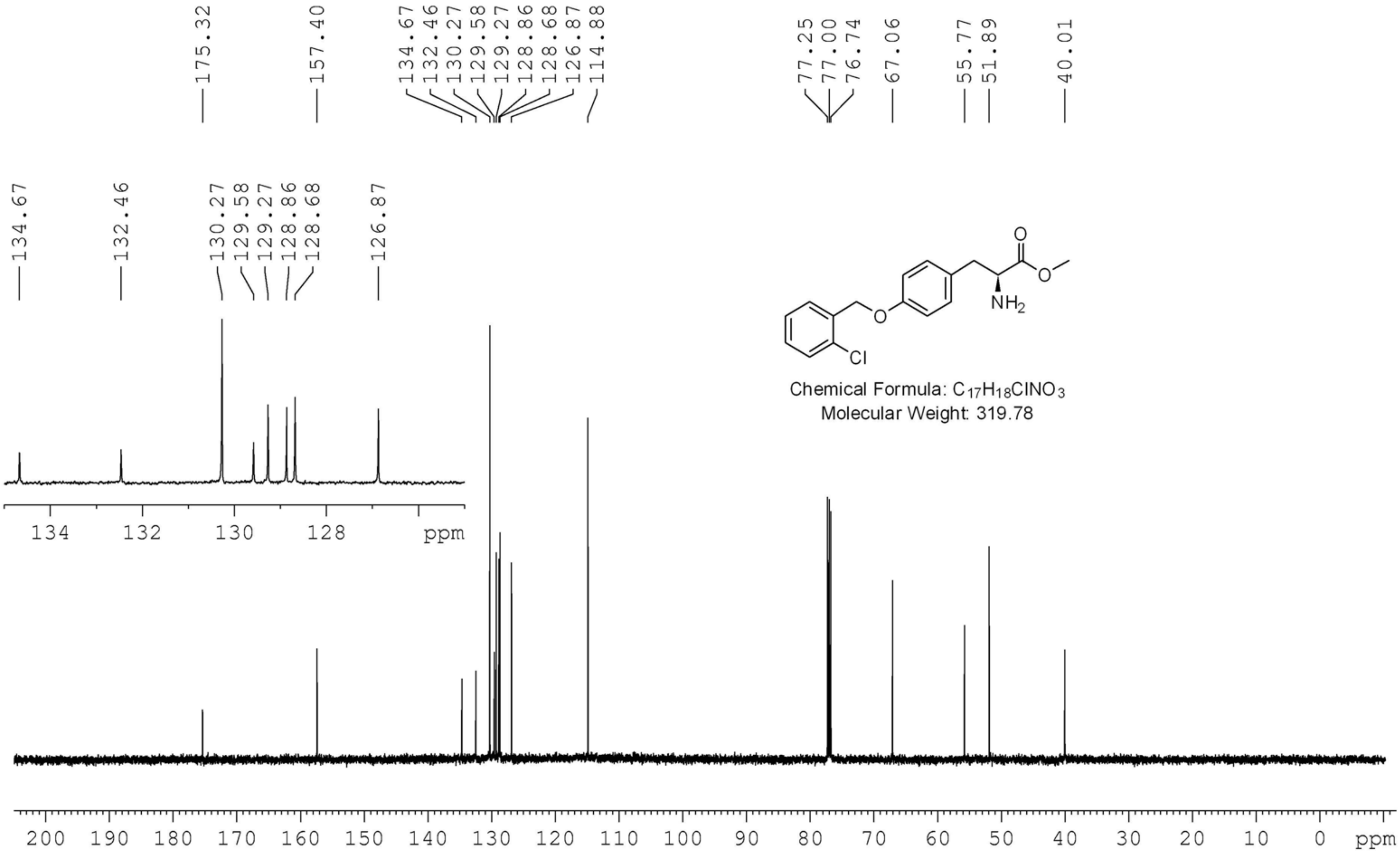

Chemical Formula: $C_{17}H_{18}ClNO_3$
Molecular Weight: 319.78

Solvent: $CDCl_3$ & NMR: 500 MHz

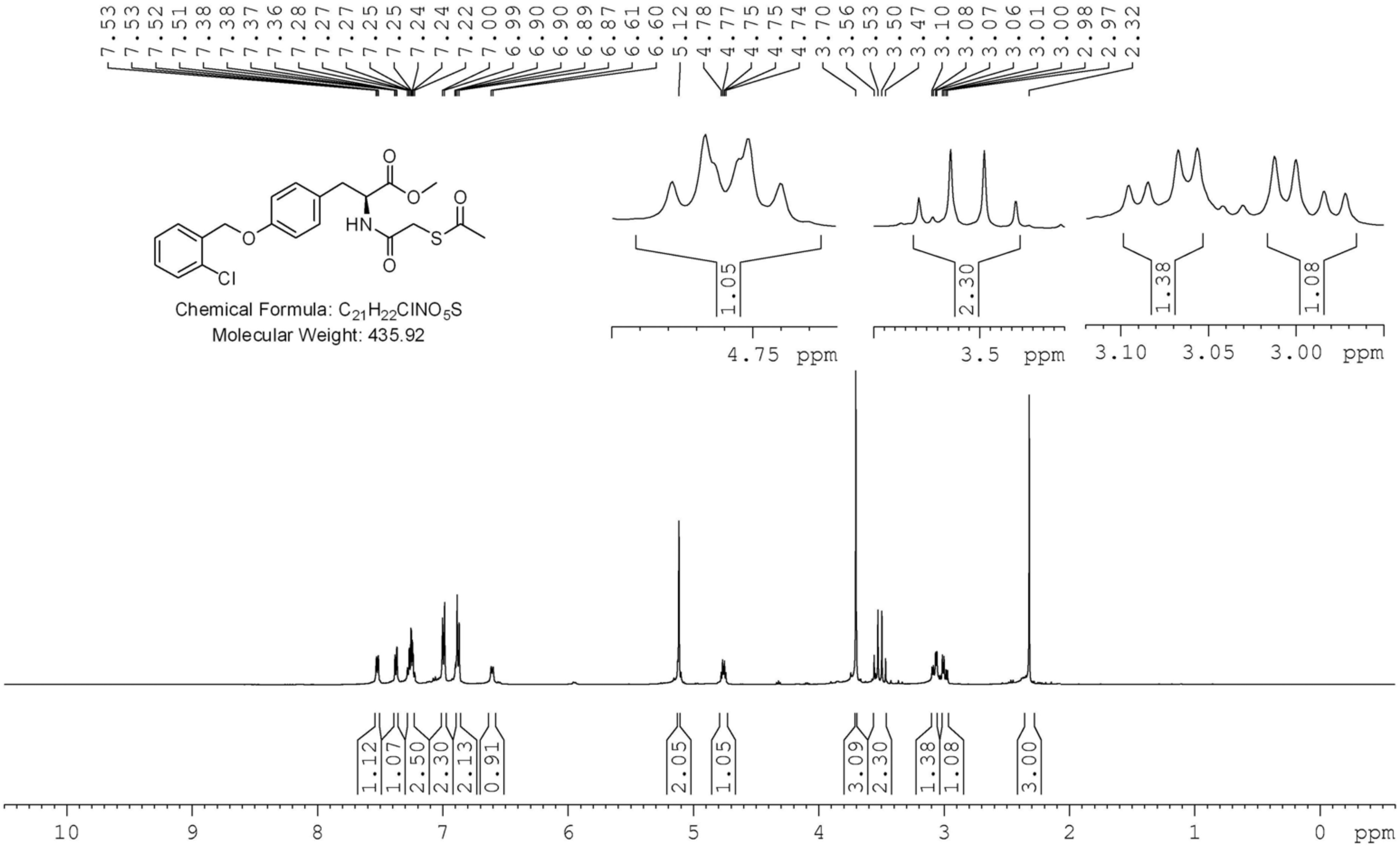

Solvent: $CDCl_3$ & NMR: 125 MHz

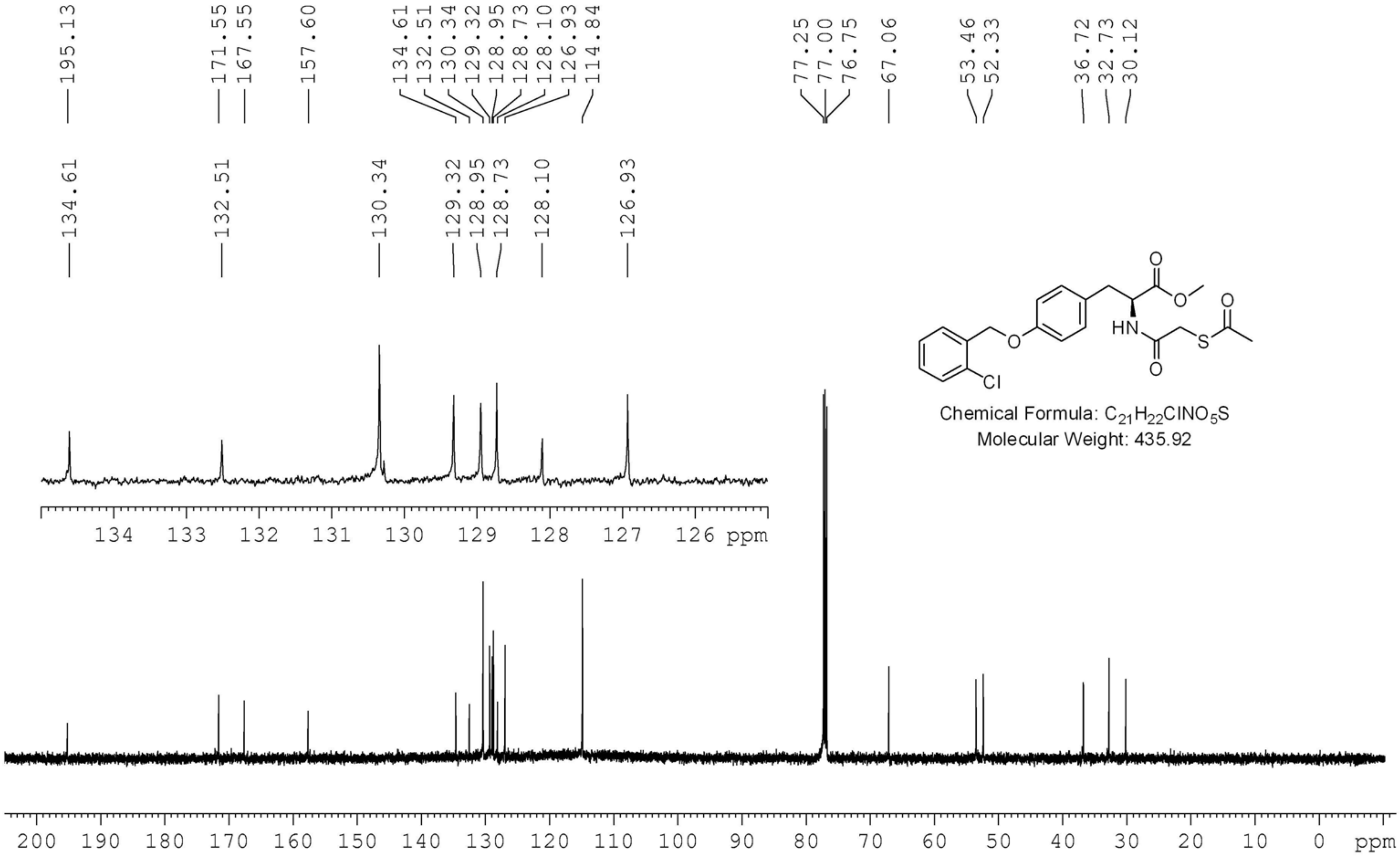

Chemical Formula: $C_{21}H_{22}ClNO_5S$

Molecular Weight: 435.92

Solvent: CD_3OD & NMR: 500 MHz

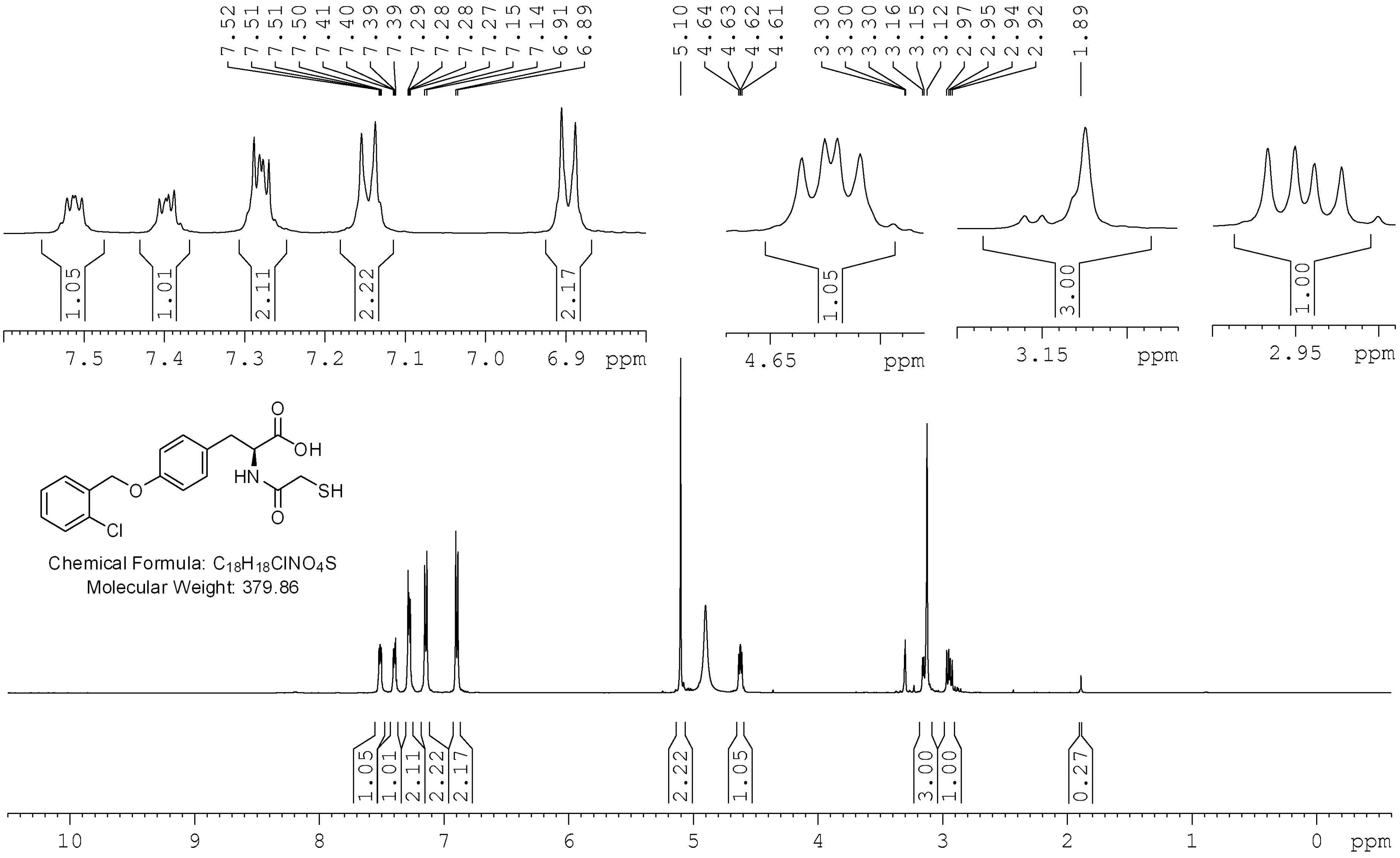

Solvent: CD_3OD & NMR: 125 MHz

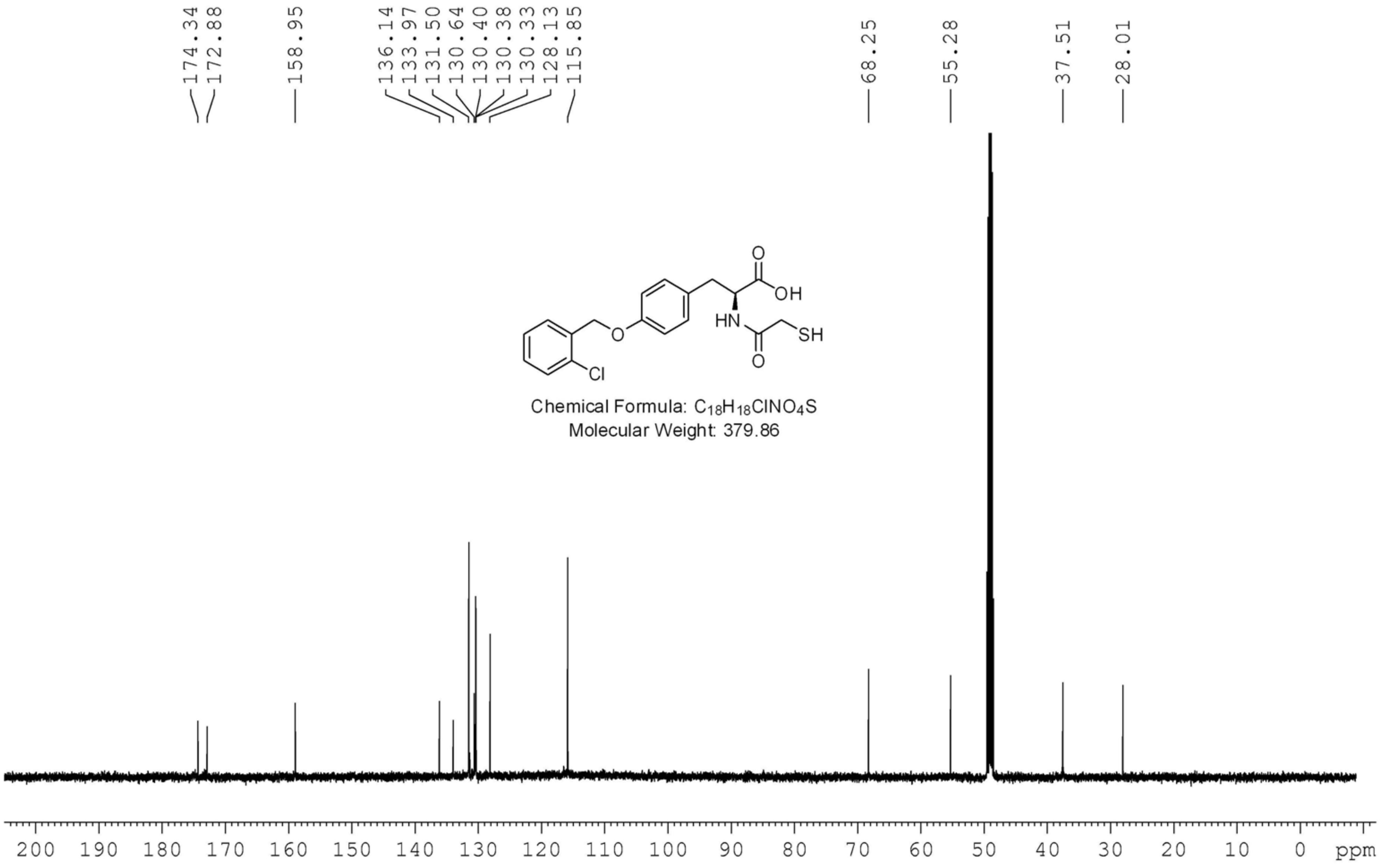

Solvent: $CDCl_3$ & NMR: 500 MHz

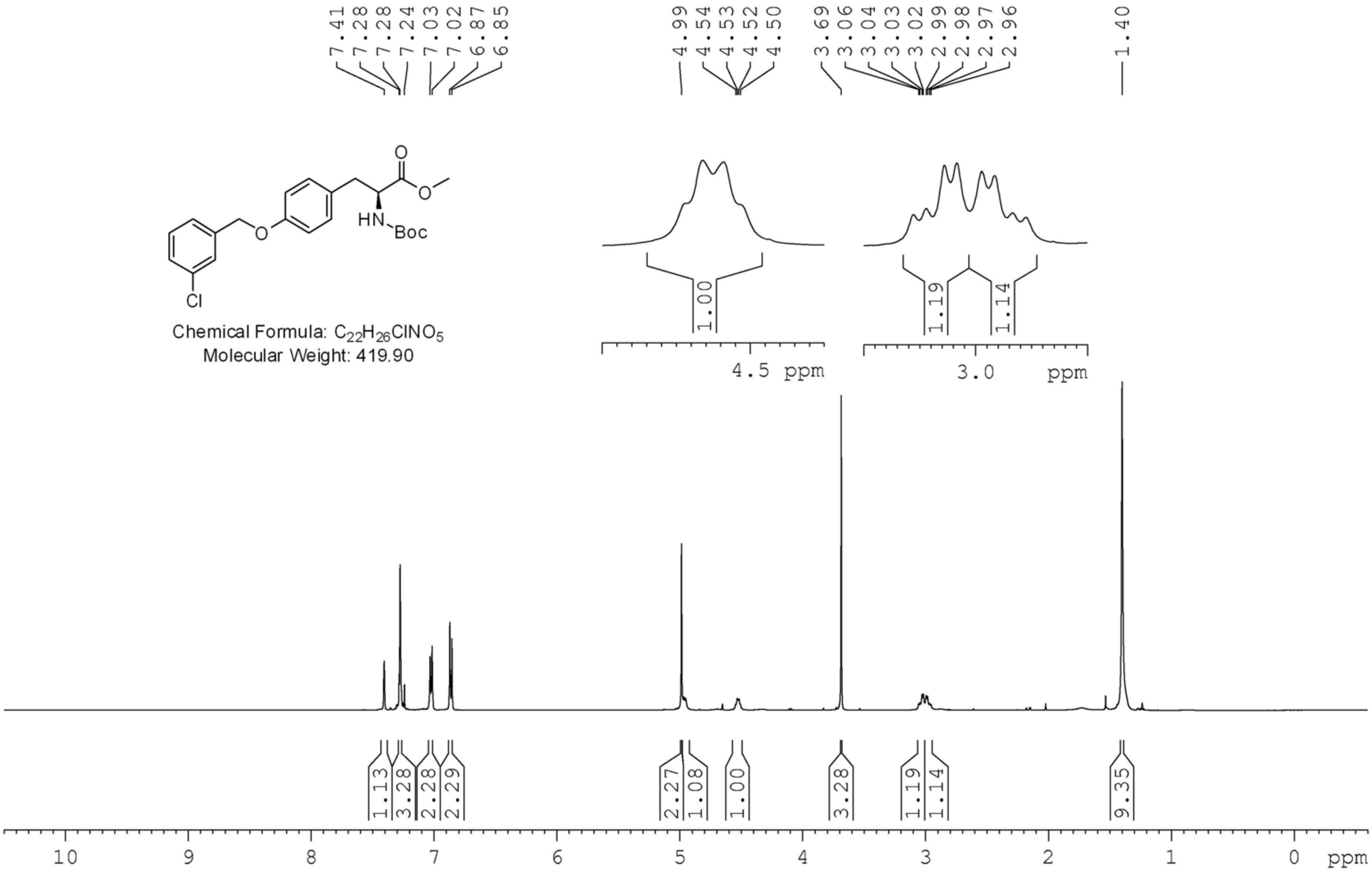

Solvent: $CDCl_3$ & NMR: 125 MHz

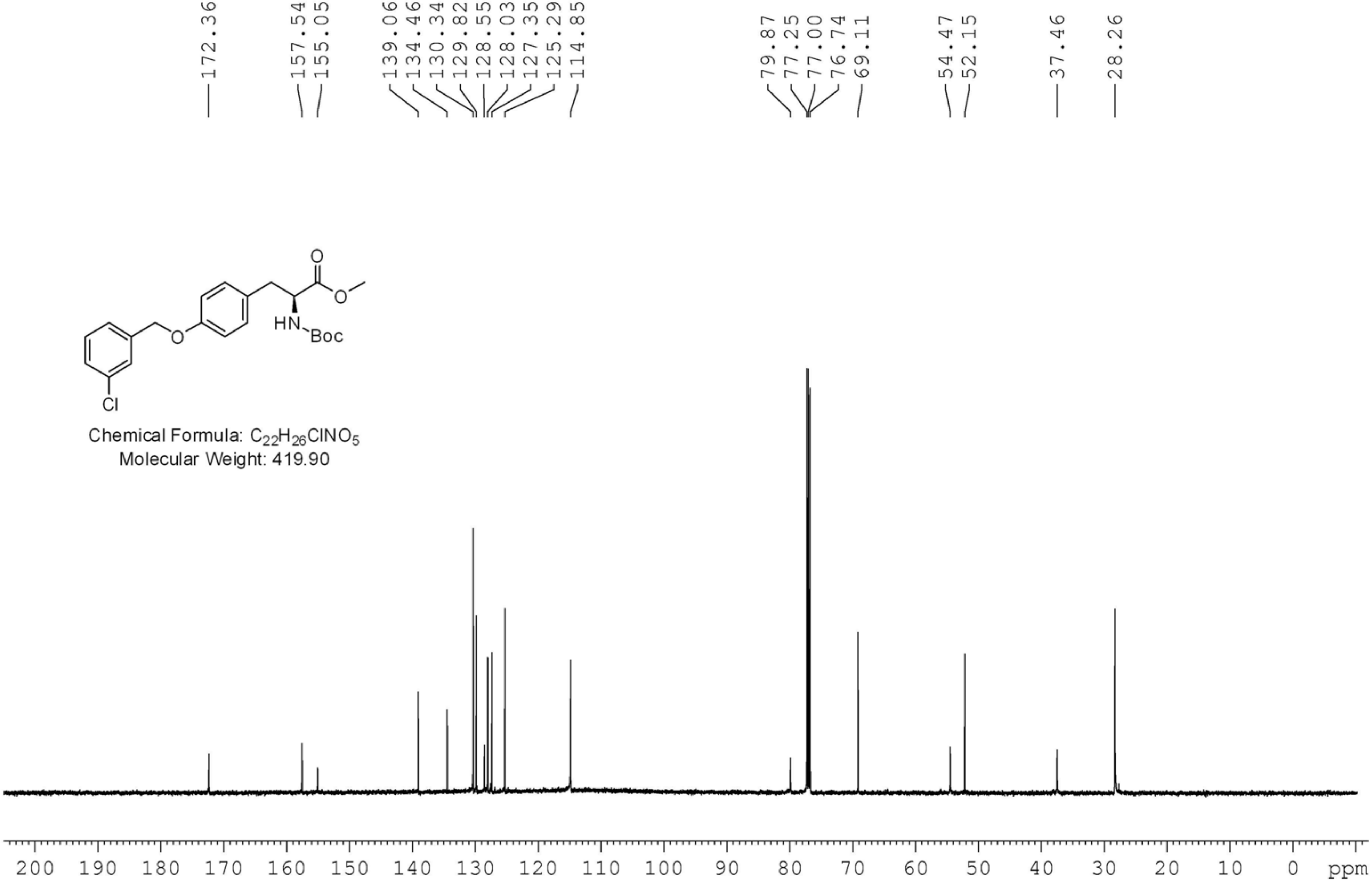

Solvent: $CDCl_3$ & NMR: 500 MHz

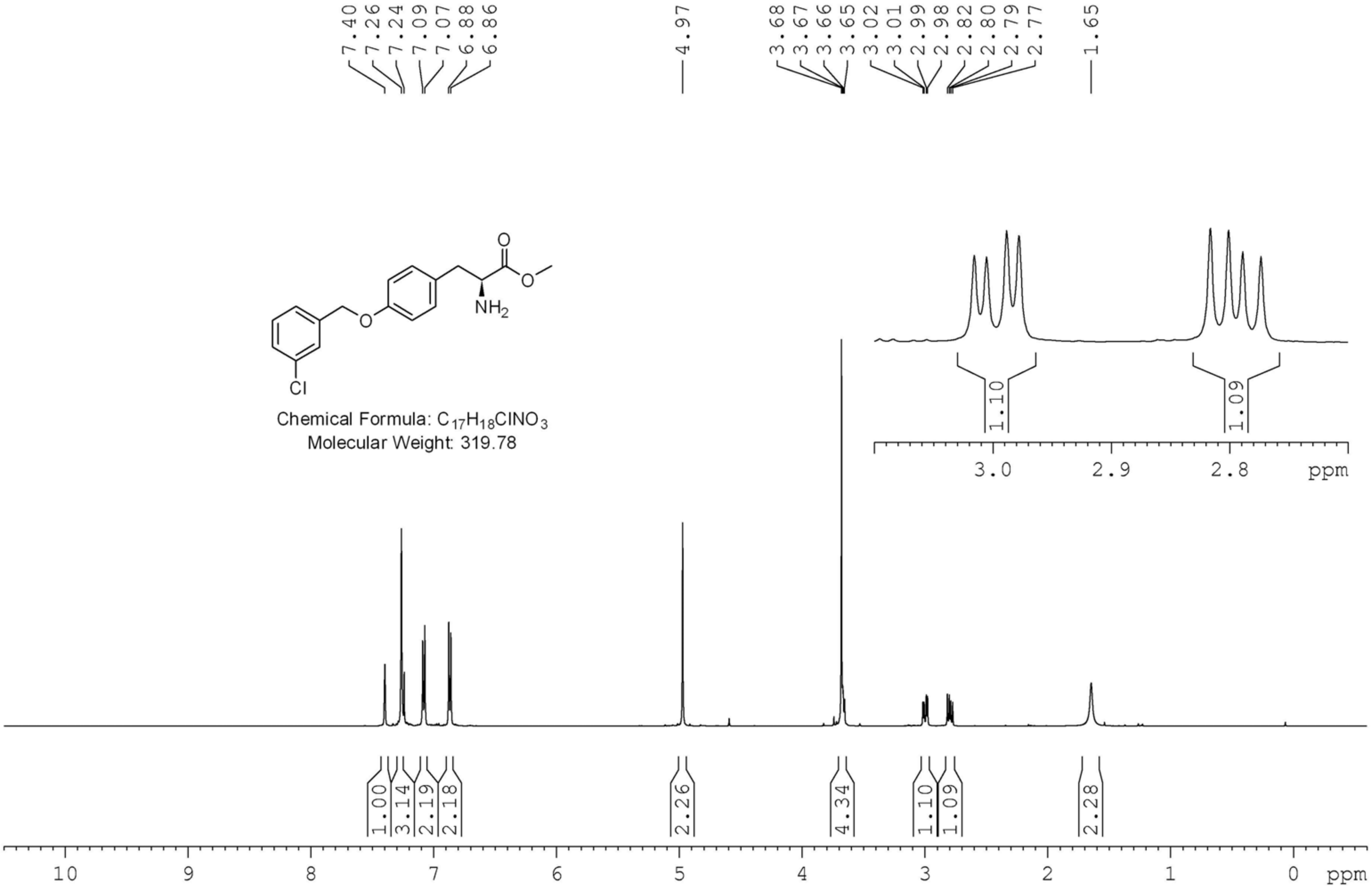

Solvent: $CDCl_3$ & NMR: 125 MHz

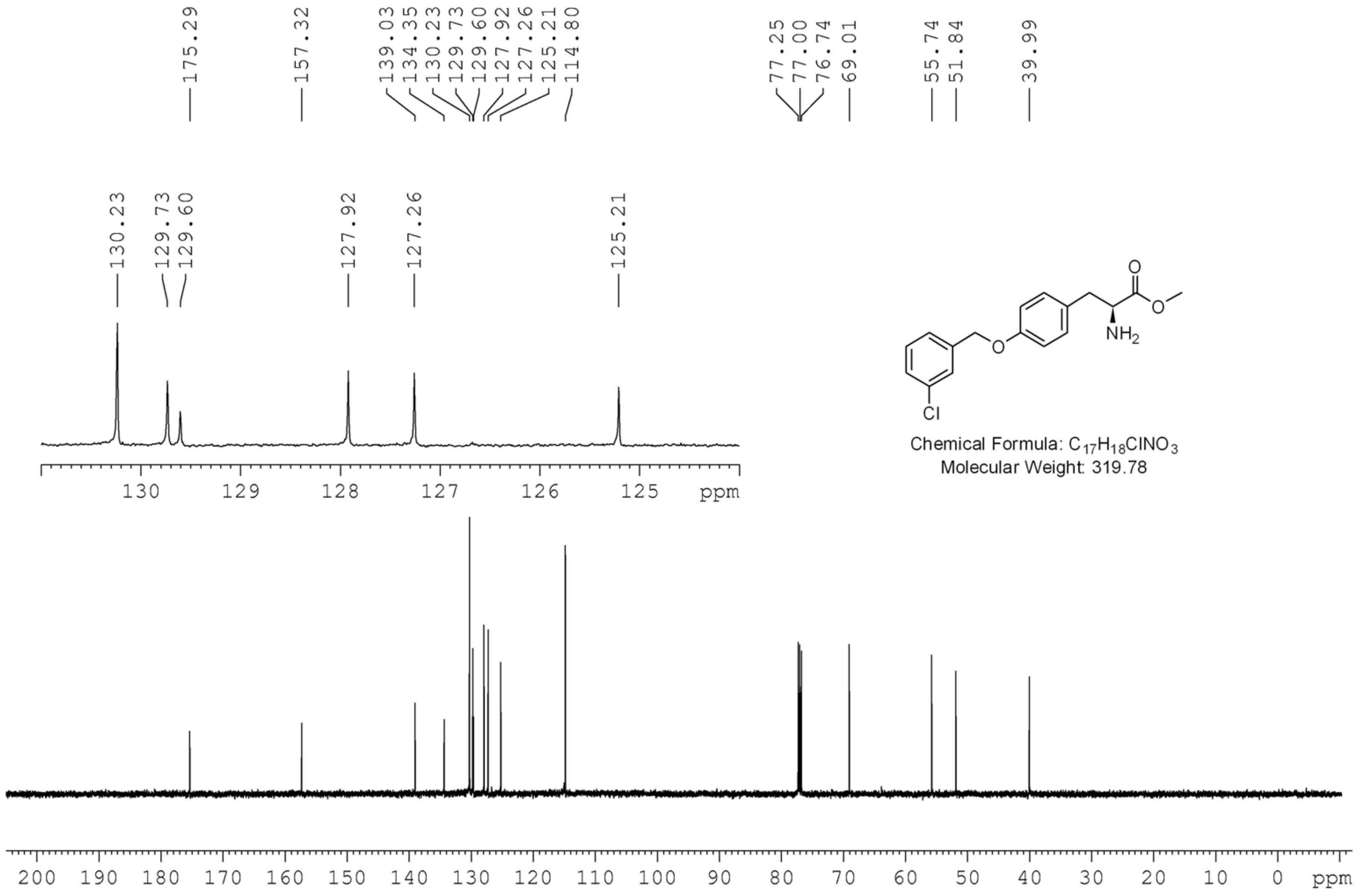

Chemical Formula: $C_{17}H_{18}ClNO_3$
Molecular Weight: 319.78

Solvent: $CDCl_3$ & NMR: 500 MHz

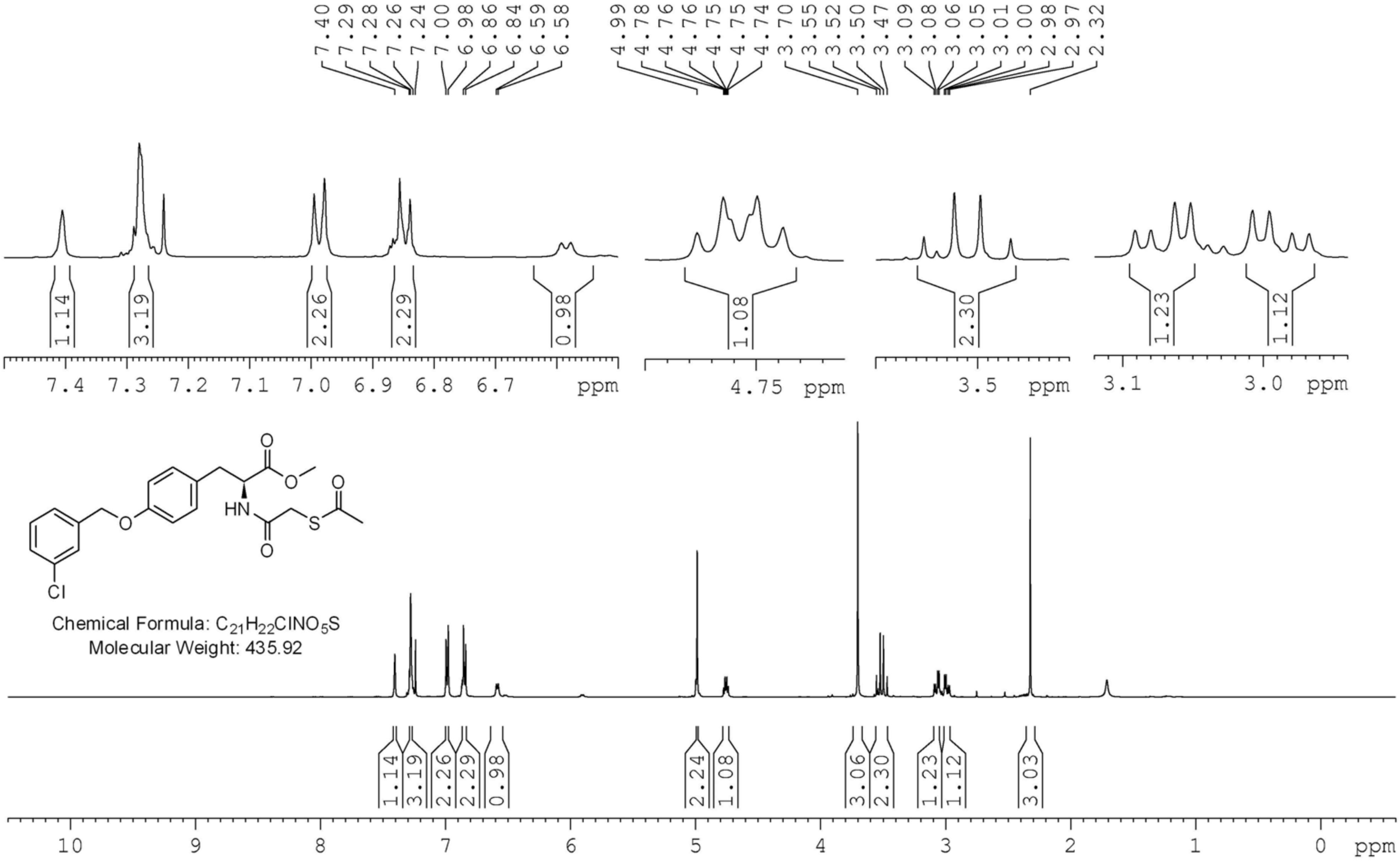

Chemical Formula: $C_{21}H_{22}ClNO_5S$
Molecular Weight: 435.92

Solvent: $CDCl_3$ & NMR: 125 MHz

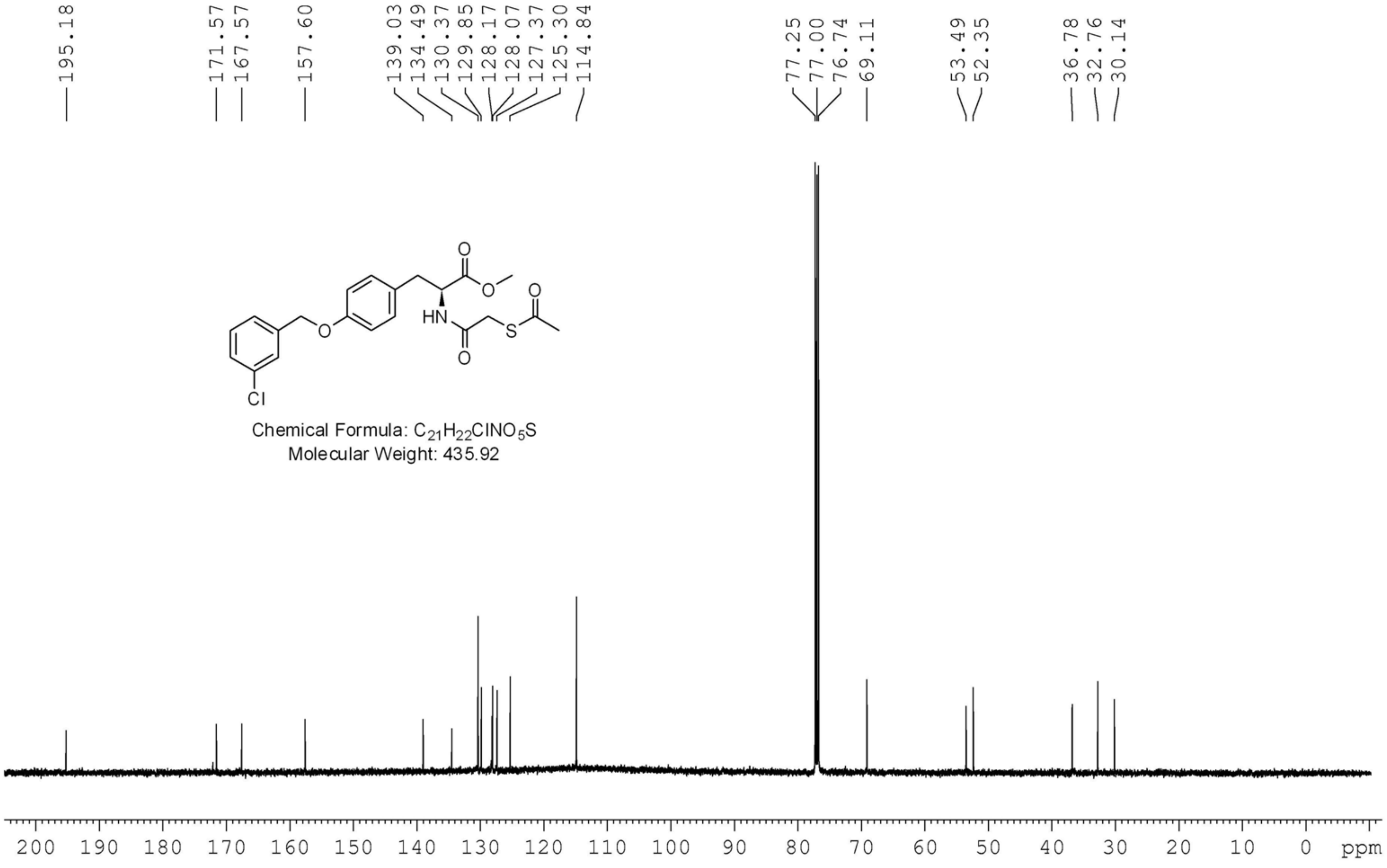

Solvent: CD_3OD & NMR: 500 MHz

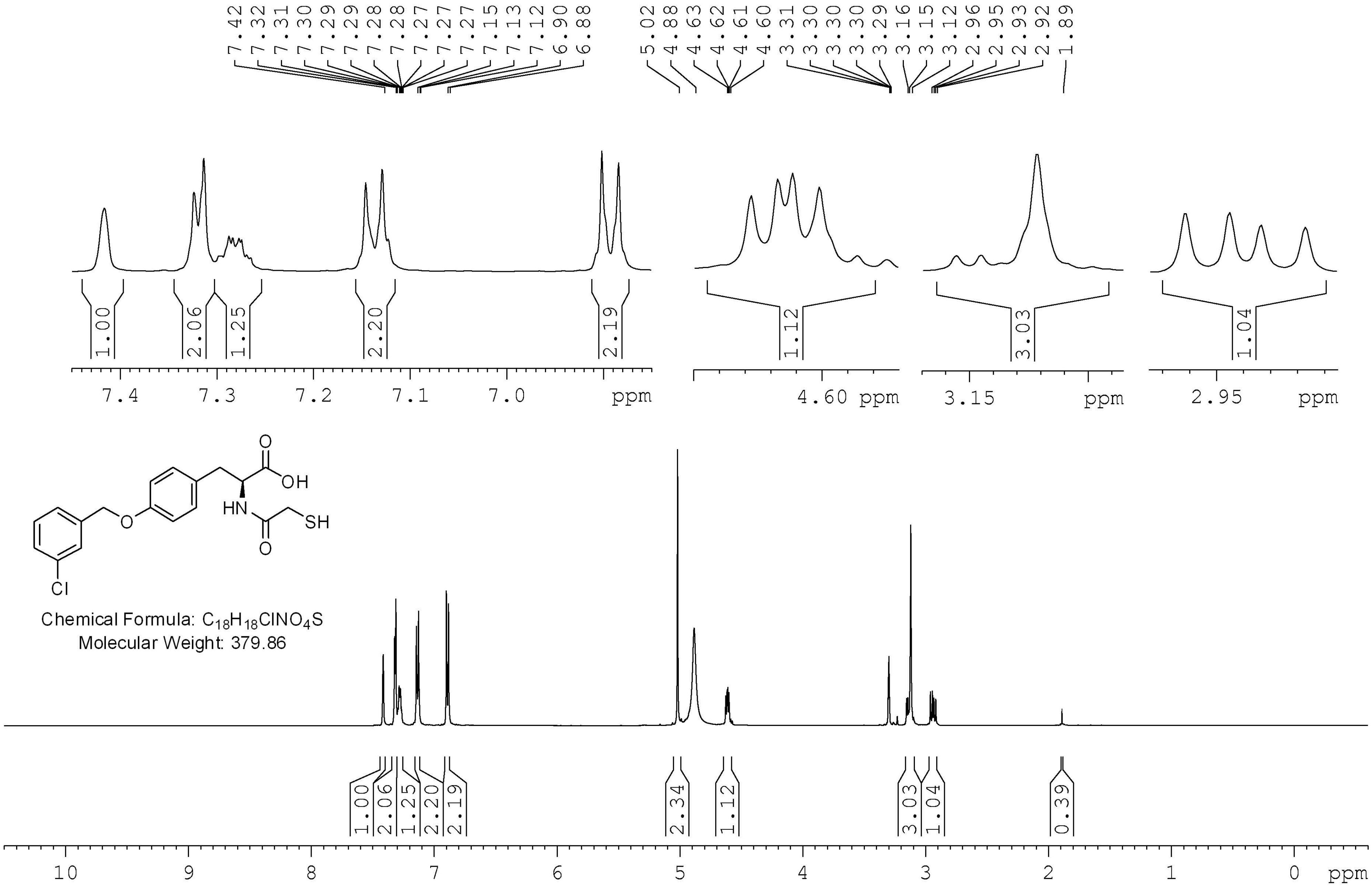

Solvent: CD_3OD & NMR: 125 MHz

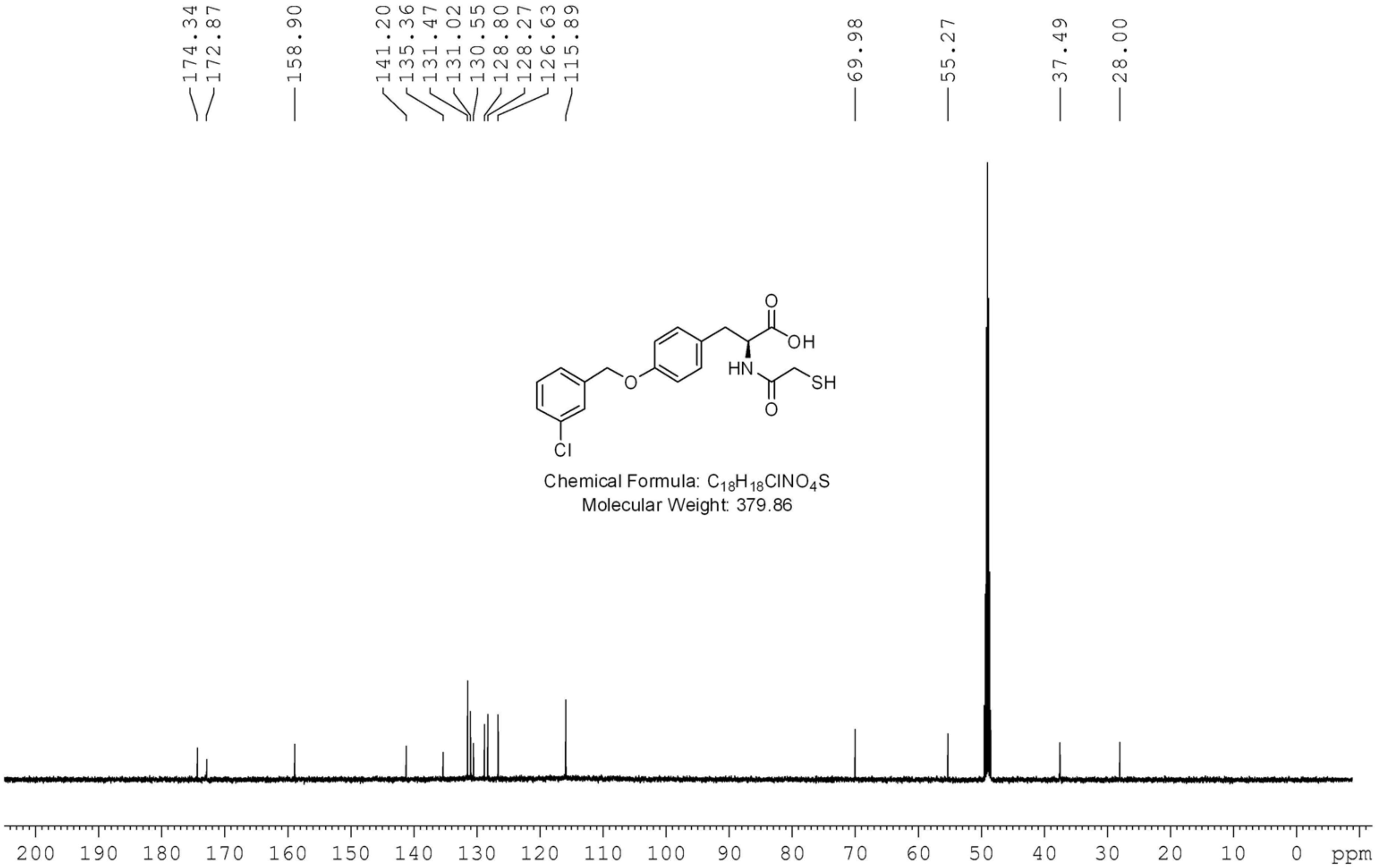

Solvent: $CDCl_3$ & NMR: 400 MHz

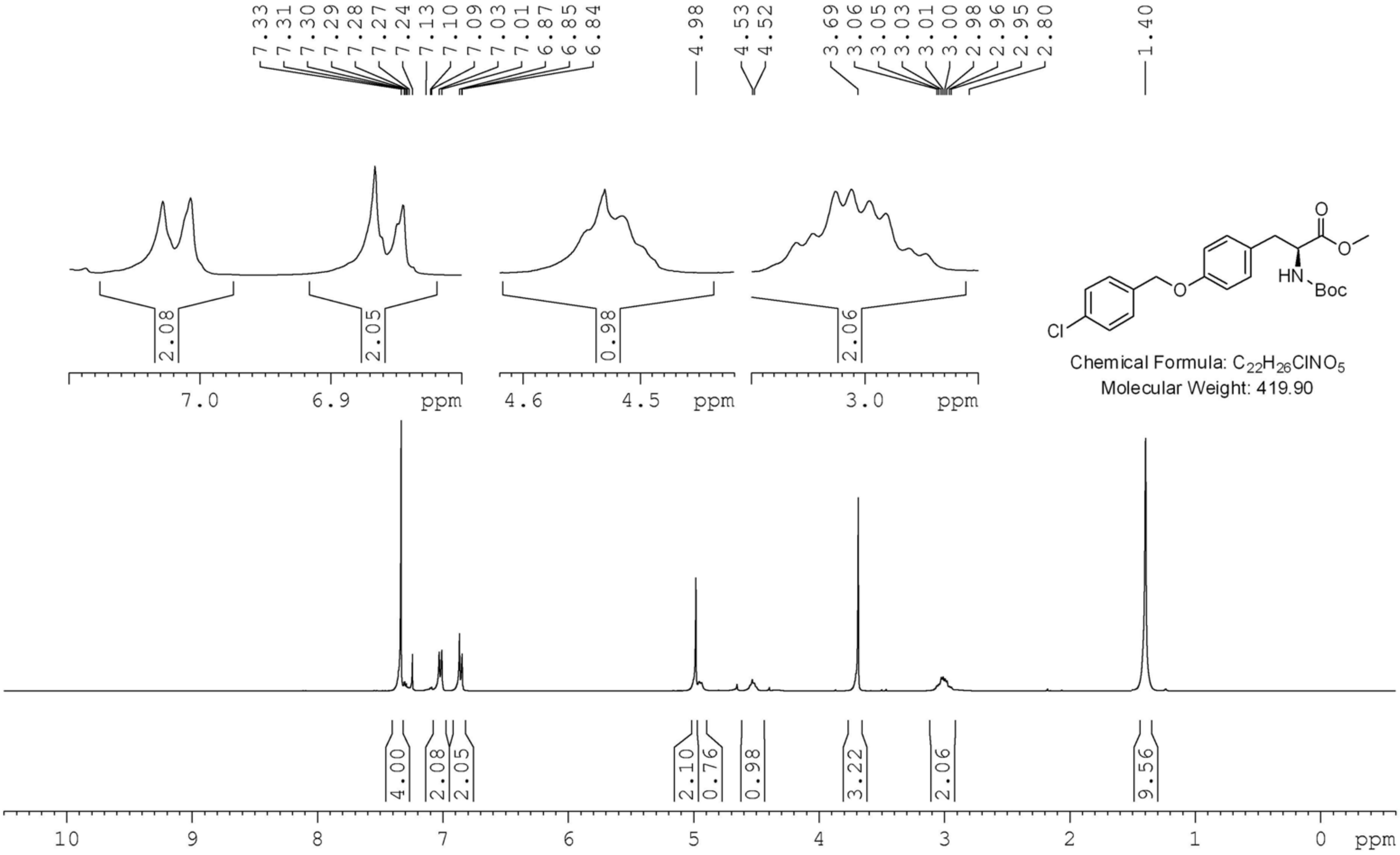

Chemical Formula: $C_{22}H_{26}ClNO_5$

Molecular Weight: 419.90

Solvent: $CDCl_3$ & NMR: 100 MHz

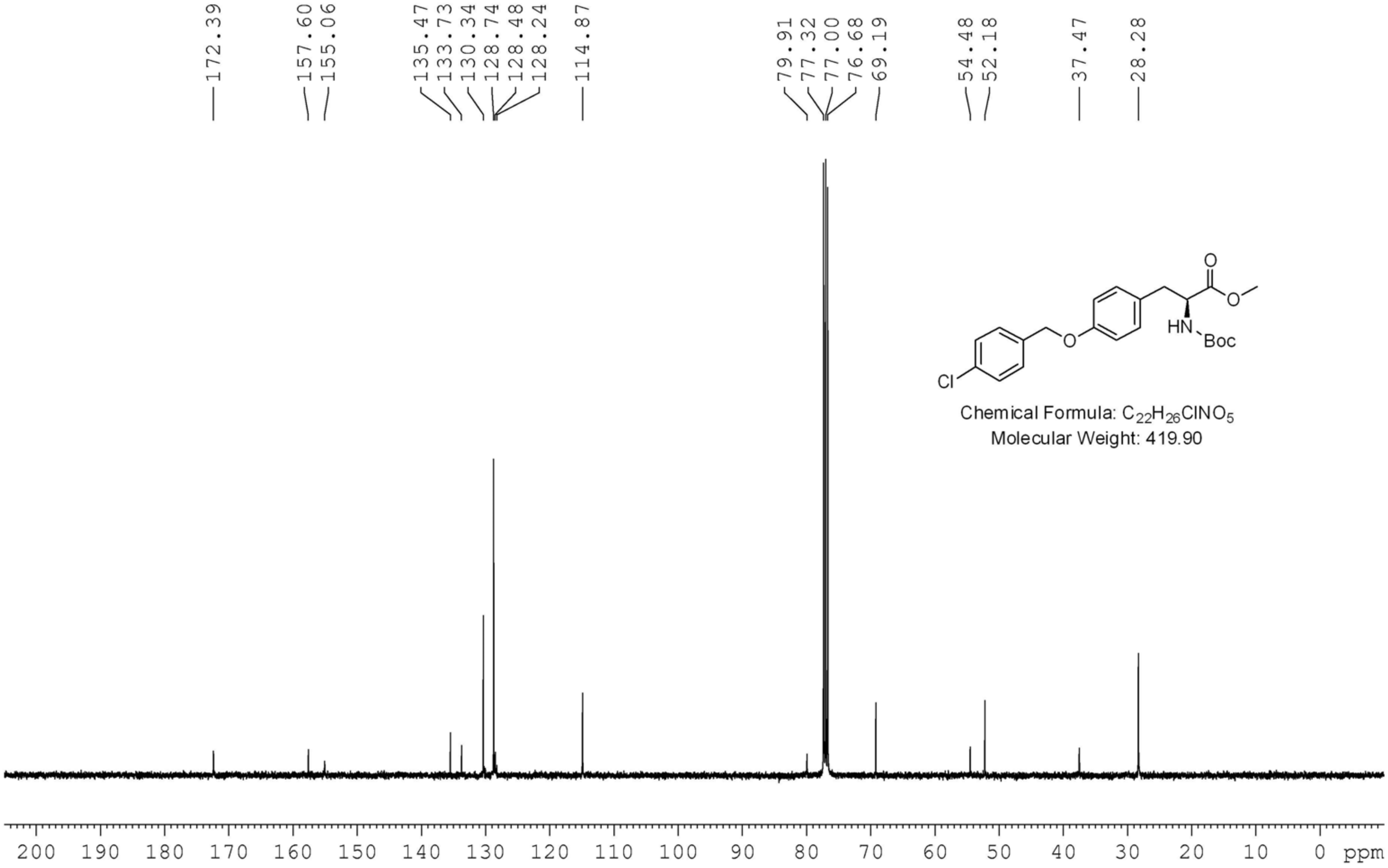

Chemical Formula: $C_{22}H_{26}ClNO_5$

Molecular Weight: 419.90

Solvent: $CDCl_3$ & NMR: 500 MHz

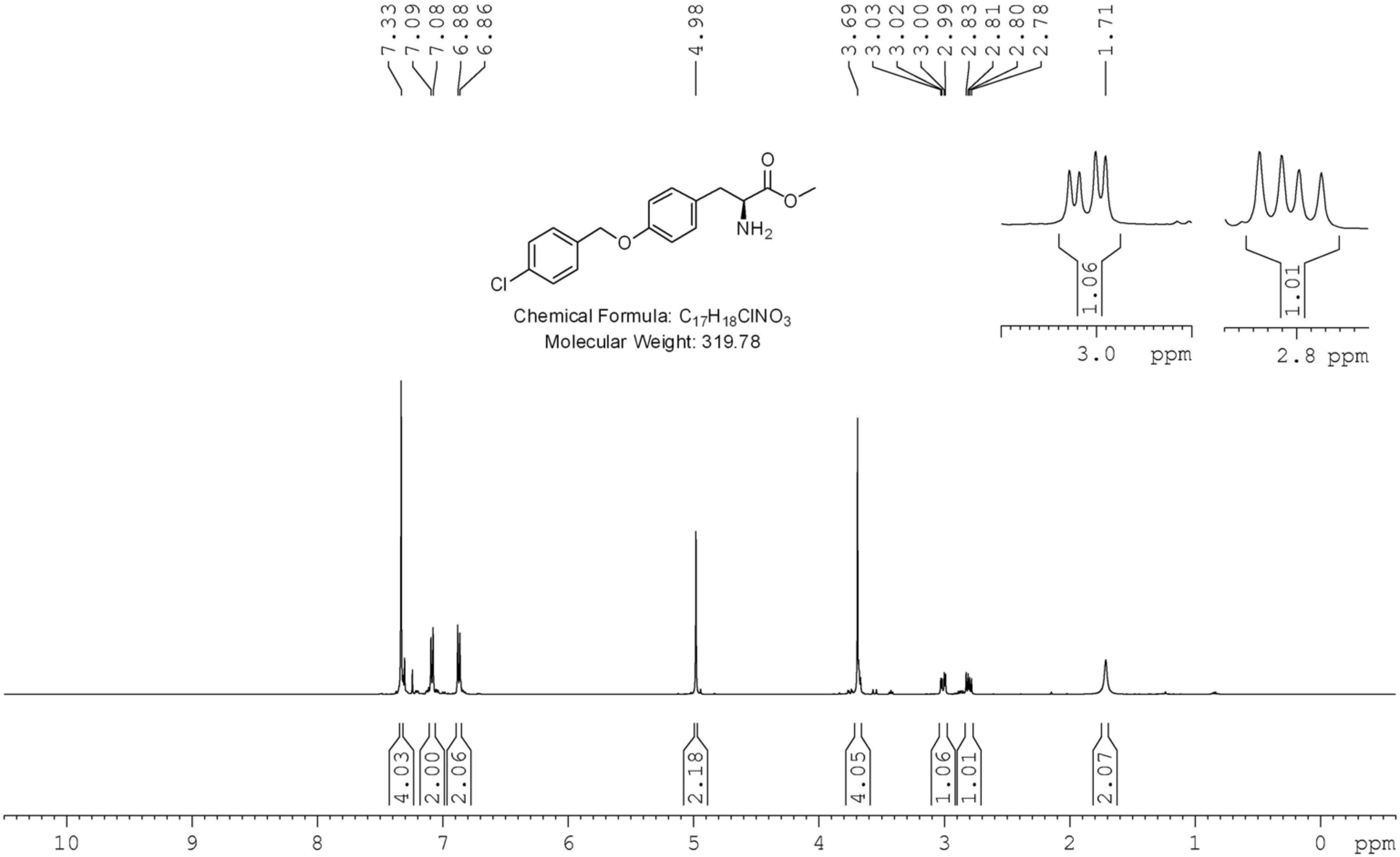

Solvent: $CDCl_3$ & NMR: 125 MHz

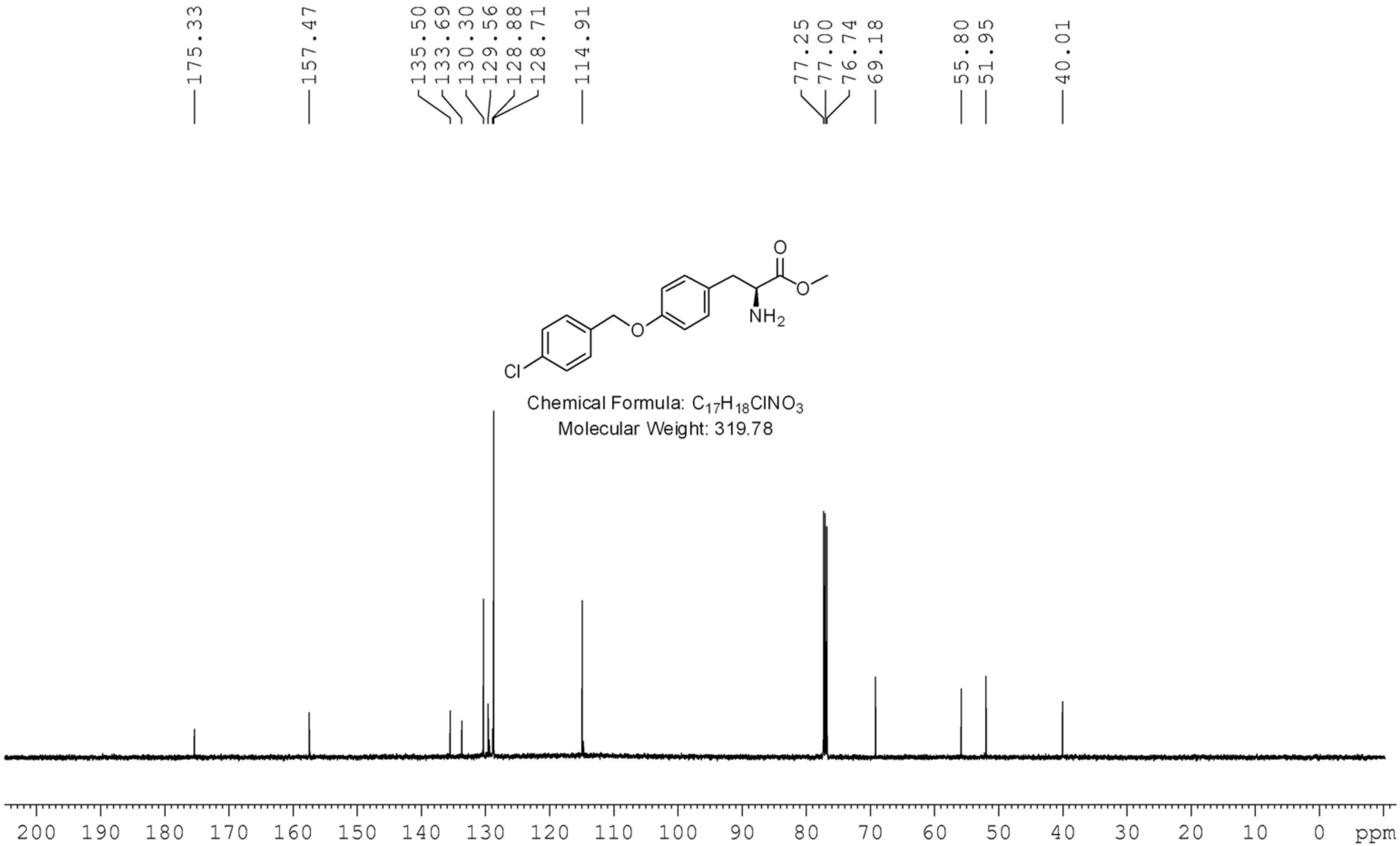

Solvent: $CDCl_3$ & NMR: 500 MHz

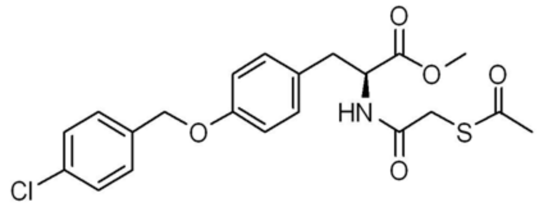

Chemical Formula: $C_{21}H_{22}ClNO_5S$
Molecular Weight: 435.92

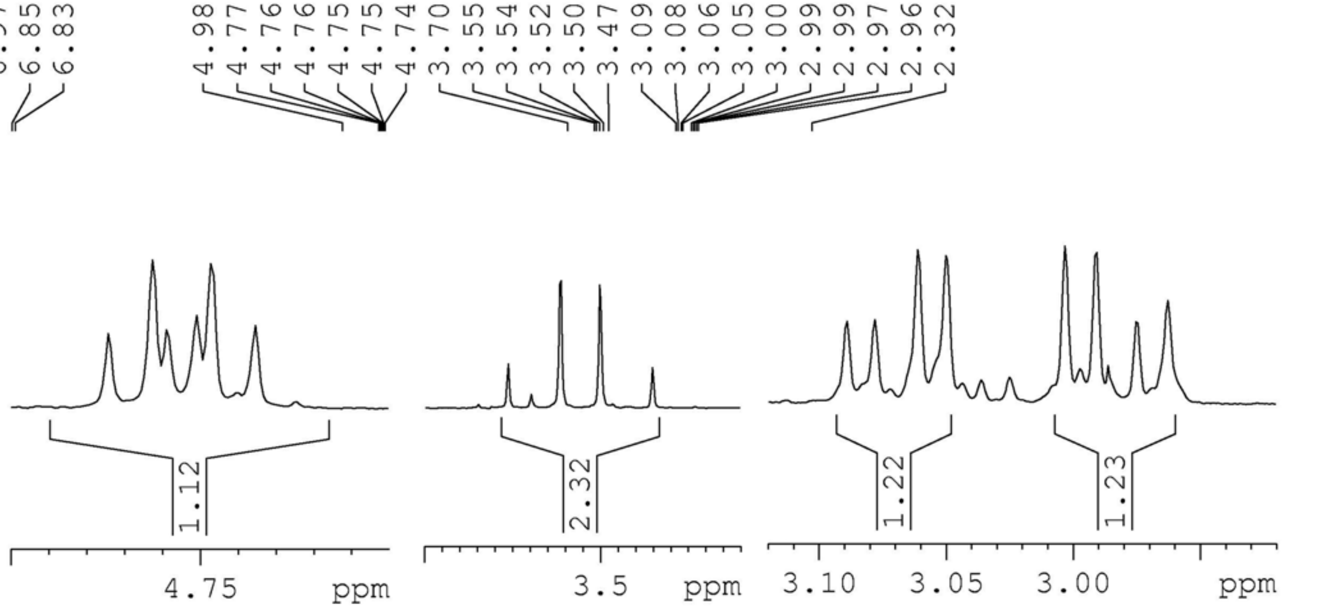

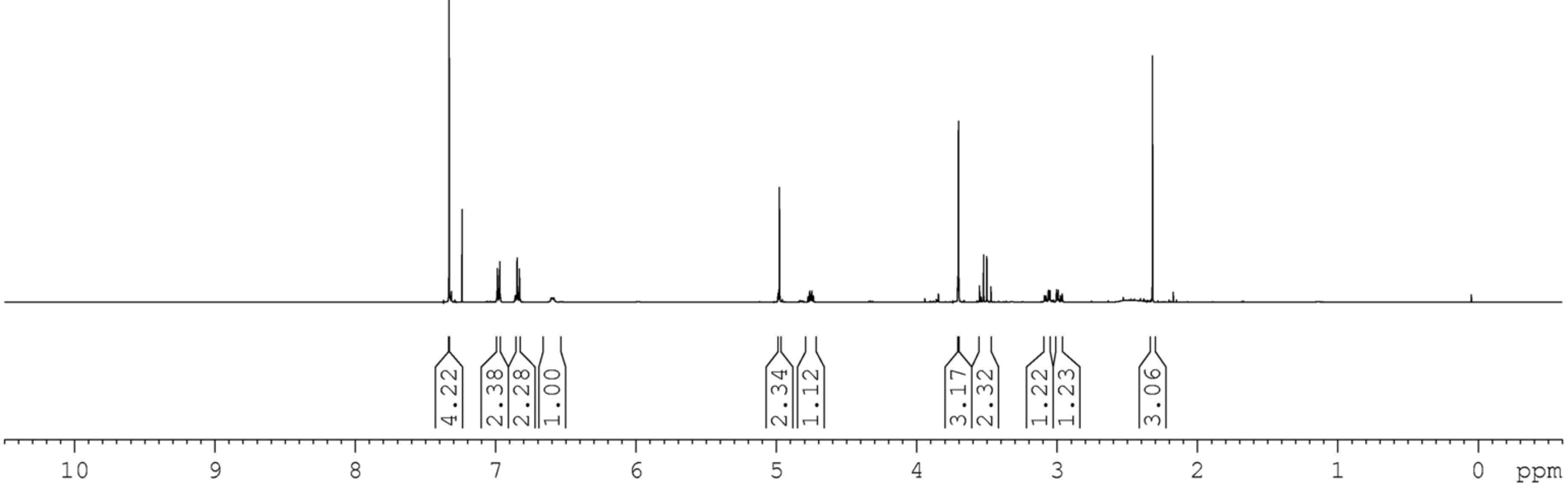

Solvent: $CDCl_3$ & NMR: 125 MHz

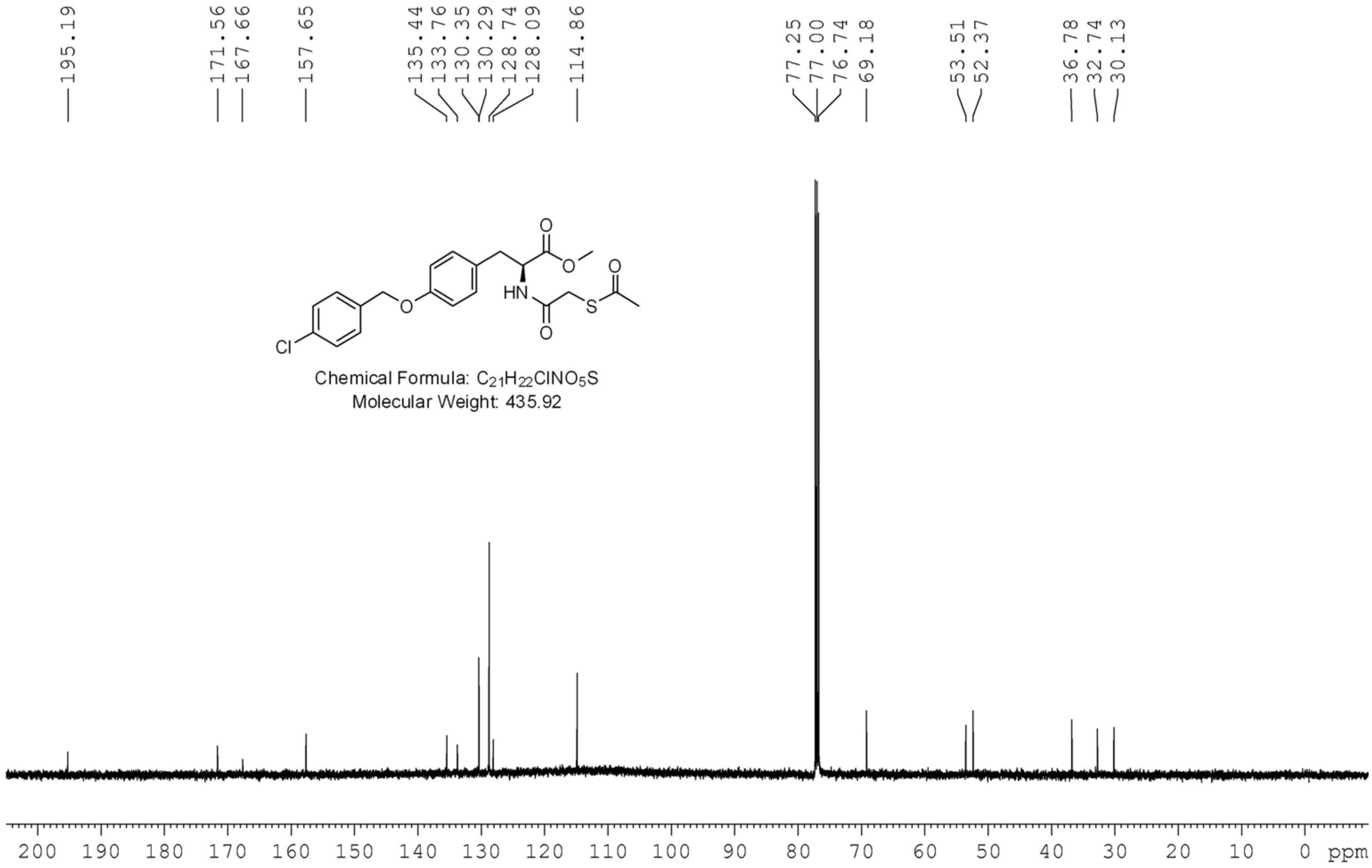

Solvent: CD_3OD & NMR: 500 MHz

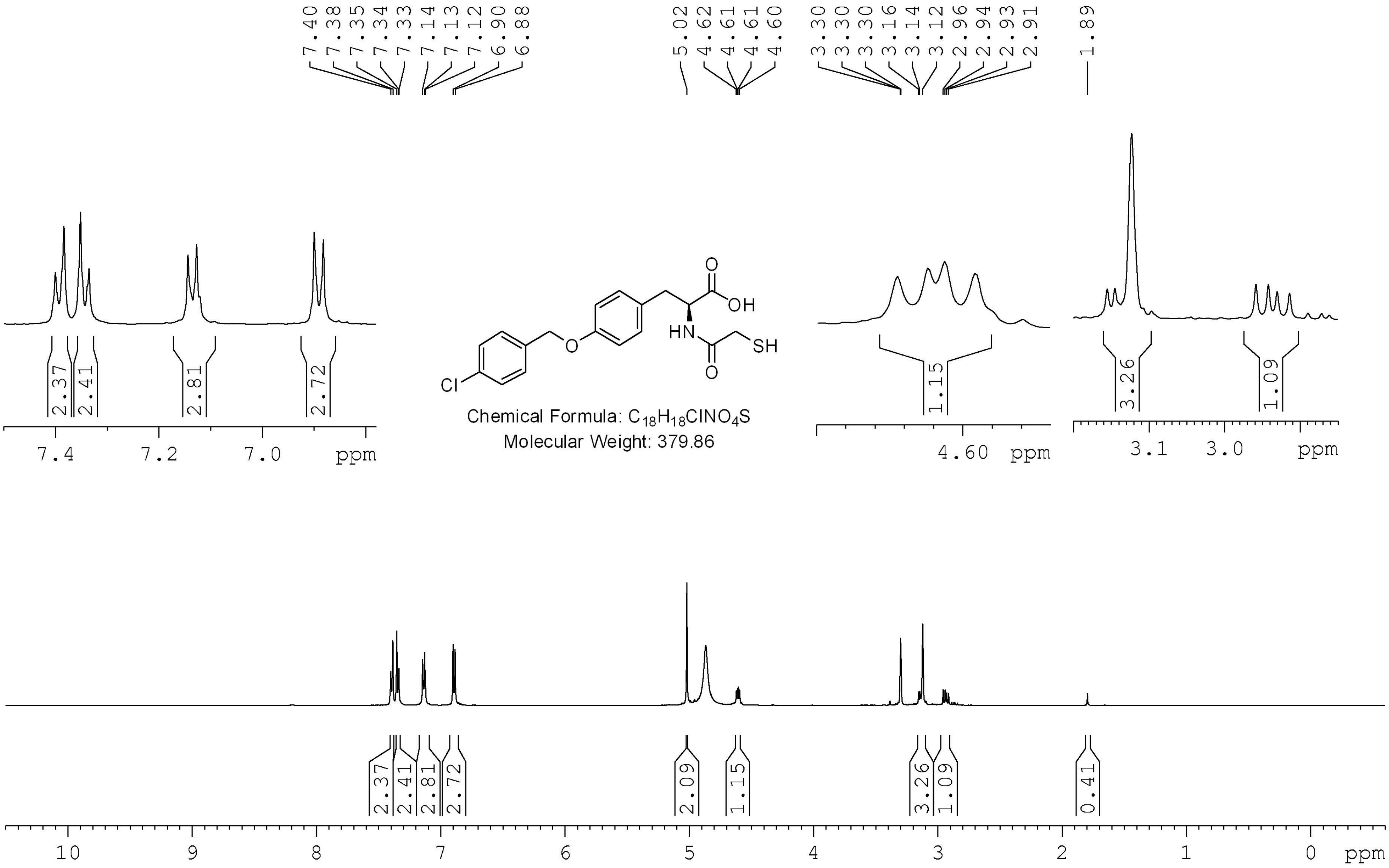

Solvent: CD_3OD & NMR: 125 MHz

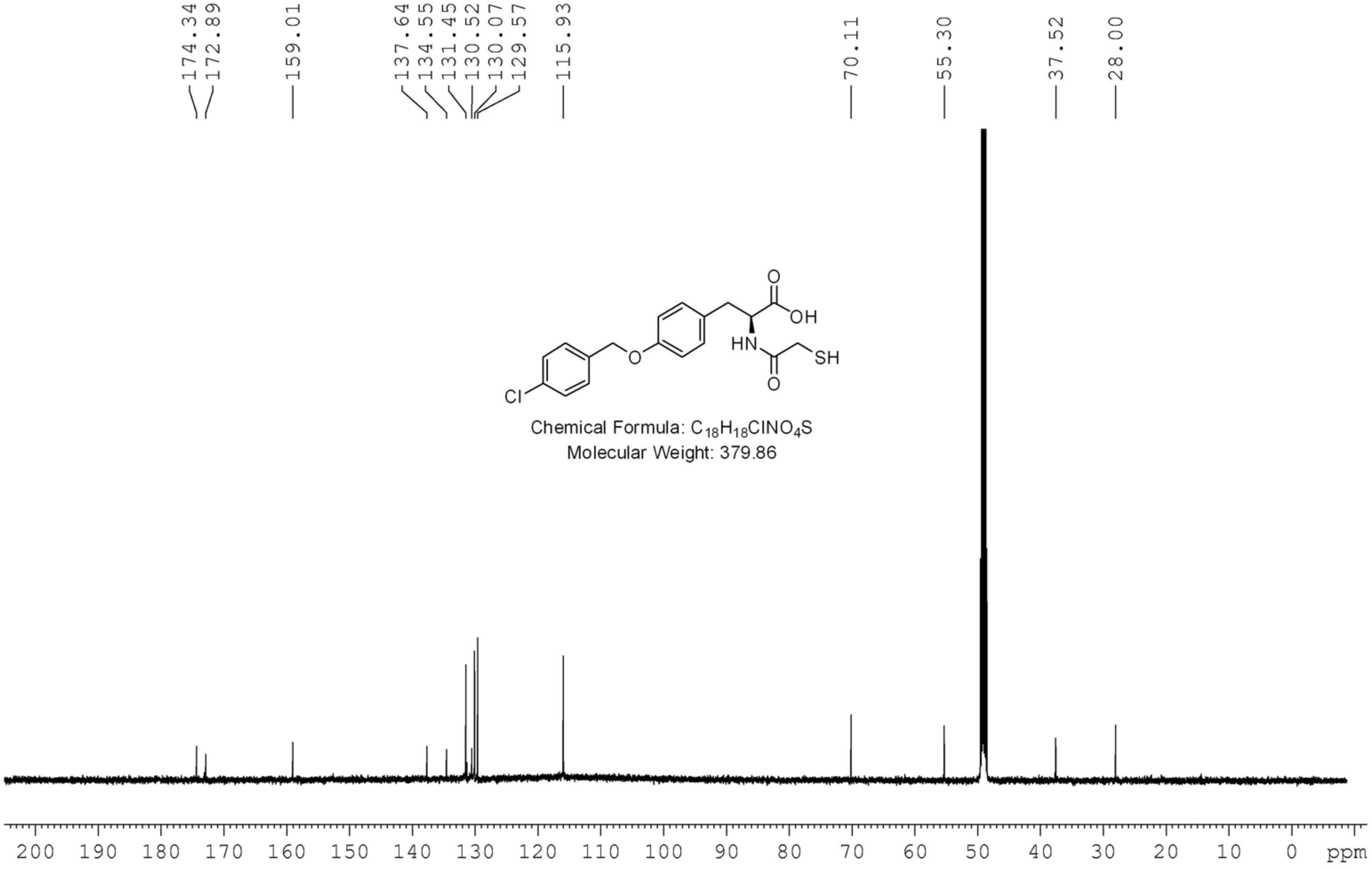

Chemical Formula: $C_{18}H_{18}ClNO_4S$

Molecular Weight: 379.86

Solvent: $CDCl_3$ & NMR: 400 MHz

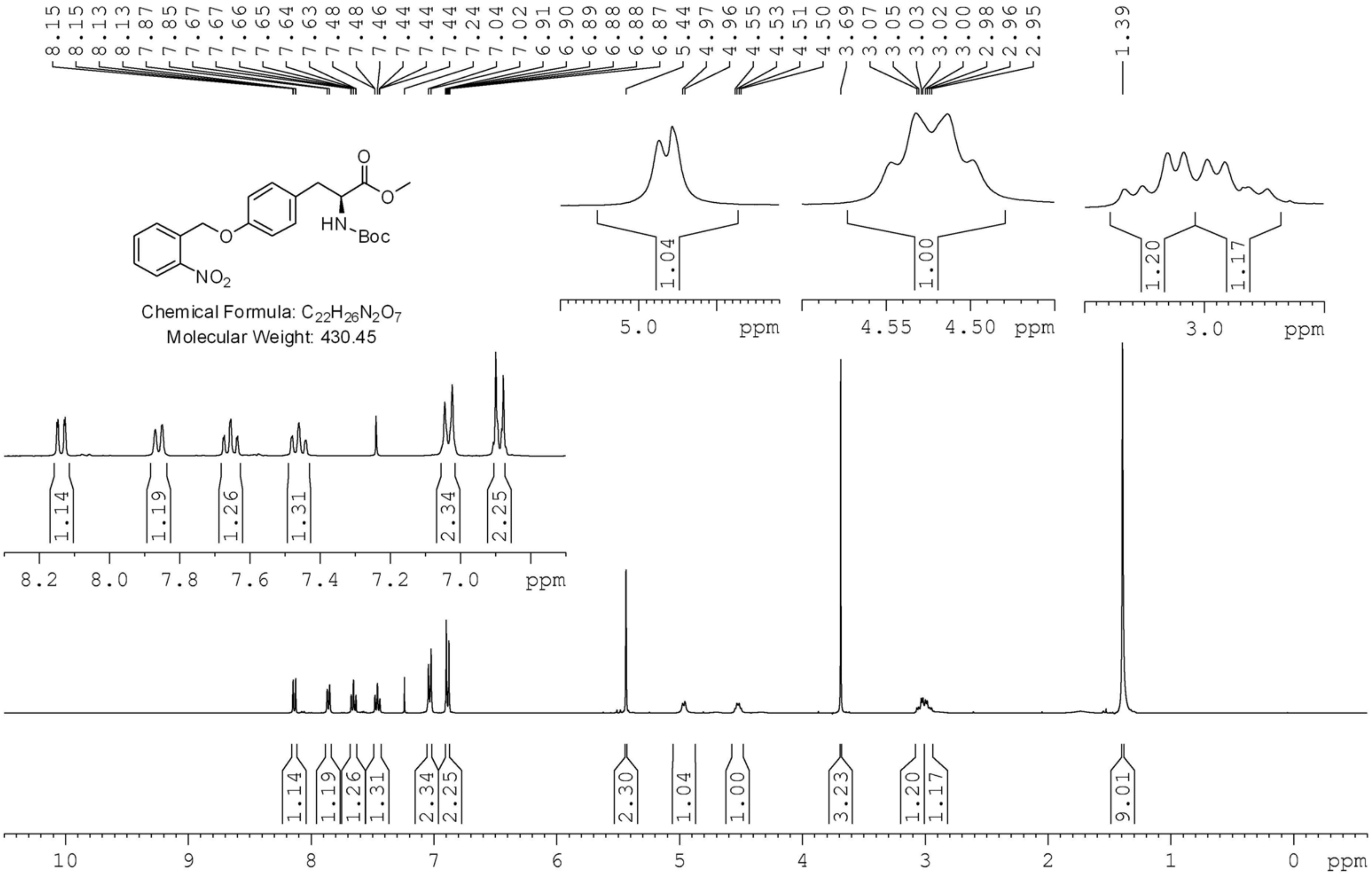

Chemical Formula: $C_{22}H_{26}N_2O_7$

Molecular Weight: 430.45

Solvent: $CDCl_3$ & NMR: 100 MHz

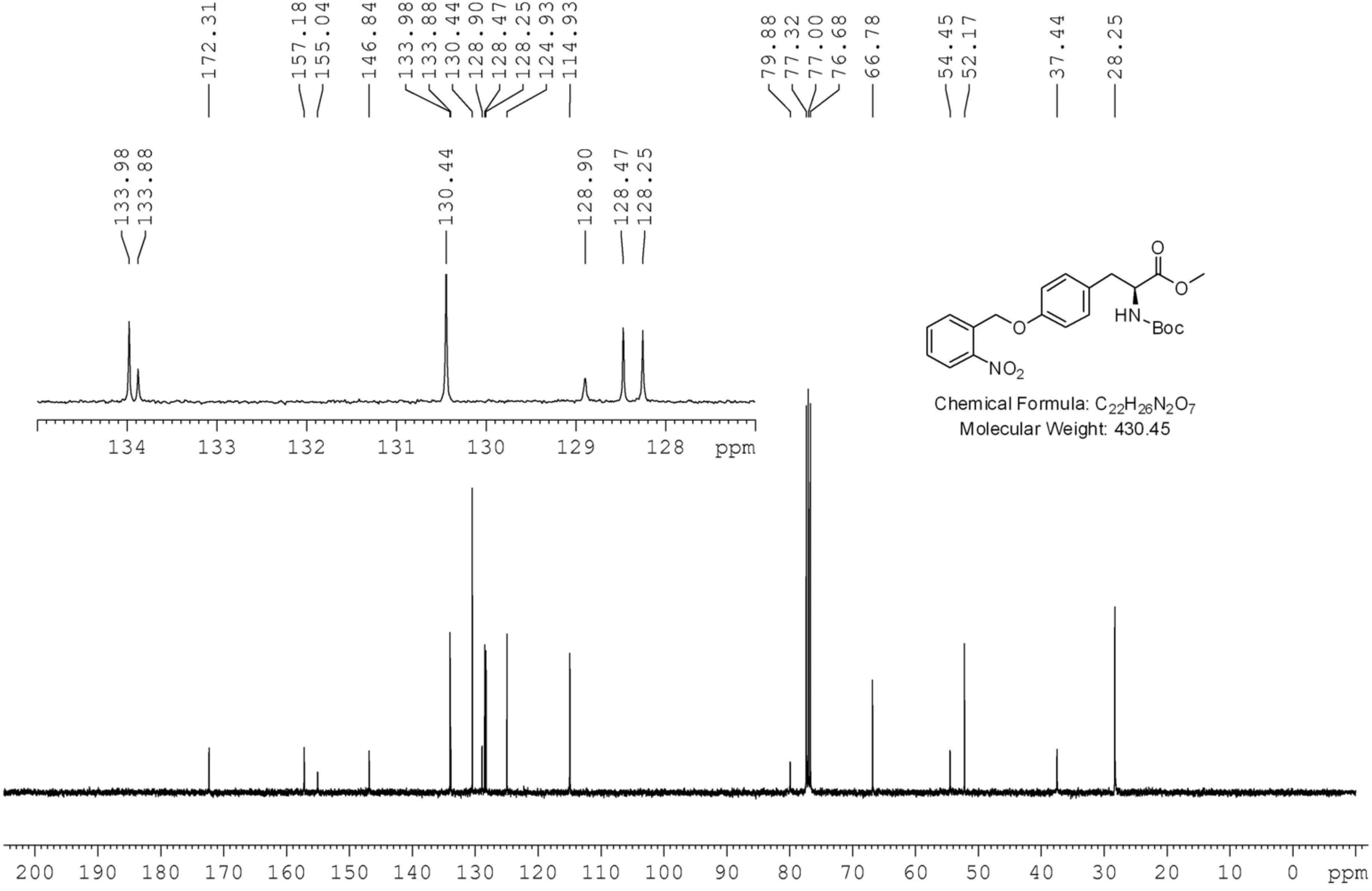

Solvent: $CDCl_3$ & NMR: 500 MHz

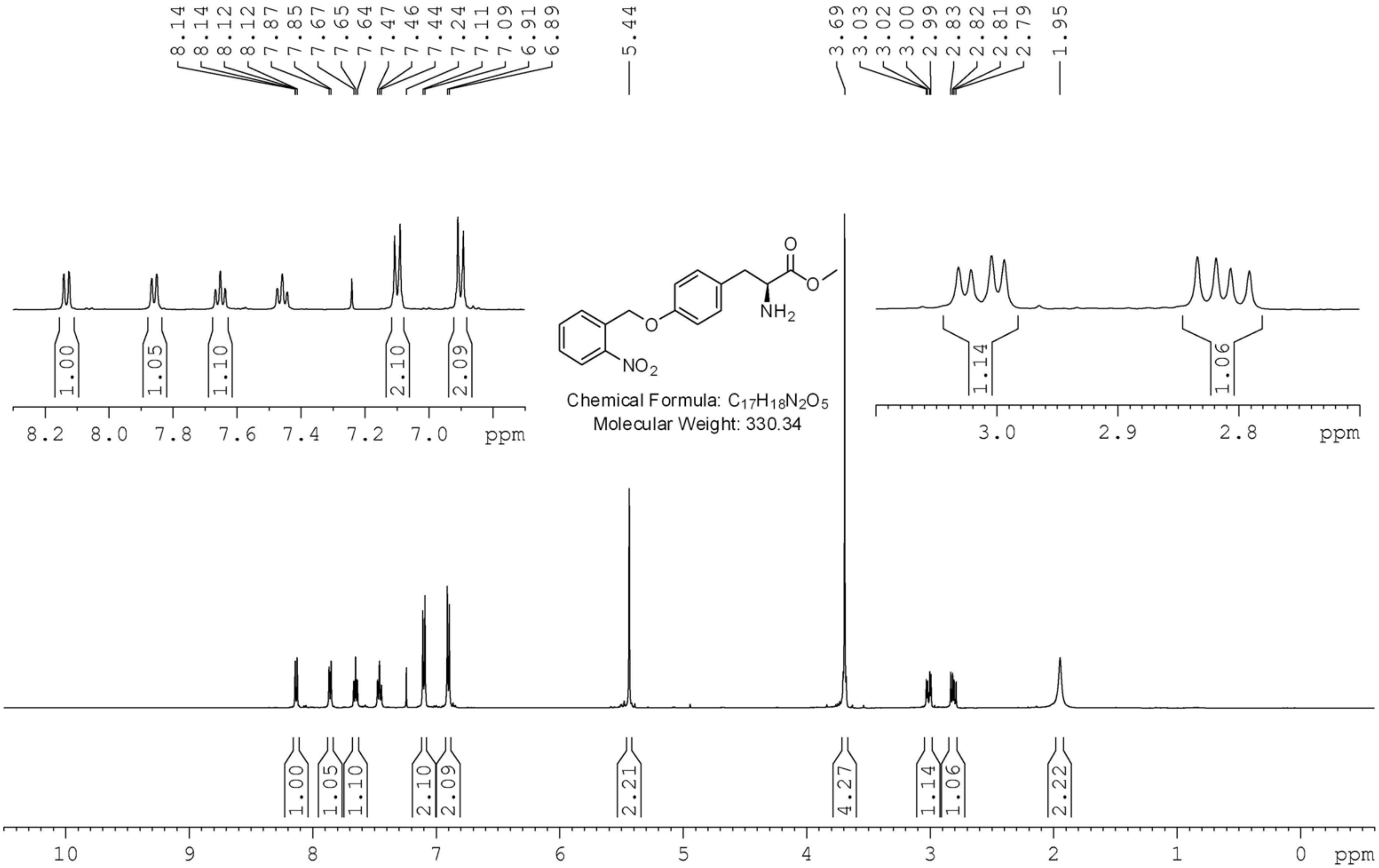

Chemical Formula: $C_{17}H_{18}N_2O_5$

Molecular Weight: 330.34

Solvent: $CDCl_3$ & NMR: 125 MHz

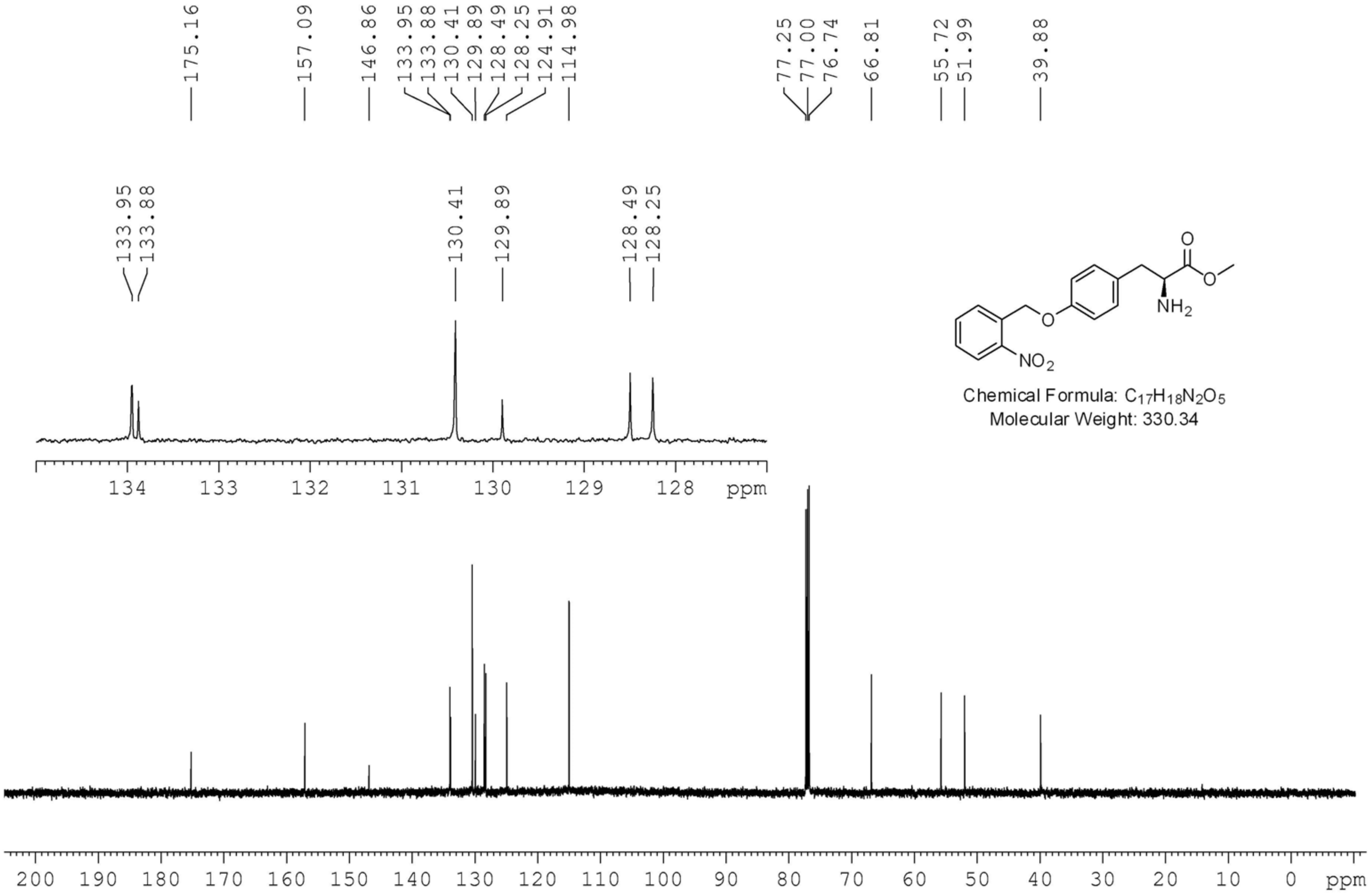

Chemical Formula: $C_{17}H_{18}N_2O_5$
Molecular Weight: 330.34

Solvent: $CDCl_3$ & NMR: 500 MHz

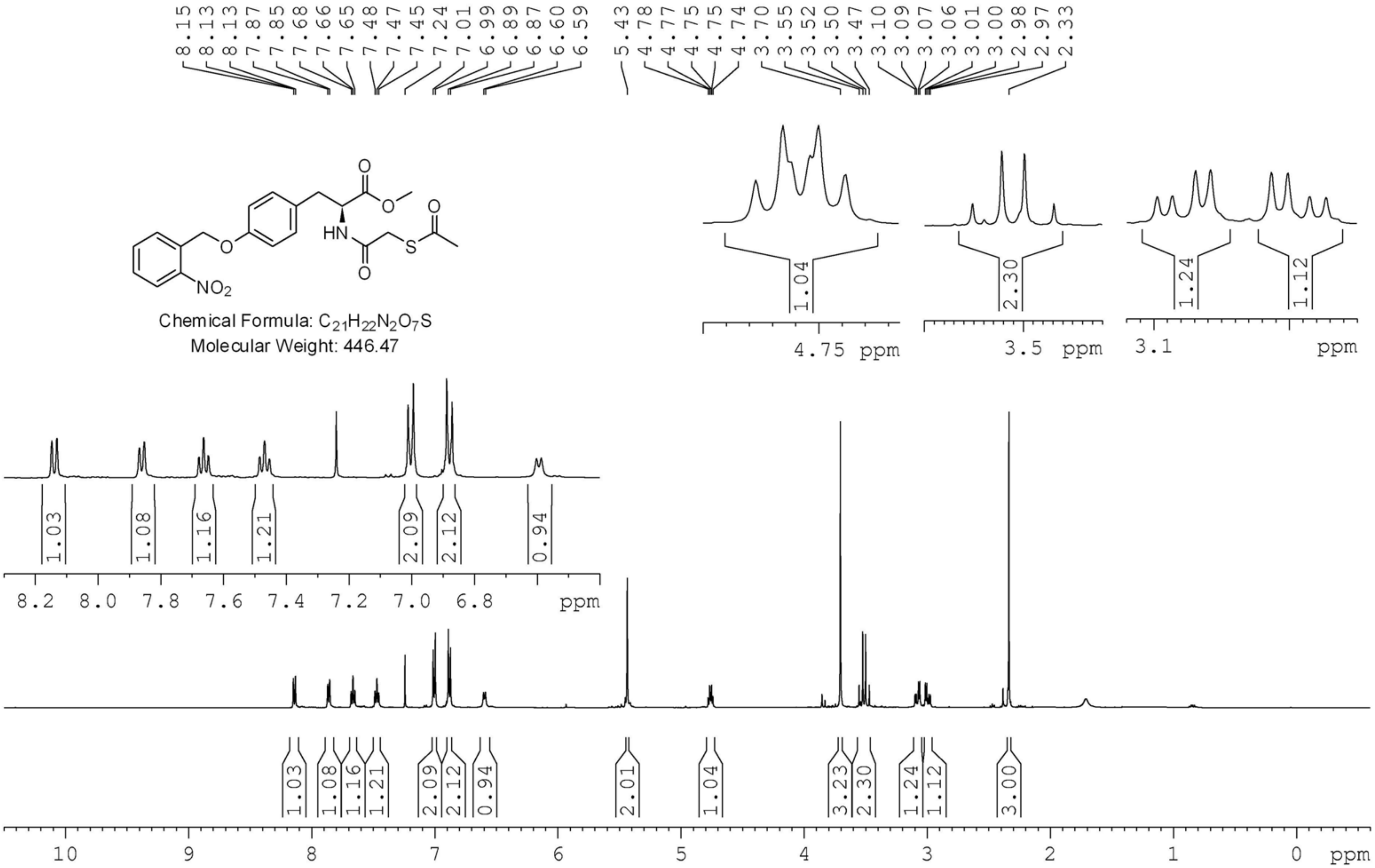

Chemical Formula: $C_{21}H_{22}N_2O_7S$
Molecular Weight: 446.47

Solvent: $CDCl_3$ & NMR: 125 MHz

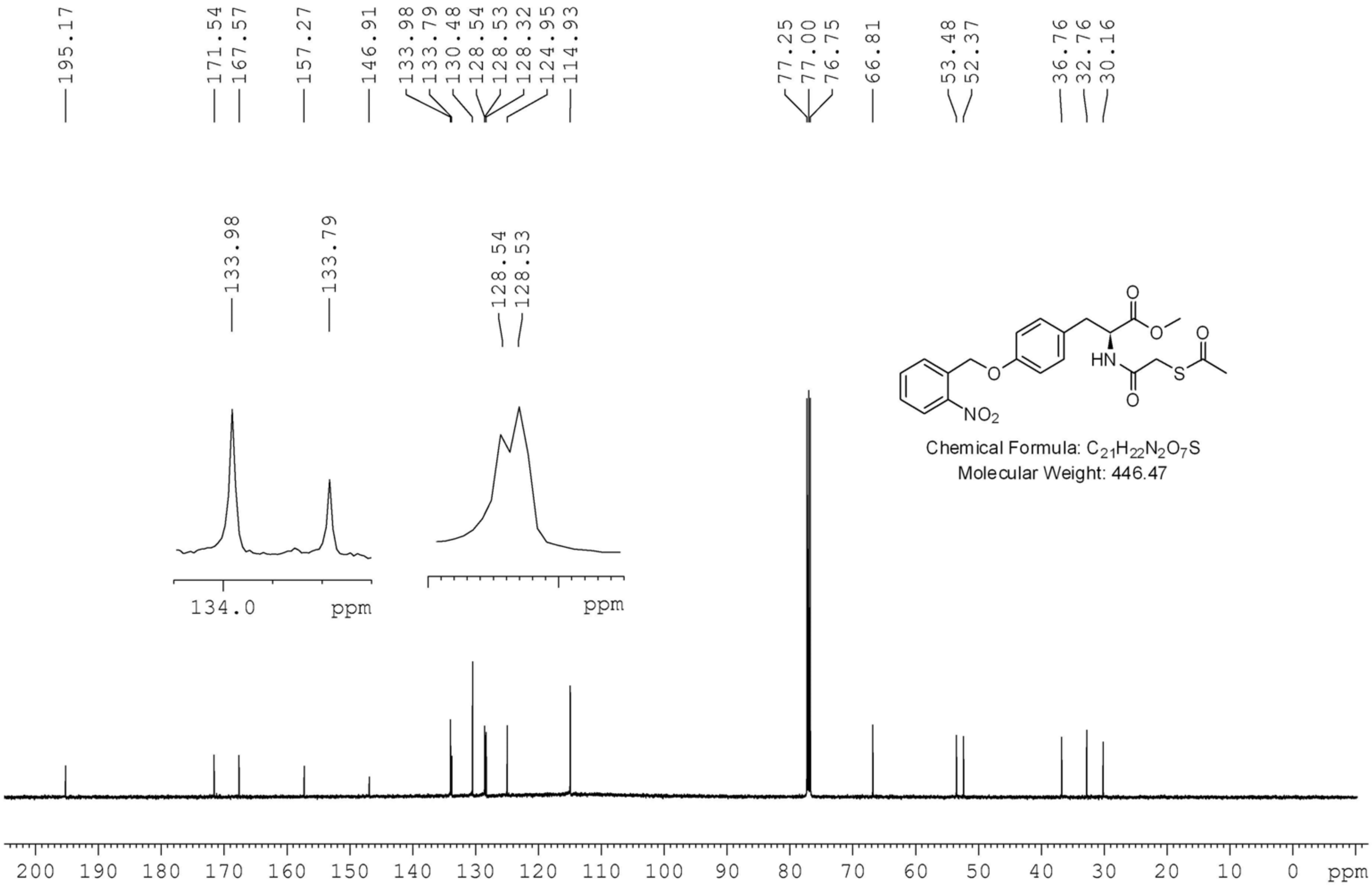

Solvent: $CDCl_3$ & NMR: 500 MHz

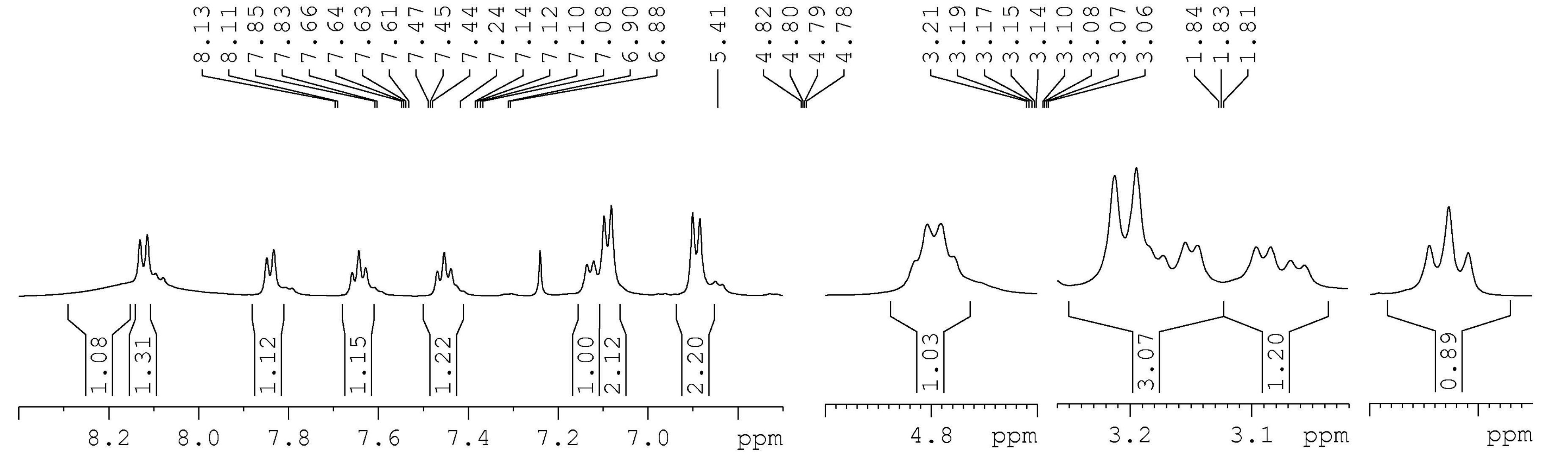

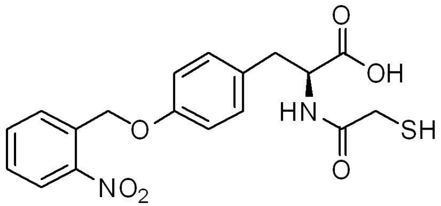

Chemical Formula: $C_{18}H_{18}N_2O_6S$
Molecular Weight: 390.41

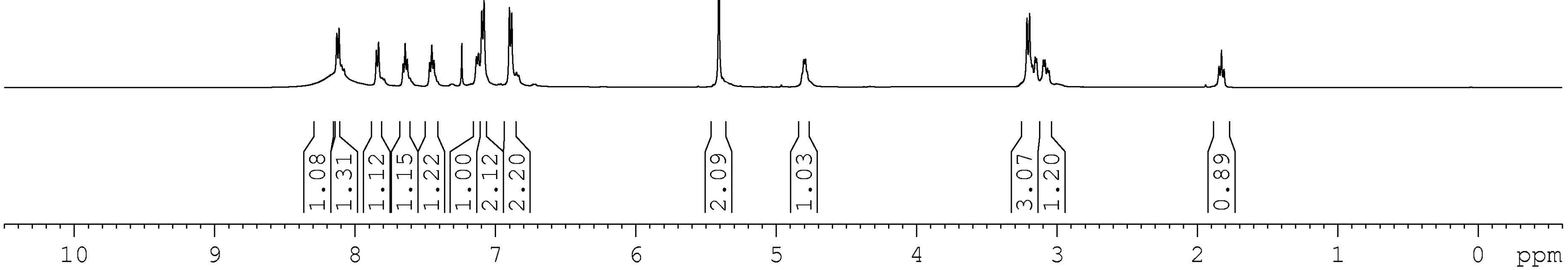

Solvent: $CDCl_3$ & NMR: 125 MHz

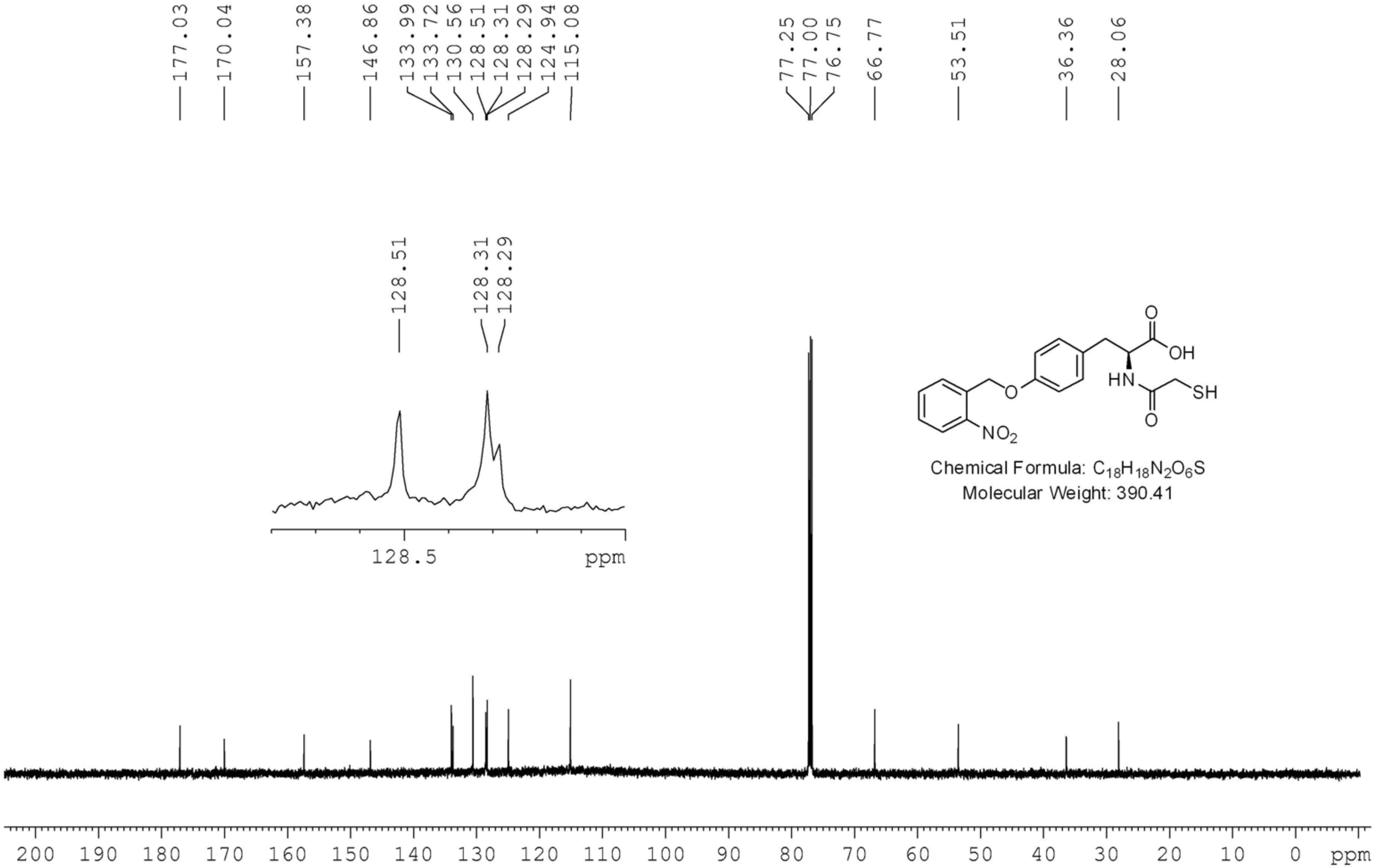

Solvent: $CDCl_3$ & NMR: 500 MHz

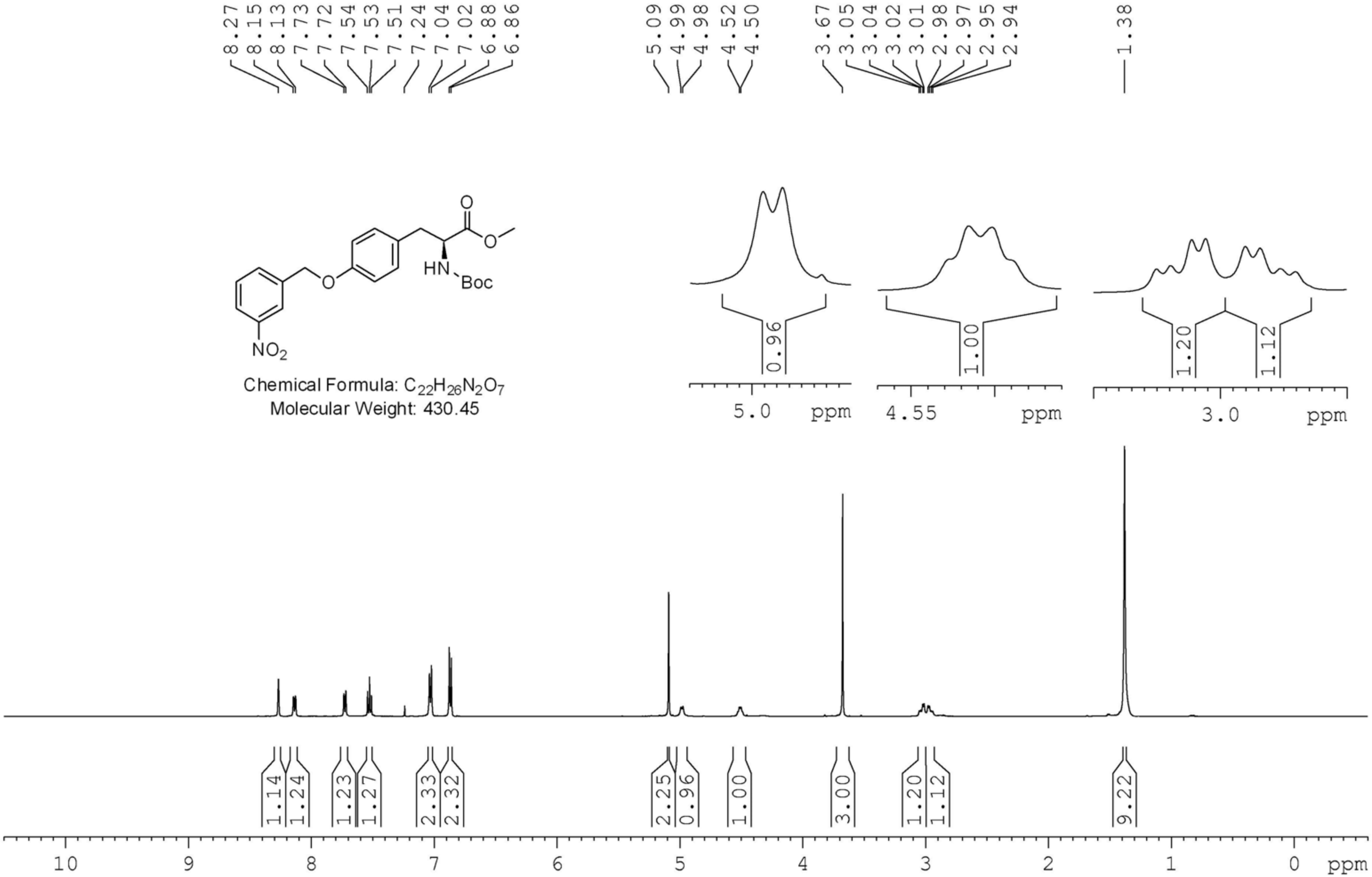

Chemical Formula: $C_{22}H_{26}N_2O_7$
Molecular Weight: 430.45

Solvent: $CDCl_3$ & NMR: 125 MHz

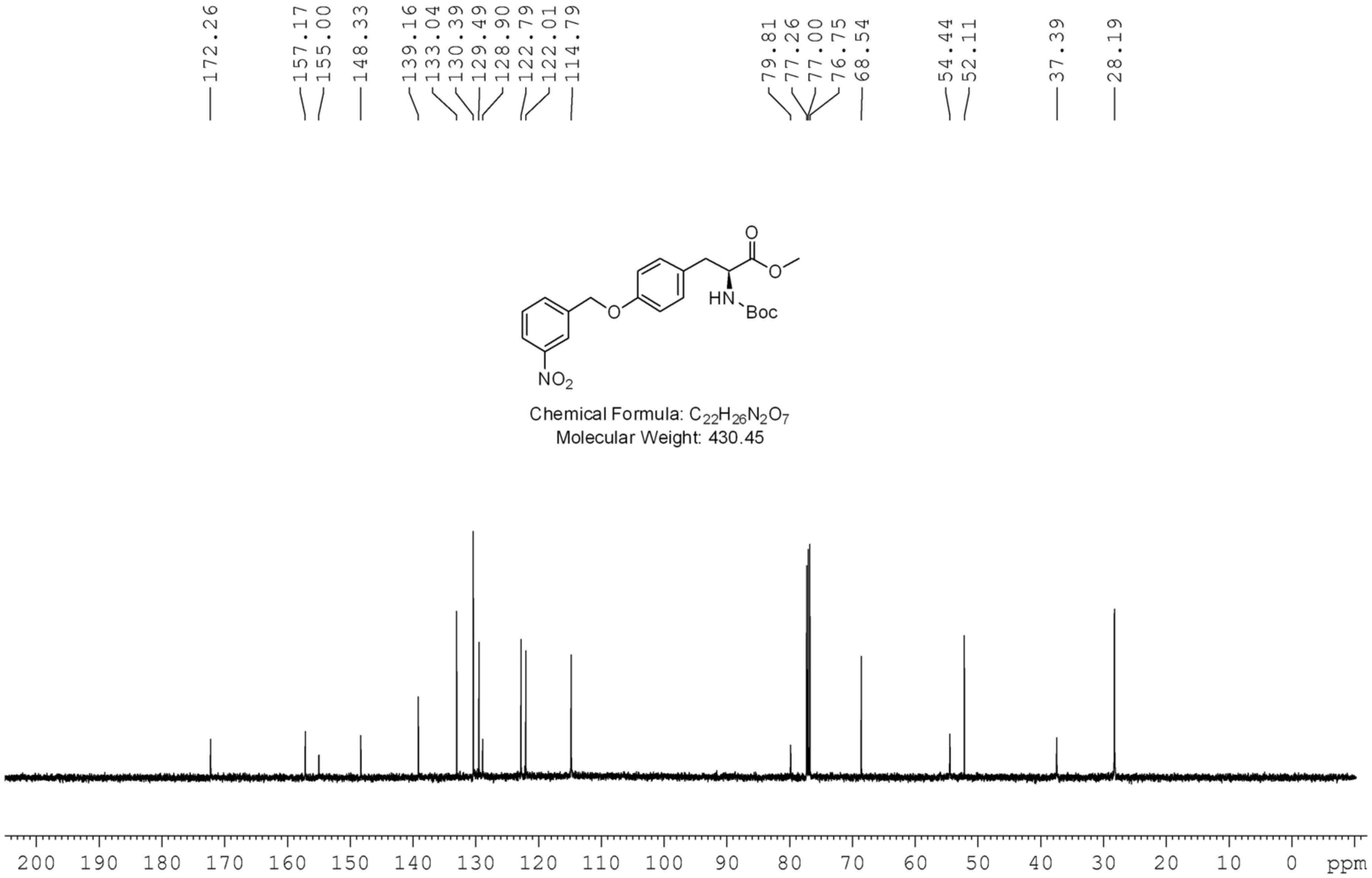

Solvent: $CDCl_3$ & NMR: 400 MHz

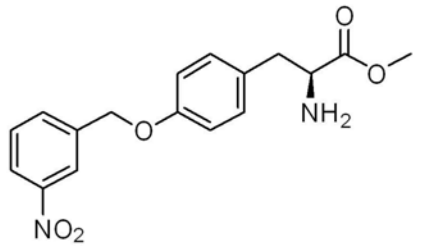

Chemical Formula: $C_{17}H_{18}N_2O_5$
Molecular Weight: 330.34

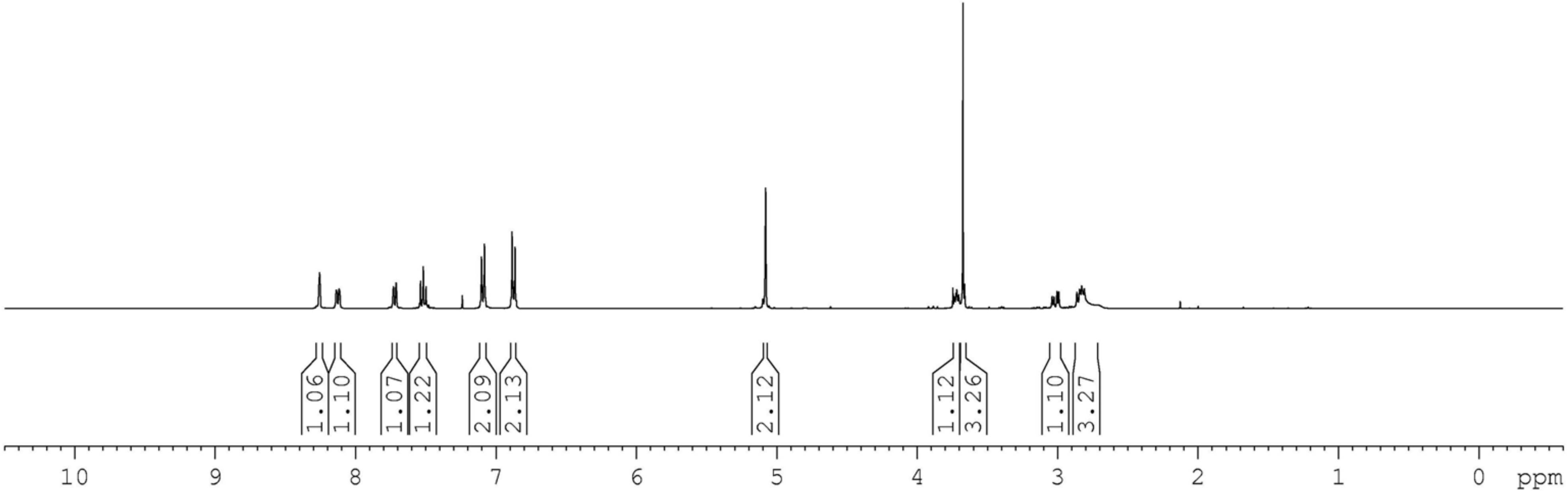

Solvent: $CDCl_3$ & NMR: 100 MHz

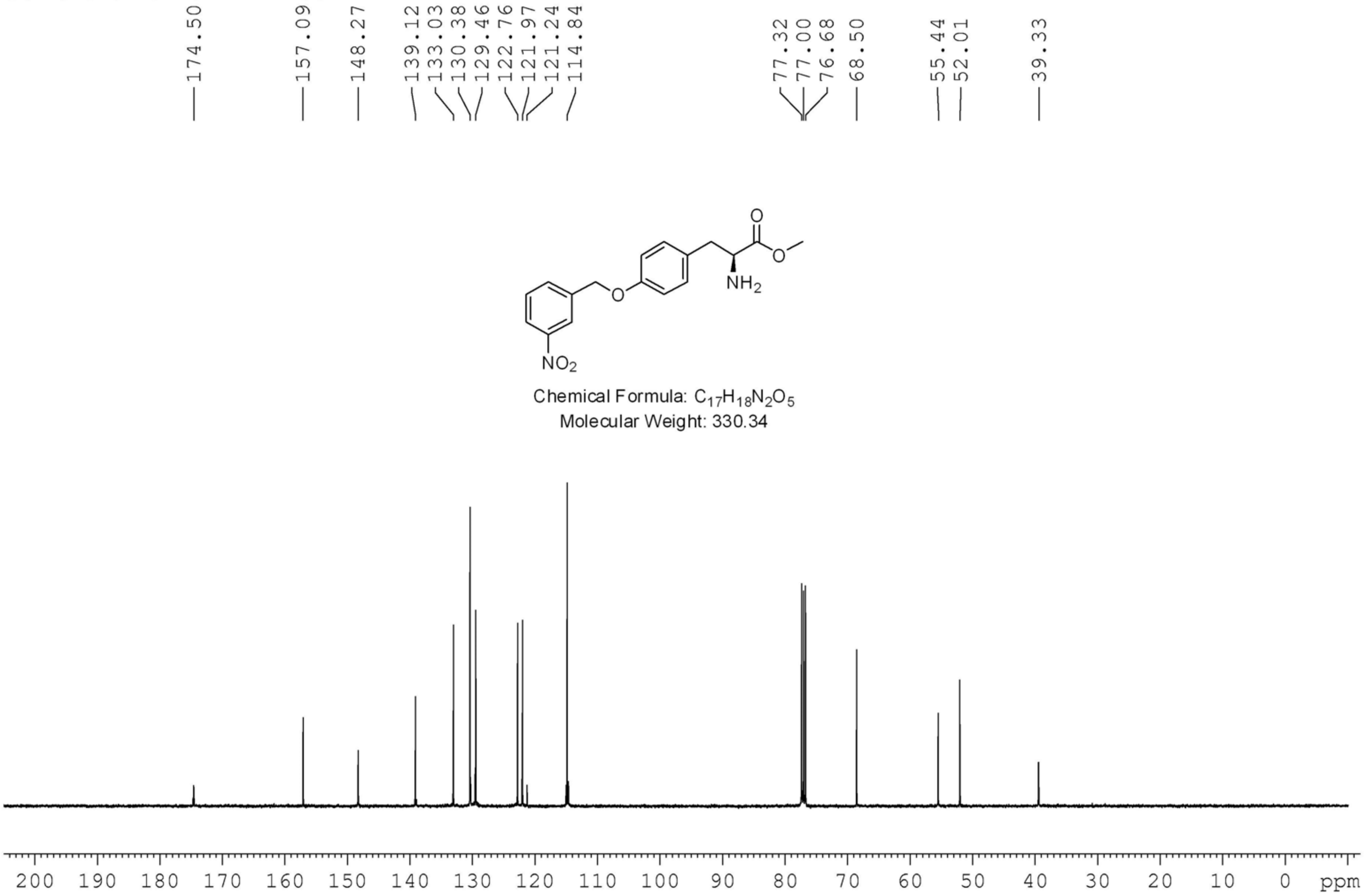

Solvent: $CDCl_3$ & NMR: 400 MHz

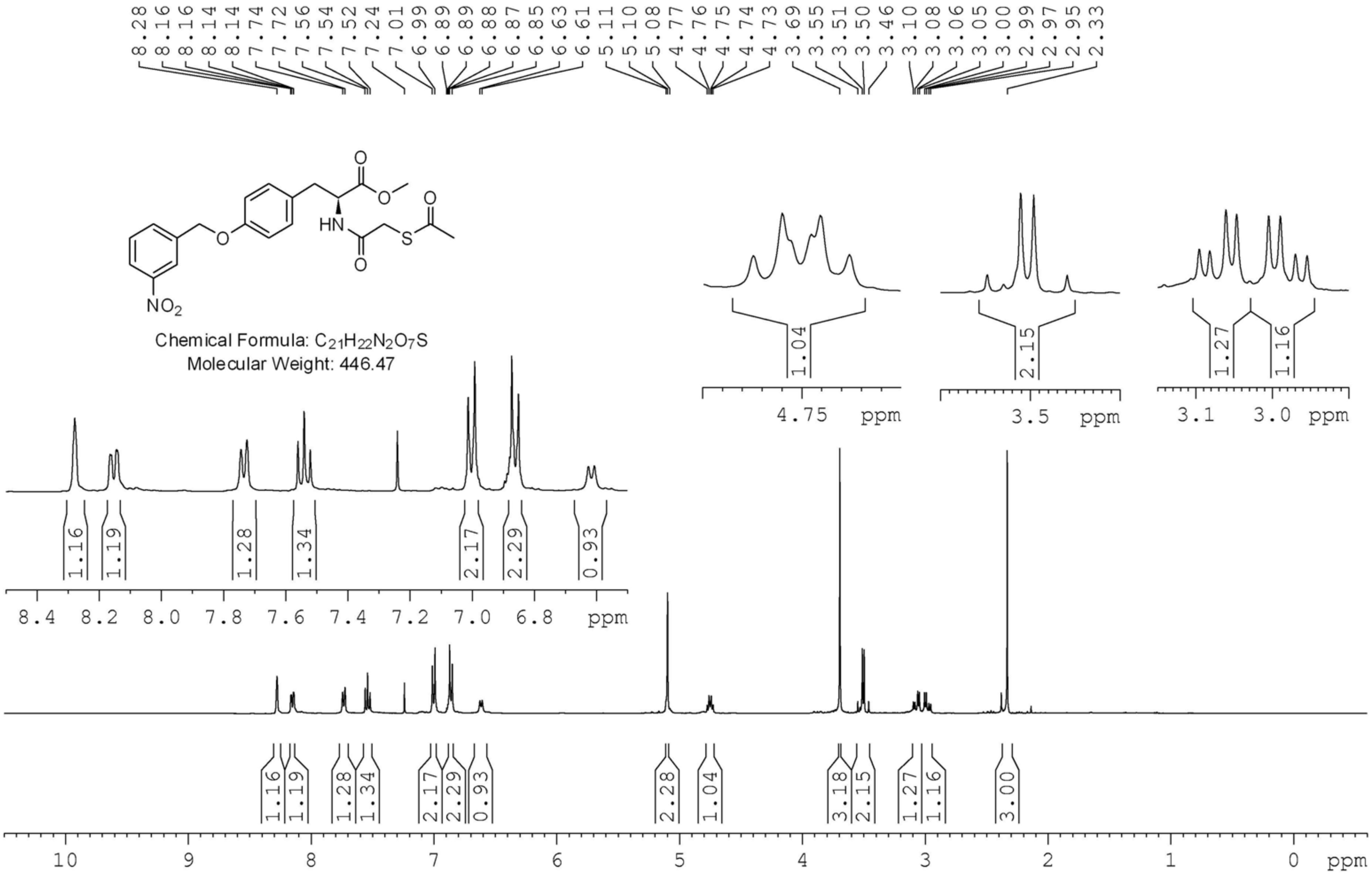

Solvent: $CDCl_3$ & NMR: 100 MHz

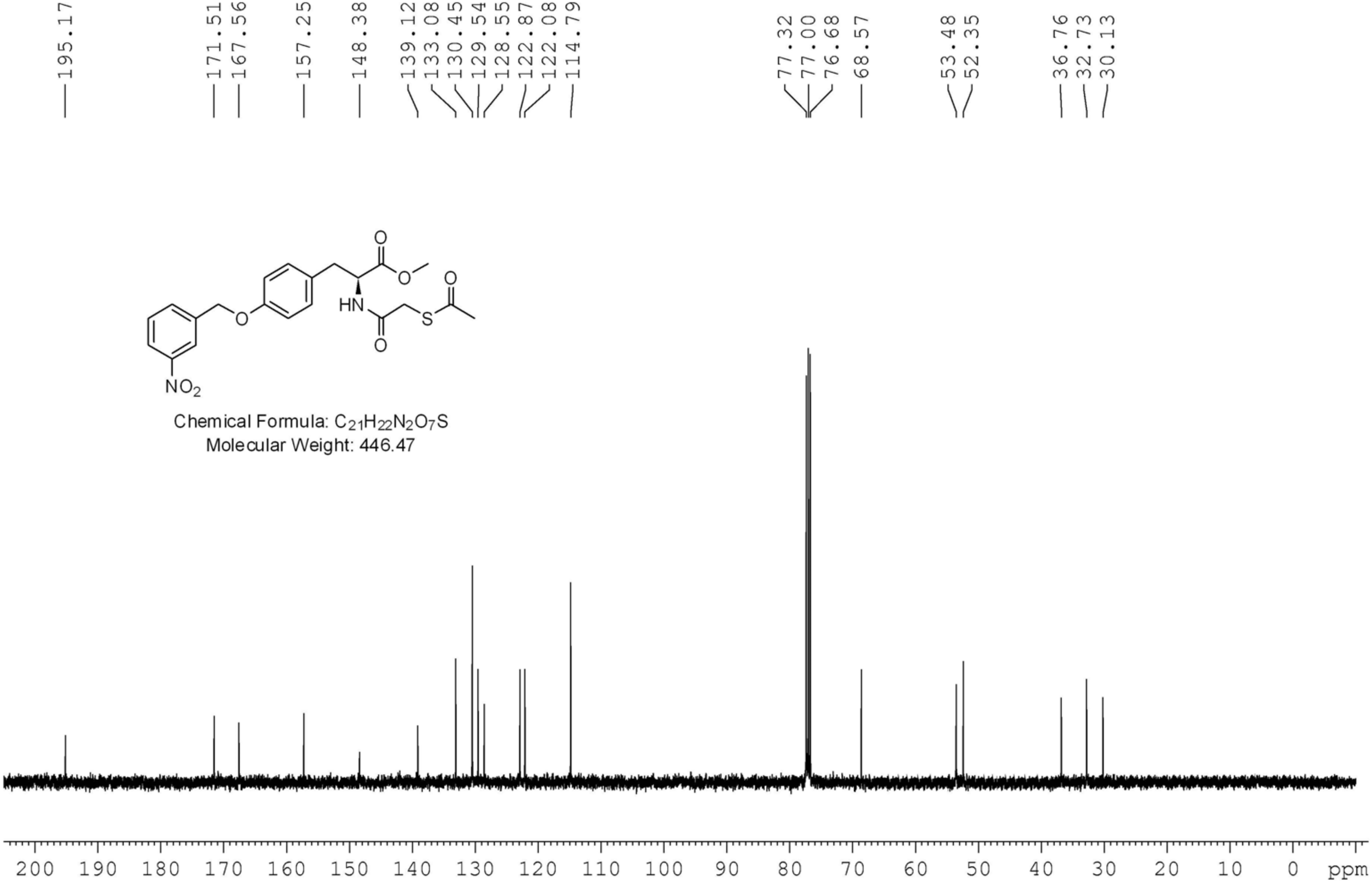

Chemical Formula: $C_{21}H_{22}N_2O_7S$
Molecular Weight: 446.47

Solvent: $CDCl_3$ & NMR: 400 MHz

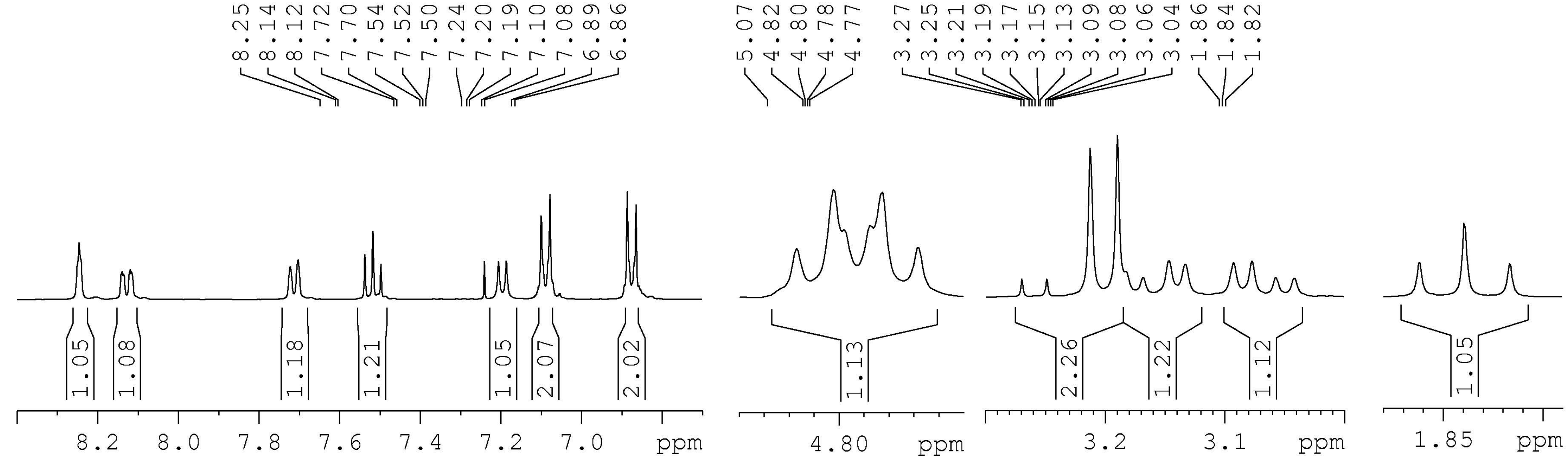

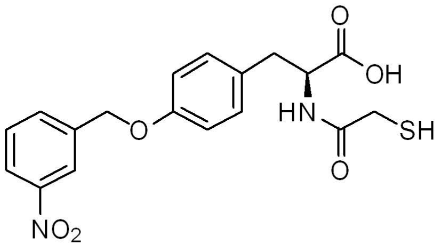

Chemical Formula: $C_{18}H_{18}N_2O_6S$
Molecular Weight: 390.41

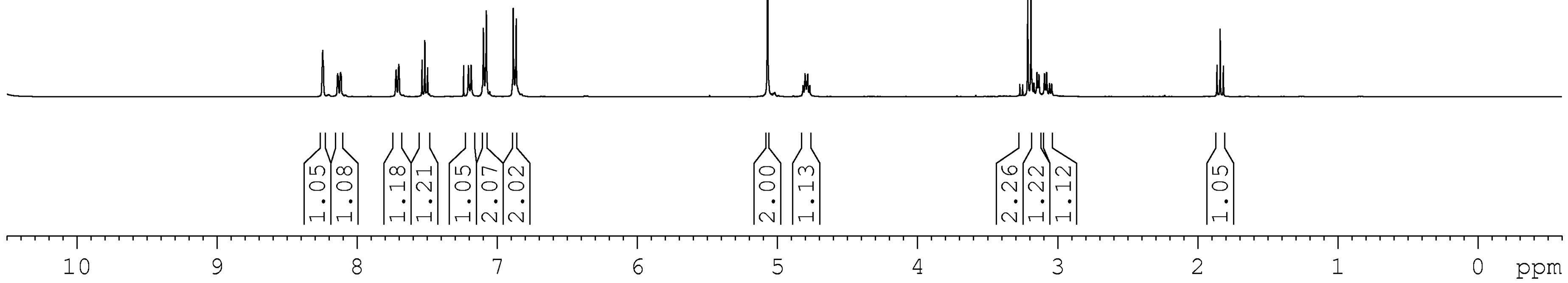

Solvent: $CDCl_3$ & NMR: 100 MHz

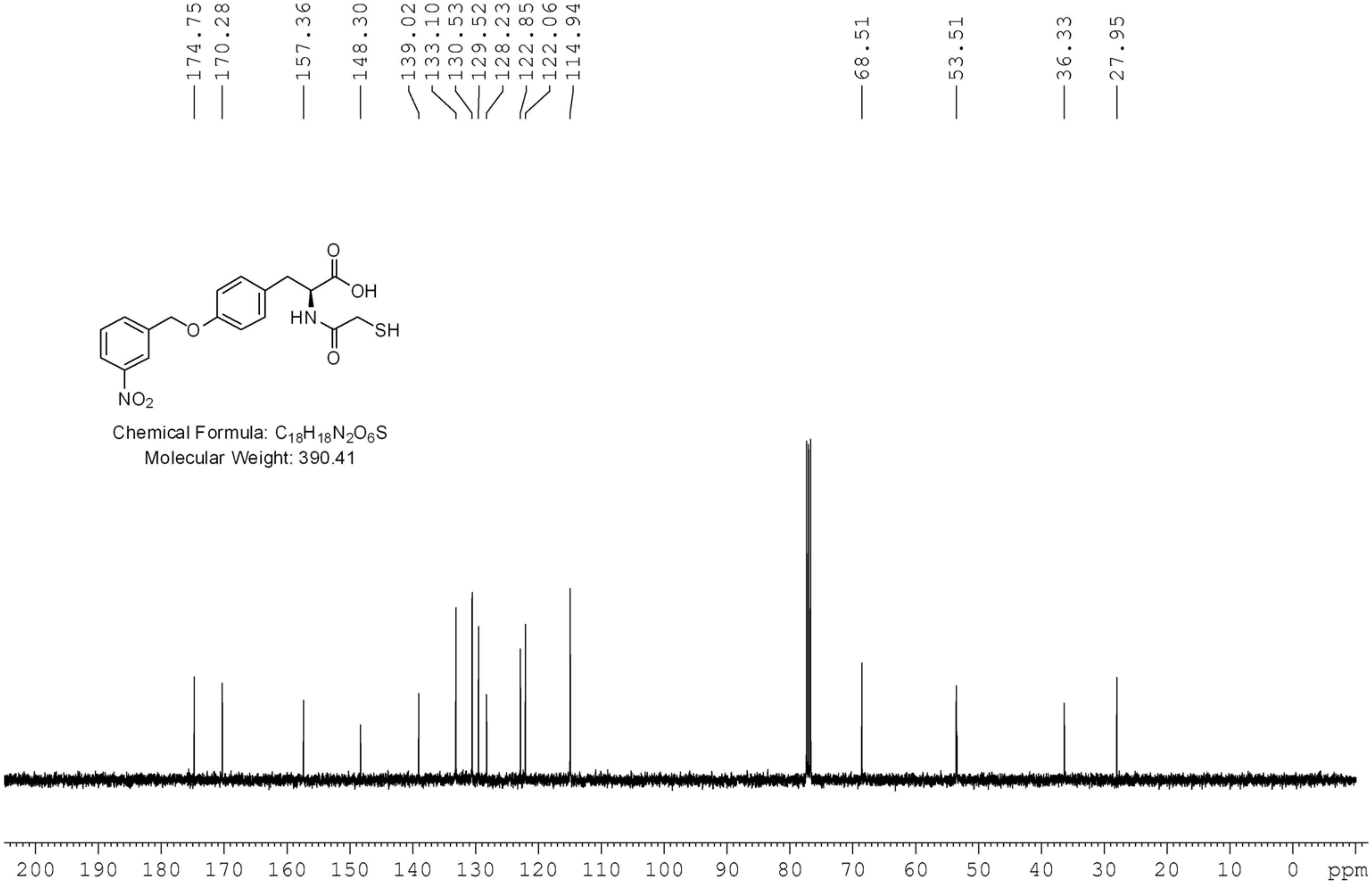

Chemical Formula: $C_{18}H_{18}N_2O_6S$

Molecular Weight: 390.41

Solvent: $CDCl_3$ & NMR: 500 MHz

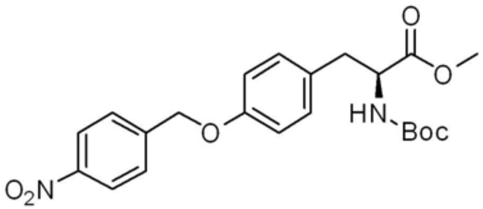

Chemical Formula: $C_{22}H_{26}N_2O_7$
Molecular Weight: 430.45

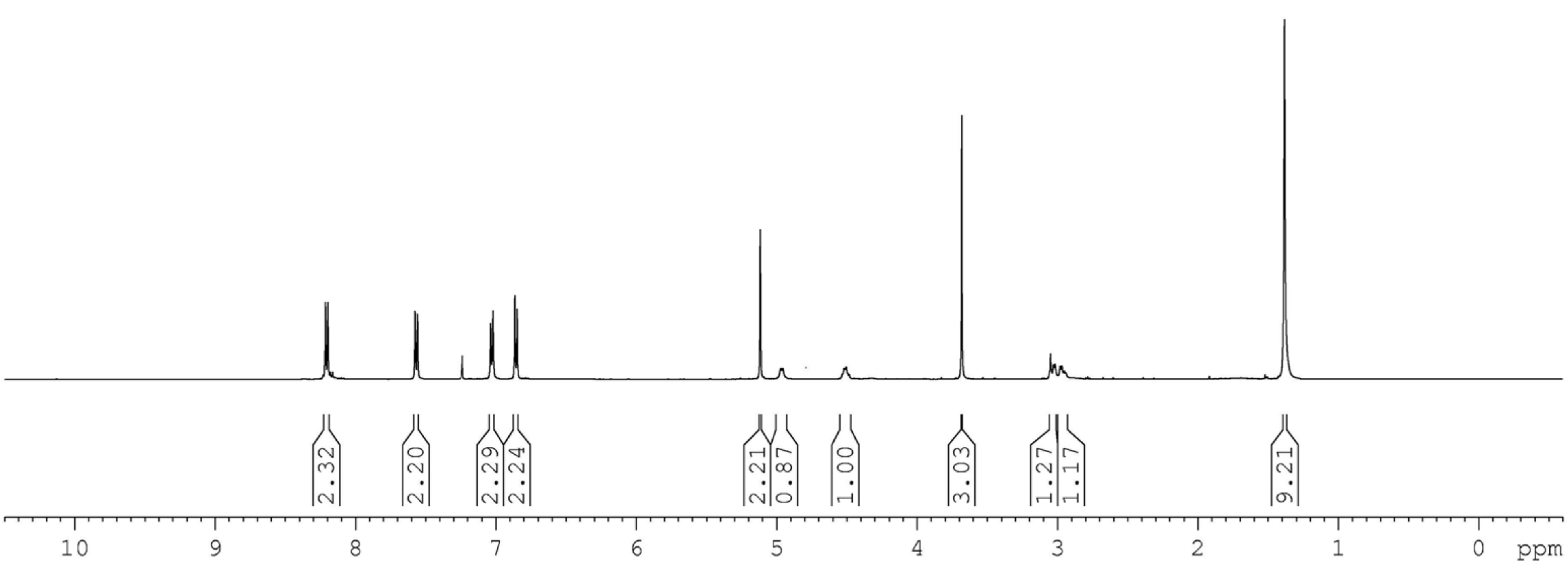

Solvent: $CDCl_3$ & NMR: 125 MHz

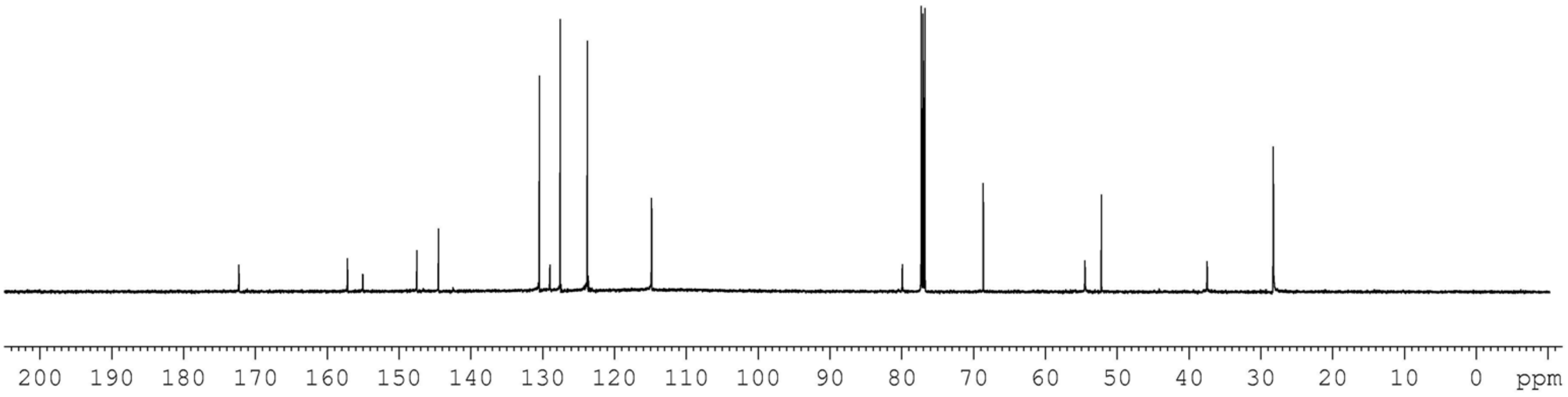

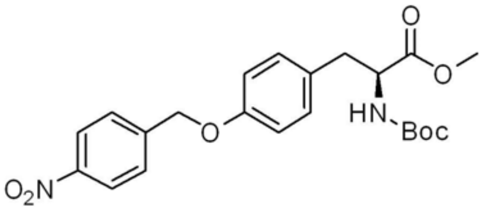

Chemical Formula: $C_{22}H_{26}N_2O_7$
Molecular Weight: 430.45

Solvent: $CDCl_3$ & NMR: 400 MHz

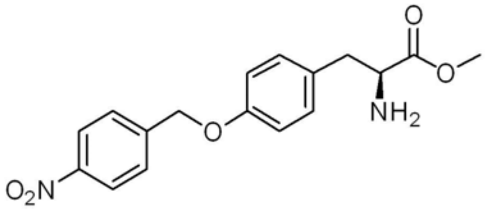

Chemical Formula: $C_{17}H_{18}N_2O_5$
Molecular Weight: 330.34

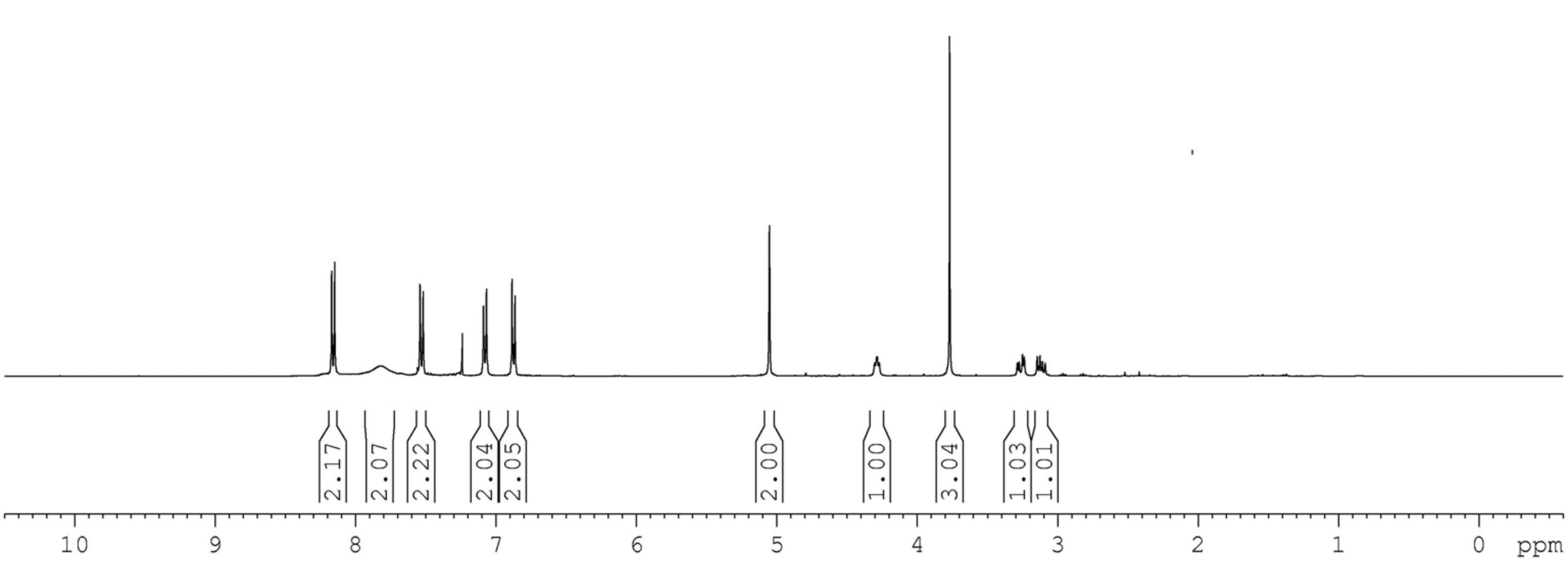

Solvent: $CDCl_3$ & NMR: 100 MHz

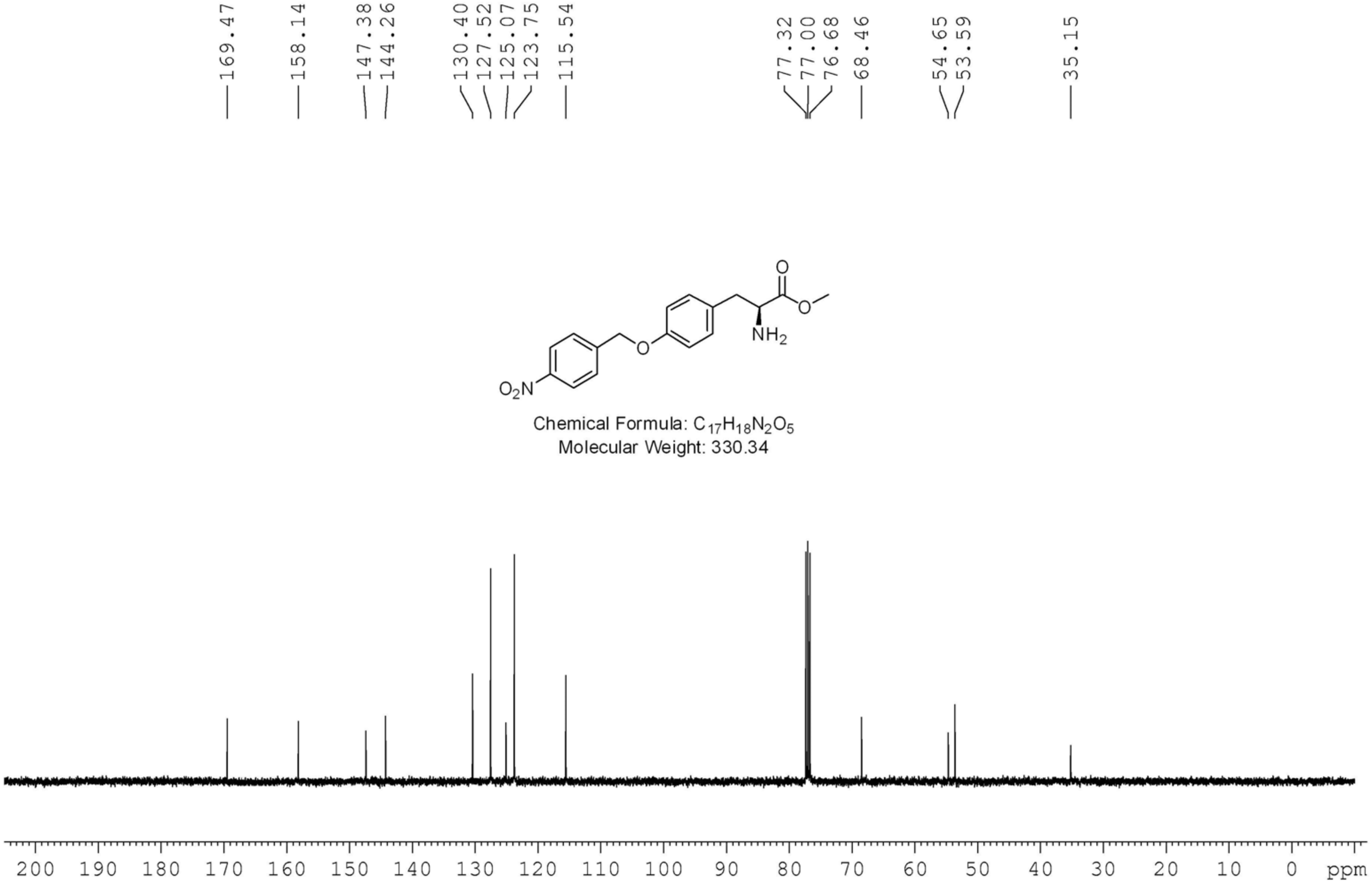

Solvent: $CDCl_3$ & NMR: 400 MHz

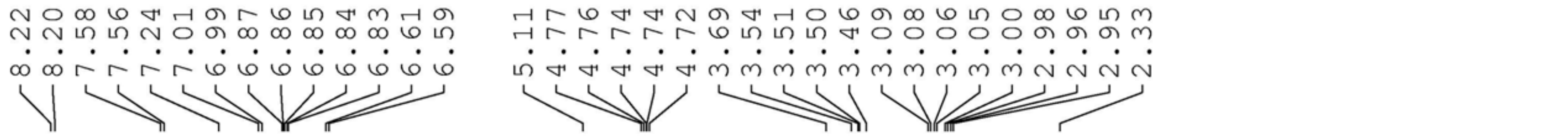

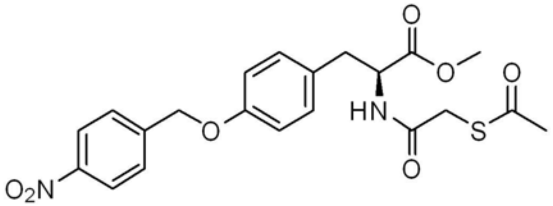

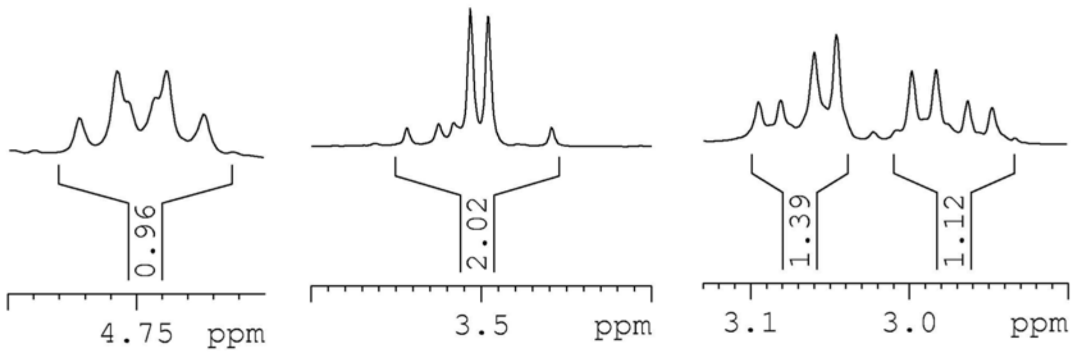

Chemical Formula: $C_{21}H_{22}N_2O_7S$
Molecular Weight: 446.47

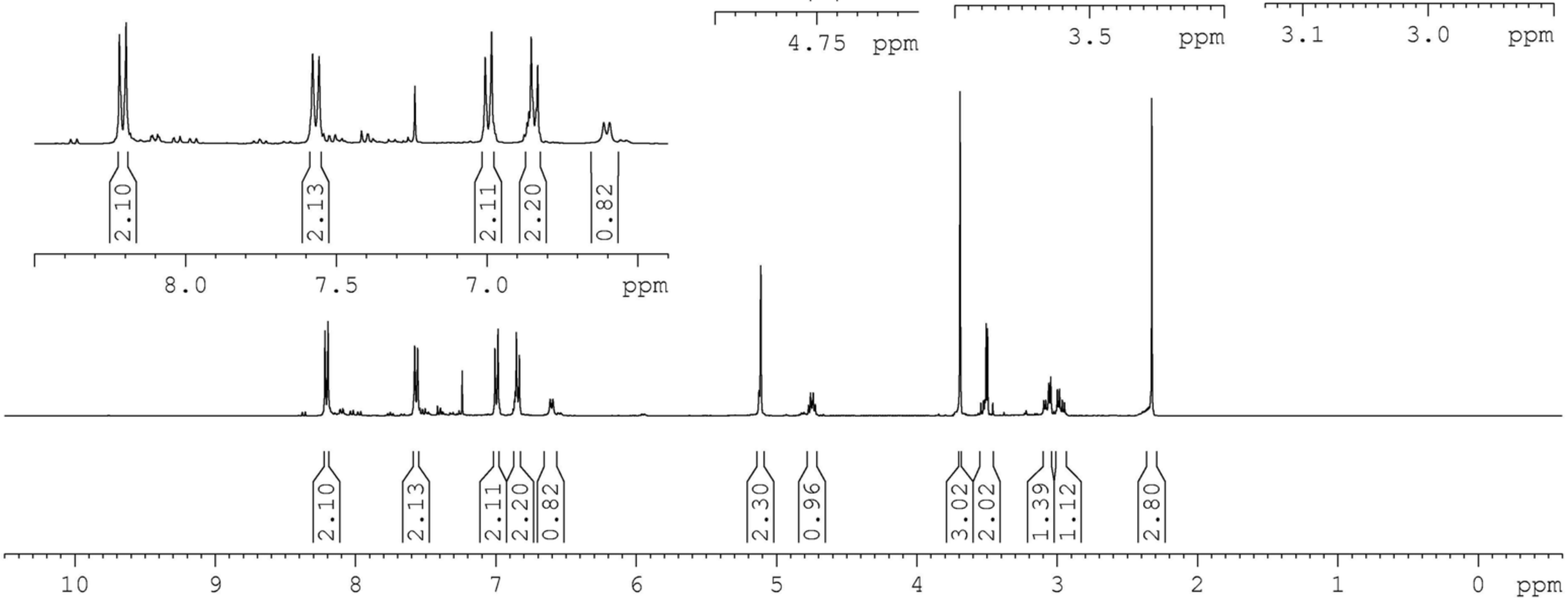

Solvent: $CDCl_3$ & NMR: 100 MHz

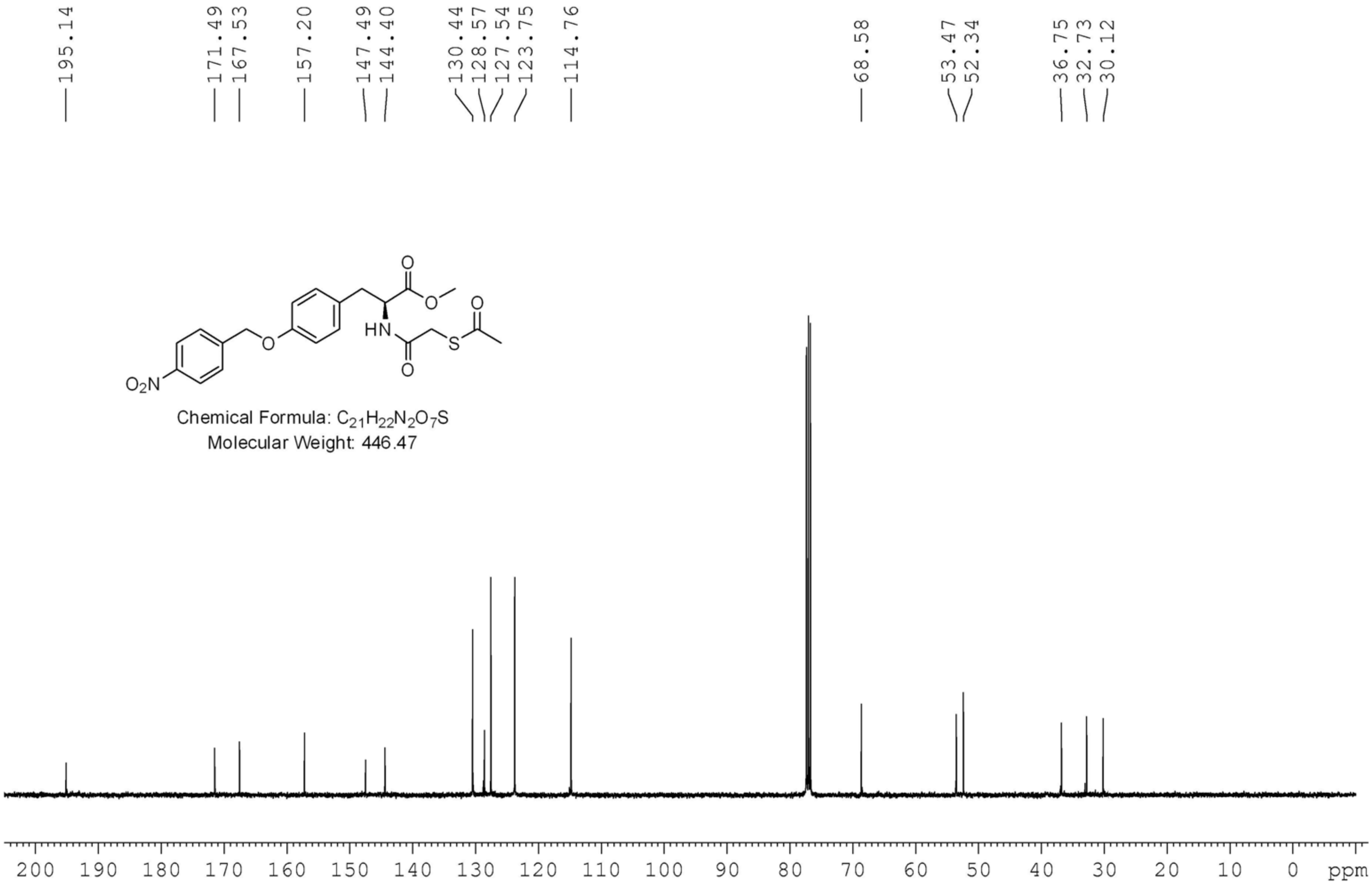

Solvent: $CDCl_3$ & NMR: 400 MHz

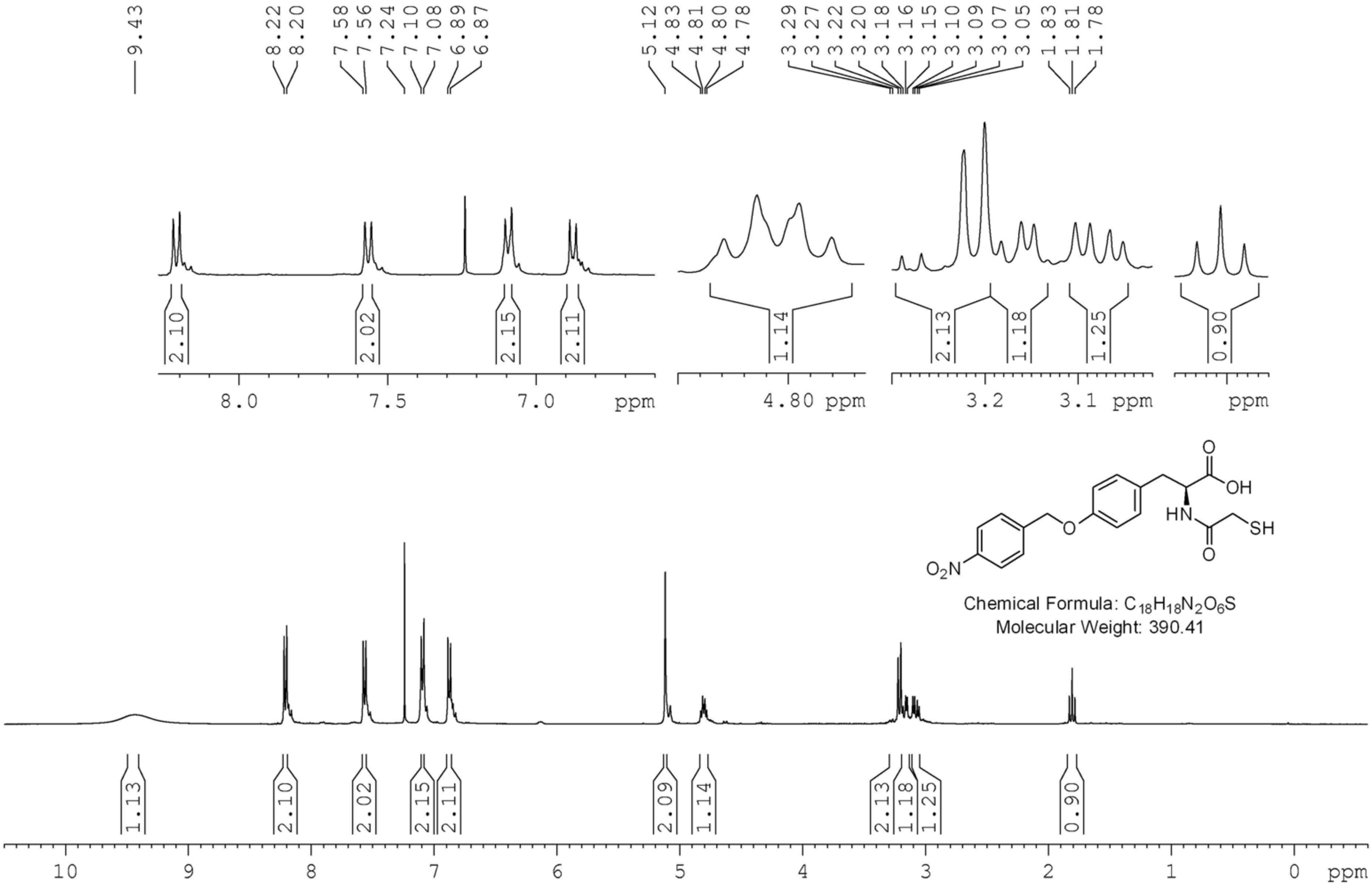

Solvent: $CDCl_3$ & NMR: 100 MHz

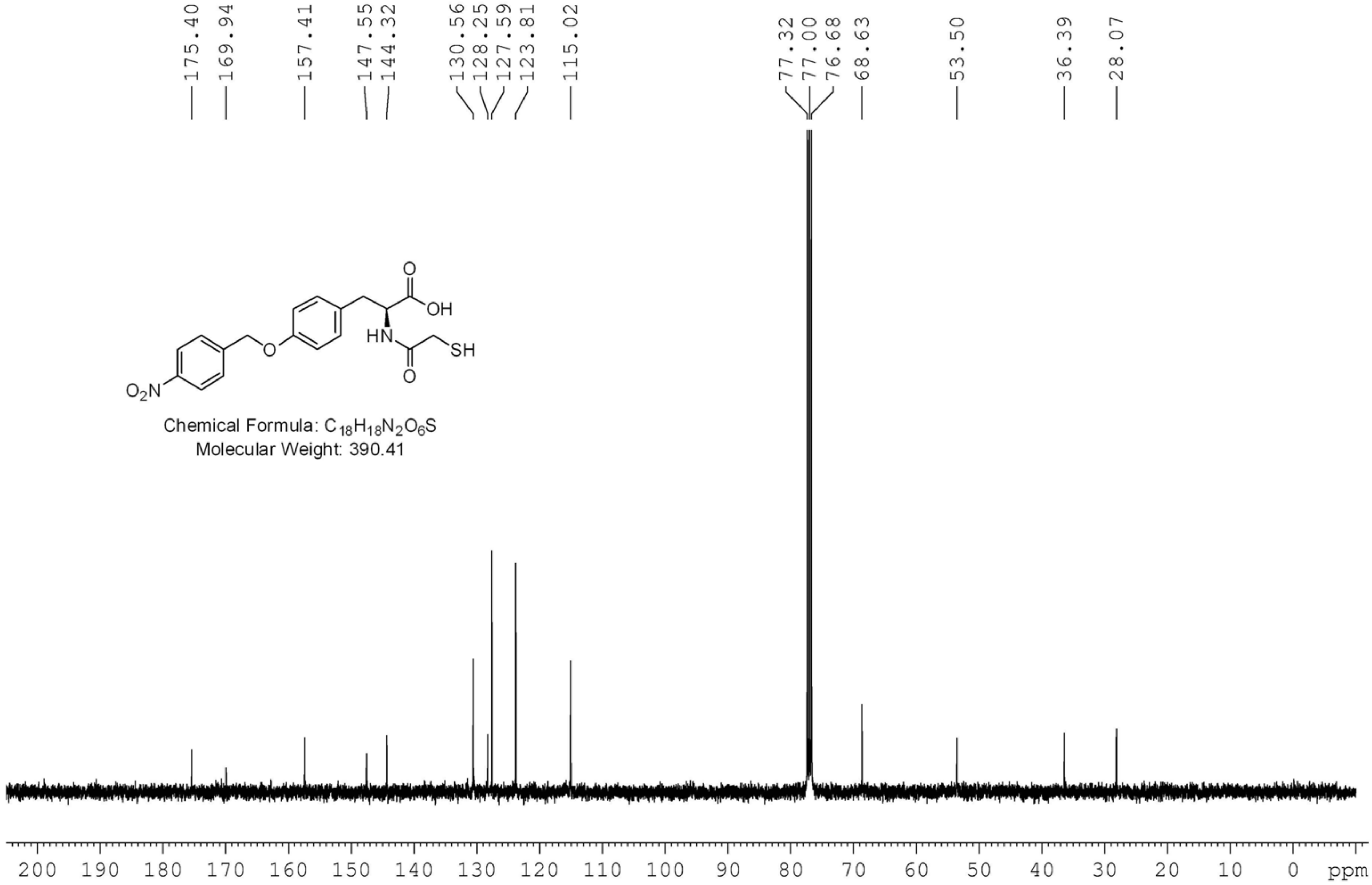

Chemical Formula: $C_{18}H_{18}N_2O_6S$
Molecular Weight: 390.41

Solvent: $CDCl_3$ & NMR: 500 MHz

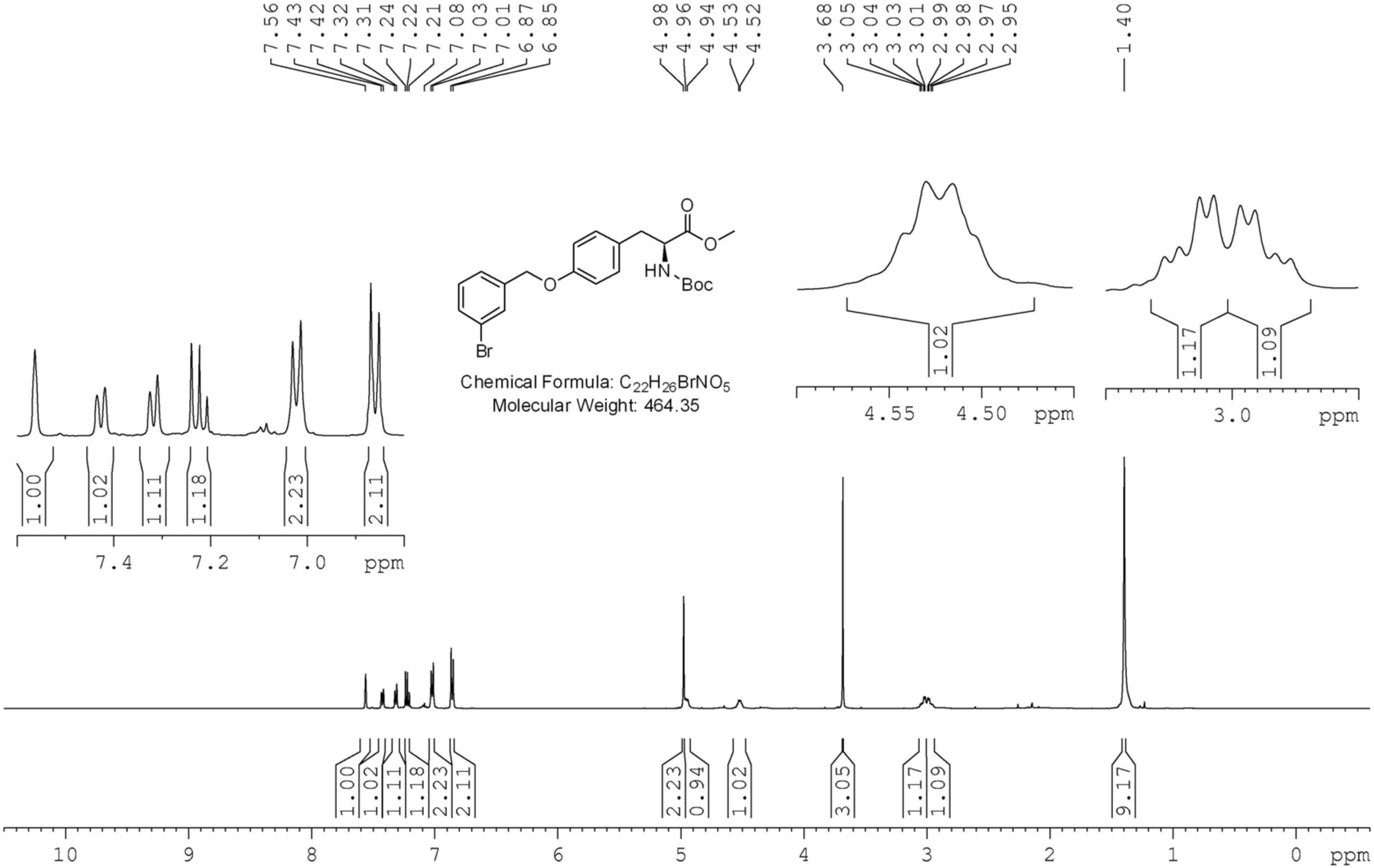

Solvent: $CDCl_3$ & NMR: 125 MHz

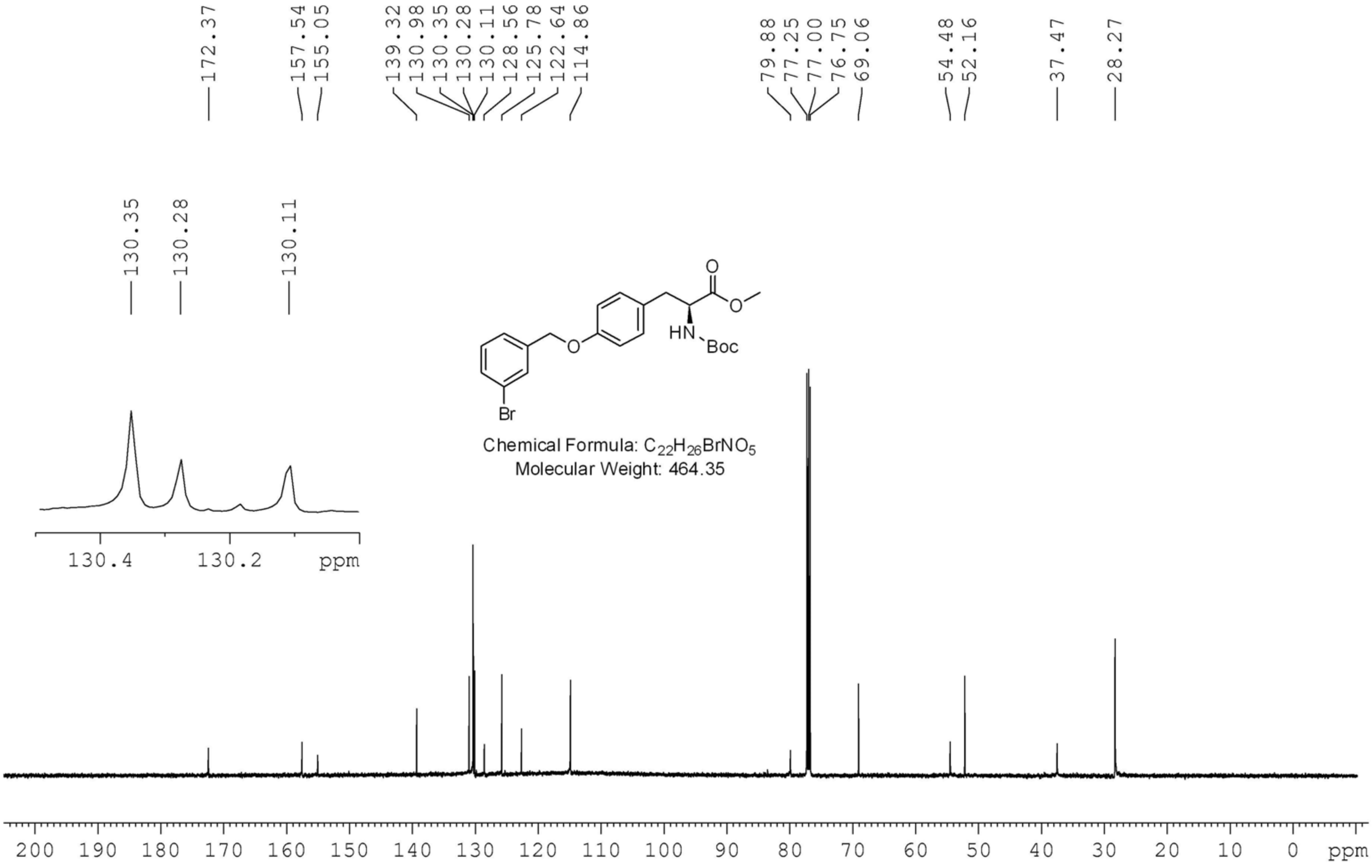

Solvent: CDCl3 & NMR: 500 MHz

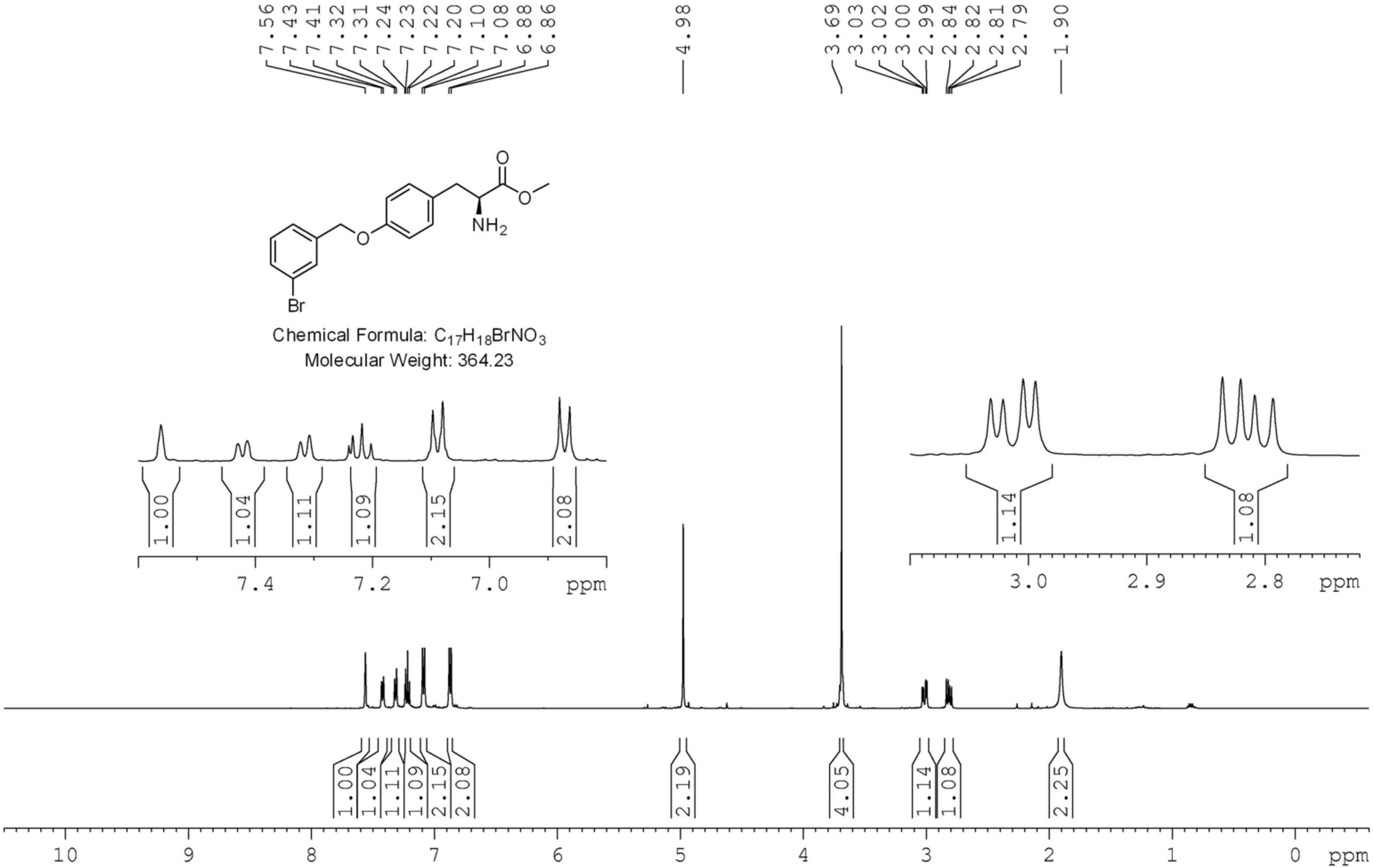

Solvent: $CDCl_3$ & NMR: 125 MHz

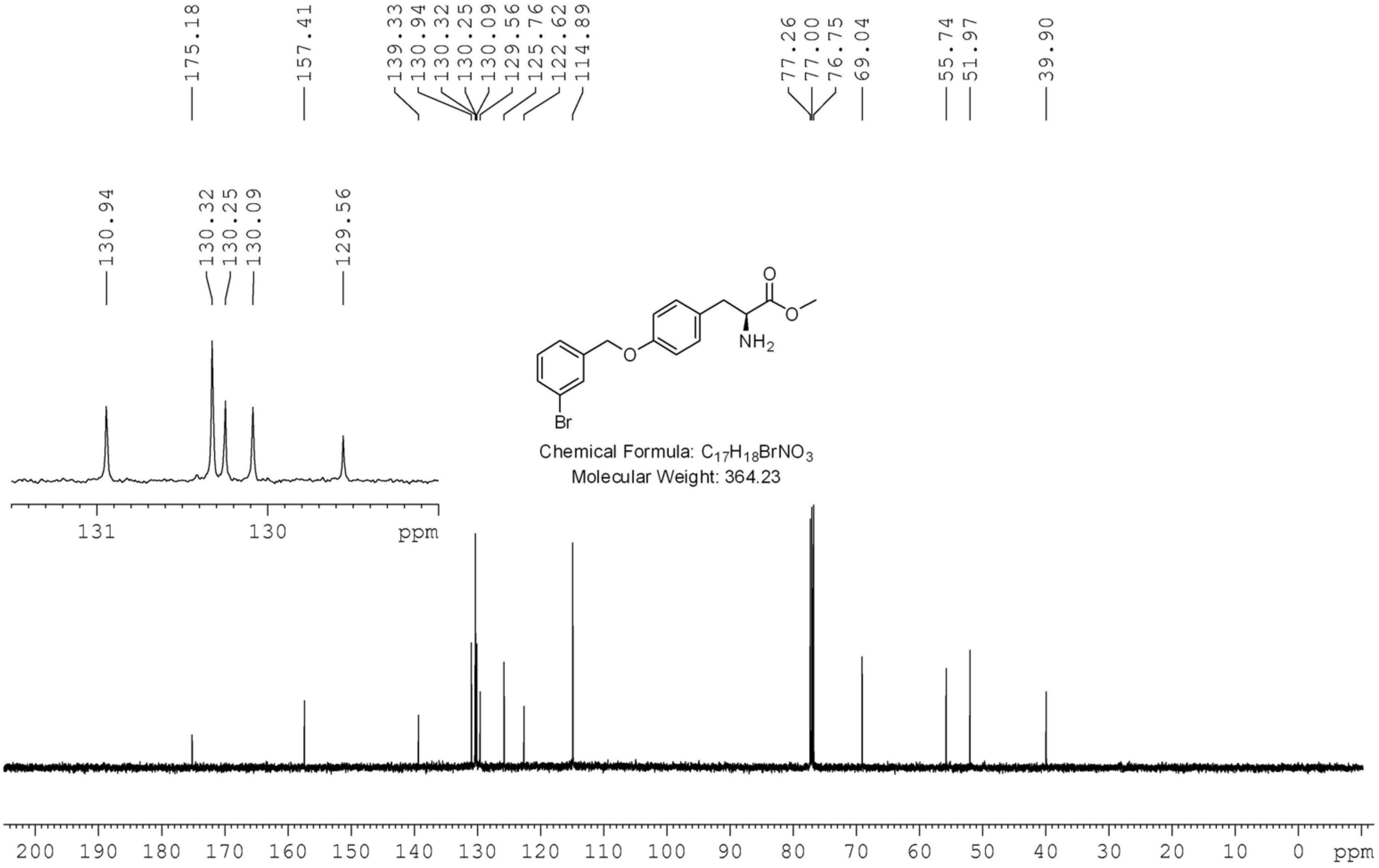

Chemical Formula: $C_{17}H_{18}BrNO_3$

Molecular Weight: 364.23

Solvent: $CDCl_3$ & NMR: 400 MHz

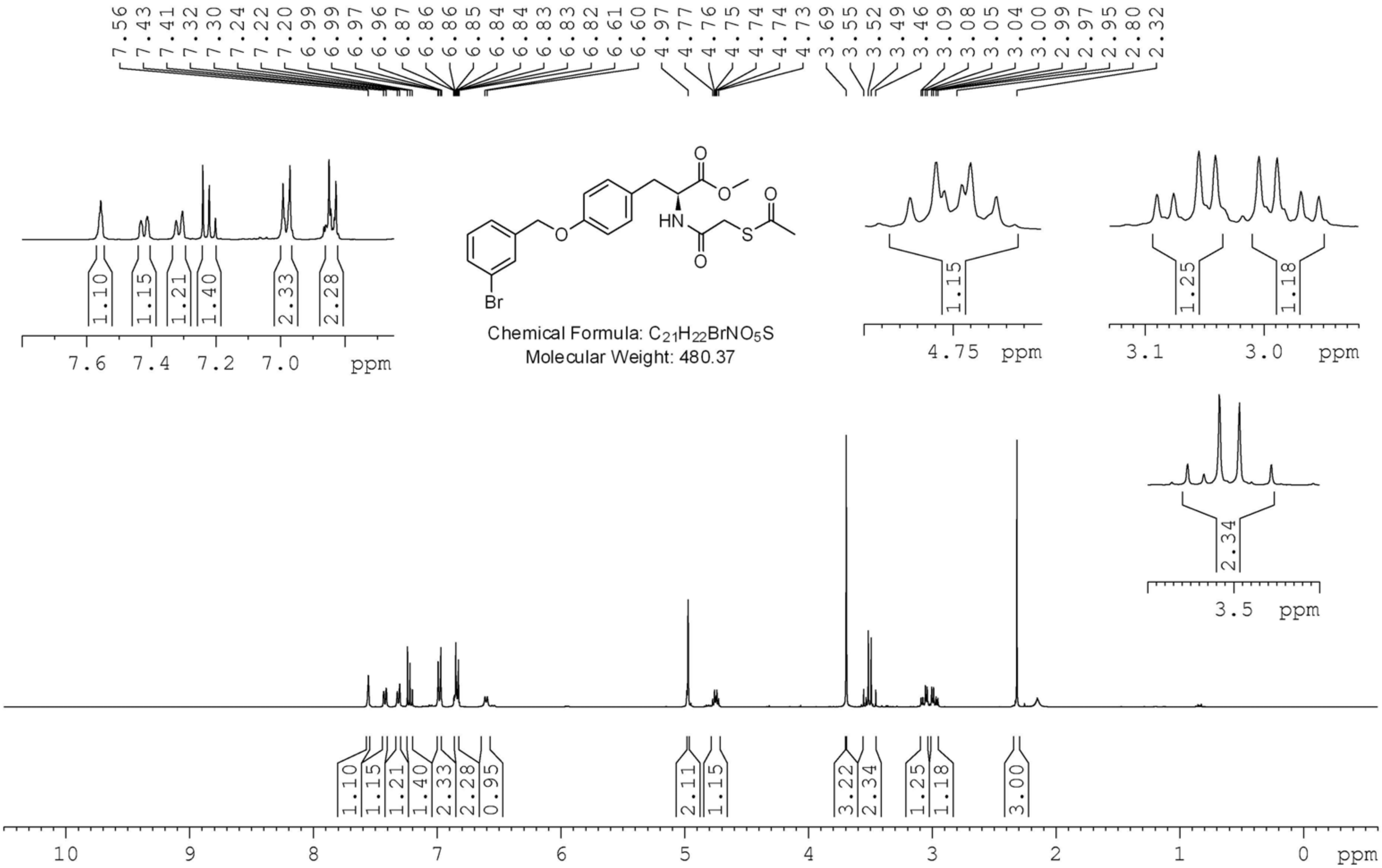

Chemical Formula: $C_{21}H_{22}BrNO_5S$

Molecular Weight: 480.37

Solvent: $CDCl_3$ & NMR: 100 MHz

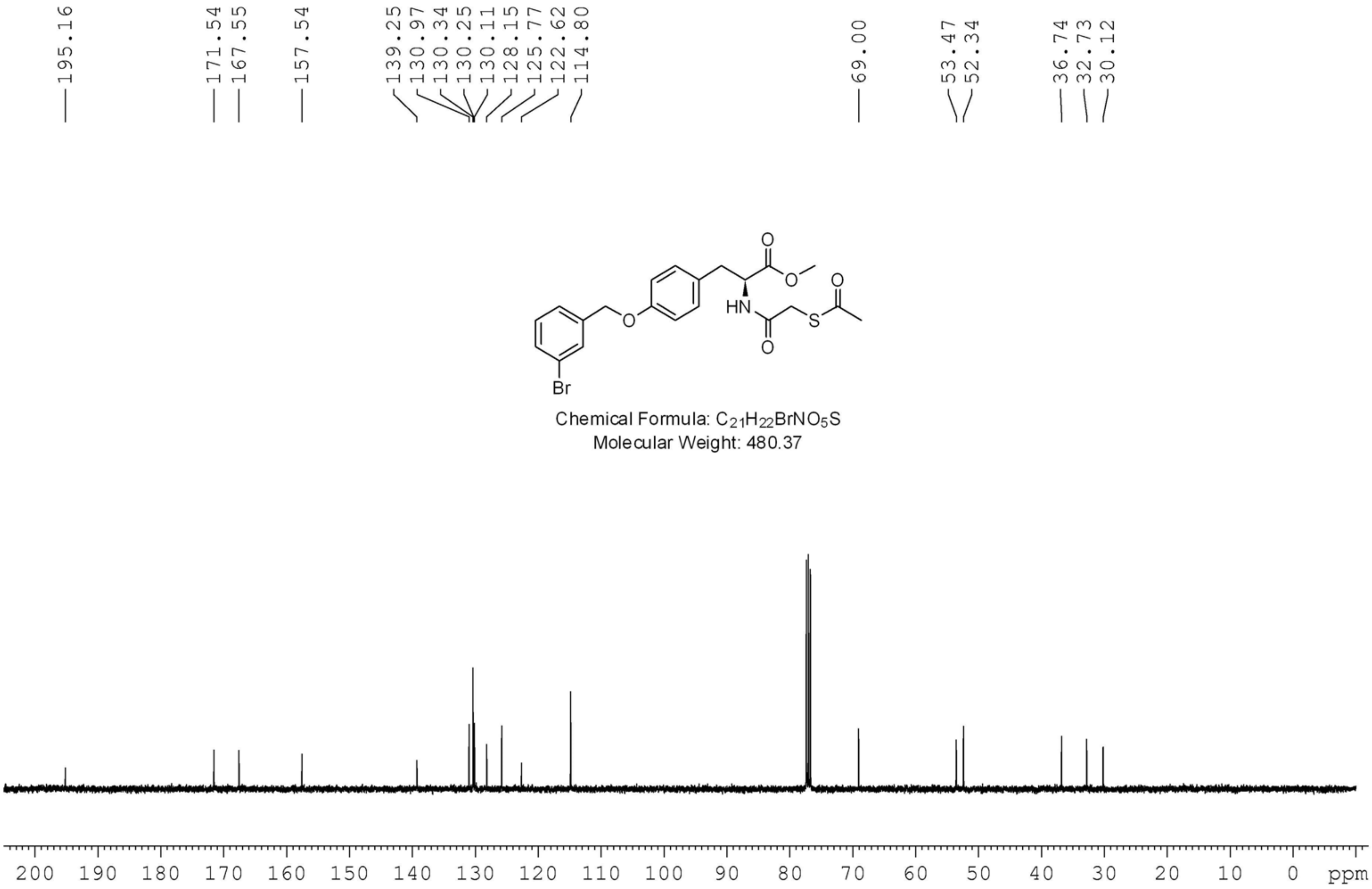

Chemical Formula: $C_{21}H_{22}BrNO_5S$

Molecular Weight: 480.37

Solvent: CD_3OD & NMR: 400 MHz

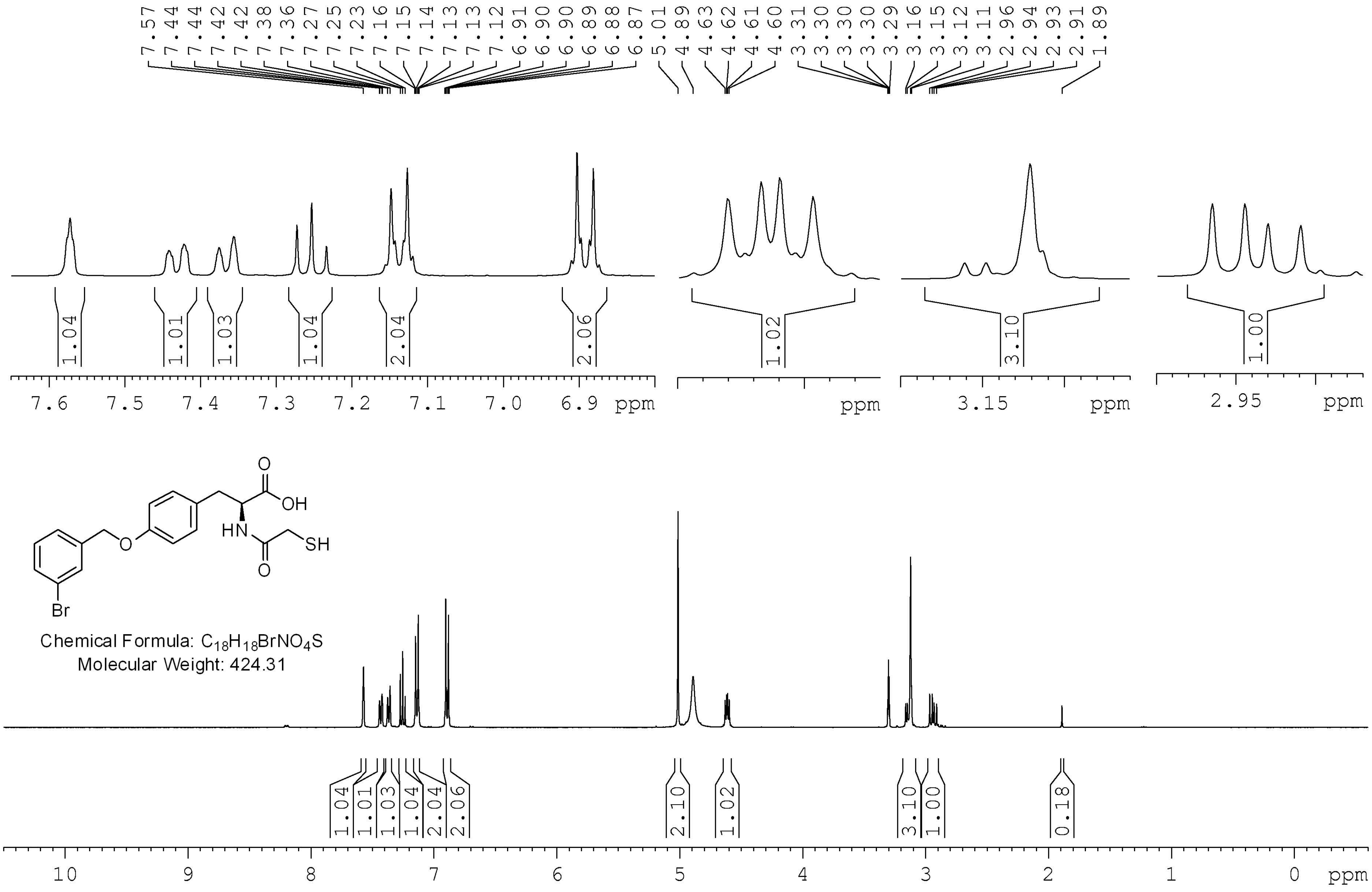

Chemical Formula: $C_{18}H_{18}BrNO_4S$
Molecular Weight: 424.31

Solvent: CD_3OD & NMR: 100 MHz

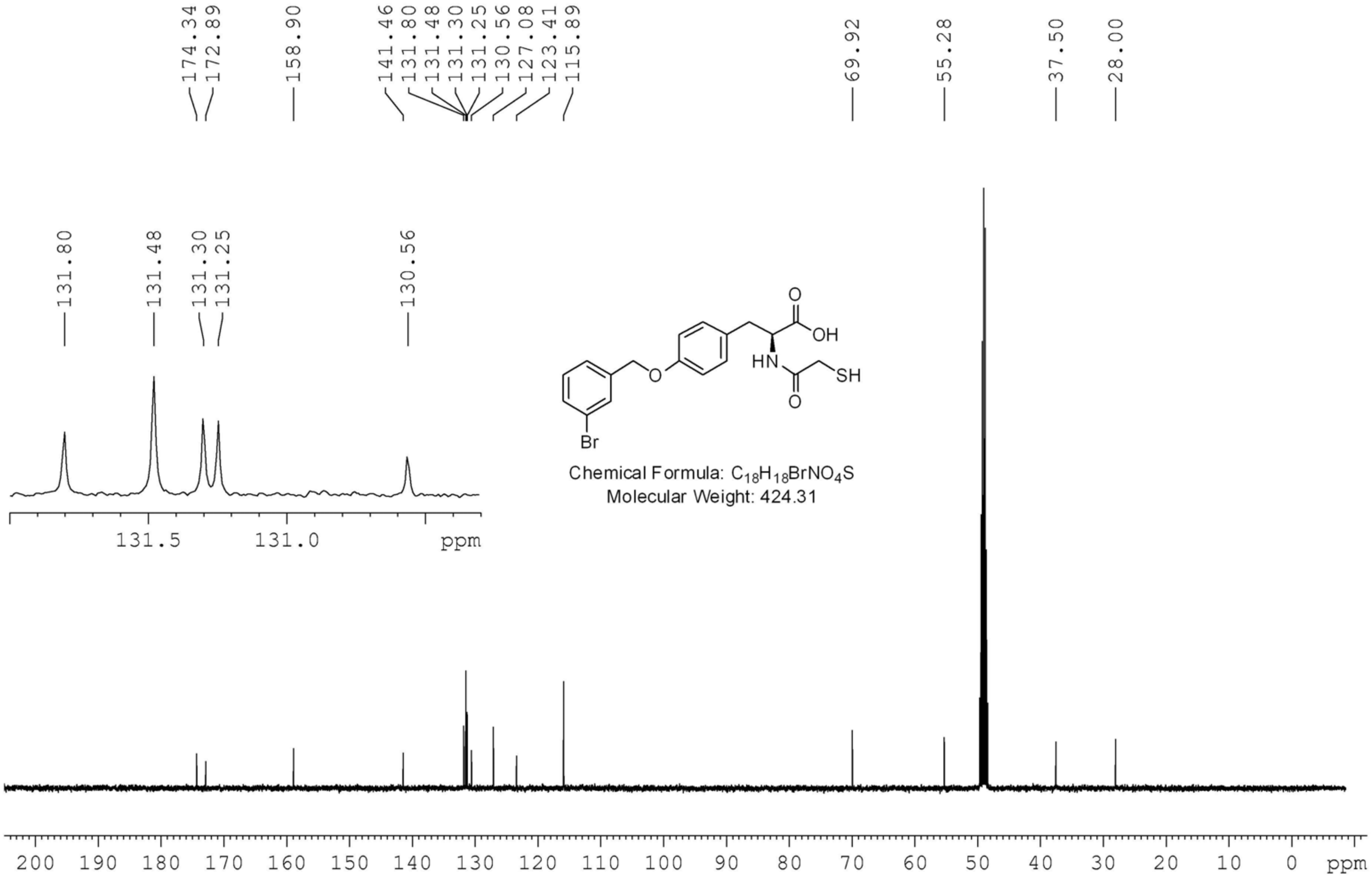

Solvent: $CDCl_3$ & NMR: 500 MHz

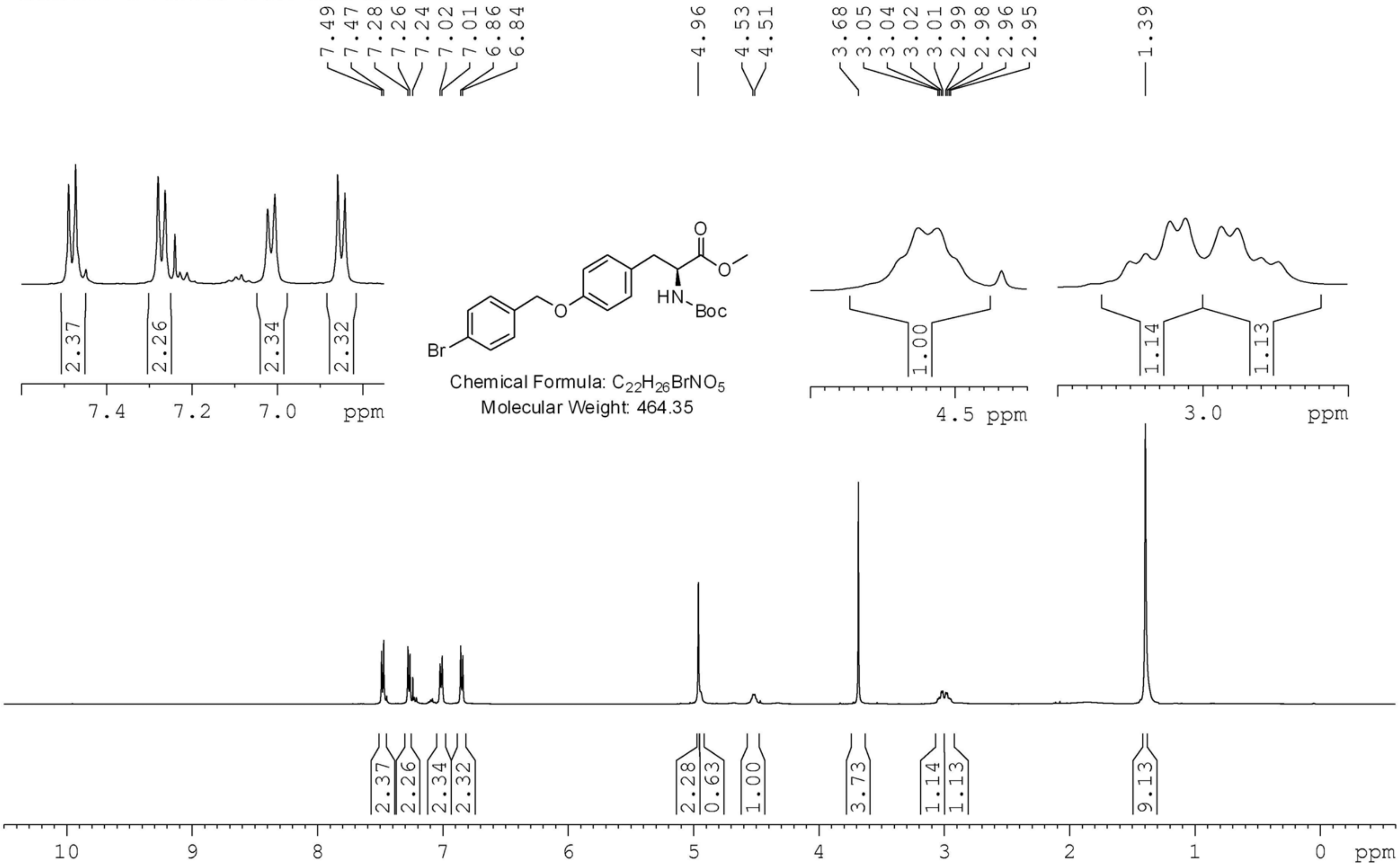

Solvent: $CDCl_3$ & NMR: 125 MHz

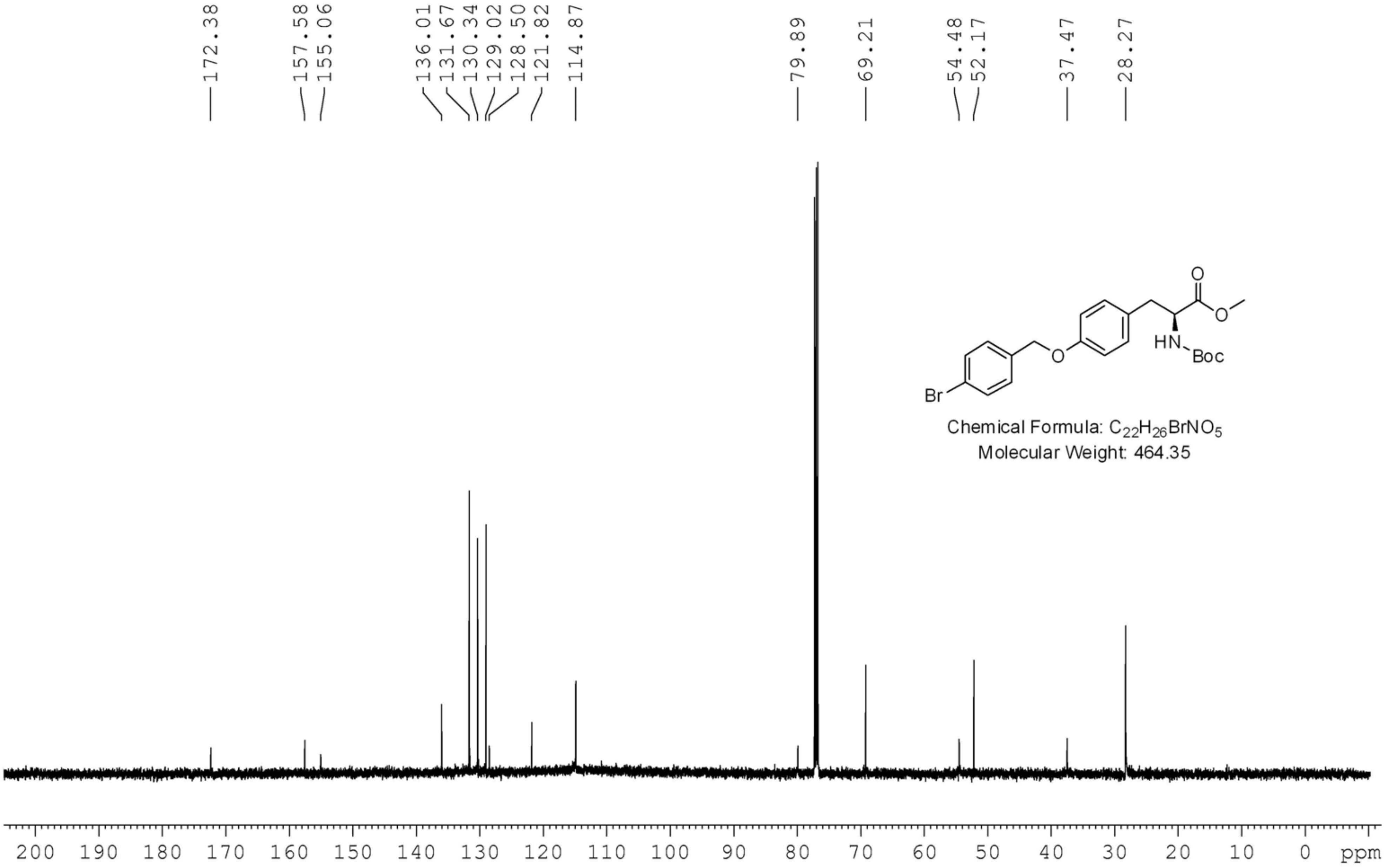

Solvent: $CDCl_3$ & NMR: 500 MHz

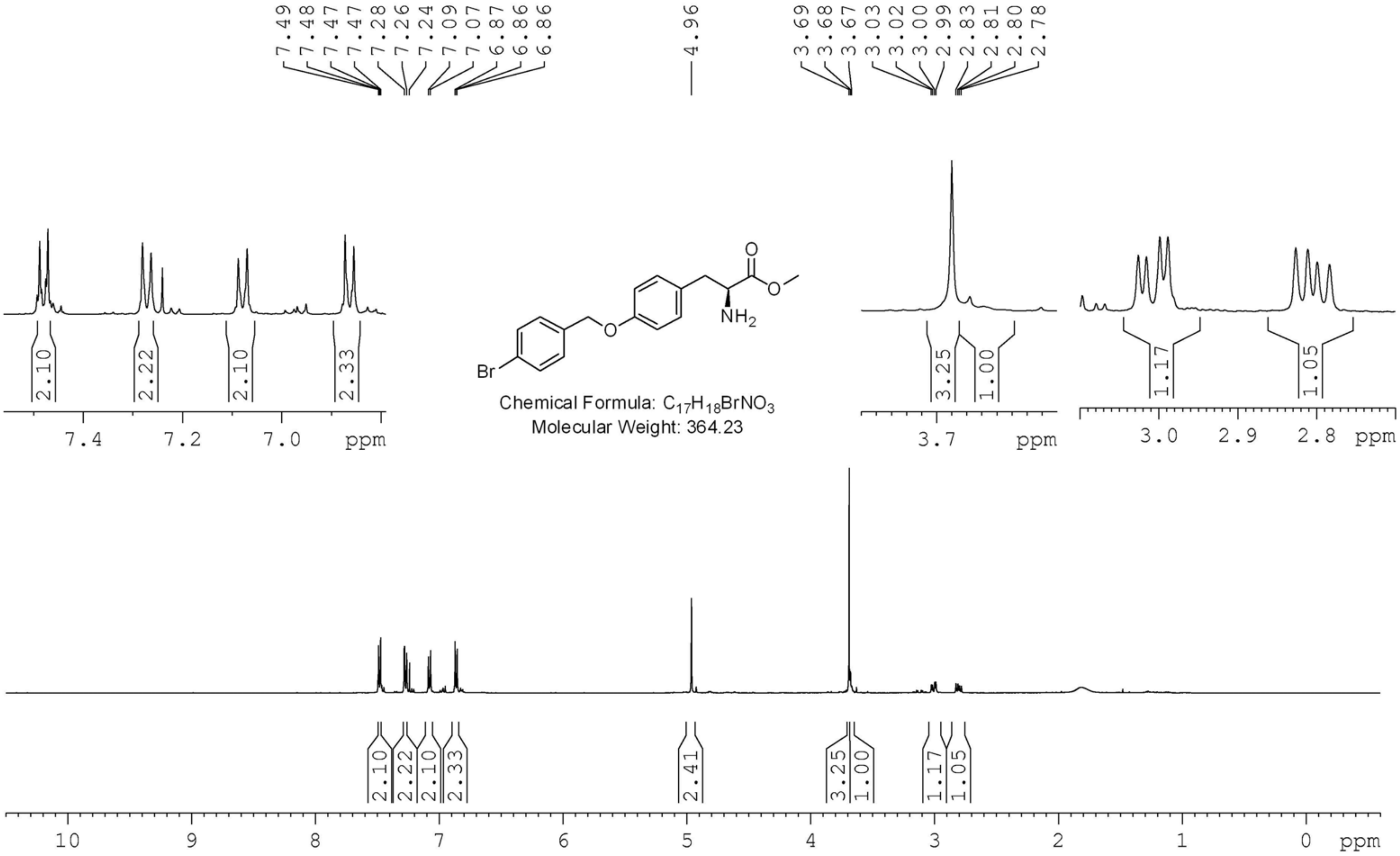

Solvent: $CDCl_3$ & NMR: 125 MHz

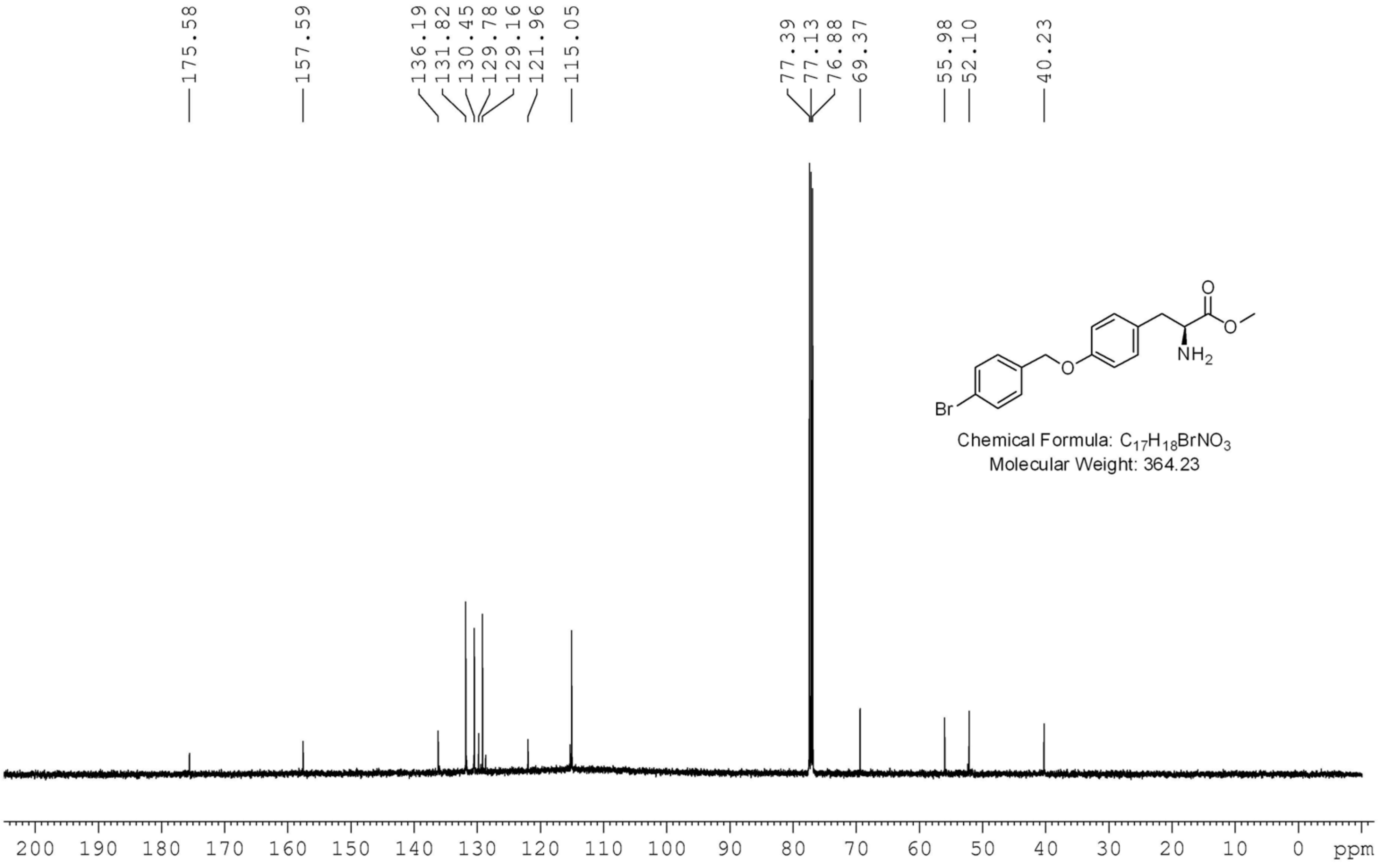

Chemical Formula: $C_{17}H_{18}BrNO_3$
Molecular Weight: 364.23

Solvent: $CDCl_3$ & NMR: 400 MHz

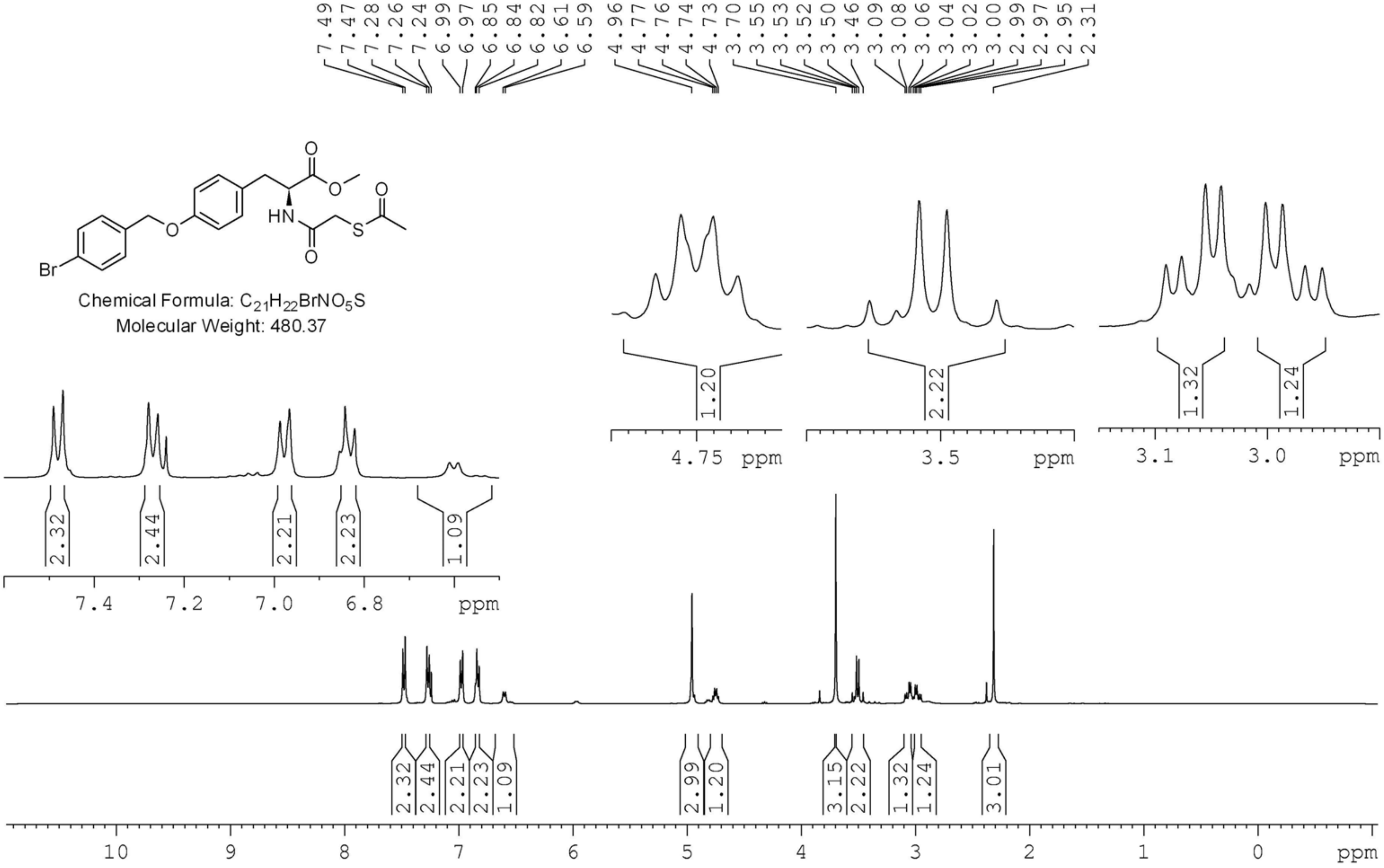

Solvent: $CDCl_3$ & NMR: 100 MHz

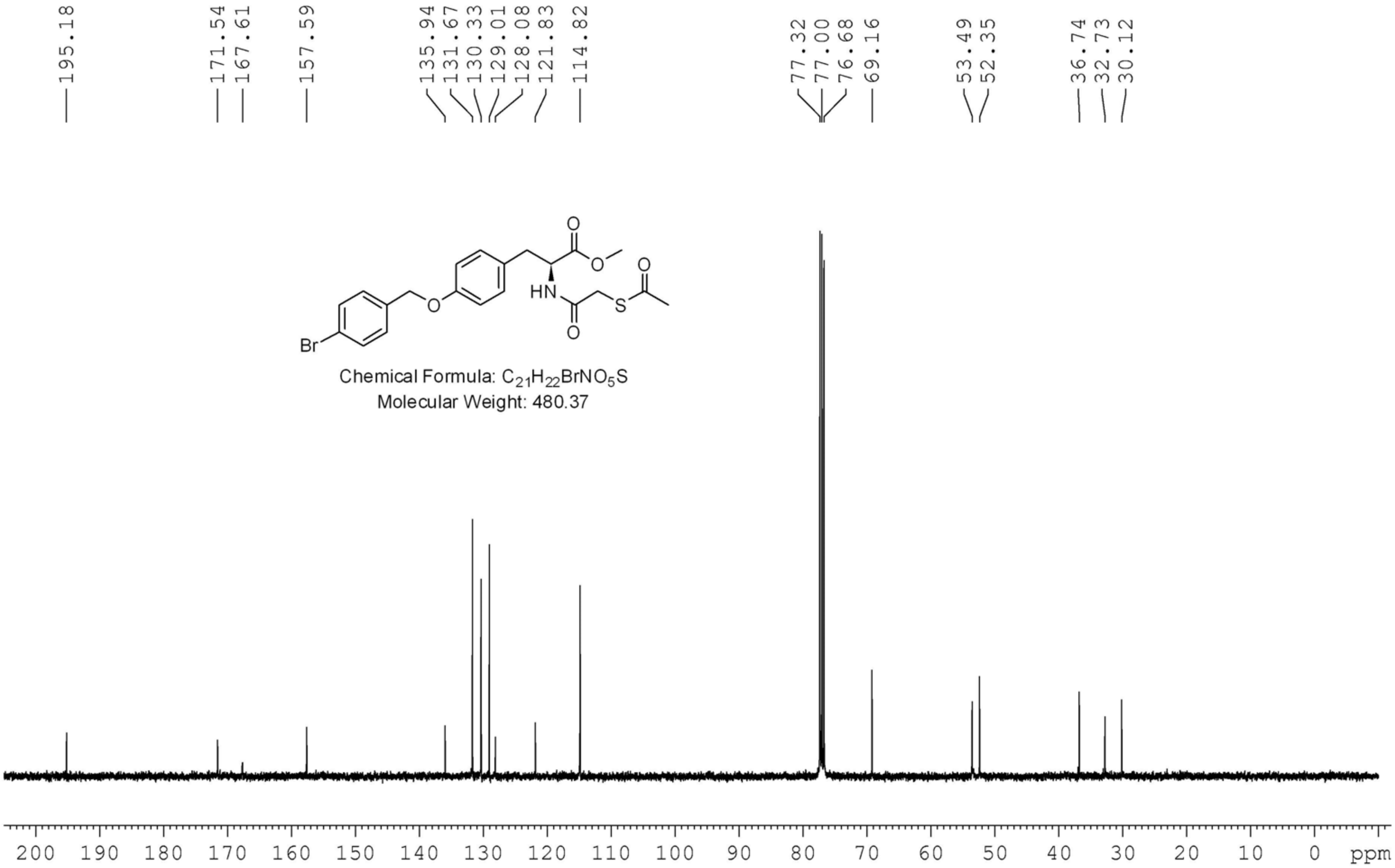

Solvent: CD_3OD & NMR: 500 MHz

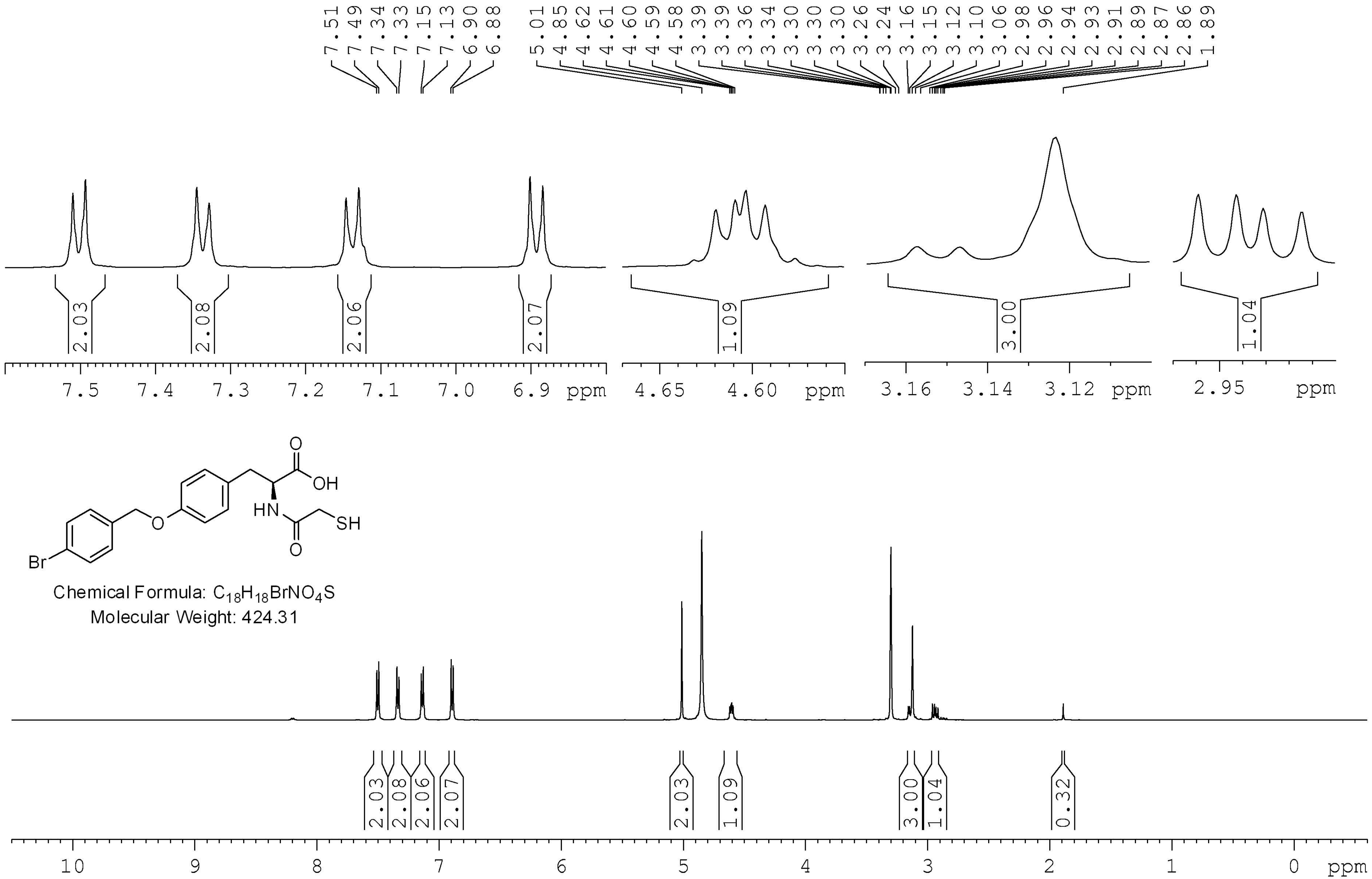

Chemical Formula: $C_{18}H_{18}BrNO_4S$
Molecular Weight: 424.31

Solvent: CD_3OD & NMR: 125 MHz

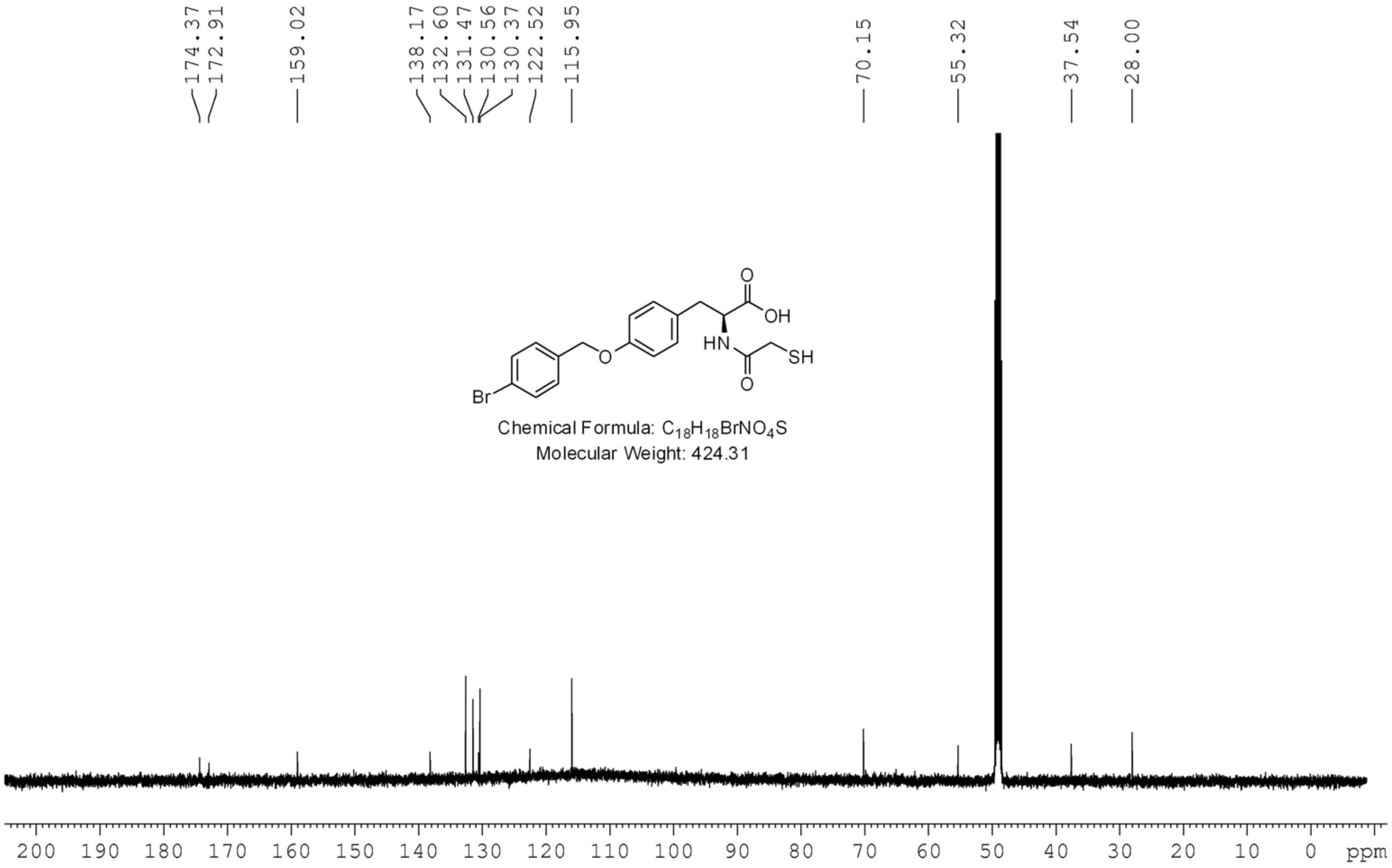

Supplement: Supplemental Material [file IENZ_A_1651314_SM7071.pdf]
